# EZH2 cooperates with E2F1 to stimulate expression of genes involved in adrenocortical carcinoma aggressiveness

**DOI:** 10.1038/s41416-019-0538-y

**Published:** 2019-07-31

**Authors:** Houda Tabbal, Amandine Septier, Mickael Mathieu, Coralie Drelon, Stéphanie Rodriguez, Cyril Djari, Marie Batisse-Lignier, Igor Tauveron, Jean-Christophe Pointud, Isabelle Sahut-Barnola, Bruno Ragazzon, Guillaume Assié, Jérôme Bertherat, Anne-Marie Lefrançois-Martinez, Antoine Martinez, Pierre Val

**Affiliations:** 1 CNRS, UMR 6293, GReD, Inserm U1103, Université Clermont Auvergne, 63001 Clermont-Ferrand, France; 2 Centre Hospitalier Universitaire, Service d’Endocrinologie, Faculté de Médecine, 63000 Clermont-Ferrand, France; 3 Institut Cochin, Inserm U1016, CNRS UMR 8104, Université Paris Descartes UMR-S1016, 75014 Paris, France

**Keywords:** Adrenal tumours, Cancer epigenetics

## Abstract

**Background:**

EZH2 is overexpressed and associated with poor prognosis in adrenocortical carcinoma (ACC) and its inhibition reduces growth and aggressiveness of ACC cells in culture. Although EZH2 was identified as the methyltransferase that deposits the repressive H3K27me3 histone mark, it can cooperate with transcription factors to stimulate gene transcription.

**Methods:**

We used bioinformatics approaches on gene expression data from three cohorts of patients and a mouse model of EZH2 ablation, to identify targets and mode of action of EZH2 in ACC. This was followed by ChIP and functional assays to evaluate contribution of identified targets to ACC pathogenesis.

**Results:**

We show that EZH2 mostly works as a transcriptional inducer in ACC, through cooperation with the transcription factor E2F1 and identify three positive targets involved in cell cycle regulation and mitosis i.e., *RRM2*, *PTTG1* and *ASE1/PRC1*. Overexpression of these genes is associated with poor prognosis, suggesting a potential role in acquisition of aggressive ACC features. Pharmacological and siRNA-mediated inhibition of RRM2 blocks cell proliferation, induces apoptosis and inhibits cell migration, suggesting that it may be an interesting target in ACC.

**Conclusions:**

Altogether, these data show an unexpected role of EZH2 and E2F1 in stimulating expression of genes associated with ACC aggressiveness.

## Background

Adrenocortical carcinomas (ACC) are endocrine malignancies associated with dismal prognosis. At diagnosis, 80% of patients present metastases reducing the 5 years survival rate below 30% for most series.

Although clinical management relies on complete surgical resection and mitotane treatment alone or in combination with chemotherapy, surgery remains ineffective in patients with metastatic disease.^1^ It is thus essential to identify the contributors of malignancy in order to develop more effective therapeutic approaches.

Molecular analyses have shown three molecular mechanisms predominantly altered in ACC. These alterations include inactivating mutations of the *TP53* gene associated with loss of heterozygosity of the locus in 25–35% of ACC,^2,3^ suggesting that P53 is involved in ACC pathogenesis. Consistent with this idea, inactivation of both P53 and RB in the adrenal cortex of mice expressing the simian virus 40 (SV40) large T antigen (AdTAg mice) results in metastatic ACC development.^4^ Other frequent alterations found in ACC result in overexpression of IGF2 (90% of ACC) and constitutive activation of the Wnt/β-catenin signalling pathway (about 40% of ACC).^2,3^ However, using transgenic mouse models, we have shown that these alterations, even when combined together, were not sufficient to induce malignant progression,^5–7^ indicating that further alterations are required to allow ACC progression.

Screening of epigenetic factors in publicly available transcriptome data from ACC patients identified the histone methyltransferase EZH2 as the most deregulated histone modifier in adrenal cortex cancer. We have also shown that EZH2 overexpression was associated with tumour progression and poor prognosis in ACC^8^ in agreement with other cancers.^9^ Overexpression of EZH2 in ACC patients was also consistent with the high expression of EZH2 detected in AdTAg mice during tumour progression.^4^

Although EZH2 was initially described as the catalytic core protein of the polycomb repressive complex 2 (PRC2), initiating target genes silencing by promoting H3K27 trimethylation,^10^ emerging literature suggests that EZH2 can work as a transcriptional activator by directly interacting with transcription factors, such as STAT3 and AR.^9,11,12^

In this paper, we show that EZH2 is essentially associated with stimulation of transcription and that this positive effect is the result of a functional cooperation between EZH2 and the transcription factor E2F1 in ACC. We further show that this interaction results in the upregulation of three genes implicated in cell cycle regulation and mitosis i.e., *RRM2*, *PTTG1* and *Ase1/PRC1*, that are associated with poor prognosis, suggesting a potential role in acquisition of aggressive ACC features. Interestingly, pharmacological and siRNA-mediated inhibition of RRM2 blocks cell proliferation, induces apoptosis and inhibits cell migration, suggesting that it may be an interesting target in ACC. Altogether, these data show an unexpected role of EZH2 and E2F1 in stimulating expression of genes associated with ACC aggressiveness.

## Methods

### Patients cohorts

The clinical datasets used in this paper are derived from three cohorts of patients. Cochin’s cohort (47 ACC, 41 ACA and 4 NAd, GSE49280^2^) and Michigan’s cohort (33 ACC, 22 ACA and 10 NAd, GSE10927^13^), were analysed by Affymetrix microarray (HG-U133 Plus 2.0 and HG-U95Av2, respectively). Data analysed were provided by our collaborators after RMA normalisation and inter-array normalisation. The third transcriptome (74 patients) is from the TCGA database (The Cancer Genome Atlas,^3^) and it relies on Illumina HiSeq 2000 RNA Sequencing Version 2. Expression data were standardised by the Relative Standard Error of the Mean (RSEM) algorithm and transformed into Log2 in order to refocus and symmetrise values’ distribution. For each cohort, patients’ survival data were available and distribution in the good (C1B) and poor prognosis (C1A) groups, was defined by our collaborators on the basis of unsupervised clustering.^14^ Datasets were treated independently and intersected on the basis of gene names.

### Microarray analysis of gene expression

Transcriptome data from our mouse model of Ezh2 ablation (Ezh2-KO) in the adrenal were obtained and analysed as described in Mathieu et al.^15^

### Expression data analyses process

Correlation analyses were conducted in R using cor.test with parameter method=‘pearson’ (package stats v3.4.2). Significance was calculated using the method previously described and adjusted using the Benjamini-Hochberg correction (‘p.adjust’ function).

Survival curves were obtained by the Kaplan–Meier method (package ‘survival’ v2.41-3), which allows evaluation of significance for the outcome prediction model, based on mRNA expression. Differences between survival curves were assessed using the log-rank test.

Univariate and multivariate Cox proportional hazards regression analyses were performed to evaluate the association of gene expression levels and prognostic factors to overall patients’ survival.

Enrichment analyses were conducted by GSEA v3.0 software (http://software.broadinstitute.org/gsea/index.jsp). cPRC2 gene sets were previously defined in Mathieu et al.^15^

### Chip sequencing datasets

Chip sequencing data were obtained from various published sources and were downloaded from Gene Expression Omnibus (GEO) or Encyclopedia of DNA Elements (ENCODE) (Supplementary Table 1).

### Chip sequencing analyses process

All data were retrieved in .sra format and converted to .fastq (except for the E2F1 mark of the K562 cell line) using fastq-dump tool (v2.1.7). The reads were aligned on the reference genome GRCh38 using bowtie2 software (v2.2.6, default parameters). The resulting .sam files were converted to .bam and then sorted using samtools (v0.1.19). Specific peaks detection and identification of studied marks were obtained by Macs14 software (v1.4.2) (J Feng et al., 2012 protocol). To eliminate redundant reads densities, normalisation was carried out using input during software setup. Annotations for peaks with an FDR < 0.05 were performed under R with the ‘GenomicRanges’ package (v1.28.5), taking into account a ±2 Kb window around the CDS (coding DNA sequence). To illustrate the peaks, .wig files obtained through Macs14 software were converted to .Bigwig files using the wigToBigWig tool delivered by UCSC (http://hgdownload.soe.ucsc.edu/admin/exe/). Peaks were visualised with IGV software (version 2.3.91).

### Measure of cell growth and apoptosis

H295R cells were cultured as previously described^8^ and cell growth rate was determined as described in Drelon et al.^8^ For knockdown experiments, cells were counted 5 days after the first round of siRNA transfection. For pharmacological inhibition, cells were counted 5 days after initiation of treatment. These experiments were repeated at least four times with triplicate samples. Cell cycle distribution was determined by analysing propidium incorporation by flow cytometry. At least 20,000 events were measured for each sample. These experiments were repeated three times with triplicate samples. Caspase 3 activity was measured as described in Drelon et al.^8^

### Transfections and treatments

siRNA-mediated EZH2 and E2F1 knockdown was performed as previously described.^8^ siRNA sequences were: siGFP: ACUACCAGCAGAACACCCCUU; siEZH2: Silencer Select (s4917, Ambion Life Technologies); siE2F1 (sc-29297, Santa Cruz Biotechnology) and siRRM2 (106532, Thermofisher). For pharmacological RRM2 inhibition, H295R cells were treated with increasing doses (1, 2.5, 5, 7.5, 10, 12.5, 15) of GW8510 (G7791, Sigma) for 5 days in complete culture medium, which was changed every 24 h. Likewise, for pharmacological EZH2 inhibition, H295R cells were treated with 5 µM of DZNep (102052-95-9, Cayman Chemical) for 24–72 h in complete culture medium, which was changed every 24 h. Mitotane (Sigma-Aldrich) treatment was conducted in a similar way with 10 or 20 µM doses. In all experiments, control cells were treated with equivalent amounts of DMSO.

### Migration and clonogenic growth assays

Wound healing assays were performed as previously described.^8^ Invasion analyses were performed using Boyden chambers (Millicell) with 8 µm pore-sized membranes, coated with Matrigel (BD Bioscience). Control or GW8510 treated H295R cells (2.5 × 10^5^ cells) were seeded in the upper chamber in minimum medium DMEM-F12 medium (Invitrogen) containing penicillin/streptomycin (100 mg ml^−1^) and l-glutamine (2 mM). The lower chamber contained DMEM-F12 medium (Invitrogen) supplemented with penicillin/streptomycin (100 mg ml^−1^), l-glutamine (2 mM), 10% FBS (Biowest) and 5% SR3 (S2640, Sigma). Cells were incubated for 48 h before fixation using Paraformaldehyde 4% and staining with Haematoxylin. Clonogenic growth assays were performed as described in Drelon et al.^8^

### Protein extraction and western blot analyses

Western blots were performed as previously described^16^ and proteins were detected with antibodies described in Supplementary Table 2.

### Real-time quantitative PCR

RTqPCR analyses were performed as in Drelon et al.^8^ with primer pairs listed in Supplementary Table 3. Relative gene expression was normalised to ACTIN by the ΔΔCt method.

### Chromatin immunoprecipitation

ChIP experiments were performed as described in Drelon et al.^8^ except for fixation, which was performed by addition of 1.5 mM ethylene glycol bis(succinimidyl succinate) (EGS) for 30 min and of 1% formaldehyde for 10 min. Antibodies used were: EZH2 (C15310039, Diagenode), E2F1 (05–379, Millipore) and Rabbit IgGs (kch-504–250, Diagenode). Sequence enrichment was detected by real-time PCR with primers designed in the regulatory regions of RRM2, PTTG1 and PRC1 (Supplementary Table 3).

## Results

**Fig. 1:**
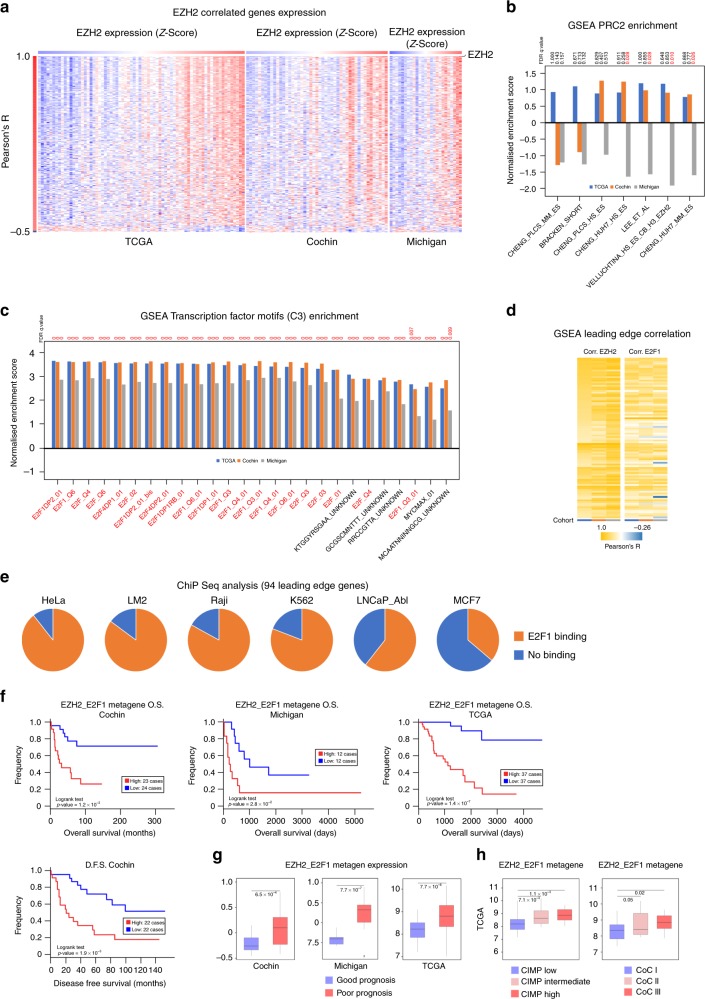
EZH2 may act as a transcriptional activator through potential cooperation with E2F1 in ACC. **a** Heatmap showing levels of expression of the genes with highest Pearson’s correlation with EZH2 (FDR < 0.001) in transcriptomes of three ACC cohorts (TCGA, Cochin, Michigan). Patients (columns) are organised according to increasing EZH2 expression shown as a z-score (from blue, low expression, to red, high expression). Genes (lines) are organised by decreasing Pearson’s R coefficient. **b** Correlation coefficients with EZH2 were used in a pre-ranked Gene set enrichment analysis (GSEA) to evaluate the presence of PRC2 targets in the genes correlated with EZH2. Curated lists of PRC2 targets were described in.^15^ **c** Correlation coefficients with EZH2 were used in a pre-ranked GSEA analysis to identify putative transcription factors binding sites in the promoters of the genes correlated with EZH2. Only gene sets with FDR < 0.01 were selected. Gene sets were extracted from MSigDB C3, which contains gene sets defined by the presence of putative transcription factor binding sites within their regulatory regions. **d** Genes constituting the leading edges of the 21 ‘E2F’ gene sets were extracted and intersected to build an EZH2/E2F core list of 94 genes. Their correlation coefficients with EZH2 and E2F1 were computed in the three cohorts of patients and represented as a heatmap. Columns represent the three cohorts, line represent correlation coefficients for each gene. **e** The capacity of E2F1 to bind to the regulatory regions (±2 kb from TSS) of the 94 leading edge genes was analysed in publically available E2F1 ChIP sequencing data obtained from six different cell lines. Pie charts represent the proportion of genes with (orange) or without (blue) E2F1 binding. **f** Patients’ overall survival (Cochin, Michigan, TCGA) and disease-free survival (Cochin) was analysed as a function of the mean expression of the 94 genes (EZH2_E2F1 metagene) by the Kaplan–Meier method. Statistical significance was evaluated by the Logrank test. **g** Expression of the metagene in ACC in the groups of good (blue) and poor prognosis (red). Statistical significance was evaluated by Wilcoxon’s test. **h** Expression of the metagene in ACC classified as CIMP low/intermediate/high and in clusters of clusters I, II and III.^3^ Statistical significance was evaluated by ANOVA

In order to identify EZH2 target genes in ACC, we performed correlation analyses on the basis of publicly available transcriptome data from Cochin, Michigan and TCGA cohorts, which represented a total of 146 cancer patients. Pearson R correlation coefficients between EZH2 and all genes were computed. After determination of a false discovery rate (FDR) threshold of 0.001, we selected genes that were correlated with EZH2 in all three cohorts. These data were then represented as a heatmap in which patients were ordered as a function of EZH2 expression and genes as a function of correlation coefficients (Fig. 1a). Unexpectedly, most genes that passed our selection criteria were positively correlated with EZH2 (*n* = 314/15701) and only a small proportion of genes were negatively correlated (*n* = 11/15701, difference in proportions F-test *p* value = 6.37 × 10^−79^) (Fig. 1a, Supplementary Table 4). Even though decreasing the stringency of our selection criteria (FDR < 0.01) increased the number of correlated genes, the proportion of positively correlated genes (653/15701) was still much higher than negatively correlated genes (172/15701, F-test *p* value = 2.2 × 10^–16^). This suggested that the main mode of action of EZH2 in ACC might not be associated with gene repression. This hypothesis was further supported by low enrichment for EZH2/PRC2 ChIP targets in the list of EZH2 correlated genes (Fig. 1b). Recent data suggest that EZH2 can function as a transcriptional activator by associating with a number of transcription factors (TF). To gain insight into which TF may be associated with EZH2-dependent gene regulation in ACC, we performed GSEA analysis using gene sets defined by the presence of conserved putative TF binding motifs within their promoters (MSigDB C3). Out of the 33 significantly enriched gene sets (FDR < 0.01), 21 (63%) were defined by the presence of putative E2F binding motifs (Fig. 1c). To further confirm the involvement of E2F transcription factors in the control of these genes, we extracted and intersected leading edge genes from the 21 gene sets. This represented a list of 94 potential targets (Supplementary Table 5). These 94 genes were highly correlated with both EZH2 and E2F1 expression in the three cohorts of patients (Fig. 1d). Analysis of publicly available ChIP sequencing data showed evidence of E2F1 binding within their regulatory regions (±2 kb from TSS) in HeLa (84/94), LM2 (80/94), Raji (78/94), K562 (76/94), LNCaP-Abl (57/94) and MCF7 cells (34/94) (Fig. 1e).

To gain insight into the potential involvement of these genes into ACC pathogenesis, we analysed patient survival as a function of the mean expression of the 94 genes (EZH2_E2F1 metagene). High expression of the metagene was associated with decreased overall survival (Cochin, TCGA) and shorter disease-free survival (Cochin) analysed as continuous variables (Wald test, TCGA O.S. *p* = 3.69 × 10^−^^5^, Cochin O.S. *p* = 2.74 × 10^−3^, Michigan O.S. *p* = 0.036, Cochin D.F.S. *p* = 3.33 × 10^−3^) or after dichotomisation according to median of expression (Fig. 1f). Cox proportional hazards regression analysis was then performed to further evaluate significance of high expression of EZH2_E2F1 metagene in predicting death. In this analysis, metagene expression, prognosis group (C1A vs C1B), expression of MKI67 (high vs low), Weiss score (4–9 vs 0–3) and ENSAT staging (3–4 vs 1–2) predicted overall survival as univariates in TCGA and Cochin cohorts (Supplementary Table 6). Metagene expression remained significant in most multivariate models but did not increase predictive power of other parameters (Supplementary Table 6). In further support of a potential role of the genes composing the metagene in ACC aggressiveness, we observed higher expression in the group of poor prognosis in the three cohorts (Fig. 1g) and in CIMP intermediate and CIMP high as well as in CoC II and CoCIII clusters of clusters (TCGA, Fig. 1h).

**Fig. 2:**
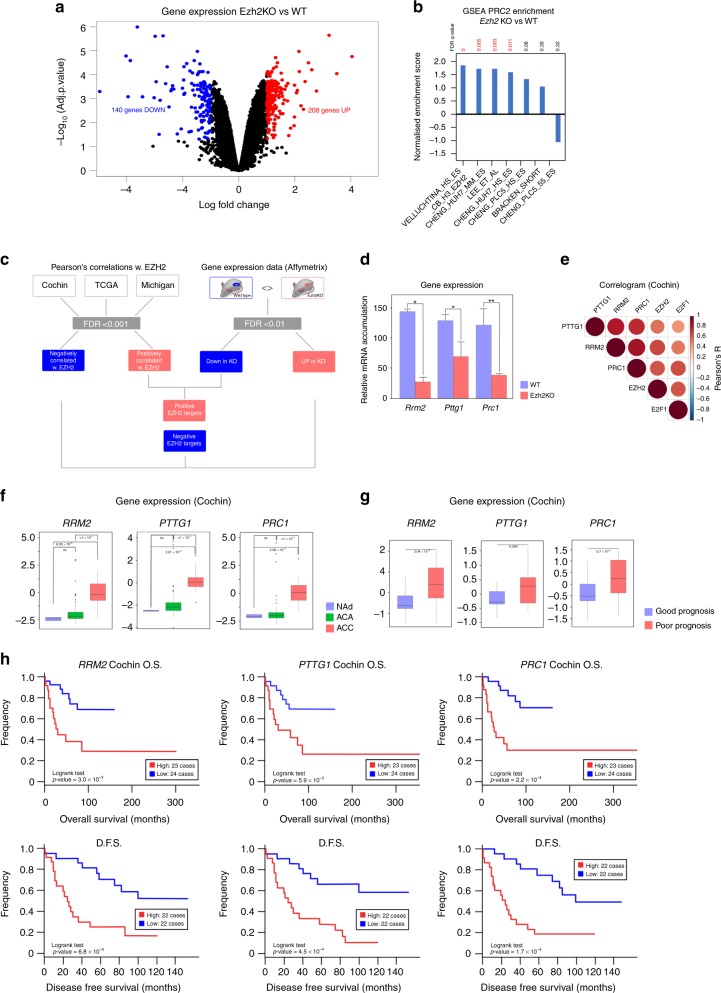
Identification of EZH2 target genes. **a** Differential gene expression was evaluated by micro-array analysis in a mouse model of adrenal-specific inactivation of Ezh2, compared with wild-type mice.^15^ Volcano plot represents Log Fold Change and –Log10 of adjusted *p*-value (FDR) for four knockout compared with three wild-type adrenals. Red dots show genes significantly up-regulated in knockout compared with wild-type adrenals. Blue dots show genes significantly down-regulated in knockout compared with wild-type adrenals. **b** GSEA evaluation of PRC2 targets enrichment in differentially expressed genes. **c** Strategy for identification of EZH2 targets in ACC by intersection of patients’ correlation data with mouse gene expression data. **d** Expression levels of the three identified positive target genes (*RRM2*, *PTTG1*, *PRC1*) were determined by RTqPCR in eight wild-type and eight Ezh2 knockout adrenals. Bars represent the mean ± SEM. Statistical analysis was conducted by Wilcoxon’s test. *p < 0.05, **p < 0.01 **e** Correlogram shows correlation of expression of *RRM2*, *PTTG1* and *PRC1* with expression of *EZH2* and *E2F1* in Cochin’s cohort. **f** Expression of *RRM2, PTTG1* and *PRC1* in normal adrenals, adrenocortical adenomas and adrenocortical carcinomas. Significance was evaluated by ANOVA. **g** Expression of *RRM2, PTTG1* and *PRC1* in the groups of good (blue) and poor (red) prognosis in Cochin’s cohort. Significance was evaluated by Wilcoxon’s test. **h** Overall (O.S) and disease-free (D.F.S) survival as a function of *RRM2*, *PTTG1* and *PRC1* expression in Cochin’s cohort. Statistical significance was evaluated by the Logrank test

Altogether, these data suggested that EZH2 might positively control expression of genes involved in ACC aggressiveness, through positive interaction with the E2F family of TF. To further refine the list of relevant EZH2 targets in the adrenal gland, we made use of transcriptome data obtained by analysis of a mouse model of adrenal-specific Ezh2 knockout. This model, which relies on excision of Ezh2 exons 16 to 19 by Sf1:Cre mediated recombination was recently characterised.^15^ Comparison of gene expression in three control and four knockout adrenals showed significant deregulation of 348 genes (FDR < 0.1, Abs(LogFC) > 1.0) upon inactivation of Ezh2 (Fig. 2a). In this physiological context, *Ezh2* ablation was essentially associated with gene overexpression (Fig. 2a) and positive enrichment of PRC2 target genes (Fig. 2b). This suggested that the main mode of action of EZH2 differed between normal and cancer cells in the adrenal gland. However, intersection of human and mouse data (Fig. 2c) resulted in the identification of *RRM2*, *PTTG1* and *PRC1* as three potential positive targets of EZH2 in the adrenal. Consistent with this, their expression was significantly decreased in knockout adrenals (Fig. 2d) and was positively correlated with EZH2 and E2F1 in all three cohorts of patients (Fig. 2e and Supplementary Fig. 1A).

*PTTG1* encodes Securin, a protein involved in the G2/M transition by inhibiting separation of sister chromatids^17^ but also in DNA repair^18^ and regulation of transcription.^19^ It is a putative oncogene in a number of tissues such as the pituitary (where it was isolated)^20^ and kidney^21^ and was shown to be overexpressed in ACC by an independent study.^22^

*PRC1*, also known as ASE1, encodes another key regulator of mitosis, which binds microtubules and is essential for the formation of the midzone bundle, central spindle organisation and subsequent cytokinesis.^23,24^ It is associated with cancer progression and WNT pathway activation in a number of tissues.^25,26^ RRM2 encodes one of the small subunits of ribonucleotide reductase (RR), the rate limiting enzyme for production of deoxyribonucleotides. RRM2 associates with RRM1 to form an active RR and its accumulation plays a central role in controlling RR activity.^27^ RRM2 is known to play an active role in tumorigenesis^28^ and is a prognostic factor for cancers such as colon, breast and pancreas.^29–31^ These data suggest that these three genes may be involved in ACC pathogenesis and aggressiveness.

Consistent with this idea, all three genes were markedly overexpressed in ACC compared with normal adrenals and ACA in both Cochin and Michigan cohorts (Fig. 2f and Supplementary Fig. 1B). This was further supported by the observation of higher expression of the three genes in the groups of poor prognosis in the three cohorts of patients (Fig. 2g and Supplementary Fig. 1C). Higher expression of *RRM2, PTTG1* and *PRC1* was also associated with significantly lower overall survival (Cochin, TCGA, Michigan) and lower disease-free survival (Cochin) (Fig. 2h and Supplementary Fig. 1D–E). Altogether, these data suggested that EZH2 positively controlled expression of three genes associated with ACC aggressiveness.

**Fig. 3:**
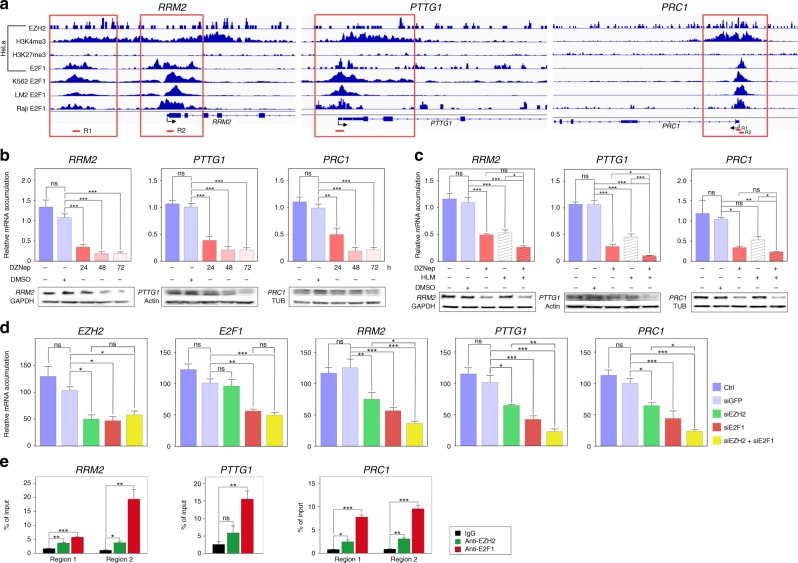
EZH2 and E2F1 cooperate to up-regulate expression of *RRM2*, *PTTG1* and *PRC1* in ACC. **a** Graphical representation of enrichment for EZH2, H3K4me3, H3K27me3 and E2F1 on the regulatory regions of *RRM2*, *PTTG1* and *PRC1* in HeLa, K562, LM2 and Raji cells. Red boxes show regions with EZH2, E2F1 and H3K4me3 enrichment, in absence of H3K27me3. Regions amplified by qPCR in **e** are shown as red segments under graphical representations. **b** Effect of 5 µM DZNep treatment for 24, 48 and 72 h on expression of RRM2, PTTG1 and PRC1 was evaluated by RTqPCR (graphs) and western blot (bottom panels) in H295R cells. **c** Effect of DZNep and/or HLM treatment for 48 h on expression of RRM2, PTTG1 and PRC1 was evaluated by RTqPCR (graphs) and western blot (bottom panels) in H295R cells. **d** Effect of EZH2 and/or E2F1 knockdown on expression of *RRM2*, *PTTG1* and *PRC1* was evaluated by RTqPCR after transfection of siRNAs targeting EZH2 and/or E2F1 in H295R cells. **e** Binding of EZH2 and E2F1 to the regulatory regions of *RRM2*, *PTTG1* and *PRC1* was evaluated by ChIP qPCR in H295R cells. Data are expressed as percent of enrichment over chromatin input. IgG were included as negative control. In **b**–**d** graphs represent the mean of five independent experiments ± SEM. In **e**, graphs represent the mean of three independent IP experiments ± SEM. Significance was evaluated by ANOVA in **b**–**e**. *p < 0.05, **p < 0.01, ***p < 0.001

Analysis of publicly available ChIP sequencing data showed strong E2F1 binding peaks in the regulatory regions of these three genes in HeLa, K562, LM2 and Raji cell lines (Fig. 3a). Interestingly, these E2F1 binding peaks were also associated with the H3K4me3 mark (active chromatin) and EZH2, in the absence of the H3K27me3 mark (inactive chromatin) in HeLa cells. This suggested that EZH2 and E2F1 could cooperate to activate rather than repress, transcription of *RRM2, PTTG1* and *PRC1* (Fig. 3a). To evaluate this hypothesis in adrenocortical cells, we treated H295R cells with DZNep, a compound that we previously used as an EZH2 inhibitor.^8^ Treatment with 5 µM DZNep for 24, 48 and 72 h resulted in a strong reduction in the accumulation of RRM2, PRC1 and PTTG1 mRNA and protein, suggesting that EZH2 controlled their expression positively (Fig. 3b). To evaluate the possibility that EZH2 cooperated with E2F1 to activate gene expression, we used HLM006474 (HLM), a compound that has been shown to inhibit E2Fs accumulation and transcriptional activity.^32^ Treatment with HLM alone induced a robust dose-dependent decrease in accumulation of RRM2, PRC1 and PTTG1 (Fig. 3c and Supplementary Fig. 2A), which was associated with decreased accumulation of E2F1 (Supplementary Fig. 2A and D), inhibition of H295R cell growth and deregulation of cyclins expression (Supplementary Fig. 2B–C). To further assess cooperation between EZH2 and E2F1, we combined inhibition of EZH2 with DZNep and inhibition of E2F with HLM. Consistent with our previously published data,^8^ inhibition of E2F with HLM resulted in decreased expression of EZH2 (Supplementary Fig. 2D). In contrast, DZNep had no effect on E2F1 expression ruling out a direct effect of EZH2 on E2F1 expression (Supplementary Fig. 2D). Consistent with a potential positive cooperation between EZH2 and E2F1, accumulation of RRM2, PRC1 and PTTG1 was further decreased upon treatment with HLM and DZNep (Fig. 3c). To further confirm cooperation between EZH2 and E2F1, we transfected H295R cells with siRNAs targeting EZH2 and E2F1 (Fig. 3d). Interestingly, transfection with either EZH2 or E2F1 siRNAs decreased mRNA accumulation of *RRM2*, *PTTG1* and *PRC1* (Fig. 3d). This decrease was further amplified by transfection of both siRNAs even though expression of EZH2 and E2F1 was not further altered by co-transfection of the two siRNAs (Fig. 3d). This further supported the idea that EZH2 and E2F1 cooperated to stimulate expression of these three targets. To further confirm this theory, we analysed the binding of these two factors by chromatin immunoprecipitation in H295R cells (Fig. 3e). Primer pairs used for real-time PCR quantification of enrichment were designed in zones of coincident EZH2 and E2F1 binding on the regulatory regions of the three genes in HeLa cells (Fig. 3a). These experiments showed significant enrichment of E2F1 on the regulatory regions of RRM2, PTTG1 and PRC1. This was associated with significant binding of EZH2 on the regulatory regions of RRM2 and PRC1. Although not significant, there was a trend for EZH2 recruitment on the regulatory regions of PTTG1 (Fig. 3e).

**Fig. 4:**
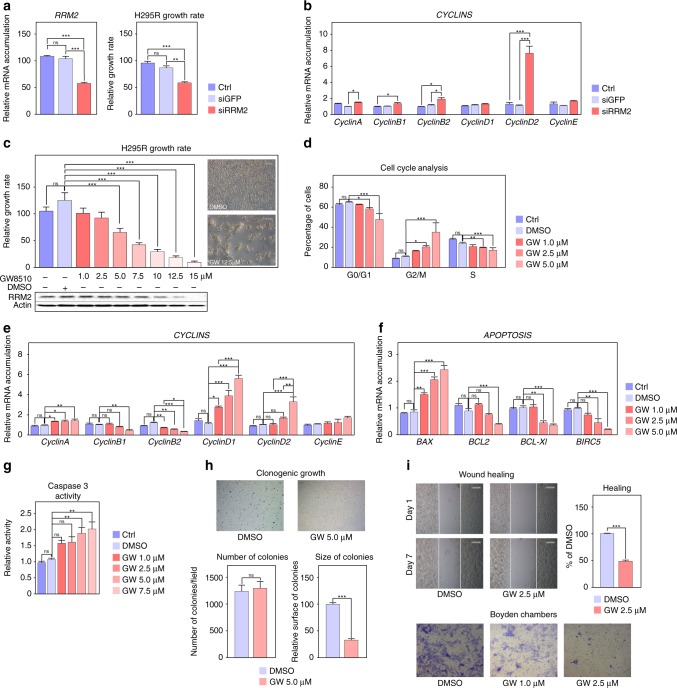
Inhibition of RRM2 reduces H295R cells growth and aggressiveness. **a** Effect of siRNA-mediated knockdown of RRM2 on RRM2 expression (left panel) and H295R cells growth after five days. **b** Effect of siRNA-mediated knockdown of RRM2 on expression of Cyclin coding genes in H295R cells was evaluated by RTqPCR. **c** Effect of pharmacological inhibition of RRM2 on the growth of H295R cells was determined by counting live cells after 5 days of treatment with increasing amounts of GW8510. Bottom panel shows RRM2 accumulation following GW8510 treatment. Representative images were taken at the end of treatment. **d** Effect of pharmacological inhibition of RRM2 on H295R cell cycle was determined by FACS following propidium iodide incorporation after 2 days of treatment with increasing amounts of GW8510. **e** Effect of RRM2 inhibition on expression of Cyclin coding genes in H295R cells was evaluated by RTqPCR. **f** Effect of RRM2 inhibition on expression of apoptosis-related genes in H295R cells was evaluated by RTqPCR. **g** Caspase 3 activity in H295R cells was determined after 1 day of GW8510 treatment at increasing concentrations. **h** Effect of RRM2 inhibition on clonogenic cell growth was determined by growing H295R cells in soft agar for 21 days in the presence or absence of 5 µM GW8510. Number and sizes of colonies were determined using Image J. **i** Effect of RRM2 inhibition on cell migration was determined by wound healing assays (top panel) in the absence or presence of 2.5 µM GW8510 for 7 days. For Boyden chambers migration assays (bottom panel) cells were incubated for 48h with two doses of GW8510 and migrating cells were stained with Haematoxylin. Graphs in **a**–**g** represent the mean of four experiments ± SEM. Statistical significance in these panels was evaluated by ANOVA. *p < 0.05, **p < 0.01, ***p < 0.001. Graphs in **h**, **i** represent the mean of three experiments ± SEM. Statistical significance was determined by Wilcoxon’s test

Altogether, our data demonstrate that EZH2 cooperates with E2F1 to induce expression of RRM2, PTTG1 and PRC1, three factors associated with ACC aggressiveness. Whereas drugs targeting PTTG1 and PRC1 are scarce, recent data have shown that RRM2 can be inhibited by compounds such as GW8510.^33^ Interestingly, transfection of H295R cells with siRNAs targeting RRM2 resulted in cell growth inhibition (Fig. 4a). This was associated with increased expression of genes coding *Cyclins A*, *B2* and *D2* (Fig. 4b), suggesting that RRM2 inhibition resulted in cell cycle arrest. To further gain insight into the role of RRM2 in ACC cell growth and to evaluate its potential as a therapeutic target, we treated H295R cells with GW8510 (GW). Incubation with increasing concentrations of GW for 5 days resulted in a dose-dependent decrease in RRM2 accumulation (Fig. 4c), which was associated with a dose-dependent reduction in cell growth and an almost complete absence of cells at the highest doses at the end of treatment (Fig. 4c). The growth inhibitory effect of GW at 5 µM was already evident at day 2 of treatment and was maintained up to 5 days (Supplementary Fig. 2E). Cell cycle analysis by FACS following propidium iodide incorporation showed a dose-dependent decrease in G0/G1 and S phases and a concomitant increase in G2/M, suggesting that RRM2 inhibition resulted in G2/M arrest (Fig. 4d). Consistent with this, GW inhibited expression of Cyclins B1/B2 and increased expression of Cyclins D1/D2 (Fig. 4e). Interestingly, RRM2 inhibition was also associated with decreased expression of the anti-apoptotic *BCL2*, *BCL-XL* and *BIRC5* genes and increased expression of the pro-apoptotic factor *BAX* (Fig. 4f). Consistent with potential induction of apoptosis, this was associated with increased caspase 3 activity (Fig. 4g).

Altogether, these data established RRM2 as a potential therapeutic target in the context of ACC. To further confirm this idea, we evaluated the effect of GW on the aggressive characteristics of H295R cells in culture. Consistent with its effect on cell growth, GW treatment induced a marked reduction in the size of H295R colonies growing in soft agar, even though it did not prevent clonogenic growth per se (Fig. 4h). However, even low doses of GW (1.0 and 2.5 µM) were associated with a significant reduction in H295R migratory capacities as shown by wound healing and Boyden chambers migration assays (Fig. 4i).

**Fig. 5:**
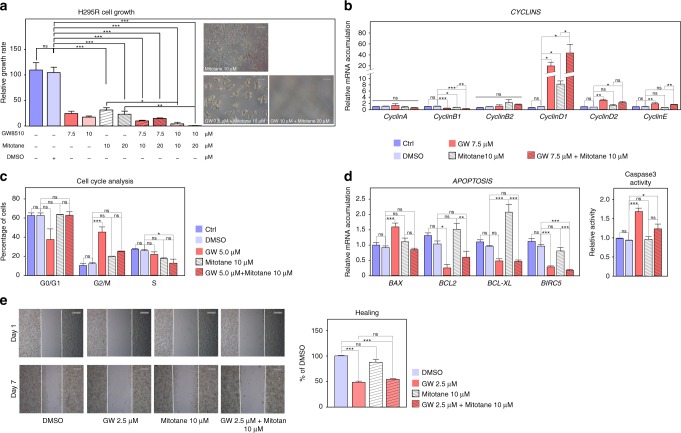
Combined effects of mitotane and RRM2 inhibition on H295R cells growth and aggressiveness. **a** Effect of pharmacological inhibition of RRM2 and/or mitotane treatment on the growth of H295R cells was determined by counting live cells after 5 days of treatment with two doses of GW8510 and/or mitotane. Representative images were taken at the end of treatment. **b** Effect of pharmacological inhibition of RRM2 and/or mitotane treatment on expression of Cyclin coding genes in H295R cells was evaluated by RTqPCR. **c** Effect of pharmacological inhibition of RRM2 and/or mitotane treatment on H295R cell cycle was determined by FACS, following propidium iodide incorporation after 2 days of treatment with GW8510 and/or mitotane. **d** Effect of RRM2 inhibition and/or mitotane treatment on expression of apoptosis-related genes in H295R cells was evaluated by RTqPCR (left panel). Caspase 3 activity was determined after 1 day of treatment with  5 µM  GW8510 and/or 10 µM mitotane (right panel). **e** Effect of GW8510 and/or mitotane on cell migration was determined by wound healing assays in the absence or presence of 2.5 µM GW8510 and/or 10 µM Mitotane for 7 days. Graphs in **a-e** represent the mean of four experiments ± SEM. Statistical significance was determined by ANOVA. *p < 0.05, **p < 0.01, ***p < 0.001

A vast majority of ACC patients are treated with Mitotane, which induces adrenostatic effects. However, these effects are only achieved when plasma mitotane concentrations exceed 14 mg/l (equivalent to 100 µM in tissue culture), which in practice is difficult to reach in a significant number of patients over a short time course. We thus evaluated the potential of RRM2 inhibition to synergise with low doses of Mitotane. Interestingly, combination of 10 µM mitotane with 10 µM GW had a significant effect on H295R cell growth compared with mitotane alone (Fig. 5a). This was further amplified by the combination of 20 µM mitotane and 10 µM GW which resulted in the death of almost all cells after 5 days of incubation (Fig. 5a). These growth inhibitory effects were reflected in a significant effect of the combination of both compounds on expression of CylinB1 and CyclinD1 (Fig. 5b). However, GW alone was more efficient than the combination of both compounds to induce G2/M arrest and only mildly enhanced the effect of mitotane on S phase arrest (Fig. 5c). We then investigated apoptosis. As expected, GW induced expression of the pro-apoptotic gene BAX and inhibited expression of the anti-apoptotic factors *BCL2*, *BCL-XL* and *BIRC5* (Fig. 5d). Mitotane alone had no effect on *BAX*, *BCL2* or *BIRC5* expression and only induced *BCL-XL* accumulation (Fig. 5d). Accordingly, there was no effect of mitotane alone on Caspase3 activity (Fig. 5d, right panel). Surprisingly, mitotane reversed the effect of GW on *BAX* and *BCL2* expression and abrogated induction of Caspase3 activity by GW (Fig. 5d). This showed that mitotane did not cooperate with GW8510 but rather prevented its pro-apoptotic effects. Investigation of the combination of mitotane and GW on cell migration further showed no effect of mitotane alone (Fig. 5e) or in combination with GW (Fig. 5e). Altogether, these data show a synergistic effect of RRM2 inhibition and mitotane treatment on H295R cell growth but not on other aggressiveness parameters.

## Discussion

We have previously shown that the methyltransferase EZH2 was the most deregulated epigenetic regulator in ACC and that it was associated with tumour aggressiveness.^8^ Although EZH2 is the catalytic component of PRC2 complex that deposits the H3K27me3 repressive histone mark, recent data suggest that EZH2 can also stimulate transcription by interactions with a number of transcription factors. By a combination of bioinformatics analyses relying on datasets from three distinct ACC patients’ cohorts, we show that the main mode of action of EZH2 in ACC is related with transcription activation rather than repression, although some PRC2 gene sets show significant enrichment, in particular in the cohort from Michigan (Fig. 1b). This is in sharp contrast with normal mouse adrenal, in which *Ezh2* ablation is associated with up-regulation of PRC2 target genes (Fig. 2a, b and ref. ^15^). Interestingly, these differential activities are also associated with differential effects on cell homeostasis. Indeed, whereas knockdown of EZH2 in H295R cells results in decreased proliferation and induction of apoptosis,^8^ inactivation of *Ezh2* in the adrenal cortex does not alter proliferation but rather modulates differentiation of steroidogenic cells.^15^ This suggests that ACC cells have rerouted EZH2 activity from PRC2-mediated control of differentiation to PRC2-independent stimulation of proliferation. How this is achieved is unclear. In breast cancer and castration resistant prostate cancer, phosphorylation of serine 21 by AKT has been shown to play an essential role in diverting EZH2 away from its function as a histone methyltransferase, although its enzymatic activity was still required. This phenomenon was associated with cancer aggressiveness.^12,34^ Interestingly, the growth factor IGF2, which signals through PI3K/AKT pathway is almost systematically upregulated in ACC.^2,3^ Although the role of IGF2 in ACC pathogenesis is still unclear,^5,6^ it may be involved in modulation of EZH2 activity through activation of AKT.

Using a combination of bioinformatics and in vitro analyses, we show that EZH2 functionally interacts with E2F1 to stimulate gene expression in ACC. These findings are consistent with recent data showing a similar functional interaction in castration resistant prostate cancer (CRPC). In this study, using a combination of ChIP sequencing and expression data, Xu et al. showed that EZH2 cooperated with E2F1 to stimulate expression of a group of 56 genes involved in cancer aggressiveness. Interestingly, collaboration between EZH2 and E2F1 was also found to regulate the majority of these genes in diffuse large B-cell lymphomas, suggesting that this activity was generally found in cancer.^35^ In contrast, even though we showed functional interaction between EZH2 and E2F1 in ACC, only six out of 56 CRPC genes were also found in our list of EZH2/E2F1 targets (Supplementary Table 7). This suggests that the repertoire of EZH2/E2F1 positive targets may vary from one tissue to the other.

In contrast with CRPC, where EZH2 expression seems to be independent of E2F1 levels,^35^ we have previously shown that E2F1 directly stimulates EZH2 expression in ACC.^8^ A reciprocal effect of EZH2 on E2F1 expression has been shown in hepatocellular carcinoma cells, where EZH2 repressed expression of E2F1, which may constitute a negative feedback loop.^36^ In contrast, we show that tampering with EZH2 expression/activity has no impact on E2F1 expression (Fig. 3d and Fig. S2D). Therefore, our data show that ACC progression may be dependent on a feed-forward mechanism involving E2F1 stimulated EZH2 expression, which would in turn stimulate E2F1 transcriptional activity.

Consistent with observations in CRPC,^35^ the 94 positive targets of EZH2/E2F1 interaction are mostly involved in cell cycle control, DNA replication and mitosis and their overexpression is strongly associated with reduced overall and disease-free survival. By combining patients’ data with transcriptome analysis of our mouse model of adrenal-specific *Ezh2* deletion,^15^ we identified *PTTG1*, *PRC1* and *RRM2* as three robust positive target genes. Interestingly, recent reports have also shown overexpression of *PTTG1*^22^ and *RRM2*^37^ in ACC. However, they did not provide a molecular mechanism accounting for their deregulation in these tumours. Here, we show that these three genes are transcriptionally stimulated by cooperation of EZH2 and E2F1 on their regulatory regions. Increased expression of these genes is strongly associated with poor prognosis and results in decreased overall and disease-free survival, suggesting that they may play a role in ACC aggressiveness.

Consistent with this idea, we show that pharmacological inhibition and/or siRNA-mediated knockdown of RRM2, which is required for the catalytic activity of the ribonucleotide reductase, is associated with decreased cell growth, G2/M arrest, increased apoptosis and inhibition of cell migration. These findings are consistent with the anti-tumour effects of RRM2 inhibition in pancreatic,^38^ cervical^29^ and breast cancer^39^ as well as in neuroblastoma^40^ and glioblastoma.^41^ It further suggests that RRM2 may be an interesting therapeutic target in the context of ACC. Interestingly, a number of compounds that can inhibit ribonucleotide reductase activity, such as Triapine (https://clinicaltrials.gov/ct2/results?term=triapine) or COH-29 (NCT02112565) have undergone or are undergoing phase I and II clinical trials^42,43^ and may prove interesting in the context of ACC.

Altogether, our data show that EZH2 cooperates with E2F1 to stimulate expression of genes involved in ACC aggressiveness and establishes RRM2 as an interesting therapeutic target in this cancer.

## Supplementary information

Supplementary information description

## Data Availability

The authors declare that all data supporting the findings of this study are available within the article and its supplementary information files or from the corresponding author upon reasonable request. Gene expression data from patients cohorts are readily available from GEO (GSE49820 and GSE10927) and Broad GDAC (TCGA ACC data). Mouse gene expression data are available from GEO (GSE109578). ChIP sequencing data were downloaded from GEO and Encode. Details can be found in Supplementary Table 1.
