# Supplementary material for: EZH2 cooperates with E2F1 to stimulate expression of genes involved in adrenocortical carcinoma aggressiveness
Source: Br J Cancer. 2019 Jul 31;121(5):384–94. doi: 10.1038/s41416-019-0538-y (PMC6738105; doi:10.1038/s41416-019-0538-y)
Supplement: Supplementary file 1 — Supplementary information description [file 41416_2019_538_MOESM1_ESM.pdf]

Figure S1. Expression of RRM2, PTTG1 and PRC1 is associated with poor prognosis in ACC. A- Correlogram shows correlation of expression of *RRM2*, *PTTG1* and *PRC1* with expression of *EZH2* and *E2F1* in TCGA and Michigan cohorts. **B-** Expression of *RRM2, PTTG1* and *PRC1* in normal adrenals, adrenocortical adenomas and adrenocortical carcinomas. Significance was evaluated by ANOVA. **C-** Expression of *RRM2, PTTG1* and *PRC1* in the groups of good (blue) and poor (red) prognosis in TCGA and Michigan cohorts. Significance was evaluated by Wilcoxon's test. **D-** Overall (O.S) survival as a function of *RRM2*, *PTTG1* and *PRC1* expression in TCGA and Michigan cohorts. **E-** Overall (O.S) survival as a function of *RRM2*, *PTTG1* and *PRC1* expression in all cohorts combined. Statistical significance was evaluated by the Logrank test.

Figure S2. Effect of E2F1 inhibition by HLM. A-Effect of increasing doses of HLM treatment for 72h on expression of RRM2, PTTG1 and PRC1 was evaluated by RTqPCR (graphs) and western-blot (bottom panels) in H295R cells. **B-** Effect of pharmacological inhibition of E2F1 on the growth of H295R cells was determined by counting live cells after 3 days of treatment with increasing amounts of HLM. **C-** Effect of E2F1 inhibition on expression of Cyclin coding genes in H295R cells was evaluated by RTqPCR. **D-** Effect of 5 μM DZNep and/or 40 μM HLM treatment for 48 h on expression of EZH2 and E2F1 was evaluated by RTqPCR (graphs) and western-blot (bottom panels) in H295R cells. **E-** Kinetic effect of pharmacological inhibition of RRM2 by 5 μM GW8510 on growth of H295R cells at 2, 3, 4 and 5 days. Graphs in A-E represent the mean of 4 experiments ± SEM. Statistical significance was determined by ANOVA in A-D and by multiple t tests in E. * $p<0.05$, ** $p<0.01$, *** $p<0.001$.

Table S1. Online sources for ChIP data analysed in the manuscript

Table S2. Antibodies used in the manuscript

Table S3. Primers used in the manuscript

Table S4. Correlation coefficients of EZH2 with all other genes in ACC patients' transcriptome data.

Table S5. EZH2/E2F1 metagene in ACC

Table S6. Association of expression of EZH2_E2F1 metagene and different prognostic factors with overall survival in TCGA, Cochin and Michigan cohorts. Univariate and multivariate hazard ratios (HR) and 95% confidence intervals (95% CI) were determined by Cox proportional hazards regression using clinical an molecular data from the three cohorts of patients.

Table S7. Intersection of EZH2/E2F1 metagenes from ACC and prostate cancer patients

Figure S1

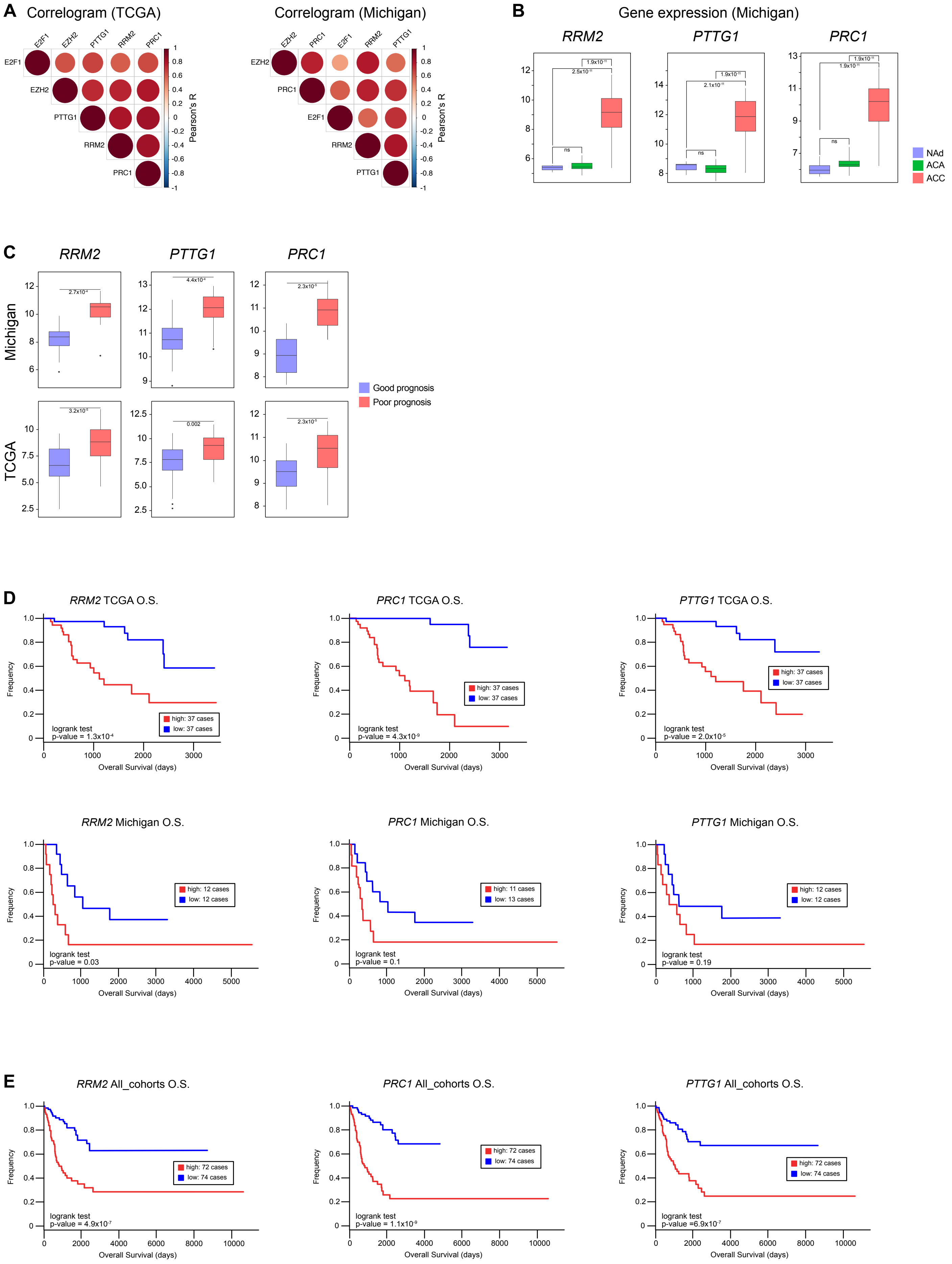

A
Correlogram (TCGA)
Correlogram (Michigan)
Pearson's R
B
Gene expression (Michigan)
RRM2
PTTG1
PRC1
NAd
ACA
ACC
C
Michigan
TCGA
Good prognosis
Poor prognosis
D
RRM2 TCGA O.S.
PRC1 TCGA O.S.
PTTG1 TCGA O.S.
logrank test
p-value = 1.3x10-4
p-value = 4.3x10-9
p-value = 2.0x10-5
high: 37 cases
low: 37 cases
RRM2 Michigan O.S.
PRC1 Michigan O.S.
PTTG1 Michigan O.S.
p-value = 0.03
p-value = 0.1
p-value = 0.19
high: 12 cases
low: 12 cases
high: 11 cases
low: 13 cases
E
RRM2 All_cohorts O.S.
PRC1 All_cohorts O.S.
PTTG1 All_cohorts O.S.
p-value = 4.9x10-7
p-value = 1.1x10-9
p-value =6.9x10-7
high: 72 cases
low: 74 cases
Frequency
Overall Survival (days)

Figure S2

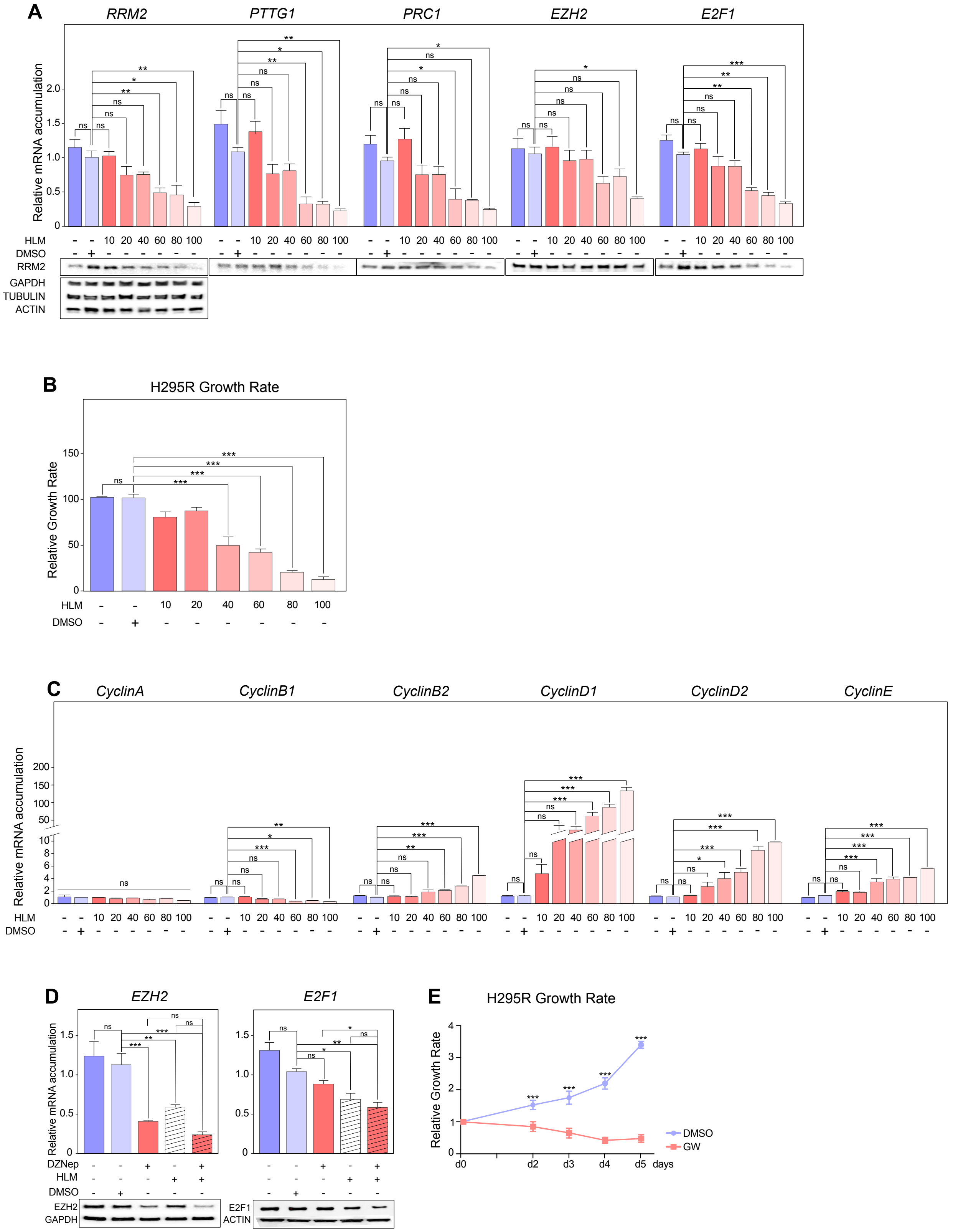

A
RRM2
PTTG1
PRC1
EZH2
E2F1
Relative mRNA accumulation
HLM
DMSO
RRM2
GAPDH
TUBULIN
ACTIN
B
H295R Growth Rate
Relative Growth Rate
HLM
DMSO
C
CyclinA
CyclinB1
CyclinB2
CyclinD1
CyclinD2
CyclinE
Relative mRNA accumulation
HLM
DMSO
D
EZH2
E2F1
Relative mRNA accumulation
DZNep
HLM
DMSO
EZH2
GAPDH
E2F1
ACTIN
E
H295R Growth Rate
Relative Growth Rate
DMSO
GW
d0
d2
d3
d4
d5
days

Experiment	Datasets GEO	Datasets ENCODE	Initial Data
Hela			
E2F1	GSM935484		SRR502355.sra<br>SRR502356.sra
H3K27me3	GSM733696		SRR227472.sra<br>SRR227473.sra
H3K4me3	GSM733682		SRR227441.sra<br>SRR227442.sra
EZH2	GSM1003520		SRR568331.sra<br>SRR568332.sra
Input	GSM509051		SRR036650.sra<br>SRR036651.sra<br>SRR036652.sra<br>SRR036653.sra<br>SRR036654.sra
K562			
E2F1		Replicat 1 :<br>ENCLB809EWT	ENCFF749JUG.fastq<br>ENCFF942QFN.fastq
Input	GSM777645		SRR332096.sra
LM2			
E2F1	GSM2501567		SRR5282135.sra
Input	GSM2501570		SRR5282138.sra
LnCap_ABL			
E2F1	GSM1656410		SRR1970880.sra
Input	GSM1656411		SRR1970881.sra
MCF7			
E2F1	GSM935477		SRR1970880.sra
Input	GSM594608		SRR065825.sra
Raji			
E2F1	GSM1976292		SRR3048087.sra
Input	GSM1976298		SRR3048093.sra

Supplementary Table 1.

Antibody	Supplier	Reference	Dilution
EZH2	Cell Signaling Technology	3147	1/1000
E2F1	Millipore	05-379	1/1000
RRM2	Abcam	ab57653	1/400
PTTG1	Abcam	ab79546	1/5000
PRC1	Abcam	ab51248	1/5000
ACTIN	Sigma Aldrich	A2066	1/500
TUBULIN	Sigma Aldrich	T6074	1/500
GAPDH	Novus Biologicals	NB30021	1/1000

Supplementary Table 2.

RT-qPCR Primers

Gene	Orientation	Sequence 5'-3'
EZH2	Forward	ACCAGTTTGTTGGCGGAAGCG
	Reverse	CCAAGTCACTGGTCACCGAACAAG
BCL-XL	Forward	GATCCCCATGGCAGCAGTAAAGCAAG
	Reverse	CCCCATCCCGGAAGAGTTCATTCACT
Birc5	Forward	ATTCGTCCGGTTGCGCTTTCC
	Reverse	CACGGCGCACTTTCTTCGCAG
BCL-2	Forward	TTGTGGCCTTCTTTGAGTTCGGTG
	Reverse	GGTGCCGGTTCAGGTACTCAGTCA
Actin	Forward	CGCGAGAAGATGACCCAGATC
	Reverse	TCACCGGAGTCCATCACGA
RRM2	Forward	GATATTCTGGCTCAAGAAACGAGGACTG
	Reverse	CTCTCCTCCGATGGTTTGTGTACCAG
PTTG1	Forward	GCTCTGTTCCTGCCTCAGATGATG
	Reverse	GTCAAGGATCATGAGAGGCACTCC
PRC1	Forward	GCAGAAATTCATGGAGTATGTGGCAGAAC
	Reverse	CGCCGCTTGCTAGGTGTTCGAG
CyclinA	Forward	TCCTGTCTTCCATGTCAGTGC
	Reverse	CAAACTCTGCTACTTCTGGGG
CyclinB1	Forward	CAGTCAGACCAAAATACCTACTGGGT
	Reverse	ACACCAACCAGCTGCAGCATCTTCTT
CyclinB2	Forward	CATGTGCGTTGGCATTATGG
	Reverse	CAAGAGCAGAGCAGTAATCC
CyclinD1	Forward	CGGTGTCCTACTTCAAATGTGTGCAGAA
	Reverse	GGGCTCCAGCGACAGGAAGC
CyclinD2	Forward	CATTGCTCTGTGTGCCACCG
	Reverse	GCGAGCTCACTTCCTCATCC
CyclinE	Forward	CACTTTCTTGAGCAACACCC
	Reverse	CCGGTCAAAGAAATCTTGTGCC

ChIP qPCR Primers

Gene	Orientation	Sequence 5'-3'
PRC1_ChIP1	Forward	GCGAGTCCCCACCCCGATTGCG
	Reverse	GGGCGAAGCCTCGTCGCG
PRC1_ChIP2	Forward	GCTCCGATGAGGTAGCTGGCTTG
	Reverse	GGGGGATGACGTCACTGTCTGTG
PTTG1_ChIP	Forward	GACCCTCCCATTCACTCACGCAG
	Reverse	GGGCCGCGAGTTGTGGTTTAAACC
RRM2_ChIP 1	Forward	GCAGACCAATGGTGGAGTAGATGC
	Reverse	GGCTGGTCTCAAGCGATCCTCC
RRM2_chIP 2	Forward	GCGCTTGAAAATCGCGCGCGG
	Reverse	CGGCCCTTCCCATTGGCTGTG

Supplementary Table 3.

Supplementary Table 4.

	TCGA						Cochin						Michigan				
Gene	P.estimate.cor	P.p.value	S.estimate.rho	S.p.value	FDR	Gene	estimate.cor.x	p.value.x	estimate.rho.x	p.value.1.x	FDR	Gene	estimate.cor.y	p.value.y	estimate.rho.y	p.value.1.y	FDR
A1BG	-0.0418399378651341	0.714279720245056	-0.116236611489776	0.306989804	0.858415947831621	A1BG	0.164691622760676	0.0388545301922259	0.13299642811567	0.0957385541998008	0.089829575970573	A1BG	0.256839449543463	0.0388934227557902	0.183967830937561	0.142384148523296	0.0687192303192021
A1CF	-0.116948140149721	0.304695128080636	0.0614677112444582	0.590481733	0.577227992641649	A1CF	0.113303260464271	0.156348473868475	0.0314364231998308	0.694977654789292	0.275653649101573	A1CF	-0.352875128786344	0.00393731730761085	-0.390078671328671	0.00142468339707973	0.0096362959784706
A2M	-0.135020464614881	0.235463781756445	-0.174488802336904	0.123912154	0.507537629347679	A2M	-0.221524987023923	0.00515385903239687	-0.375292933764561	1.18165162656577e-06	0.0161427896840765	A2M	-0.521128201130778	8.55702196137156e-06	-0.458216783216783	0.000146600916983976	4.40793213134709e-05
A2ML1	-0.127334816720681	0.263440458082502	-0.266589638493282	0.017554460	0.536942688629362	A2ML1	-0.0153134667701906	0.848549039069132	0.0691981620379339	0.387622481875011	0.907768482146709	A2ML1	-0.242364267903316	0.0517511586240035	-0.325262237762238	0.00845835418216238	0.0875458343535136
A4GALT	0.223301615832082	0.0479097891162512	-0.0578383641674781	0.611952334	0.195788155402787	A4GALT	-0.0327250440057378	0.683133784701793	0.069230108076511	0.387402879186737	0.792819326505706	A4GALT	-0.527728871702489	6.26225132542893e-06	-0.507255244755245	2.11963528685308e-05	3.3007223645183e-05
A4GNT	0.00246200548280835	0.982819668968575	-0.0249904868051836	0.826950746	0.992371824561201	A4GNT	-0.108481064833474	0.1748555589231	0.0107142558334514	0.893710528980453	0.299924610060027	A4GNT	-0.420305460212208	0.000490658233251525	-0.387019230769231	0.00156192619629113	0.00158887649358296
AAAS	-0.136331972750949	0.230903837179869	-0.0555744888023369	0.625971439	0.501903076353824	AAAS	0.199894071067269	0.0117974301907582	0.174521208746876	0.0282974309085546	0.0330132577970514	AAAS	0.0723206682960752	0.566979922829365	0.042417421521561	0.737251176183751	0.636265869402466
AACS	0.245819560544221	0.0289848775811632	0.404259980525803	0.000247551	0.140025136492488	AACS	0.495875859272249	3.47557538304955e-11	0.441183920189796	6.55286890121 07e-09	6.82146511747785e-10	AACS	0.612099019145537	6.03586476355389e-08	0.641739510489511	1.96012244632133e-08	4.30920554635968e-07
AADAC	-0.127014238567628	0.264654282980241	-0.145611687019178	0.200392971	0.537834526736468	AADAC	-0.49708050556017	3.06370789160846e-11	-0.399009064308252	2.06975804389577e-07	6.08445116337775e-10	AADAC	-0.542793814428645	2.99480402617274e-06	-0.536975524475524	5.72388936765063e-06	1.67819165850861e-05
AADACL2	-0.115383771617781	0.311254249307759	-0.115187772864035	0.312082425	0.583395237188103	AADACL2	-0.0220957016419758	0.782880680708128	-0.0381756612856902	0.633914784597379	0.865375762944259	AADACL2	-0.102273513277174	0.417546415037338	-0.187901569304009	0.133898739652405	0.497780149908105
AADAT	-0.0380632861227631	0.739102464881434	-0.115092502434275	0.311800323	0.87224587090071	AADAT	-0.0615353736278546	0.442443124899686	-0.127133160317513	0.11142602373632	0.596075955316738	AADAT	0.0686806395871827	0.586703252668463	0.00528846153846154	0.966692244686866	0.654656356321141
AAGAB	0.0880011379853397	0.440587253852054	0.21292599805258	0.059705615	0.691383308946756	AAGAB	0.337407365527365	1.45567915490474e-05	0.301897670753565	0.000115726910442074	8.94494433971838e-05	AAGAB	0.547885804028478	2.31492197588778e-06	0.544405594405594	4.05600986872313e-06	1.32411936185629e-05
AAK1	-0.199343056748456	0.0781906741242192	-0.0363193768257059	0.750189993	0.266171282758831	AAK1	-0.328615359510332	2.49215502999618e-05	-0.194946897317122	0.0141047404990341	0.000144825044514614	AAK1	-0.521920715653213	8.24502953807491e-06	-0.487762237762238	4.72282420552254e-05	4.25953343970143e-05
AAMP	0.252247295643905	0.024917054179705	0.203042843232717	0.072795552	0.126415206509968	AAMP	-0.236371320627852	0.00278942468218275	-0.1653026204899	0.0379286967492418	0.00941317479455876	AAMP	0.0530173802464572	0.674893661298569	-0.00961580489576226	0.939401127280084	0.732885155683587
AANAT	0.0439123976448501	0.700781105112023	0.0510148364197455	0.655240886	0.85115399255485	AANAT	0.0785952450075828	0.326296037277736	0.166034337453868	0.0370745946590918	0.479686181129372	AANAT	-0.391059964754982	0.0012776288144142	-0.369518897737287	0.00245078349117785	0.00365307201751023
AARS	-0.0418465148497714	0.714236740608084	0.109104186952288	0.337772785	0.858415947831621	AARS	0.317149551725961	4.90359610285651e-05	0.203619486170847	0.0102856423714153	0.000262656980662171	AARS	0.28534601025662	0.0212213479212457	0.369934112064392	0.00242119452765343	0.0409723919558728
AARS2	0.29016343700252	0.00948702588628692	0.314143135345667	0.004981529	0.0661849728320927	AARS2	0.381101109731861	7.81014446449291e-07	0.287200971768152	0.000253414394117105	6.39611919890189e-06	AARS2	0.606531141508613	8.55169923763555e-08	0.59173951048951	3.82076143617712e-07	6.00537763322133e-07
AASDH	-0.138826613106819	0.222400901609391	-0.155306718597858	0.171393951	0.491731072543182	AASDH	0.0189484671341117	0.813193262937855	0.189446093722126	0.0171245560658536	0.884665254854328	AASDH	0.150263321965513	0.232183559306334	0.253146853146853	0.0421588265375724	0.309346558910125
AASDHPPT	-0.108337635096009	0.341919843085338	-0.130428432327167	0.251355591	0.612606638696734	AASDHPPT	0.0222626351008415	0.781281506904896	-0.0550840979465702	0.491817667132455	0.864490476146119	AASDHPPT	0.144493388642821	0.250812628101189	0.042701048951049	0.734984110048397	0.32937290013742
AASS	-0.103541744497962	0.363839008938541	-0.0944498539435248	0.406911693	0.630420099894015	AASS	-0.145417468937977	0.0682945612243309	-0.190815209661146	0.0163247006371656	0.142891564057272	AASS	-0.0762618152061092	0.545981412609321	-0.0795454545454545	0.52786904500226	0.61745834835841
AATF	0.168965020489834	0.136597450064934	0.0143865628042843	0.899710497	0.377192902283393	AATF	0.0297759938819859	0.710347881750604	-0.166090749706258	0.0370094258272395	0.812570181622258	AATF	-0.148858140700036	0.23662950876949	-0.286650859401502	0.02061165107266	0.314067706601953
AATK	-0.0185561944441148	0.871056906780652	0.230915287244401	0.040827900	0.940110975690369	AATK	-0.0542661961282805	0.498280013204557	0.048102338414558	0.548383897658364	0.646419212394891	AATK	-0.538907514309619	3.63493227985183e-06	-0.541477272727273	4.64962506367565e-06	2.00440551431829e-05
ABAT	-0.266437911424516	0.0176212807853749	-0.31572541382668	0.004758300	0.101494491534	ABAT	-0.169910253962431	0.0328162273490562	-0.0529787018803439	0.508542588492882	0.0783182195354064	ABAT	0.116434554416204	0.355664475205233	0.143793706293706	0.252466973868468	0.43779905637806
ABCA1	-0.144616457579973	0.203519654864226	-0.260589094449854	0.020621369	0.467287175981502	ABCA1	-0.552128036758887	5.48192974765277e-14	-0.512148241789065	6.06294160832505e-12	1.77993521436133e-12	ABCA1	-0.58181727916868	3.71530699653589e-07	-0.578277972027972	7.71130746620197e-07	2.38885369349726e-06
ABCA12	0.0135734401255424	0.905492550436755	-0.0563463935619745	0.621852056	0.955324225466544	ABCA12	0.0479043502900796	0.550033193177918	0.113060330992271	0.15724412787394	0.690024605547221	ABCA12	-0.00628990017814998	0.960339689127023	-0.118947978022595	0.345303615803436	0.969338295932964
ABCA13	-2.21758999784088e-05	0.999845240731557	0.211676860533268	0.061107341	0.999845240731557	ABCA13	0.245454473186258	0.00188031663648625	0.0888482885620695	0.266940894081858	0.00666473416975585	ABCA13	0.0970823929356086	0.441693775320094	0.056993006993007	0.651284719540061	0.520671239192415
ABCA17P	-0.07737417752628	0.497916148800955	-0.148222979552093	0.191957402	0.733421377615822	ABCA17P	0.182371667762046	0.0218225536607184	0.299001229922572	0.000135510328471929	0.0557603348915789	ABCA17P	0.327821696640163	0.0076804616046644	0.260708041958042	0.0362355507367026	0.0171643171901395
ABCA2	0.080267340519875	0.4819336015472	0.0249513145082765	0.826906902	0.721702805588884	ABCA2	-0.0193765543120139	0.80905272863856	-0.0463902117337934	0.562725801575277	0.881497350667923	ABCA2	-0.424495847923036	0.000424709986004515	-0.418181818181818	0.000588480660579707	0.0014028495291122
ABCA3	-0.17173172899595	0.130197727621258	-0.200292112950341	0.076823927	0.366398784418503	ABCA3	-0.0983328782596466	0.218999825391856	0.0434907284229355	0.587414837155222	0.355223505327231	ABCA3	-0.011727724325332	0.926125628489325	0.0186188811188811	0.882770116579	0.942153630669102
ABCA4	0.109915171697562	0.334894834016111	-0.0463643917265888	0.684929559	0.605797477791944	ABCA4	0.0444231875701077	0.579420960735399	0.124191080050749	0.120020547114337	0.714333170285603	ABCA4	-0.43536650398438	0.000289498205597386	-0.44763986013986	0.00021497857602575	0.00100536488853433
ABCA5	-0.270852612548495	0.015764029735696	-0.330769230769231	0.003041576	0.0938533955787464	ABCA5	-0.345928208855517	8.50913542214555e-06	-0.336102750630081	1.57821767671853e-05	5.4946749604244e-05	ABCA5	-0.198289337269067	0.113328590552143	-0.163243006993007	0.193369610686356	0.170114081093242
ABCA6	0.050490058590379	0.658565542817255	-0.124732229795521	0.272790669	0.827270911334895	ABCA6	-0.189715297637091	0.0169646472151769	-0.268447125883443	0.000648789184023013	0.0450546963692162	ABCA6	-0.664032921660699	1.64134162499476e-09	-0.675218531468532	0	1.34799546222974e-08
ABCA7	-0.0572016434093991	0.61656409497656	-0.00963972736124635	0.932732429	0.805710452672533	ABCA7	0.0365993009472201	0.647997277818499	0.0849935194936636	0.288330342942002	0.76811730633176	ABCA7	0.381104676184028	0.00173583853021775	0.37771391735119	0.00192269915272362	0.00473243323835704
ABCA8	-0.14796671376444	0.193131516890101	-0.285783836416748	0.010904078	0.453760411149016	ABCA8	-0.463185063255009	8.89289384197694e-10	-0.433722238322137	1.24994106089003e-08	1.32458070898702e-08	ABCA8	-0.669422657699969	1.08368958380479e-09	-0.568793706293706	1.24607539160313e-06	9.02490138832037e-09
ABCA9	0.242308280898483	0.0314344858900708	-0.0885345666991237	0.437020686	0.147745071752334	ABCA9	-0.0969608561744539	0.225527916881261	-0.18888380962282	0.0174627857076279	0.362870909787101	ABCA9	-0.638500003346128	1.05106190921868e-08	-0.610270979020979	1.38735985513728e-07	8.0899281547041e-08
ABCB1	-0.0786032041148648	0.491093114862821	0.02682570593963	0.814123732	0.728262699689351	ABCB1	-0.42593607971476	2.41218660904096e-08	-0.428839058139632	1.89146751970422e-08	2.69795598253002e-07	ABCB1	-0.544805817714595	2.70644538470458e-06	-0.616914335664336	9.48758615975 74e-08	1.52806618990146e-05
ABCB10	0.377745201096106	0.000599033643845726	0.347395326192795	0.001808986	0.00929230384939668	ABCB10	0.0343619469921408	0.668199802518225	-0.220108205796444	0.00545411262811774	0.782591663869236	ABCB10	0.301431959710332	0.0146825629913376	0.182604895104895	0.145158862936479	0.0299178968489355
ABCB11	-0.0514655420037817	0.652390840284685	0.0508849258215347	0.656063296	0.824490580655049	ABCB11	0.221733935221033	0.00511085202606099	0.233029795027976	0.00321333515053582	0.016026364469389	ABCB11	-0.396854806046497	0.00106416893465527	-0.370323426573427	0.00254354406919749	0.00311976614260963
ABCB4	0.0047160127239768	0.967097442301023	0.0870496592015579	0.444772625	0.984976219878966	ABCB4	0.195272514103526	0.0139415712072959	0.0804888048150519	0.314739279915046	0.0380920638062211	ABCB4	-0.00163612483415357	0.989679716320378	-0.0590034965034965	0.639821227178672	0.991692217798639
[illegible]	[illegible]	[illegible]	[illegible]	[illegible]	[illegible]	[illegible]	[illegible]	[illegible]	[illegible]	[illegible]	[illegible]	[illegible]	[illegible]	[illegible]	[illegible]	[illegible]	[illegible]

ALMS1P	0.102234799203965	0.369958415285416	0.150561874292769	0.185351287	0.636707060347629	ALMS1P	0.0697615104429377	0.38376095381939	0.167688309159926	0.0352035919421038	0.539274800382793	ALMS1P	-0.200734970237596
ALOX12	-0.0562466389131374	0.622470088487574	-0.121387706316272	0.286574157	0.808192776877584	ALOX12	-0.0518853301223102	0.517343210906417	-0.0233997838312089	0.770412637691545	0.662513113020808	ALOX12	-0.243967562511763
ALOX12B	0.0224276342287751	0.844462644226804	-0.0745138701888959	0.513984824	0.926110928838	ALOX12B	0.0488453463992472	0.542216095865927	0.0409609064156131	0.609353226016744	0.683712214033679	ALOX12B	-0.395189616535493
ALOX12P2	-0.0428204481616737	0.707882103870612	-0.0329134770201892	0.773380020	0.855566624929905	ALOX12P2	0.00961278121330509	0.904582869443479	0.0445601939886637	0.578250692319641	0.943295333358253	ALOX12P2	-0.203091081196717
ALOX15	-0.082330353671691	0.470706323650133	-0.177892300679282	0.116769664	0.713709726245962	ALOX15	-0.0580203321706157	0.468989820391613	-0.00276409295688881	0.972503724732115	0.620838027370074	ALOX15	-0.33440238230191
ALOX15B	0.313862085093164	0.0048511711832111	0.172956261908399	0.127439161	0.0418980190661787	ALOX15B	-0.21864563404198	0.00578038177079522	-0.17763214440816	0.0255581765782346	0.0178402059346756	ALOX15B	-0.455034493017429
ALOX5	-0.020426108692503	0.858192073351783	-0.310516066212269	0.005528766	0.933728838527427	ALOX5	-0.430545666985609	1.63774418773169e-08	-0.374699650190986	1.2321495201079e-06	1.90106371350192e-07	ALOX5	-0.583263116119933
ALOX5AP	0.034604423481624	0.76207346136927	-0.115603700097371	0.309644924	0.884784650336979	ALOX5AP	-0.518930152725634	2.84832647379763e-12	-0.439756997133351	7.42316185708041e-09	6.71417290430402e-11	ALOX5AP	-0.474702043541066
ALOXE3	-0.261972411461969	0.0196881446710246	-0.197126544017759	0.081630621	0.109441294351749	ALOXE3	0.0728083530952053	0.363282970211892	0.20751097575446	0.0088913837072039	0.518680603142154	ALOXE3	-0.479969043450322
ALPI	0.0850277622633307	0.456242659972054	0.176915854420687	0.118823799	0.702605880941157	ALPI	0.110303075684401	0.167681258403412	0.0760406992531693	0.342314023144216	0.290898225330458	ALPI	-0.322011132588197
ALPK1	-0.279011471025665	0.0127727585697422	-0.234664070107108	0.037599100	0.0820459951258075	ALPK1	0.088330717714462	0.269748235961452	0.147374794252143	0.0646216666632154	0.415188113372655	ALPK1	0.12507330696084
ALPK2	0.272984049998743	0.0149486063859845	0.0310844805896713	0.785662885	0.0904415836959319	ALPK2	0.0458084335390634	0.567639643620437	0.117386568658174	0.141861688303405	0.705354665348483	ALPK2	-0.10405849527472
ALPK3	-0.144591111662733	0.20359973545243	-0.177190847127556	0.118129588	0.467362531079174	ALPK3	-0.342694814611187	1.04514212244047e-05	-0.416071291388305	5.41938179715512e-08	6.62343080004442e-05	ALPK3	-0.32122293231227
ALPL	0.171979147633217	0.129636724587582	0.288120740019474	0.010249996	0.365287810071691	ALPL	0.289769417453078	0.00022164469049768	-0.0178121983666498	0.824208266501485	0.00101077006491511	ALPL	0.123607868614317
ALPP	0.291618365337776	0.00911827551032163	0.393886192886737	0.000328804	0.0644766725216808	ALPP	0.0369620515164838	0.644745323682314	0.0799548495988341	0.317970800071338	0.765741655957125	ALPP	-0.332425101465811
ALPPL2	-0.0899131606747497	0.430681758963337	-0.0774176035155564	0.497674226	0.684456599491758	ALPPL2	-0.0132036272750652	0.869214466300431	0.00306529846347249	0.969508806700257	0.920462371273565	ALPPL2	-0.512297229791664
ALS2	0.281546261436443	0.0119498565996752	0.294401168451801	0.008659536	0.0780787128603819	ALS2	0.0315724949145308	0.693723454758012	-0.012859041147262	0.87259842158132	0.800617402337951	ALS2	0.390537105782185
ALS2CL	-0.149269894105056	0.189195716800826	-0.149780915287244	0.187288301	0.449101402650832	ALS2CL	0.0447774082692268	0.576397571361002	0.0746670763876001	0.3511295632223	0.71227542547698	ALS2CL	-0.569452763443733
ALS2CR11	-0.0740743661891105	0.516477102258304	-0.00428458402259498	0.970105972	0.744790777608821	ALS2CR11	-0.0668982300230677	0.403629427183397	-0.176088564637155	0.0268892980618832	0.559549529702285	ALS2CR11	0.0902756753861581
ALS2CR12	0.0690752029029354	0.545251380444894	-0.0597930619159174	0.600661035	0.763089569943328	ALS2CR12	-0.0451582995864025	0.573154787076749	-0.0711529553508679	0.374323588959041	0.709467032261419	ALS2CR12	-0.280093818001323
ALX1	0.0305145063548063	0.78950149428303	0.166918213594887	0.141483060	0.898965115109815	ALX1	0.105155525871278	0.188530810593161	0.10089280985975	0.207151978183314	0.317175566391309	ALX1	0.155439084060899
ALX3	0.38483804950207	0.000461966239815759	0.413169425511198	0.000175633	0.00773646764697382	ALX3	-0.0132584463260855	0.868676341215407	0.0202385760585797	0.800731260054101	0.920318124309562	ALX3	-0.506936055387656
ALX4	0.27797681676664	0.0131225698065234	0.464385859622128	1.624847169	0.0836321276727372	ALX4	0.185576866980478	0.0195717345496007	0.0896497116870278	0.262633304670829	0.0509280046725299	ALX4	-0.302486793415477
AMBN	-0.0292978447957452	0.797712018867895	-0.117541886555212	0.302229377	0.902737888699948	AMBN	-0.0814003962177243	0.309271872767103	0.0441402978740039	0.581840797499998	0.461177318778743	AMBN	-0.400986413637862
AMBP	-0.111352917482207	0.328572348401464	0.0100283288750826	0.930103038	0.600206930181217	AMBP	0.0918142075275332	0.251237791391631	0.06931073073379292	0.386848977986265	0.393273338960966	AMBP	-0.164286750138956
AMBRA1	-0.0656479997883719	0.565418185223726	0.0451314508276534	0.692313103	0.776521637388717	AMBRA1	0.0483258255988732	0.546525099193733	-0.0437204356527044	0.585440893521958	0.687096562497853	AMBRA1	-0.00219057884100395
AMD1	-0.14969042882039	0.187938095896081	-0.177482960077897	0.117517149	0.44713577055608	AMD1	0.139166476562668	0.081176207379273	0.0869449470874972	0.277363235533152	0.164370213138189	AMD1	0.285058229942426
AMDHD1	0.0389098631555436	0.733513903926921	0.242940603700097	0.031222294	0.86943624919045	AMDHD1	-0.491656388127451	5.3854801382856e-11	-0.465724917016299	6.99664918135599e-10	1.02318564856386e-09	AMDHD1	-0.590727959176915
AMELX	0.0615971770461183	0.589698032516929	0.0565647678486759	0.620500025	0.791231192529808	AMELX	0.030444964337958	0.704140909483493	0.00257850560767998	0.974349233128086	0.807829135887774	AMELX	-0.0958157866294458
AMELY	-0.0144768479446006	0.899234280175824	0.136776870620243	0.229371052	0.95225339149242	AMELY	0.0516008295130027	0.519645909579917	0.110892390447823	0.165408304125873	0.664896144745854	AMELY	-0.193581657368022
AMFR	0.0466689503693795	0.682969955129471	0.115238558909445	0.311183503	0.841166097985237	AMFR	0.0496646950634991	0.535454543883945	-0.0379397239101757	0.636014366506547	0.678174840806816	AMFR	-0.238891299482323
AMHR2	-0.135332485300815	0.23437330416077	-0.121859785783836	0.284054012	0.50653659434764	AMHR2	-0.221302751208693	0.00519995614448925	-0.00791501136746768	0.921375112746979	0.0162780466961481	AMHR2	0.13042089458102
AMICA1	-0.0798814881414313	0.484049211491134	-0.319157740993184	0.004304434	0.722802967525228	AMICA1	-0.450966720407661	2.74370814285585e-09	-0.521201205131298	2.20337969157706e-12	3.70320138636189e-08	AMICA1	-0.447529090734657
AMIGO1	-0.247064218335444	0.0281561474570268	-0.230306718597858	0.041373280	0.137771470659346	AMIGO1	-0.545173426142926	1.30038000544257e-13	-0.488521864401377	7.42756480363861e-11	4.01004956452223e-12	AMIGO1	-0.678233914415558
AMIGO2	-0.293662974941731	0.00862129042235343	-0.302726387536514	0.006887906	0.0622306138588709	AMIGO2	-0.300186527688477	0.000127060878382415	-0.0594165892736023	0.458341741017118	0.000616225176770353	AMIGO2	-0.244004154031095
AMMECR1	-0.140636424026295	0.216371391114144	-0.090968841285297	0.424479924	0.484368236950259	AMMECR1	-0.0110880727457022	0.890025076867474	0.070856189583211	0.375450955525905	0.934384219042552	AMMECR1	0.441397917717634
AMMECR1L	0.249434935355816	0.0266329628112887	0.296981499513145	0.008071803	0.132615246900539	AMMECR1L	-0.0212455316290055	0.791039030836311	-0.0532859923466573	0.50608325606199	0.871150837931237	AMMECR1L	0.319634989266701
AMN	-0.127445869103945	0.263020853227616	0.152178054451071	0.180622320	0.536678678957577	AMN	-0.000621970318183182	0.993811694746102	0.00150754877285421	0.985001341421249	0.996409612257692	AMN	-0.525447049337452
AMN1	-0.428333680165438	8.21598820438049e-05	-0.424391431353457	0.000112521	0.00200433071813905	AMN1	-0.0621092376648391	0.438191450865527	0.155250141285204	0.0514394592421413	0.592211467510964	AMN1	0.280252050652591
AMOT	0.0525031814481777	0.645848243404492	-0.0312560856864654	0.784107893	0.820767695861071	AMOT	0.308399645594076	8.07129198032719e-05	0.0590941763043872	0.460788522676516	0.00041229879400062	AMOT	-0.19432561251693
AMOTL1	-0.00975110296017293	0.932030635610351	-0.0624878286270691	0.583599595	0.967394308747654	AMOTL1	0.252602592601187	0.0013645577867063	0.0886365656892153	0.268086878447627	0.00504549284058305	AMOTL1	-0.204570207878303

[illegible]

[illegible]	0.0822596114477188	0.130087633885102	0.252604502	0.273663682962655	ATG3	0.103139279174358	0.197193561721287	-0.180676145512737	0.0231009924332759	0.328447794380611	ATG3	[illegible]
[illegible]	0.0530517039101588	-0.0996105160662123	0.381668114	0.208599721657082	ATG4A	0.0603737560143170	0.451120514292149	0.135647443518302	0.0892462981770442	0.604157537517646	ATG4A	[illegible]
[illegible]	0.0287237380691574	0.2998539435248 3	0.007459143	0.139262840981484	ATG4B	0.0137169523752383	0.864177850117095	0.150879618959865	0.0584488977908761	0.917503703613363	ATG4B	[illegible]
[illegible]	0.716007034485461	-0.0691090555014606	0.544298450	0.859006573103762	ATG4C	-0.0507243740974977	0.52677255984421	-0.233670237526584	0.0031278435502959	0.670864740201676	ATG4C	[illegible]
[illegible]	0.13325377690096	0.277458617332035	0.013540259	0.37145261800808	ATG4D	0.419461510051131	4.11534131217195e-08	0.300102835897639	0.000127640955549071	4.40229131650701e-07	ATG4D	[illegible]
[illegible]	0.0772318515999685	-0.141066212268744	0.214490685	0.26411443672515	ATG5	-0.144642544137866	0.069794887941079	-0.0593024962786840	0.459206763103488	0.145436559238659	ATG5	[illegible]
[illegible]	0.00845320437148297	-0.436562804284323	6.827040618	0.0616245765054147	ATG7	-0.192019395201151	0.0156482711330694	-0.253508549748803	0.00130936897247433	0.0420930262360911	ATG7	[illegible]
[illegible]	0.0844163278048493	0.2591285296981 5	0.021362285	0.278449988546416	ATG9A	-0.100968547421711	0.206838721811187	-0.0507135756212320	0.52686067178152	0.340294983784089	ATG9A	[illegible]
[illegible]	0.392026826557895	-0.14508276533593	0.201623684	0.654525289183019	ATG9B	-0.0422794662788693	0.597873668269236	0.0273351811341765	0.733153766092952	0.729075593352099	ATG9B	[illegible]
[illegible]	4.69567245446889e-05	0.137390457643622	0.226767652	0.00129527210327037	ATHL1	0.0770481779317035	0.335938217220628	0.116549886059042	0.14474366095591	0.489738117517826	ATHL1	[illegible]
[illegible]	0.00449833917092257	0.146786757546251	0.196335910	0.0396294664242791	ATIC	-0.228361399727858	0.00390297180141028	-0.242476702192717	0.00214326393987271	0.0126236315642943	ATIC	[illegible]
[illegible]	0.262240507286811	-0.0494157740993184	0.664777547	0.535860098165706	ATL1	0.0540177973162333	0.500251461202373	0.140167047357	0.0789920046852703	0.647704302873477	ATL1	[illegible]
[illegible]	0.142798961478036	0.180525803310613	0.111282655	0.386316075497235	ATL2	-6.20000773947409e-05	0.999383123012211	-0.0322578927632428	0.687418693269884	0.999588845017652	ATL2	[illegible]
[illegible]	3.72917675317552e-06	0.426387536514119	0.000103794	0.000156593753342474	ATL3	0.279453618850202	0.000376689594289825	0.267666729798201	0.000673710870924201	0.00161764763957912	ATL3	[illegible]
[illegible]	0.139375084895314	-0.104040895813048	0.360770635	0.381161563752699	ATMIN	0.143483206248207	0.0720893990349518	0.207617304712785	0.00885575653202785	0.149337890878076	ATMIN	[illegible]
[illegible]	0.0799017269049012	-0.129990262901655	0.252962115	0.269510437065241	ATN1	-0.0257428225836878	0.748160277514038	0.00163381168723050	0.983745312480736	0.840419226861519	ATN1	[illegible]
[illegible]	0.428912229378758	0.18947119767108	0.094445448	0.683398539648198	ATOH1	-0.0376412337602167	0.638674753093321	-0.0129061995851616	0.872135171561371	0.761099700467249	ATOH1	[illegible]
[illegible]	0.935924680597950	0.0284834056592960	0.803220528	0.969452096620208	ATOH7	0.0752717714295636	0.347231382037849	0.102610675909750	0.199511699442557	0.501571576564676	ATOH7	[illegible]
[illegible]	0.746023388860018	-0.265968841285297	0.018079494	0.875635136324279	ATOH8	-0.0204193686756254	0.798988794510794	0.0400712858463783	0.617152779821142	0.875711262217247	ATOH8	[illegible]
[illegible]	0.0749238011643089	-0.230014605647517	0.041637214	0.258746802763699	ATOX1	0.0297580984596167	0.710514186320437	0.0986936332209167	0.217305890582081	0.812612451994956	ATOX1	[illegible]
[illegible]	0.363313663030414	-0.0178188899707887	0.875935961	0.630041001965412	ATP10A	-0.117634764097007	0.141015224668806	-0.25507238439915	0.00121888151949813	0.254643480740826	ATP10A	[illegible]
[illegible]	0.983863497832501	-0.111155839189372	0.329434483	0.992743388611921	ATP10B	0.0392294206406869	0.624573264823429	-0.00472344998961890	0.953030378611448	0.749862645454502	ATP10B	[illegible]
[illegible]	0.00615001256899506	0.270837390457644	0.016016803	0.0495274741300781	ATP10D	0.162167392938992	0.0417782681063312	0.120964435692279	0.130028076971774	0.0957592026690186	ATP10D	[illegible]
[illegible]	0.970075775193206	0.0969814995131451	0.394407612	0.986360034477117	ATP11A	-0.100338087441109	0.209702544917790	-0.132270290868719	0.0975806684894959	0.343808340184924	ATP11A	[illegible]
[illegible]	0.0197137754499555	0.145423563777994	0.200558029	0.109522205294725	ATP11B	0.203647815592107	0.0102748330071085	0.0770857910866212	0.335701658422705	0.0293726204442341	ATP11B	[illegible]
[illegible]	0.670775130223042	-0.0172590068159688	0.879807129	0.833799919901409	ATP11C	-0.0400172695925761	0.617627747651648	-0.0621624273513035	0.437798558033426	0.743755025789301	ATP11C	[illegible]
[illegible]	0.427966035718396	-0.125678401194059	0.269752889	0.682550782552894	ATP12A	-0.0362272485756386	0.651339542563457	0.0517960045576160	0.518065632730572	0.770541124443725	ATP12A	[illegible]
[illegible]	0.27253455094172	0.263072054527751	0.019412413	0.545665972162969	ATP13A1	0.418310302818958	4.52008892537492e-08	0.415217875786316	5.80544011095027e-08	4.80732738972572e-07	ATP13A1	[illegible]
[illegible]	0.726124629838901	0.0659201557935735	0.563064152	0.865116485435026	ATP13A2	0.209004659501806	0.00840234307705323	0.119525342716376	0.13469316352087	0.0246121139297613	ATP13A2	[illegible]
[illegible]	0.138623732565760	-0.104100721970151	0.361240812	0.38000002532025	ATP13A4	0.112961082843125	0.157611150835862	0.142891153697669	0.0732845507189513	0.277109636223248	ATP13A4	[illegible]
[illegible]	0.293416342264266	-0.153343545314222	0.177267013	0.565708901361614	ATP13A5	-0.140097743784633	0.0791417493136961	-0.0658017337718800	0.411396741339394	0.161119053949480	ATP13A5	[illegible]
[illegible]	0.216692594276851	-0.118330087633885	0.298313628	0.484758073702925	ATP1A1	-0.00262374689066823	0.973899334123931	0.0268559697638429	0.737659574921838	0.985515678624832	ATP1A1	[illegible]
[illegible]	0.208357365219803	-0.178675754625122	0.115041803	0.474047160855546	ATP1A2	-0.125123107219354	0.117243962946636	-0.130443678582661	0.102338675776041	0.220052884137105	ATP1A2	[illegible]
[illegible]	0.724217056538278	0.14087147030185	0.215129032	0.864065868434430	ATP1A3	0.334189730597343	1.77562703536083e-05	0.237567837896495	0.00265038892747605	0.000106792857631470	ATP1A3	[illegible]
[illegible]	0.151295583477591	-0.115701623208228	0.309914218	0.398286177295327	ATP1A4	0.0131806542179022	0.869439996601215	0.0274811993759953	0.731782622084988	0.920524352060982	ATP1A4	[illegible]
[illegible]	0.000408871864082009	-0.439824732229796	5.953333568	0.00705642908186211	ATP1B1	-0.0290880512277168	0.716750787896252	-0.137228011807904	0.0855458069859941	0.817456449335274	ATP1B1	[illegible]
[illegible]	0.668021026485878	-0.112317429406037	0.323670651	0.832836602485987	ATP1B2	0.0407679063961260	0.611041622366616	-0.0640563710669478	0.423940059457526	0.738383739269406	ATP1B2	[illegible]
[illegible]	0.0706229807433045	0.109469328140214	0.336150968	0.249648134609665	ATP1B3	-0.219417277603165	0.00560613462098914	-0.365782141708169	2.28840393542722e-06	0.0173840219593077	ATP1B3	[illegible]
[illegible]	0.171950774374278	0.205120578372935	0.069766029	0.425584084041355	ATP1B4	0.00197629346502808	0.980338607181142	0.136840095625182	0.0864424387291972	0.988554185145688	ATP1B4	[illegible]
[illegible]	0.272503759222107	0.485199610516066	7.706026425	0.545665972162969	ATP2A1	0.114943113007197	0.150402500417194	0.137205193208920	0.0855983431203596	0.267320914527953	ATP2A1	[illegible]
[illegible]	0.122592410036307	0.125876338851022	0.268389415	0.352127870414708	ATP2A2	-0.0895912652571313	0.262945833469907	-0.223999537543125	0.00466474290488836	0.407237553140489	ATP2A2	[illegible]
[illegible]	[illegible]	[illegible]	[illegible]	[illegible]	[illegible]	[illegible]	[illegible]	[illegible]	[illegible]	[illegible]	[illegible]	[illegible]

C11orf83	0.217280170644883	0.0544180670974483	0.131012658227848	0.249224488	0.211978717942293	C11orf83	-0.103927821147107	0.193771875734591	-0.145906685621358	0.0673609881713859	0.324038535041677	C11orf83	0.0552800579537 62	0.661843687099021	-0.00981217015104631	0.938166028617173	0.721026379096539
C11orf84	0.439861454914923	4.99072541884082e-05	0.406548198636806	0.000226857	0.00135585944139881	C11orf84	0.242371798936301	0.00215310827479742	0.271672981100004	0.000554519770520507	0.00749597642568466	C11orf84	0.405176756941641	0.000813712775550535	0.371547202797203	0.00245621561906061	0.00246347554009809
C11orf85	-0.0852895668014217	0.454852016332526	0.109870189627043	0.335093874	0.701778288756951	C11orf85	0.0401890549741819	0.616117779430162	0.0920852868604322	0.249835080713163	0.742519528627211	C11orf85	-0.185453756415566	0.139132795361143	-0.214403566485335	0.086337279384542	0.202000723455621
C11orf86	-0.0168100209608483	0.883100759722741	0.200735050178709	0.076090165	0.945333332259296	C11orf86	-0.108912846497455	0.173135212674741	-0.0239017399955558	0.765628970368457	0.297722703656079	C11orf86	-0.548991576184224	2.18779806326105e-06	-0.512981118705374	1.30497493255347e-05	1.25481434237558e-05
C11orf87	-0.0266810761852481	0.815444451661742	0.228954236403613	0.042397331	0.912244559098441	C11orf87	0.0329175756587671	0.681370731416844	-0.0304765208025846	0.703848593773868	0.791821434502215	C11orf87	0.241296851026414	0.0528226717634235	-0.157736013986014	0.208983744675941	0.0891151717687127
C11orf88	-0.271175654595452	0.0156350144972354	-0.366429465380411	0.000895965	0.0931975035341536	C11orf88	-0.0266489535656008	0.739608830281121	0.0667277191423851	0.404831542083705	0.834632742765443	C11orf88	-0.411910763212754	0.000651552052625996	-0.410275465950431	0.000687976601353596	0.00203533120269 8
C11orf92	-0.240238491683011	0.0329583694747915	-0.346178188899708	0.001880835	0.15243865522272	C11orf92	0.260491613249684	0.000947773597 76943	0.111041387614249	0.164837283999702	0.00367105237427629	C11orf92	-0.112098463906819	0.373983026430703	-0.187631118881119	0.134265700109318	0.455362330694237
C11orf93	-0.299979330254801	0.00723270728889596	-0.328860326566013	0.003084765	0.0552654054012005	C11orf93	0.0778189145567242	0.331112076817364	0.168068108954295	0.0347854100822018	0.484830456868502	C11orf93	0.101407727025674	0.421520152205555	-0.0543706293706294	0.666359299195076	0.501639521354537
C11orf94	-0.1252295547308	0.271480769978821	-0.123746218339538	0.277243798	0.544815716920914	C11orf94	-0.00983043425872654	0.902433008436941	0.0613865949858588	0.443549206562349	0.941931720451 53	C11orf94	-0.221722493327528	0.0758871612468426	-0.143008556609013	0.255768879722316	0.121101540307095
C11orf95	-0.128855022207164	0.25773572483464	-0.168549172346641	0.137385815	0.531487557687364	C11orf95	-0.199006416605092	0.0121852197088105	-0.129017879893581	0.106178177537578	0.0339430919714331	C11orf95	0.0457208991802293	0.717613151790386	0.103671328671329	0.410245784334981	0.770336995835082
C12orf10	-0.0810960248993633	0.477406679953766	-0.0866114897760467	0.447074761	0.717652148371634	C12orf10	0.0931034380401185	0.244615056030392	0.0113940870925107	0.887009835942044	0.385396934 66149	C12orf10	0.0214433874578819	0.8653663331545	-0.01429195804 1958	0.909914242 3 2991	0.893132097854644
C12orf23	0.0558257422023336	0.625080633820709	0.126703992210321	0.265235647	0.809086699980758	C12orf23	0.331513144105033	2.09121566956405e-05	0.434758202715995	1.14381228254061e-08	0.000123704486924918	C12orf23	0.668810903419721	1.13646403399059e-09	0.674256993006993	0	9.44402835565915e-09
C12orf29	-0.00985007727393397	0.931342405724421	0.0349805258033106	0.759115451	0.966984623886692	C12orf29	0.23034744010222	0.00359477418194043	0.224054302180686	0.00465440568479993	0.0117757638237586	C12orf29	0.616414532941238	4.58599453967423e-08	0.586756993006993	4.96968188974543e-07	3.32534857667048e-07
C12orf39	0.0810442597064199	0.477688792297521	-0.0412959129257176	0.717837909	0.717912532983311	C12orf39	-0.0176773087929646	0.825518212465073	-0.0122429389747 03	0.878654477162484	0.893304548 9615	C12orf39	-0.267952388903392	0.0309266188553724	-0.405026223776224	0.000898209354825 34	0.0565543223600809
C12orf4	0.388315064488594	0.000405846142955468	0.385735150925024	0.000491285	0.00701645543301857	C12orf4	0.352954450367299	5.40030979734318e-06	0.282545977575484	0.000322006142401038	3.64575369890311e-05	C12orf4	0.708102909623487	4.19726475797688e-11	0.676180069930 07	0	3.73161811122263e-10
C12orf40	-0.045259730005569	0.692054596486873	-0.0577426481322524	0.613229112	0.846367537058952	C12orf40	-0.104035999205721	0.193305851853744	-0.0137869975059311	0.863491003876823	0.323572130951777	C12orf40	-0.421384843724778	0.000472834643389216	-0.388068181818182	0.00151535824925 86	0.00154085413548533
C12orf42	-0.0482528288526171	0.672812892098791	-0.0481168032416744	0.673682932	0.835125729881379	C12orf42	0.0750224787592577	0.34883513742956	0.115952391094556	0.146828785248517	0.503340739770782	C12orf42	-0.367526301210037	0.00259732032859564	-0.408566433566434	0.000802865253125077	0.00669791101328082
C12orf43	0.0256590611361854	0.822395872647279	0.109785783836417	0.334749394	0.915583764098952	C12orf43	0.115632405424708	0.147954778271636	0.212763821235917	0.00727544368816278	0.264297250919932	C12orf43	0.610382977741007	6.72490017006311e-08	0.671722027972028	0	4.76879020020397e-07
C12orf44	0.230644860813443	0.0408546942704637	0.426850048685492	0.000101863	0.176540154164811	C12orf44	0.49794590129115	2.79745115960 85e-11	0.426371606969532	2.32610594438744e-08	5.58579998355667e-10	C12orf44	0.507687210193664	1.58428506771 28e-05	0.444493006993007	0.000240379124403 24	7.70032364495875e-05
C12orf45	0.0426575955550619	0.708943297005604	0.0646299902629017	0.570742770	0.856206665430148	C12orf45	0.398039973852634	2.22847885256172e-07	0.407814001036082	1.04628067992 06e-07	2.04314221492337e-06	C12orf45	0.392521791534746	0.00122042422552671	0.353846153846154	0.00402225765572422	0.00350673876531 58
C12orf49	-0.142954432896892	0.208818532670602	0.0291626095423564	0.798254697	0.474659572366299	C12orf49	0.247790147130816	0.00169499301068932	0.423318478425517	2.99772508115039e-08	0.000608518356606677	C12orf49	0.570797971877863	6.89034173273484e-07	0.573208041958042	9.98124993883673e-07	4.28748830011 12e-06
C12orf5	-0.0270894938495959	0.812670469851862	0.136806231742941	0.228763536	0.910857789455408	C12orf5	-0.0820056692550111	0.305676201931173	-0.00582026398076713	0.942140911890462	0.457334325684916	C12orf5	0.222895053178359	0.0743107727369416	0.234596094813991	0.0599894169781854	0.118864389570244
C12orf50	-0.0559931804511916	0.624041569793131	0.0912811467929368	0.423673160	0.808753614762809	C12orf50	-0.0150979235013692	0.850655764327825	0.0326716948755473	0.683622617543 73	0.909388108778541	C12orf50	0.00807433469776346	0.949101383286065	-0.215034965034965	0.0854646254846894	0.960464390321824
C12orf52	-0.142250695903007	0.211091471259948	-0.106937682570594	0.347497193	0.477716834898319	C12orf52	-0.0706621756281442	0.377635913885209	0.0195053384112379	0.807808135861243	0.533436443998059	C12orf52	0.112166970658242	0.373689252788661	0.0186625874125874	0.882496568264506	0.455124268751861
C12orf54	-0.0388103092880806	0.734170383619638	-0.218784267035658	0.052728225	0.869761255868 21	C12orf54	-0.0932686146608507	0.243775402348726	-0.0293721006117751	0.714104627286929	0.384414797654015	C12orf54	-0.262044105498874	0.0349726539390313	-0.310751748251748	0.0120424360822432	0.0626957709751502
C12orf56	0.0250408379792198	0.826607512540154	0.0310212767810811	0.786088294	0.917329333577554	C12orf56	0.106415970777329	0.183258490244783	0.0922223388509899	0.249127965923415	0.310490410952186	C12orf56	0.262126461322005	0.034913364815909	-0.155140575058497	0.217202346958438	0.0626137160551 69
C12orf57	0.203891293166353	0.0714944662112362	0.120301850048686	0.290290871	0.251291550155648	C12orf57	-0.211384511901194	0.00767228915255991	-0.306747383657 56	8.85233305992567e-05	0.0227157998228736	C12orf57	0.108096589181616	0.391385212473135	0.0410402097902098	0.744937440395941	0.472010077112987
C12orf60	-0.282479932324282	0.0116585750586692	-0.300389483933788	0.007349573	0.0768354873410061	C12orf60	-0.335169914556417	1.67173827524316e-05	-0.308983606357 96	7.8110306286416e-05	0.000101282400739452	C12orf60	-0.517516763359807	1.01232029813452e-05	-0.558216783216783	2.09857216378595e-06	5.13027092008654e-05
C12orf61	-0.00654778229949847	0.954329375641 44	-0.0408227848101266	0.720415189	0.978712342042286	C12orf61	-0.138896218759671	0.0817744223161855	-0.148783350333233	0.0620797116060622	0.165345097731024	C12orf61	0.0923863876830285	0.464194165576769	-0.160076049791568	0.202746652774964	0.541749894654375
C12orf65	-0.139069742566206	0.221584112250071	-0.0832765335929893	0.464813803	0.490791977815125	C12orf65	0.540435828268167	2.31592522936808e-13	0.437450797396068	9.06968310652 63e-09	6.80002926198825e-12	C12orf65	0.658545586622446	2.48323939189277e-09	0.695760489 51049	0	2.01583681444923e-08
C12orf66	-0.1558428217492	0.170225774237459	-0.0602239532619279	0.597329621	0.423314178800687	C12orf66	0.299115164049276	0.000134675927936367	0.349533777991867	6.74753661484138e-06	0.000649393356026327	C12orf66	0.279269934015392	0.0242667129492484	0.292526223776224	0.0183596326028224	0.0458490024395981
C12orf68	-0.0362610692863821	0.751044311070802	-0.180890944498539	0.110552132	0.878748393750877	C12orf68	0.256565289367417	0.00113787146791822	0.21452069152531	0.00679644030943078	0.00430501720599557	C12orf68	-0.00874049480907472	0.944908344942849	-0.053889860139 8601	0.669137611925854	0.957183102343366
C12orf73	-0.201131044839872	0.0755009303226181	-0.175851996105161	0.120968204	0.260194702896104	C12orf73	0.174441347113112	0.0283709275666655	0.247981885074959	0.00168054439630989	0.0694831298444199	C12orf73	0.625911754779787	2.46852536189834e-08	0.62416958041958	6.17566861343 77e-08	1.84303827793357e-07
C12orf74	0.0173498094292518	0.879374724832279	0.0616368522951458	0.589457958	0.943443565611544	C12orf74	0.0628796106595981	0.432520619635951	0.0970353315580744	0.225170068779924	0.587350048498772	C12orf74	-0.342798448484538	0.00518446128692676	-0.364220466529031	0.0028577361875 1151	0.0122175253814344
C12orf75	-0.229352463950161	0.0420296814580351	-0.176606621226874	0.119361861	0.179849208108399	C12orf75	0.292591705188914	0.000191027528333887	0.290254100312166	0.000216081488493266	0.000886517972429256	C12orf75	0.458849302325958	0.000120942238671429	0.488111888111888	4.65723986105863e-05	0.000467694481153831
C12orf76	0.165697574114691	0.144458657011655	0.328140214216164	0.003294034	0.389265502369504	C12orf76	0.277317387886205	0.000419355647280906	0.425938377565953	2.41172451305554e-08	0.00178298786758345	C12orf76	0.599771196561 3074	1.29856019714936e-07	0.643356643356643	1.74079433700528e-08	8.89519496183945e-07
C12orf77	0.0942739124614073	0.408570552852334	0.122465111417666	0.282286423	0.668213709940748	C12orf77	-0.078987281358868	0.323881222680786	-0.167015537956486	0.0359547843865092	0.477316515598584	C12orf77	-0.0760204136747585	0.547256706727 22	-0.0811058905509268	0.520698991101 24	0.618507970320243
C14orf1	0.114185918951142	0.316337890497574	0.1980282375852	0.080271923	0.58879454152654	C14orf1	0.219774206027575	0.00552713485445722	0.229828928963486	0.00367304812835386	0.0171911729465378	C14orf1	0.265965574554206	0.0322410299479523	0.256949300699301	0.0390866921013 26	0.0586154528294666
C14orf105	-0.108335992099159	0.341927207436118	-0.0827888668487345	0.468230384	0.612606638696734	C14orf105	-0.0182975594386386	0.819498840531813	0.117972695140698	0.139868908914131	0.889090120831556	C14orf105	-0.469193225348461	8.06365820287817e-05	-0.407779720279 72	0.000823219836527142	0.000327754166336712
C14orf119	0.0506457254184913	0.657578643387274	0.123344693281402	0.278193289	0.826879790196347	C14orf119	0.146384324953878	0.0664595684471765	0.152641214801404	0.0555345043905866	0.139894883959812	C14orf119	0.151891242231835	0.227105628784313	0.140603146853147	0.263230519439 78	0.303821994145373
C14orf132	-0.0760131733470769	0.505529176418513	0.0905063291139 24	0.426846590	0.737517292379727	C14orf132	-0.0727412760275656	0.363726376825149	-0.178695491120955	0.0246755103324218	0.519165963509924	C14orf132	-0.280690254712915	0.0235323636413779	-0.424475524475525	0.000477947019413761	0.044637292393795
C14orf142	0.437583615285902	5.51521999869742e-05	0.339435248296008	0.002327681	0.00147206506254032	C14orf142	0.0553771160666643	0.489513379299717	-0.0183978757405639	0.818526275734474	0.639170435366015	C14orf142	0.171482088815156	0.171986458335771	0.149038461538462	0.235435675770101	0.241406446973119
C14orf159	-0.276068343198117	0.0137895036250758	-0.235978578383642	0.036518833	0.0864528721289621	C14orf159	-0.216118986881213	0.00638526963951121	-0.0626522666094862	0.434189741637029	0.0194205871573437	C14orf159	-0.13127344759148	0.297252346131808	-0.164204545454545	0.190731975379249	0.378928023800786
C14orf166	0.135626132021378	0.233350249722743	0.187731256085686	0.097545202	0.505042601249931	C14orf166	0.213809444555602	0.00698686973737872	0.0363561407939053	0.650180872652169	0.0209663034825008	C14orf166	0.122651281984408	0.330384001757268	0.0561625874125874	0.656043570518775	0.412601427915827
C14orf166B	-0.0195733079770138	0.864054892313231	0.110996854741 17	0.330131019	0.936655393815899	C14orf166B	-0.00385080359556554	0.961700555056726	0.111798965100506	0.161956645960531	0.978195646221305	C14orf166B	-0.390450985946443	0.00130216389310332	-0.349868881118881	0.00447783662387842	0.00370994166124651
C14orf169	-0.0104481420834366	0.927184778353486	-0.135467380720545	0.233383823	0.965252160557703	C14orf169	-0.0298047515171187	0.710080661788021	-0.0376177933053507	0.638883869217859	0.812365460074414	C14orf169	0.0714862061028412	0.571473915868397	0.0417181131811746	0.741430375653797	0.640649751226867
C14orf178	-0.0348862275700108	0.760193929880239	0.135919671717637	0.232330674	0.883586950233 39	C14orf178	0.235481571985319	0.00289705444893706	0.158844831245097	0.0462100410763983	0.00971975860776754	C14orf178	-0.383038996712144	0.00163669784893461	-0.42229020979021	0.000513970141031 866	0.00450938879149721
C14orf180	-0.163182019898308	0.150738845485383	-0.346202904569947	0.001776081	0.397505340346379	C14orf180	-0.27560439792504	0.000456750946307174	-0.259104458540295	0.0010113254361 2281	0.00192552894928213	C14orf180	-0.542647913372572	3.01679491582423e-06	-0.52534965034965	9.67655044137666e-06	1.68928844899635e-05
C14orf183	0.0533578892108174	0.640479154644807	0.133378299920064	0.241261321	0.818477179636381	C14orf183	0.0204578966676519	0.798617592429425	-0.0126095673894 59	0.875049785654976	0.875516576944305	C14orf183	0.0830680095800161	0.510628450945117	0.0491258741258741	0.696903649583443	0.584440569502788
C14orf2	-0.0650236295922033	0.569129664639628	-0.0253651411879 26	0.824080628	0.779593185786246	C14orf2	-0.184711811120866	0.0201584923849528	-0.108491789487807	0.174812676254255	0.0522259843522349	C14orf2	0.0972265825572521	0.441012694815 45	0.111756993006993	0.374545132297189	0.519894839251034
C14orf23	0.248129537997534	0.0274628109015982	0.00486935040557136	0.966028263	0.135518830841 27	C14orf23	0.00820124917423668	0.918541361913991	0.0168492534895391	0.833570075787 3	0.951623324301439	C14orf23	0.227197681377147	0.068748800407595	-0.147925567377355	0.239612366839406	0.111196473387604
C14orf28	-0.112149887381325	0.325100582889372	-0.0868792599805258	0.445667107	0.597170777301677	C14orf28	0.00876424186123597	0.912970743292881	0.0481487650954679	0.547997501071174	0.948349114976251	C14orf28	0.0807726446160507	0.522419233704111	0.111355594405594	0.368567155734885	0.595786362644633

[illegible]

CACNG5	-0.0937479552415103	0.411201722895578	-0.0318497596085128	0.780516997	0.670630314239521	CACNG5	-0.0581421771044338	0.468055247280755	0.00115462110857351	0.988512354811544	0.619920654851705	CACNG5	-0.537263687707335	3.94245058653313e-06	-0.5788898601398	7.47312931659769e-07	2.16213960653818e-05
CACNG6	-0.0635433619343116	0.577974183793367	0.0643965169771	0.572868887	0.783982217159216	CACNG6	0.0289004480609331	0.718500321067825	0.0347420775725979	0.664750016622198	0.818792380740245	CACNG6	-0.557212130494588	1.42846956920894e-06	-0.541477272727273	4.64962506367565e-06	8.45689039439649e-06
CACNG7	-0.0959763544562028	0.400121527537916	0.0917159937460645	0.421459091	0.660512776199391	CACNG7	0.15205026814192 5	0.0564985033608205	0.0483602542298109	0.546239022714145	0.12267514562838	CACNG7	-0.300960918769568	0.0148459566659854	-0.340821678321678	0.0056888017220027	0.0302031233647419
CACYBP	0.223848429573038	0.0473519681446426	0.136441090555015	0.230017203	0.194220813479491	CACYBP	0.519722757325495	2.60480526037554e-12	0.33055783107705	2.2161384543487e-05	6.19558483369856e-11	CACYBP	0.533786589170887	4.67479103738455e-06	0.541083916083916	4.73532952524792e-06	2.53081253917312e-05
CAD	0.638188336892598	2.50756304609467e-10	0.58468841285297	2.927382742	2.72497702888244e-08	CAD	0.407574913801495	1.06612067618528e-07	0.0737953491131765	0.356797302676096	1.0440043430091e-06	CAD	0.432478369063945	0.000320932031504295	0.266083916083916	0.0324616778746479	0.00109919932706884
CADM1	0.144033814934571	0.205366204135712	0.135418695228822	0.233553053	0.469895739090843	CADM1	0.11003021127925	0.168741515921161	0.0630447465120052	0.4313105300098	0.292199954664468	CADM1	0.206334790342132	0.0991385854189193	0.191346153846154	0.126621607418219	0.151885270774768
CADM2	-0.0108039840474 63	0.924711953280842	0.107404412918121	0.346118806	0.963987541223583	CADM2	-0.0437408775189547	0.585265378319042	0.0360031854764285	0.653355731221117	0.718890820313155	CADM2	0.0603724230129156	0.632842458482171	-0.1339597902097 9	0.286628148085441	0.695183016952833
CADM3	0.0563512173762074	0.6218221766146	0.182950250104394	0.106564005	0.808041984828092	CADM3	0.11772399570904	0.140711844245645	0.12305157687931 8	0.123484193155359	0.254145497161025	CADM3	-0.390759002713762	0.00128970170890579	-0.393662587412587	0.00127783188 6393	0.00368032178145122
CADM4	-0.156264027476029	0.1690596303805	-0.239289191820837	0.033913014	0.422194636355525	CADM4	-0.0890239840088939	0.265992450036067	-0.0207907861539844	0.795412218477333	0.410677709255618	CADM4	-0.506194197889695	1.6937743922392e-05	-0.537587412587413	5.56527002763588e-06	8.15547087822451e-05
CADPS	-0.198552981305239	0.0794033234989566	-0.311542022812012	0.005192758	0.268595844222337	CADPS	0.0372004301020784	0.642611982545337	-0.128152294372134	0.108563710370045	0.764342072532605	CADPS	0.0600775975894066	0.634507099802058	-0.271153846153846	0.0292108715981 63	0.696433772153874
CADPS2	-0.0203288991263339	0.85885998704123 9	0.0473466407010711	0.678023138	0.933763955451664	CADPS2	-0.321097834691266	3.89611322558675e-05	-0.0981717724588428	0.21975932772094	0.000214498954341 36	CADPS2	-0.2386037397173	0.0556067068887907	-0.185183566433566	0.139490013016534	0.0930272437422147
CAGE1	0.394381233600675	0.000322652966837644	0.289536706166346	0.009649845	0.00590326584469466	CAGE1	-0.0869454031430523	0.277360705826086	-0.0186276396445945	0.816299762827425	0.424209570013977	CAGE1	-0.171334221607457	0.17236207970437	-0.13892896506743	0.269718670916997	0.241787145121333
CALB1	-0.138293744214058	0.224198451607972	0.000827653359298929	0.994295621	0.493837014774007	CALB1	0.416601850302864	5.19192520087586e-08	0.47853188176632 8	2.02543270591363e-10	5.45255806891755e-07	CALB1	0.543364057780915	2.91028516574698e-06	0.416783216783217	0.000616011719615 9	1.63399283487758e-05
CALB2	-0.236282864441289	0.0360433988219013	-0.270230466353996	0.016015087	0.161867925045314	CALB2	-0.377665966319059	9.98702888261708e-07	-0.114888603402895	0.150597364430422	7.96280648458122e-06	CALB2	-0.150608807246783	0.231099376366807	-0.106818181818182	0.396121596657592	0.308274482009863
CALCA	0.163491572066878	0.149955209381033	0.196708129693626	0.082293292	0.39618142280765	CALCA	0.12928327092881	0.105455070633944	0.115748103964613	0.147546896588229	0.202738915034556	CALCA	-0.323153019681594	0.00864850627339723	-0.337237762237762	0.00624343276002006	0.0190001333041055
CALCB	-0.0792339713611327	0.487610581714887	-0.0089105339027843 5	0.937877592	0.725363724013806	CALCB	-0.00526606442603338	0.947641943809682	-0.000377270659762 98	0.99624631499334	0.969442553097146	CALCB	-0.174438452203952	0.164601499609682	-0.168669959512484	0.17923293762085	0.232738923601643
CALCOCO1	-0.393304490645817	0.000336169786354086	-0.330574488802337	0.003059663	0.00608861358471714	CALCOCO1	-0.353814853076076	5.10400708183305e-06	-0.32083862914994	3.95577607672827e-05	3.46733470024157e-05	CALCOCO1	-0.227565168332965	0.0682896669775027	-0.277972027972028	0.0252759812476212	0.11054649382812 3
CALCOCO2	-0.286320465700121	0.0105243959248597	-0.426363193768257	0.000103896	0.0711142817225402	CALCOCO2	-0.446644920195925	4.04430121330077e-09	-0.454843133541418	1.92835104478639e-09	5.3144600525958e-08	CALCOCO2	-0.611261377309666	6.36341220377627e-08	-0.607954545454545	1.58012001527662e-07	4.5249194546963e-07
CALCR	-0.137342913671329	0.227431172717473	-0.207242213490065	0.066862129	0.4978565283902 8	CALCR	-0.0505038661493857	0.528574979441433	0.0277070805047771	0.729663219047159	0.672306686485256	CALCR	0.0844153422922036	0.503771298793507	-0.359222027972028	0.00347216603593374	0.578192342537173
CALCRL	0.129337851067011	0.255941569150975	0.00946932814021422	0.933920296	0.529887192993613	CALCRL	-0.136969043109159	0.0861435584885907	-0.0736143215612394	0.357981414178436	0.172643469603196	CALCRL	0.281813120486988	0.0229495621572378	0.296416083916084	0.0168132302356125	0.0437314348824772
CALD1	-0.0021397570039512	0.985068101873308	-0.211635832521908	0.061296581	0.993413284917493	CALD1	-0.38435265792962	6.17248982189047e-07	-0.363442474692377	2.68382069789382e-06	5.16995166610571e-06	CALD1	-0.523146249401979	7.78338398481397e-06	-0.471765734265734	8.82438138265885e-05	4.03544152872826e-05
CALHM1	-0.148987930910302	0.190042334518731	-0.130813819514304	0.250509814	0.44977911106011	CALHM1	0.0890167605315628	0.266031398668733	0.17572021312609	0.0272133675629781	0.410703400376635	CALHM1	-0.216628845973328	0.0830465165731992	-0.327622377622378	0.00797402282162341	0.130622636119066
CALHM2	0.0641437801971475	0.57437908049446	-0.00569620253164557	0.960255507	0.781458998006427	CALHM2	-0.279663092701463	0.000372729791750112	-0.0883231904661106	0.269789211732577	0.00160438519198493	CALHM2	-0.105043085113973	0.404980394955356	-0.0461975524475524	0.714173792310766	0.48539802034705
CALHM3	0.0976463954922924	0.3919342111745	-0.0706079853490896	0.536346693	0.654425903639448	CALHM3	0.0671744242141501	0.401686734271245	0.11008511382853	0.168527784071107	0.557607616983721	CALHM3	-0.355734053980857	0.00363576658991254	-0.371765734265734	0.00244090738938896	0.00899619169042462
CALML3	-0.0790822604571 48	0.488446998367421	0.0757627521711182	0.506936468	0.726005162968476	CALML3	-0.0180262436320898	0.822130616393803	0.121725055658401	0.127613082049962	0.890832659802912	CALML3	-0.393948211784232	0.00116684087328514	-0.387412587412587	0.00154363648556 89	0.00337126180455138
CALML4	-0.171117772217134	0.131597805054649	-0.232546251217137	0.039395797	0.368816974404258	CALML4	0.0249017635313634	0.756125197767992	-0.0992076621813194	0.214908407842693	0.846165272075177	CALML4	0.0496153584358833	0.694694597347271	-0.0106643356643357	0.932752632375871	0.75025515700477
CALML5	-0.0819002214161308	0.473035433094483	-0.0295306752690951	0.796139056	0.714993316986762	CALML5	-0.117466160627169	0.141589822138487	-0.0846798208283853	0.290119990552606	0.255505648458101	CALML5	-0.371358769952728	0.00232208086282276	-0.322184464447385	0.00886216003114988	0.00608226563933218
CALML6	0.105602433351629	0.354317348650761	0.294986236069935	0.008312443	0.622691367850683	CALML6	0.000464457093314484	0.995378855210552	-0.0242474490311949	0.762339473796959	0.997274814934763	CALML6	-0.503524691668036	1.90725861348086e-05	-0.538680069930 07	5.29225408033941e-06	9.07211121138154e-05
CALN1	-0.169760731745582	0.134732977844209	-0.0593003724706489	0.603670564	0.37349386743383	CALN1	0.0807860947414926	0.312949366807207	0.0287255736405713	0.720132466911348	0.465266723970014	CALN1	-0.163380902082475	0.193457599971619	-0.242089160839161	0.0522503602031068	0.265856406298148
CALR	0.047473987687794	0.677800255731349	0.057935735150925	0.611352443	0.837793479023475	CALR	0.25536336618766 3	0.0012026896000854	0.256045977955786	0.00116547448502877	0.00451223059661142	CALR	0.313684933010915	0.0109429917195347	0.359746503496504	0.00342227156402841	0.0232257778798042
CALR3	-0.260630829057392	0.0203482799241872	-0.31484233697625	0.004712940	0.111574239074182	CALR3	0.0967002031388036	0.226783499344875	0.0251932545178997	0.753361791245012	0.364317995020868	CALR3	-0.136367180333209	0.278735443780933	-0.13208041958042	0.293489999105051	0.359658637136688
[illegible]																	

CDH1	-0.120989431837843	0.288170023624859	-0.0251411900290891	0.825923529	0.561353957180386
CDH10	0.267607861381331	0.0171116452600937	0.126997789949312	0.264716665	0.0993057863715179
CDH11	0.195010395164077	0.0850260937173717	0.325900681596884	0.003523738	0.279425777726539
CDH12	-0.0883850626288237	0.438588069239677	-0.111016434176133	0.330045188	0.689899114809497
CDH13	0.320216695843919	0.00401539298059816	0.248855890944499	0.027246711	0.0363814367828315
CDH15	-0.0922335486546487	0.418832598276465	0.188842362310765	0.095564663	0.676052487448945
CDH16	-0.105944976737226	0.352749679103799	-0.164183178703645	0.148215408	0.621586168328131
CDH17	-0.101479773065747	0.373522010997818	0.0219042989767212	0.848048156	0.638727160082556
CDH18	-0.105360866053849	0.355425485122745	-0.0649789706092463	0.569395570	0.623578927060911
CDH19	-0.0441915354693695	0.69896993597154	0.111994382307133	0.325776150	0.850829030970162
CDH2	-0.0984090668078033	0.388228631820411	-0.0767039922103213	0.500876041	0.651594185348276
CDH20	0.0162715232930239	0.886820412773644	0.0190604393076429	0.867584310	0.946971975940793
CDH22	-0.209104354268386	0.0643945226688844	-0.167644553889661	0.139734482	0.235119638815663
CDH24	0.107763610144683	0.344498828905337	0.19493670886076	0.085179275	0.614469190779621
CDH26	-0.232068272696679	0.0395923050042189	-0.296268822695602	0.008022394	0.172434028286521
CDH3	0.212576491229493	0.0599904585440798	-0.0664698557252217	0.560550233	0.225226136880184
CDH4	-0.0541091903097502	0.635774819684853	0.0905973470973 01	0.427168258	0.815591554281482
CDH5	0.0986480455001353	0.387071829217426	-0.11292599805258	0.321042994	0.650434648756126
CDH6	0.0343061455304774	0.76406436723027	-0.0105647517039922	0.926286654	0.886164141771111
CDH7	0.0219032201479537	0.848055551437131	0.0151533075219248	0.894552169	0.927958102147916
CDH8	-0.085745286599954	0.452436933960512	0.000656881639657417	0.995415835	0.69874670897332
CDH9	-0.12732204435205	0.26348874674003	0.105086524883098	0.356686560	0.536942688629362
CDHR1	0.218689262562239	0.0528336748863207	0.0545520934761441	0.632346988	0.207907764007491
CDHR2	-0.0678959890498139	0.552150616373951	-0.364430921750557	0.000960551	0.76778390161347
CDHR5	0.171358152155942	0.131048282077742	0.241650794368305	0.031912005	0.367902473092063
CDIPT	-0.343142470948224	0.00196228683928539	-0.241723466407011	0.032098418	0.0221096811916778
CDK1	0.786201280120655	0	0.805550146056475	0	0
CDK10	0.298422002519843	0.00755540774103247	0.0192307692307692	0.866187066	0.0570346772145573
CDK12	-0.105539751261881	0.35460468309658	-0.124561830574489	0.273450313	0.622967793777191
CDK13	-0.0346847229705454	0.761537752071281	0.0726874391431354	0.523612253	0.884421236668573
CDK14	-0.226248239315624	0.0449667941521629	-0.262560856864654	0.019656205	0.18778573790994
CDK15	-0.0694826436570066	0.54287735394664	-0.124255485700224	0.275256063	0.761666085612728
CDK16	-0.0465530010427838	0.683715755095087	0.16166017526777	0.154374804	0.841369723502236
CDK17	0.431321006126222	7.23274963205789e-05	0.350243427458617	0.001650463	0.00181823618213768
CDK18	0.0649351186264194	0.569656727039599	-0.00333495618305745	0.976759831	0.779681156270704
CDK19	0.0112079324836358	0.921905690978592	0.124318403115871	0.274394523	0.96264456766668
CDK2	0.607501086760635	2.91335622293332e-09	0.600219084712756	9.36227646[illegible]	2.62339224654795e-07
CDK20	0.0167351408078634	0.883617842302286	-0.134444985394352	0.236955657	0.945425679472795
CDK2AP1	-0.0181205301429512	0.874059173661955	-0.0311830574488802	0.784600234	0.94167201173266
CDK2AP2	0.179358689744564	0.113736344474863	0.174951314508277	0.122907203	0.335042809208198
CDK4	0.016599713102697	0.884553157453394	0.0919912366114898	0.419275249	0.946016998221664
CDK5	-0.00583794485169566	0.959276001171818	0.0412852969814995	0.717379893	0.981457801002339
CDK5R1	0.0980893111379133	0.389779668573628	0.11263388510224	0.322302551	0.652999372697861
CDK5R2	-0.128818063888539	0.25787341066113	0.00976262940890996	0.931950482	0.531665136446516
CDK5RAP1	-0.0509245312423678	0.655812527887218	-0.0534810126582279	0.639055132	0.826285038516639
CDK5RAP2	0.350648998352511	0.00153387263353499	0.390652385589094	0.000411060	0.0184749658676231
CDK5RAP3	0.0548410455722198	0.631206055584353	-0.0402872444011685	0.723935170	0.812865826757868
CDK6	0.118740593555142	0.29729108045579	0.178821811100292	0.114741496	0.569971208729606
CDK7	-0.0764155821681581	0.503271990430896	0.0351509250243427	0.757977698	0.736483743401464
CDK8	0.0230362364009097	0.840296919277381	0.163242453748783	0.150340098	0.92432444378321
[illegible]	[illegible]	[illegible]	[illegible]	[illegible]	[illegible]

CDH1	0.191120188475432	0.0161510216266754	0.0820845855039763	0.30520942529317	[illegible]
CDH10	0.231343785801396	0.00344857145177446	0.127542374170543	0.110269709923049	[illegible]
CDH11	0.197290759033666	0.0129665488834998	0.246448475223256	0.00179930414007706	[illegible]
CDH12	-0.0932671425353473	0.243782876839252	-0.167995217218015	0.0348653407807075	[illegible]
CDH13	0.200686395120235	0.0114604869529515	0.375416154199073	1.17141384934571e-06	[illegible]
CDH15	-0.0977159530226938	0.221918336855916	-0.0262809410694545	0.74307810565695	[illegible]
CDH16	0.0528844625082661	0.509298054210842	-0.00543691565326656	0.945945784268356	[illegible]
CDH17	0.00484223961367669	0.9518505474432	0.044643558000359	0.579069198190337	[illegible]
CDH18	-0.0788074392818456	0.324987555531747	-0.0229068481231359	0.77511877192029	[illegible]
CDH19	-0.225170066711347	0.00448221185727 56	-0.266696381575945	0.000705927288391498	[illegible]
CDH2	-0.439922864330061	7.31655134477365e-09	-0.491613023943698	5.40960654389737e-11	[illegible]
CDH20	-0.0311836370188143	0.678937186402223	-0.0225524163031597	0.778507665106279	[illegible]
CDH22	0.0932488758673	0.243875636296127	0.0803199471825728	0.315758893998778	[illegible]
CDH24	0.22855050490979	0.00387263465205212	0.162678355874353	0.0411294130458653	[illegible]
CDH26	0.0419383805879233	0.600833869916974	0.0268103325658755	0.738089149712159	[illegible]
CDH3	0.160534742130061	0.0439092984811353	0.0598501426542919	0.455062924603782	[illegible]
CDH4	0.129545357736172	0.104744769987183	0.1460406658168	0.0671071357394591	[illegible]
CDH5	-0.261262350018965	0.000914066792702287	-0.0504078063948509	0.529358734574203	[illegible]
CDH6	-0.0387282739801739	0.629008574398573	0.0482035297330287	0.547541881317466	[illegible]
CDH7	-0.000267378098674324	0.997339695099389	0.0232369399650334	0.771966417733949	[illegible]
CDH8	-0.0216989195458682	0.786685373997587	-0.000760619966122207	0.992432234728613	[illegible]
CDH9	-0.0176072621789437	0.826198642501008	-0.0970414903294704	0.225140494379149	[illegible]
CDHR1	0.125652695520169	0.115688675960831	0.0302650684520026	0.705808157418747	[illegible]
CDHR2	0.00738408098604255	0.926633908733256	0.0505721387736971	0.528015436428503	[illegible]
CDHR5	0.0484185632387294	0.545754685644509	-0.135355365451311	0.0899438994932805	[illegible]
CDIPT	0.0980293457188842	0.220432328675235	0.181850542740429	0.0222088338853837	[illegible]
CDK1	0.854351344076699	0	0.640159060847501	1.35597121263287e-19	[illegible]
CDK10	-0.100948741150095	0.206928260579252	-0.181737970985443	0.0222930460187081	[illegible]
CDK12	-0.101641797693437	0.203811685888178	-0.0657845498670003	0.411519164384711	[illegible]
CDK13	0.463369664987625	8.73980443572009e-10	0.428930332535567	1.87699073923197e-08	[illegible]
CDK14	-0.0210268851091666	0.793140902637407	0.0409502577360874	0.609469328872143	[illegible]
CDK15	0.411251170055659	7.97366377369713e-08	0.178182833263788	0.0250977392739606	[illegible]
CDK16	0.241232801554119	0.00226266790089191	0.0639057683136555	0.425032672360026	[illegible]
CDK17	0.162973463632076	0.0407585319778765	0.30230255051281	0.000113186946655947	[illegible]
CDK18	-0.137367981401464	0.0852241141404148	-0.125846094634851	0.115124725281824	[illegible]
CDK19	0.289559724256627	0.000224092577465029	0.418456595602064	4.46661115030345e-08	[illegible]
CDK2	0.732778228076202	0	0.466009034644092	6.81061031379303e-10	[illegible]
CDK20	-0.132248721074711	0.0976358142436692	-0.253616750689883	0.00130291581860458	[illegible]
CDK2AP1	-0.108053038307564	0.176573382735478	0.0374559696117219	0.640328299919886	[illegible]
CDK2AP2	-0.186782215149129	0.0187788259871137	-0.33847436368445	1.36219637039128e-05	[illegible]
CDK4	0.447526749293373	3.73810804532582e-09	0.463129327452354	8.93962178166172e-10	[illegible]
CDK5	0.442327160760167	5.92734750171076e-09	0.347262566773026	7.81174237850972e-06	[illegible]
CDK5R1	0.350266442783772	6.43461253702071e-06	0.224725168990806	0.00452943142672366	[illegible]
CDK5R2	0.145782485964688	0.0675970078633588	0.257895805713395	0.00106985907416141	[illegible]
CDK5RAP1	0.428398303388401	1.96289859917106e-08	0.286124369159939	0.000267948872104973	[illegible]
CDK5RAP2	0.336315493242534	1.55759223205987e-05	0.0784777256246248	0.327022169561263	[illegible]
CDK5RAP3	-0.193597046044938	0.0147990934095001	-0.349880163966065	6.59783870156328e-06	[illegible]
CDK6	0.12895384164983	0.106353243141289	0.0981640915877997	0.219795584406546	[illegible]
CDK7	0.390253688838003	4.00085353735591e-07	0.506431422123691	1.13156661858405e-11	[illegible]
CDK8	0.31836439408513	4.5701460238412e-05	0.329095919502163	2.42100333511577e-05	[illegible]
[illegible]	[illegible]	[illegible]	[illegible]	[illegible]	[illegible]

CNTN5	0.369701177274974	0.000798704158158969	0.339396925442809	0.002213977	0.0116238196026991	CNTN5	0.0223425102820191	0.780516649401196	0.0287544771992839	0.719862615526644
CNTN6	-0.0745804591826474	0.513607757114278	-0.0820473776288077	0.472237902	0.742770855528385	CNTN6	-0.18467503429414	0.0201837710269081	-0.211040094560335	0.00777433243678
CNTNAP1	0.274437799749142	0.0143824027571886	0.141504381694255	0.213059327	0.0887149013494656	CNTNAP1	0.318840022702847	4.44549273373695e-05	0.136199757209922	0.0879389499013645
CNTNAP2	0.231728436224119	0.0398907091302658	0.153679495651562	0.176308357	0.173359359520632	CNTNAP2	0.310276562405624	7.26248015634479e-05	0.29921724599295	0.000133932401258995
CNTNAP3	0.251003434654736	0.0256640344845223	0.112804284323272	0.321567424	0.129221811108677	CNTNAP3	0.174077396669649	0.0287079370056764	0.0417116700488927	0.602805057207344
CNTNAP4	-0.0828541256839496	0.46787856730969	0.0239254277957858	0.834218539	0.711330794392424	CNTNAP4	-0.0978744604756867	0.221165859310357	-0.117547730804458	0.141311610438164
CNTNAP5	0.146613610615049	0.197280133102965	0.154369572554352	0.174351073	0.459022267642215	CNTNAP5	-0.0768922372795204	0.336920101179731	-0.100552744211356	0.20872430563876
CNTROB	0.385893517923836	0.000444222868666078	0.338315481986368	0.002410521	0.00749645042361578	CNTROB	0.0722447655311001	0.367018928286349	0.0291925942997703	0.715776486963853
COASY	-0.0624304833414243	0.58466502966373	-0.0673076923076923	0.554861223	0.787973080523969	COASY	-0.231760914162684	0.00338897071424413	-0.372005534270981	1.48847136512114e-06
COBL	0.297792427311283	0.00768941458275885	-0.228310563053261	0.042997217	0.0576720673560123	COBL	0.160340707276223	0.0441685029984189	0.189575399116367	0.0170475851207881
COBLL1	-0.301096620742668	0.00700872052632454	-0.225073028237585	0.046319060	0.0541005811902809	COBLL1	-0.523129888291388	1.76907496301581e-12	-0.544374188513731	1.43426653405486e-13
COCH	0.309708014770607	0.00547768612944055	0.151430311529726	0.182799189	0.0458087426080656	COCH	0.0621804229151588	0.437665676813125	-0.160059999353738	0.0445457537926148
COG1	-0.245568960275737	0.0291542002572211	-0.135686465433301	0.232623349	0.140602724181204	COG1	-0.140460378807235	0.0783607273787288	-0.0967265198518288	0.226656506420272
COG2	-0.0966102628703147	0.397002016277295	-0.0209591041869523	0.854280471	0.658993359457183	COG2	-0.276086224300616	0.000445932461857299	-0.212611535410344	0.00731834388654514
COG3	-0.109533005689162	0.336588250558059	0.000632911392405063	0.995657846	0.607815925102272	COG3	-0.0582244596031005	0.467424703681067	-0.09475499289964	0.236309744770178
COG4	0.18005291605769	0.112321680579534	0.109298928919182	0.336907201	0.332691577330759	COG4	0.1627209910713	0.0410756562811811	0.147117592607425	0.0650948866284378
COG5	0.141721904862448	0.212810869804976	0.231913339824732	0.039946471	0.479561549388002	COG5	0.257708078926913	0.00107922397961513	0.276532516123322	0.000436123611426269
COG6	-0.15171132408873	0.18197886565233	-0.0545277507302824	0.632499118	0.439045434738562	COG6	-0.154660928058637	0.052341722326651	-0.153228413415251	0.0545901040588414
COG7	-0.233231662163946	0.038584782069954	-0.167453748782863	0.139987896	0.169758334922962	COG7	0.0929337954737229	0.245479502285218	0.218340524995176	0.00585060280044945
COIL	0.0107307723521029	0.92522066016891	-0.0885589094449854	0.436894247	0.964273289604295	COIL	0.326798500791581	2.7793597900283e-05	0.155320118322087	0.051333164978317
COL10A1	0.160927619456625	0.156538427920001	0.103044309720761	0.366160755	0.406301447165588	COL10A1	-0.0467710911080766	0.559519852704652	0.040649052229503	0.612082399078112
COL11A1	0.208313964803067	0.06543274845873	0.141242425050761	0.214378479	0.237624082588928	COL11A1	0.413260135209665	6.79327758490444e-08	0.24092028930948	0.00229360001879972
COL11A2	0.194445171533695	0.0859517175427378	0.326949409386589	0.003272038	0.281402593868609	COL11A2	0.15172760658371	0.0570717974191364	0.17952608812396	0.0240043594773847
COL12A1	0.132494809516566	0.244420296139537	0.000170399221032132	0.998893171	0.517352805762817	COL12A1	-0.235178395600959	0.00293457733864718	-0.26980102942314	0.000607557619052954
COL13A1	0.235102100310658	0.0370097617254768	0.334331447274895	0.002600424	0.164971617625982	COL13A1	0.248738466829366	0.00162461971678241	0.129296266801182	0.105419760612092
COL14A1	-0.261126655419397	0.0201021328494917	-0.278919182083739	0.013041171	0.11074977857754	COL14A1	-0.166742623245591	0.0362633473896724	-0.26246638021795	0.000856276142786853
COL15A1	0.116355483617177	0.30716936996532	0.174172346640701	0.124603384	0.579804905420509	COL15A1	0.0496346361783308	0.535701854766334	-0.0722969277799158	0.366672158070962
COL16A1	0.160709974103857	0.15710699486303	0.0798928919182084	0.483199073	0.407189378115713	COL16A1	-0.309269971272512	7.68629375632292e-05	-0.24033327349785	0.0023527411570152
COL18A1	0.343130954404445	0.00196301952536082	0.227580331061344	0.043891718	0.0221096811916778	COL18A1	-0.328709223218299	2.47810459638742e-05	-0.317484295099341	4.80950504249477e-05
COL19A1	-0.168444527097021	0.137827563008855	-0.0375740978789962	0.742337964	0.378446972260049	COL19A1	0.217932814033362	0.00594562615437488	0.215253062816328	0.00660519551131673
COL1A1	0.317683057316284	0.00433188796552875	0.331986368062317	0.002930691	0.0384197668978599	COL1A1	0.0588204034411	0.462871841192273	0.224769284948841	0.00452131964585265
COL1A2	0.226710348093955	0.0445191048702434	0.219206426484908	0.052435707	0.18627011976384	COL1A2	-0.242104548437204	0.00217837339873241	-0.00333151545161527	0.966862136327748
COL20A1	0.0764234923447538	0.503227673141895	0.407038328237969	0.000197076	0.736483743401464	COL20A1	-0.0358194461838452	0.655010947170364	0.0957727024143115	0.231291347284539
COL21A1	-0.0584016282558573	0.609177463126557	0.106049817734545	0.352270729	0.801564224982838	COL21A1	0.050219873040145	0.530897060543679	0.0950866232048693	0.234666145211943
COL22A1	0.0900147116230749	0.430159228227114	0.159815915389497	0.159458699	0.684282028068447	COL22A1	0.14144717702247	0.0762668396792519	0.244259410960756	0.00198210123140941
COL23A1	0.0513327480012839	0.653230051503505	-0.000243427458617332	0.998382328	0.824836431077875	COL23A1	-0.0825520131088156	0.302454274470029	0.0530046435919725	0.508334730097626
COL24A1	-0.006871548349539	0.952073717013789	0.0409994274602773	0.719779603	0.977474877606166	COL24A1	0.0241082734488991	0.763663252169474	-0.0164324587458166	0.83762951964969
COL25A1	-0.116989309097421	0.30452373772298	-0.114181376378523	0.316357270	0.577164991613781	COL25A1	0.353212147925736	5.30989612723687e-06	-0.0430877309345443	0.590885266483039
COL27A1	-0.0162653006556007	0.886863409863104	0.0810856864654333	0.476673902	0.946971975940793	COL27A1	0.146893124249024	0.0655101910754454	0.107931973192741	0.177061512778627
COL28A1	0.0148421816829468	0.896705187463924	-0.147943938317846	0.193200822	0.950944886900442	COL28A1	-0.197848858149517	0.0127077001557341	-0.106581193200661	0.182575482348191
COL2A1	0.246726993177793	0.0283788801636494	0.280514975633193	0.012278923	0.138381049377874	COL2A1	0.20477112051706	0.00985421617374715	0.176946065198873	0.0261419236730049
COL4A1	0.425753077667356	9.16359773803777e-05	0.422127555988316	0.000123241	0.0021732863711173	COL4A1	0.134968572760587	0.0908743998373274	0.172636392470825	0.0300759915744643
COL4A2	0.474302959091994	1.00665297928515e-05	0.527555988315482	8.869047081	0.000370755728571726	COL4A2	0.193188258950879	0.0150151721305631	0.190809124701417	0.0163281823653158
COL4A3	-0.261684112512138	0.0198284361856762	-0.335856864654333	0.002601798	0.109870707102953	COL4A3	-0.396456429468623	2.51318334359013e-07	-0.438327031597041	8.40643535795665e-09
COL4A3BP	-0.00903925976707797	0.936981960755156	0.127629016553067	0.261740723	0.969940675492222	COL4A3BP	-0.180570887905975	0.0231824383315973	0.0554522380101733	0.488923550431807
COL4A4	-0.186854310067194	0.0991716328182909	-0.305160662122687	0.006434461	0.307576690274448	COL4A4	-0.360157709661105	3.34980683421807e-06	-0.453798386875949	2.12154029714694e-09
COL4A5	0.0735382802632547	0.519525354583994	-0.0950827653359299	0.403764030	0.746796457806689	COL4A5	-0.124918665398173	0.117848695294455	0.0126247701976964	0.874900366507522
COL4A6	0.34326834425566	0.00195429476938691	-0.055111976630964	0.628852242	0.0220365119435201	COL4A6	0.002028534477577881	0.979818989574728	0.155960560313562	0.0503687753788799
COL5A1	0.399302857117887	0.000266990112211207	0.425365141187926	0.000108182	0.00512672858185753	COL5A1	0.108136966949378	0.176235569006163	0.252159209289802	0.00139233482669813
COL5A2	0.229307993516822	0.042070605638471	0.18356864654333	0.105308393	0.179895040630173	COL5A2	0.112500278518079	0.159323644971973	0.249734353476904	0.00155359224167206
COL5A3	0.325877057646323	0.0033815196861795	0.363753651411879	0.001056546	0.0323429906605796	COL5A3	0.102304478015497	0.200863485171639	0.255944054880326	0.00117096342878586
COL6A1	0.127261295666806	0.263718502030043	-0.0391918208373905	0.731153004	0.53706062805976	COL6A1	-0.195119112460093	0.0140182353134583	-0.136179981075761	0.0879854952862385
COL6A2	0.325579904082426	0.00341243046635409	0.178140214216164	0.116148138	0.0325258071149644	COL6A2	-0.134442399978433	0.0921524951767975	-0.177974423393071	0.0252711498418793
COL6A3	0.237284796181223	0.0352400217449764	0.272030185004869	0.015543678	0.159783849168304	COL6A3	-0.28385552116426	0.000301147533721697	-0.161692592398259	0.0423888721206738
COL6A6	-0.0397341541230742	0.728085728950764	-0.168580251358603	0.137505993	0.86617019832711	COL6A6	-0.119901722916761	0.133460862016808	-0.104195887172591	0.192618558949715

[illegible]	1.234079038	0.124599480746195	CYP4F12	-0.142377956559908	0.0743334616033824	-0.0801102598059609	0.317028037234841	0.153125101053851	CYP4F12
[illegible]	0.099526636	0.827270911334895	CYP4F22	-0.0212587799138366	0.79091172186581	0.0422919913563269	0.597765091083722	0.871114854893782	CYP4F22
[illegible]	0.589256979	0.655866330285218	CYP4F3	0.0458843659746099	0.566997146735011	-0.0893728460193593	0.264116029957808	0.705007298436734	CYP4F3
[illegible]	0.585345736	0.686221776332398	CYP4F8	-0.0218190781057431	0.785532651720322	-0.00568489524704619	0.94348435120503	0.867629956693732	CYP4F8
[illegible]	0.002350828	0.0896601637150528	CYP4V2	-0.250362681454519	0.00151024850198759	-0.1078969846743	0.177202770450824	0.00550676865153328	CYP4V2
[illegible]	0.125137111	0.635447990446321	CYP4X1	-0.310643321830685	7.11357686234845e-05	-0.311618393920607	6.73149076905417e-05	0.000368281636771189	CYP4X1
[illegible]	0.029445800	0.685312528738596	CYP4Z1	-0.105080249886027	0.18884915291923	-0.169143882894821	0.0336236573077412	0.317544070678573	CYP4Z1
[illegible]	0.016330396	0.0910517782436253	CYP7A1	-0.0772240255333425	0.33483317915398	-0.0923257131774074	0.248595527364809	0.48869300017021	CYP7A1
[illegible]	0.001853911	0.0310431124533511	CYP7B1	-0.200607535188814	0.0114936395423071	-0.0511775538005666	0.523081495720834	0.0321395848959	CYP7B1
[illegible]	0.167993104	0.382616581944474	CYP8B1	0.13255225435687	0.0968620603258801	0.0156946562563034	0.844825914179659	0.189362910757083	CYP8B1
[illegible]	0.172344468	0.0263667426370377	CYR61	-0.206613793671568	0.00919708221096133	-0.309115954232065	7.75314788481651e-05	0.0266726974643664	CYR61
[illegible]	0.670595848	0.418732738661259	CYS1	-0.16388325240939	0.0396326750423376	-0.13023344965756	0.102897819360338	0.0918245087609	CYS1
[illegible]	0.027961070	0.327165801326475	CYSLTR1	-0.268907302088201	0.000634494443393358	-0.421880906689546	3.37508790248189e-08	0.00257745867230179	CYSLTR1
[illegible]	0.003109900	0.141820801119788	CYSLTR2	-0.0383992686419088	0.631927616049017	-0.11944623823989	0.134953262681509	0.756128130213337	CYSLTR2
[illegible]	0.000719202	0.181675687803299	CYTH1	0.170579647685146	0.0321244809449992	0.158666846173025	0.0464581075691614	0.0769265991411857	CYTH1
[illegible]	0.013890785	0.433942584888295	CYTH2	0.495123955109877	3.75932618368324e-11	0.559011559141787	2.28548608322866e-14	7.33925716241871e-10	CYTH2
[illegible]	7.248367432	0.00514719415903435	CYTH3	0.42135998046864	3.52277740311199e-08	0.237456606597927	0.00266304436684287	3.82170489047951e-07	CYTH3
[illegible]	0.249490193	0.783991816093721	CYTH4	-0.227500765102315	0.00404375663914964	-0.156064004648954	0.0502144265426449	0.0130149579941862	CYTH4
[illegible]	0.195245899	0.685919270627409	CYTIP	-0.152366227184683	0.0559813840413234	-0.186417305017027	0.0190158828638323	0.121889244308719	CYTIP
[illegible]	0.162511244	0.467976249845165	CYTL1	-0.244115127155988	0.00199472302632876	-0.278364089001744	0.000379721022743139	0.00700951534924842	CYTL1
[illegible]	0.000124328	0.0969822881637293	CYYR1	-0.714188357893468	6.01266226149971e-26	-0.547331478942014	9.96755865657143e-14	3.71453396759093e-24	CYYR1
[illegible]	0.287843877	0.900862025739846	D2HGDH	-0.324677320789893	3.15405406857713e-05	-0.195396257586765	0.0138799964307998	0.000178214397239701	D2HGDH
[illegible]	0.564653782	0.761666085612728	DAAM1	0.142186933744161	0.0747269777436099	0.192047414006264	0.0156328272312504	0.153763681710055	DAAM1
[illegible]	0.946995824	0.72663538453445	DAAM2	-0.365981022651157	2.25747781311598e-06	-0.423965005396721	2.84154782960182e-08	1.66174196479269e-05	DAAM2
[illegible]	0.718212446	0.62385378711345	DAB1	0.0078987795953435	0.921535837058549	0.114563058057395	0.151765112586913	0.953277224265207	DAB1
[illegible]	0.980164233	0.549387985467213	DAB2	-0.167488816121778	0.0354249424753661	-0.172821983742559	0.0298967400442318	0.0834194411577756	DAB2
[illegible]	0.147775508	0.275221725908898	DAB2IP	0.0300591032456476	0.707718730222089	0.0531171347141782	0.507433903901911	0.810469135567844	DAB2IP
[illegible]	0.941950619	0.740510005353529	DACH1	0.0128348130435348	0.872836437596837	-0.0105695750492342	0.895137548107958	0.922546722709712	DACH1
[illegible]	0.148666876	0.514525123340812	DACH2	0.535231943069817	4.32098801184111e-13	0.0117067286117116	0.883930967842938	1.21635151831796e-11	DACH2
[illegible]	0.204613730	0.619406435681698	DACT1	0.0387243605725788	0.629043261811826	-0.15174368324138	0.0570040381894285	0.75346040663423	DACT1
[illegible]	0.738962712	0.75281083561852	DACT2	0.0275660831842927	0.730985933476807	0.113598678345742	0.155264471105488	0.828150832942032	DACT2
[illegible]	0.184126571	0.899509999185547	DACT3	0.0219401372091978	0.784371761010686	0.0678503434579653	0.396955958139896	0.866659711931745	DACT3
[illegible]	0.004622120	0.0780628500306828	DAD1	0.371613161016951	1.52979323253533e-06	0.316816470769086	4.99893298435147e-05	1.17214100307799e-05	DAD1
[illegible]	0.815118119	0.826813048019301	DAG1	-0.0783717523469228	0.327677850849163	-0.231345605175934	0.00344830945468273	0.48125642075254	DAG1
[illegible]	0.111727768	0.0666474918835866	DAGLA	0.0168327937150186	0.833730306023676	0.0451428293258772	0.573286328686994	0.898651928214044	DAGLA
[illegible]	0.497340989	0.936903965598228	DAK	-0.514471396032278	4.68931960552221e-12	-0.588389744713291	4.31067076814691e-16	1.07376086987144e-10	DAK
[illegible]	0.826075414	0.735039959167709	DALRD3	-0.272900622820616	0.000522092174142674	-0.305207888846129	9.64319472247221e-05	0.00216577668453507	DALRD3
[illegible]	0.031066758	0.253929163489753	DAND5	0.264271057688622	0.000792748699479873	0.391151231370926	3.74272728634204e-07	0.00312717025014452	DAND5
[illegible]	0.915757747	0.886181266829796	DAO	0.121653944490601	0.127837387774139	0.0606366236992623	0.449148560976855	0.235618378173431	DAO
[illegible]	0.920359435	0.899327837347252	DAOA	-0.0854653897541669	0.28565223906207	-0.0469667616681 14	0.557876268383546	0.433970764245983	DAOA
[illegible]	0.620981306	0.609112825983848	DAP	-0.00894060716566506	0.911226553154804	-0.00781309423480052	0.922384331709949	0.947510920281273	DAP
[illegible]	0.003980482	0.00143799145760316	DAP3	0.18000751328805	0.0236225748055765	-0.102870807938164	0.198368463945703	0.0594033085584 8	DAP3
[illegible]	0.642573320	0.914044601566858	DAPK1	-0.2890363406396	0.000230312453639507	-0.249471178968626	0.00157208146438614	0.00104357673431044	DAPK1
[illegible]	0.009756826	0.275599440831337	DAPK2	-0.00363786879 86168	0.963816888276982	0.0246775541808689	0.758252863766359	0.979350309812299	DAPK2
[illegible]	0.038005937	0.230789391159465	DAPK3	0.482125938861096	1.41707756640 13e-10	0.404617875938437	1.34332556222938e-07	2.48224528995699e-09	DAPK3
[illegible]	0.656796206	0.550203784348907	DAPL1	-0.339422227751632	1.28393515416035e-05	-0.492586992171693	4.89214298627939e-11	7.9990680839567e-05	DAPL1
[illegible]	0.096376824	0.737383304407549	DAPP1	0.0329455218803364	0.681114964820883	-0.0642556534980718	0.422496782456161	0.791695507082077	DAPP1
[illegible]	0.426413478	0.676578777393089	DARC	-0.33394789002293	1.8021806135232e-05	-0.360786389770679	3.21127537579777e-06	0.000108283763364759	DARC
[illegible]	0.861991046	0.932669218949368	DARS	-0.128500321532907	0.107599556542949	-0.220981397517552	0.00526726520312162	0.205805455380626	DARS
[illegible]	0.720894845	0.831237401718389	DARS2	0.332783495776933	1.93535695653946e-05	0.0542504584637003	0.498404795581118	0.000115644016779914	DARS2
[illegible]	0.001168177	0.0492401122298945	DAXX	0.21275187949766	0.00727879968297618	0.148147472041554	0.0632169188072063	0.0217113904996098	DAXX
[illegible]	0.007692914	4.39678471517241e-05	DAZAP1	-0.00750843152810178	0.925401939695491	-0.190123045491975	0.0167249140343606	0.955236877476534	DAZAP1
[illegible]	0.820260428	0.80927040196489	DAZAP2	0.321733792329083	3.75329475446051e-05	0.304292102406918	0.000101444945318986	0.000207751522638659	DAZAP2

[illegible]

DPP10	0.104439307048586	0.359672589628179	0.277786378942773	0.013187797	0.627304027444573	DPP10	-0.289474467619484	0.000225095001728917	-0.24862689080623	0.0016327590762279	0.00102295704315709
DPP3	-0.0803140556809723	0.48167779959857	-0.0554040895813048	0.627032136	0.721549914448271	DPP3	0.216625078742967	0.00625977655560495	0.255292964189325	0.0012065890288992	0.0190830412963549
DPP4	0.00263434664552183	0.981617242172012	-0.0509493670886076	0.655026063	0.992046190004596	DPP4	0.296234869921601	0.000157312226044	0.159366616541857	0.0454891611832768	0.000745036375196031
DPP6	-0.130549317798117	0.251477365859207	-0.192374034708237	0.089411843	0.525207955856747	DPP6	-0.13595583633094	0.0885144218801526	-0.147720003620594	0.063909460900148	0.17639646101035
DPP7	0.108471400541458	0.341320604394681	-0.00287244401168452	0.979994004	0.612413944817019	DPP7	-0.0487755707684637	0.542793851229369	-0.0135694601956202	0.865624455865241	0.684065909168448
DPP8	0.0410462719008477	0.719472699558673	0.170934761441091	0.131846390	0.860997127524808	DPP8	-0.191169700089144	0.0161229780864664	-0.252118136450731	0.0013949335542316	0.0431492988184106
DPP9	0.341633465928789	0.00206041396752843	0.343476144109056	0.002049640	0.022971176690968	DPP9	0.39379319835247	3.0720655708194e-07	0.379861217281091	8.53768856354899e-07	2.7308897710664e-06
DPPA2	-0.0684522983735802	0.548890582124492	0.0508105052402211	0.656534606	0.7655823648278	DPPA2	0.00560194332198765	0.94430766954377	0.0627217242689881	0.433679404492386	0.96721406655073
DPPA4	-0.0525040815082535	0.645842579834065	-0.036072900343302	0.752294588	0.820767695861071	DPPA4	-0.0762275175143691	0.341125996642331	-0.0297950053129391	0.71017122092553	0.495122169519498
DPPA5	-0.0645802756864521	0.571772024141134	-0.0417582204977048	0.714813805	0.78044905096139	DPPA5	-0.0269622708804367	0.736659296293652	0.13148086735082	0.0996150410863255	0.832529014411672
DPT	0.102996254351818	0.366385529596256	-0.0849970294613478	0.456406058	0.63287100474714	DPT	-0.380821602000332	7.96879867790409e-07	-0.533336071565366	5.40831841106123e-13	6.51156607457676e-06
DPY19L1	0.0689097779041641	0.546216698595184	0.120934761441091	0.287746283	0.763494487628232	DPY19L1	0.393774001919723	3.07649553832334e-07	0.230379617818958	0.00358996654730881	2.73350786006432e-06
DPY19L2	-0.123382054727288	0.278671039882045	-0.244157740993184	0.030366464	0.552204771745024	DPY19L2	0.107505627532916	0.178788449227693	-0.0219590984219481	0.784189976293538	0.305082640894293
DPY19L2P2	0.340520814632841	0.00213556992315689	0.382740993184031	0.000546926	0.023496288455036	DPY19L2P2	0.477370470553543	2.27123653218086e-10	0.266457749398236	0.000714064345816068	3.76697878895943e-09
DPY19L2P4	-0.0766426065076125	0.502000875316289	-0.233825668368913	0.038078652	0.735964009719649	DPY19L2P4	0.109536571372293	0.17067227718968	-0.0356426516124866	0.656605188215386	0.294670976560256
DPY19L3	-0.0448120461121213	0.694949814352352	-0.00491723466407011	0.965698720	0.847893979303765	DPY19L3	-0.231298641356204	0.00345507817860529	-0.147957317050024	0.0635602855271043	0.0113646577216586
DPY19L4	-0.0764872456423689	0.502870564860951	-0.0608325219084713	0.593624551	0.736351284174932	DPY19L4	-0.00880211004457052	0.912596220669793	0.0441068641440433	0.582127098207122	0.948261996508829
DPY30	0.0462591719546036	0.685607055083486	-0.0209347614410906	0.854447949	0.842127458417661	DPY30	-0.214533799342548	0.00679297459660884	-0.222152752265381	0.00502561451068195	0.0204536235106553
DPYD	0.212345593289315	0.0602755207546415	-0.294329931419347	0.008464389	0.225910413015975	DPYD	-0.341269398624581	1.14346886943783e-05	-0.272886104005732	0.00052246523323433	7.18718398304898e-05
DPYS	-0.257379261303713	0.0220272216834576	-0.277367908329464	0.013332190	0.116797423714591	DPYS	-0.0751409075502332	0.34807267839825	-0.139479552998518	0.0804876169736548	0.502510822562091
DPYSL2	0.150979740396074	0.184120088086676	0.149415774099318	0.188375138	0.44214753547575	DPYSL2	-0.505166455144434	1.2970801485172e-11	-0.511760325606343	6.32749302797113e-12	2.75741865291012e-10
DPYSL3	0.059513919784951	0.60236532296455	-0.0666747809152873	0.558595764	0.797869101662458	DPYSL3	0.0569079912052138	0.477568776003158	0.114240555191759	0.152928667862754	0.628586628027321
DPYSL4	-0.0611287712527063	0.592535677164198	0.182814021421616	0.106766098	0.792562709449101	DPYSL4	0.629342965198517	0	0.418821693185803	4.33578507931046e-08	0
DPYSL5	0.0229033841279728	0.841205888964474	0.164703775649196	0.146915802	0.924460672109075	DPYSL5	0.313698497803722	5.97974306484339e-05	0.011430605545228	0.886650117177646	0.000314444643883938
DQX1	0.118041586610964	0.300164304733906	0.121947392900863	0.284341428	0.573201662549695	DQX1	-0.0664387967821981	0.406873300243351	0.0287834245444913	0.719592189982831	0.562859742822369
DR1	0.554126782675066	1.17192156867674e-07	0.481426484907498	9.228691285	7.67492211433396e-06	DR1	0.47103525078849	4.21067625211435e-10	0.0857919124738727	0.283808834907631	6.61985907783306e-09
DRAM1	0.115247993548343	0.311827816688785	-0.230452775073028	0.041241838	0.583892087042925	DRAM1	-0.524263340558614	1.5539379307704e-12	-0.363109323147216	2.74514810832026e-06	3.90819635320807e-11
DRAM2	0.141952921220871	0.212058489533386	-0.00219084712755599	0.984760729	0.478714963424944	DRAM2	-0.309794566839048	7.462611088063e-05	-0.398980160749539	2.0743313168626e-07	0.000384728843828731
DRAP1	0.151238987812737	0.183359232408319	0.132692307692308	0.243167004	0.440855689104271	DRAP1	0.0200435278204513	0.80261222174438	-0.0402626572867129	0.615471317441013	0.877752821807891
DRD1	-0.0377075807346376	0.741454660618832	-0.0967280757022517	0.396423839	0.873302184237688	DRD1	-0.0834248495562751	0.297353524491534	-0.00409065151512183	0.959317056331413	0.447357477131379
DRD2	-0.0538251899128223	0.637551431968093	-0.177769812406541	0.117025831	0.816610375444598	DRD2	0.0388542518624578	0.627892373180291	0.0723075764594415	0.366601391425517	0.752571522802679
DRD3	0.137351471091945	0.227401933917416	0.247544700957258	0.027841627	0.497847625527856	DRD3	-0.00799873822010858	0.920546114009536	-0.0225675943948459	0.778362454125287	0.95256539874933
DRD4	-0.0574237735638534	0.615193846563111	0.112122687439143	0.324514408	0.804770848377143	DRD4	-0.0644499809364609	0.421092140790565	-0.0321088112498828	0.688788235362879	0.576808505353743
DRD5	-0.030514213902701	0.7895034651565	0.0524111234871135	0.646427618	0.898965115109815	DRD5	0.110088927497423	0.1685129451976	0.131766860675614	0.0988741899886469	0.291896611493746
DRG1	0.22325140687727	0.0479612790030899	0.0660662122687439	0.562197978	0.195906273466153	DRG1	-0.29109537640622	0.000206732486917164	-0.372220029101428	1.46633386761343e-06	0.000951486271036247
DRG2	-0.308972443089835	0.00559579460822956	-0.383106134371957	0.000539845	0.0465602127730603	DRG2	-0.390573660408534	3.90693990865828e-07	-0.283402435657337	0.000308218250360802	3.41046769646273e-06
DRP2	0.0157846583480909	0.890185522188696	0.288704965920156	0.010091949	0.948399550627453	DRP2	0.0347419207130641	0.664751438721127	0.000742365086935274	0.99261385589378	0.779942259056526
DSC1	0.120779942800083	0.289011788908792	0.216374712123855	0.055456525	0.562439748208018	DSC1	0.0306018158883294	0.702688380872757	0.055499438662038	0.488553142984635	0.806962882765317
DSC2	-0.196168731938523	0.0831538740147906	-0.281061343719572	0.012338170	0.275599440831337	DSC2	-0.383867724997923	6.39399362574791e-07	-0.338328324650954	1.37464759057078e-05	5.33605723708155e-06
DSC3	0.245709008400735	0.029059471599957	-0.102943945624816	0.366630294	0.140282701807164	DSC3	-0.0509391569562509	0.525021553200435	-0.0435151013602938	0.587205248537741	0.669388544525248
DSCAM	0.0944252540546481	0.407815271497254	0.123435452553613	0.278461455	0.667695504051925	DSCAM	0.208801491963658	0.00846742976319659	0.171249021652618	0.0314451701105791	0.0247751719549347
DSCAML1	-0.152406733477074	0.179960361444788	-0.0914292726049701	0.422918213	0.436183336844978	DSCAML1	-0.000555758562820156	0.994470458770959	0.139080882045378	0.081365288777404	0.996574023915833
DSCC1	0.498218379565412	2.978518295782e-06	0.567624148003895	8.807467925	0.000129470626052695	DSCC1	0.533884707673475	5.06927833043846e-13	0.304088256255997	0.000102593455060358	1.39918162014051e-11
DSCR10	0.0461983332862489	0.685998896588287	0.139818344525468	0.219082435	0.842294895500568	DSCR10	0.123636455212372	0.121696860009778	0.214339663973373	0.0068444652699577	0.226650398864948
DSCR3	-0.170728724060539	0.132490900263104	-0.167185978578384	0.140629606	0.370165987484627	DSCR3	0.044369913789236	0.579876306690951	0.0450089258753155	0.574425484335792	0.71467399567124
DSCR4	-0.0341830159346142	0.764886663165788	0.0148960897931054	0.896332084	0.886181266829796	DSCR4	0.107495608876926	0.178829179024551	0.131625385254306	0.099240128592204	0.30512003575922
DSCR8	-0.0293035268054818	0.797673622572414	0.201378855742922	0.075141439	0.902737888699948	DSCR8	0.255177727339412	0.00121299673915546	0.393902261855707	3.04701249236784e-07	0.00454534296094354
DSCR9	0.0304960836082094	0.78962565018989	0.142599725814551	0.209961987	0.898987802754757	DSCR9	0.109075772770976	0.172489332711141	0.174985586218954	0.0278732756181558	0.296833858217621
DSE	0.22288829531917	0.0489572604803166	0.126582278481013	0.265697852	0.19837670513823	DSE	-0.472093872724486	3.80131641971339e-10	-0.429040188147098	1.8597080931066e-08	6.05906799020983e-09
DSEL	0.0319459858964914	0.779870613932012	0.0266309639727361	0.815449647	0.893931529973284	DSEL	0.0964879875572483	0.227809395609363	0.0834264509279424	0.297831439751728	0.35494185897133
DSG1	[illegible]	[illegible]	[illegible]	[illegible]	[illegible]	DSG1	[illegible]	[illegible]	[illegible]	[illegible]	[illegible]

ESR2	-0.146042388357234	0.199050561917572	-0.0485150925024343	0.670530904	0.461580726181937
ESRP1	-0.0148469294485247	0.896672326864401	0.343590158902728	0.001933994	0.950944886900442
ESRP2	-0.106042349295232	0.35230483448934	-0.160564751703992	0.157215175	0.621369153363559
ESRRA	-0.188515266210433	0.0961509383323213	-0.169741966893866	0.134594402	0.302188663330153
ESRRB	0.112721531487271	0.322624849050621	-0.0181242435732608	0.874033575	0.595175288174907
ESRRG	0.0219039198027088	0.848050755827364	-0.0215925412074982	0.850185565	0.927958102147916
ESX1	0.113642510891231	0.318661607905383	0.131701470532682	0.247281412	0.59131111049171
ESYT1	0.0369417582530246	0.746526884362755	0.131085686465433	0.248958977	0.875730054503355
ESYT2	0.236583687043268	0.0358005984794281	0.313729308666018	0.005041428	0.161106766035524
ESYT3	0.0691113256738949	0.545040700740851	0.107337860426608	0.346419479	0.763064697335071
ETAA1	-0.25144065966665	0.0253993305616635	-0.275973709834469	0.014064617	0.128084640450938
ETF1	0.0715764455754597	0.530757664815746	0.143500486854917	0.206624662	0.754226547975702
ETFA	0.0215392679392524	0.850550915011749	0.0824001947419669	0.469538332	0.929197746194342
ETFB	-0.281072095254469	0.012100191245266	-0.318743914313535	0.004357025	0.0788266081847401
ETFDH	-0.324503806902403	0.00352649004868855	-0.311027263875365	0.005448539	0.0330710859094178
ETHE1	-0.0197391161594096	0.862914412142267	-0.0444498539435248	0.696732104	0.936085124957606
ETNK1	-0.145122944912803	0.201924115309278	-0.131329113924051	0.248075351	0.46497323932785
ETNK2	-0.200028479625344	0.0771506650795506	-0.290141187925998	0.009712467	0.26390277653811
ETS1	0.15292194867821	0.178475444499941	-0.0993184031158715	0.383071202	0.434037802530598
ETS2	0.101966102343942	0.371224238316983	-0.243330087633885	0.030946238	0.6373486022046
ETV1	-0.0290467090391183	0.799409551808862	0.0722492697176242	0.526123741	0.903831367728558
ETV2	-0.0471086969920112	0.680144213705733	-0.0316699123661149	0.781319576	0.839128855721344
ETV3	0.0960452105542907	0.399781983808072	0.25856864654333	0.021652294	0.660227795236811
ETV4	0.227486435371494	0.0437755321275617	0.389191820837391	0.000433533	0.184211164769982
ETV5	0.0637402528746027	0.576794123662732	0.206523855890945	0.067943664	0.78307483373157
ETV6	0.0360115301927134	0.752702496891673	0.176533592989289	0.119516590	0.879467052665282
ETV7	0.317614363522898	0.00434076933917882	0.00988315481986368	0.931035734	0.0384812801390761
EVC2	0.376097025175368	0.000635785576205139	-0.00258039387598062	0.981993667	0.00971009044493069
EVI2A	-0.0660310783515411	0.563146688048705	-0.223588120740019	0.047808375	0.775353335180534
EVI2B	-0.087634209150231	0.442502714911165	-0.217989289191821	0.053784601	0.692953614835456
EVI5	-0.207561149367658	0.0664341591732382	-0.183154819863681	0.106105841	0.239550556085379
EVI5L	0.0765940687734799	0.502272499290022	0.169328140214216	0.135557916	0.736149442576584
EVL	0.100417239359225	0.378572131652378	0.134444985394352	0.236955657	0.643224977483528
EVPL	0.0863948580185947	0.449006847890368	-0.120788801909892	0.288976158	0.697571219249012
EVPLL	-0.0243343832391424	0.831426214876197	0.0115424220375572	0.919582684	0.920091041265992
EVX1	0.00806273284088793	0.943778181563321	0.0138818073695675	0.903355699	0.972696449971827
EWSR1	0.317847839045402	0.00431064924905611	0.152799415774099	0.178478033	0.0382657185133895
EXD1	-0.107656009494718	0.344983609042052	-0.0544240629186526	0.633807480	0.614581680745244
EXD2	-0.15017812205041	0.186487222459716	-0.138120740019474	0.224290045	0.445365002489054
EXD3	0.0582197351408278	0.610294641535648	0.158154819863681	0.163601055	0.801991113672318
EXO1	0.733327000588981	1.53210777398272e-14	0.838802336903603	0	3.48299167285405e-12
EXOC1	-0.14326446211054	0.207822733105473	-0.123588120740019	0.277240308	0.473375332875739
EXOC2	-0.0802311273235751	0.482131946873167	-0.0769717624148004	0.499378843	0.721702805588884
EXOC3	-0.097608009629047	0.39212127027575	-0.103213242453749	0.364619861	0.654572458095356
EXOC3L2	-0.0347763481066963	0.760926621805341	-0.187999026290166	0.097061669	0.883938940853698

FGF18	-0.0245676598418031	0.82983434719609	-0.120058955277896	0.291921251	0.919414246756163	FGF18	0.102520284119438	0.199910069644921	0.134074481428361	0.0930546493462537	0.33183160960806
FGF19	0.184273153457452	0.104012581943871	0.248123427480957	0.027466746	0.316900576321011	FGF19	0.0962629969197591	0.228900621197695	0.177280872827287	0.0258556438251958	0.366631323842837
FGF2	0.13901330040451	0.221773540683148	-0.102142161635833	0.369638585	0.490967484721 66	FGF2	0.0344859558707855	0.667073621648132	-0.11493271936093	0.150439641513578	0.781738103773771
FGF20	-0.0858402719177531	0.451934456304059	-0.166708378204999	0.141991270	0.699467015011004	FGF20	-0.0620572371991969	0.438575753385689	0.0130339936533822	0.870880034785839	0.592513364631286
FGF21	-0.00107050736714856	0.99252934198133	0.285936006977357	0.010633425	0.996813351233499	FGF21	0.12941068428759 2	0.105109287049222	0.168874494567648	0.0339114469443705	0.202243099349517
FGF22	0.00968095089174954	0.932518476721789	-0.00827847827510801	0.942276320	0.967592593931261	FGF22	0.0807035561603537	0.313445645328591	0.162973724343346	0.040758205570333	0.465816461938922
FGF23	0.214094468639465	0.0581435681605151	0.13392678039684	0.239313860	0.220764732401357	FGF23	0.0423205938392204	0.597517175619616	0.087985542124687	0.271631604068947	0.728906488344483
FGF3	-0.0545687961797682	0.632904029871636	0.0187160936555582	0.869955457	0.81371818637253	FGF3	0.0495255177898953	0.536600110024619	0.0714724157366393	0.372177108659887	0.679095897817642
FGF4	-0.0664890087168037	0.560437026131499	-0.0645497224367903	0.571954329	0.773158663705869	FGF4	0.00854232281890545	0.915166077376206	0.01312677937533 7	0.869968936066414	0.949676313877231
FGF5	0.290944810980547	0.0092873983362316	0.180697733867656	0.111019919	0.0652294617521266	FGF5	0.11228950550102 1	0.160111574687968	0.16883785636001 1	0.0339507484770052	0.280675498953103
FGF7	-0.0639266833244959	0.575677785600769	-0.143622200584226	0.206236851	0.782348465812461	FGF7	-0.03668605656219	0.64721893044736	-0.137761967024122	0.0843238133607925	0.767590060525407
FGF8	0.118585938857458	0.297925220783802	0.221007656705022	0.050309232	0.570689105729979	FGF8	0.033044047930667	0.680213534390989	0.0470946174646352	0.556803569335026	0.790975945435706
FGF9	-0.00183904678845284	0.98716636051358	0.0903602726387537	0.427595529	0.994457101728599	FGF9	-0.22333034992805	0.00479272982915348	-0.14311521034569	0.0728303827668546	0.015137100043698
FGFBP1	0.225617914716666	0.0455834446184356	-0.0443049166186252	0.698234747	0.189368855473469	FGFBP1	-0.0357547887615817	0.655593813867151	0.0431499706781128	0.590348676928483	0.773162639844327
FGFBP2	-0.0672471487564467	0.555964626539419	-0.27985341832248	0.012494134	0.770720430622 88	FGFBP2	-0.282372005001452	0.000324875830706989	-0.23222505849172	0.00332374516541269	0.00142341466912 81
FGFBP3	0.243847217444572	0.0303402346264039	0.234006815968841	0.038149198	0.14459969724981	FGFBP3	0.126116293772001	0.114340414757556	0.269337460970706	0.000621395082915219	0.215827601006305
FGFR1	0.0816229688212273	0.474540008405325	0.274318403115872	0.014669862	0.715541579299755	FGFR1	0.509175722690445	8.39883718128931e-12	0.396187164233938	2.56493225165238e-07	1.83637283263107e-10
FGFR1OP	0.321338692347717	0.0038819353462216 1	0.308325219084713	0.005884605	0.0354827828184672	FGFR1OP	0.262833542114428	0.000848735827222669	0.0189379159165107	0.813295381996799	0.00332239561538791
FGFR1OP2	0.0621167296980944	0.586557739372672	0.0517283349561831	0.650094912	0.789286907695789	FGFR1OP2	-0.0967773914320265 6	0.22642792819967 1	-0.15845691506237 5	0.0467521247653798	0.363927312623994
FGFR2	-0.101569567941682	0.373097106422895	-0.131548198636806	0.247281937	0.63849741029956	FGFR2	-0.37104385312307	1.59169512775768e-06	-0.304188658091525	0.000102026260749794	1.21329582719837e-05
FGFR3	-0.141913903390731	0.212185431425545	-0.111489776046738	0.327266354	0.478845671238525	FGFR3	0.15655702675659 9	0.0494841753144308	0.123053098119251	0.123479518207625	0.110331072730855
FGFR4	-0.0434895385947056	0.703527977149569	0.173807205452775	0.125404633	0.852857881217541	FGFR4	0.19803198574427	0.012623757433844 6	0.17733702386146	0.0258078952957345	0.0350092417850621
FGFRL1	-0.0734725414499953	0.519899779261344	-0.0294790652385589	0.796111911	0.746973801977728	FGFRL1	0.0271662399428318	0.734741214250429	-0.121032983549299	0.129809005196424	0.830871360832334
FGG	-0.15297276864679	0.178329460485556	-0.131084078854457	0.249523837	0.433942584888295	FGG	0.0710328049853159	0.375132847186628	0.108738230356831	0.173829419972 82	0.530878293235 9
FGGY	-0.164234290239793	0.148087434561484	-0.0560370009737098	0.623096252	0.394036496805735	FGGY	-0.332679107170531	1.94773916442113e-05	-0.282812194563627	0.000317660331847106	0.000116224217978444
FGL1	0.176772692765 97	0.119127285803018	0.163344081294393	0.150328203	0.345216037891881	FGL1	0.194621693038833	0.0142693664527567	0.0709471460037869	0.375710446081751	0.0388550564110 71
FGL2	-0.167317481806877	0.140519841146096	-0.26110029211295	0.020367314	0.38298836408812	FGL2	-0.606656531049079	2.96038474645662e-17	-0.575332942374837	2.64365444779557e-15	1.47299143735855e-15
FGR	0.151124049511446	0.183696278301338	-0.125267770204479	0.270724489	0.441505042197577	FGR	-0.295381246433426	0.00016467337751967 5	-0.236128563126941	0.0028184255534222	0.000776944356775299
FH	-0.0446944865241479	0.695710809989275	-0.0139483933787731	0.902752390	0.848321109739 08	FH	0.210308337259329	0.00777485615986134	-0.0991285577048427	0.215276124736557	0.0229751367414711
FHAD1	0.15459391257619	0.173718211137511	0.110961663943284	0.330285322	0.427909815141916	FHAD1	0.105668279219203	0.186372798757654	0.119681419513007	0.134181101588031	0.314464284934159
FHDC1	0.326885307483456	0.00327849256468804	0.229795520934761	0.041836083	0.0318191360557483	FHDC1	0.29297340460221	0.000187203031968508	0.255095202998133	0.00121760459121531	0.000871054080126077
FHIT	-0.413518944875273	0.000151939735913344	-0.431864654333009	8.29673704 2	0.00325928860834503	FHIT	-0.624906634235056	1.70672787807983e-18	-0.519678774731577	2.61773970218977e-12	9.18738603375721e-17
FHL1	-0.0105188659920117	0.926693246500709	-0.0854186952288218	0.453375398	0.965085577107288	FHL1	-0.251280571176217	0.00144891084217553	-0.415012824062383	5.90205703180782e-08	0.00531257689791904
FHL2	0.225732942671769	0.0454703938827894	0.203164556962025	0.072621316	0.189050546607906	FHL2	-0.237886299596223	0.00261445614801749	-0.370584696174265	1.64334975912483e-06	0.00889523889946442
FHL3	0.473467712231872	1.04867905068584e-05	0.186684518013632	0.099453595	0.000383378452208217	FHL3	-0.0507234563535496	0.526780048036688	-0.160134964509445	0.0444447436072408	0.670864740201676
FHL5	-0.169952011833345	0.134287668174052	-0.197127555988315	0.081677678	0.372893197665828	FHL5	-0.292108665137721	0.000195971678252163	-0.346777555000472	8.05872359885146e-06	0.000907404073597163
FHOD1	0.0644480306563809	0.572561301342686	0.186611489776047	0.099587826	0.780542970633833	FHOD1	-0.268073042804748	0.000660627485167 79	-0.0657981907893676	0.411421980512833	0.00266889444852732
FHOD3	-0.0378344802655494	0.740615230923117	0.106596884128 53	0.349042691	0.873145448092359	FHOD3	-0.266640361746284	0.000707829792796 12	-0.159069974755069	0.0458978308311283	0.00283470665321389
FIBCD1	0.120383632850482	0.290608673308863	-0.0696202531645 57	0.541318950	0.564060308643737	FIBCD1	0.345462963023326	8.7658535439239e-06	0.0531247409139394	0.507373023617028	5.64263509593143e-05
FIBIN	0.108550075037987	0.340968468601258	0.0583863421351947	0.609271315	0.612131375098801	FIBIN	-0.172679521021104	0.0300342548991059	-0.150720003407 58	0.0587190703891462	0.0727827606545399
FIBP	0.279394085478154	0.0126454796219686	0.245301850048685	0.029580163	0.0813870146317263	FIBP	-0.0584275886796809	0.465870092447322	-0.0430967272804842	0.590807692254291	0.618182816920513

[illegible]

GHRLOS	0.0413499327630886	0.605956220155198	-0.00367987939609924	0.963399329565619	0.734900384904107
GHSR	0.143547881582796	0.0719598049873742	0.0983375129400756	0.218978003741878	0.149102983659827
GIF	-0.083053745267684	0.299515211626967	-0.00206128167604258	0.979493272357333	0.449944924198487
GIGYF1	0.0751599419750523	0.347950230390653	0.143740439957842	0.0715750822027269	0.502373440631474
GIGYF2	-0.408607542439506	9.82935854941583e-08	-0.29855246414256	0.000138844017876267	9.68210224155941e-07
GIMAP2	0.00443178773325106	0.955927626349835	-0.0492942962586597	0.538505986372496	0.974561280379913
GIMAP4	-0.620936907956771	3.22560765355293e-18	-0.52016517623199	2.47774823403211e-12	1.71628430352339e-16
GIMAP6	-0.602165677417751	5.81051022100338e-17	-0.531524679094192	6.69376931700125e-13	2.81503929391243e-15
GIMAP7	-0.404445385556028	1.36146488851631e-07	-0.390511084929819	3.9251388940261e-07	1.30612655537182e-06
GIMAP8	-0.558741419346129	2.36621931117565e-14	-0.522215807660659	1.96339692651992e-12	8.14244813434462e-13
GIN1	0.0257080388212215	0.748489162950932	0.109970434701949	0.168974451005772	0.840658851859595
GINS1	0.79321252838625	0	0.653763509561562	1.25215904496071e-20	0
GINS2	0.71207623828572	0	0.389464804973335	4.24169925299428e-07	0
GINS3	0.585854544956988	6.66133814775094e-16	0.494904987157075	3.84609541668e-11	2.91295095724691e-14
GINS4	0.655819222840548	0	0.351925167165355	5.77620204095165e-06	0
GIP	-0.0665632962865764	0.405992741500822	0.0284654849149898	0.722562274994593	0.561960057153359
GIPC1	0.0970138056109634	0.225273457896482	0.190488287964853	0.0165126781822525	0.362619949276404
GIPC2	-0.634746167754539	3.38545290176236e-19	-0.544559779785465	1.40202942445294e-13	1.86605353058219e-17
GIPC3	-0.021181972605123	0.791649875971585	0.0220305966987636	0.783504613819504	0.871458635292806
GIPR	-0.107045456136823	0.180666287885717	-0.0427332809519236	0.593945324274717	0.307257862353446
GIT1	0.101234111646831	0.205640867510019	0.0976605611702269	0.22218172462382	0.338687454898859
GIT2	-0.0254964724102411	0.750490527750495	0.0578816581819677	0.470054718230343	0.841986437216084
GJA1	-0.556331418055797	3.22073700370133e-14	-0.368483863827835	1.90072901720664e-06	1.08795905024113e-12
GJA10	0.182944585077389	0.0214045780079557	0.0083592197858701	0.91697789115256	0.0548904138635554
GJA3	0.246856274036929	0.00176699705989614	0.187352867575357	0.0184132445332002	0.00631535932298349
GJA4	-0.364026930935104	2.57936584097017e-06	-0.28554586272187	0.000276074892157395	1.86869907210354e-05
GJA5	-0.102985141735161	0.197867490266177	0.00576245686334184	0.942714587084021	0.32923631755086
GJA8	0.0193967385394638	0.808857632921057	0.110725138147844	0.166051040421406	0.881401591615009
GJB1	0.0432300795838501	0.589658358598313	-0.0770888335664856	0.335682528128973	0.72216673641531
GJB2	0.288563571071216	0.000236068386873844	0.129063615259874	0.106053286161659	0.00106755563801215
GJB3	0.142304306969541	0.0744849841734117	0.145266243609883	0.0685852670642388	0.153368589490271
GJB4	-0.168231312413424	0.0346070083001548	-0.0686475776118977	0.391419094165251	0.0818235549653029
GJB5	0.0485520121836812	0.544647001770457	0.037237081001341	0.642284237843722	0.685603507869683
GJB6	-0.00572772850303441	0.943059243916093	0.0161205918236334	0.840669809438972	0.966383832239856
GJC1	0.625696606804746	0	0.487201428140189	8.49651771852003e-11	0
GJC2	0.0456024957276336	0.5693839071753	0.136799126199118	0.0865375729235176	0.706786982071153
GJD3	-0.138275786131768	0.0831611496340727	-0.113642707898387	0.155103394662288	0.167637168455916
GJD4	0.0740896858920121	0.35487725301933	0.0983908311757064	0.218727074599064	0.509598033134534
GK	0.235006275126984	0.00295607491166949	0.167544802417603	0.0353627036323445	0.00988772775682669
GK3P	0.239668012569354	0.00242143450102006	0.111325944158282	0.163750829321032	0.00830605723193205
GK5	-0.208526353955252	0.00855628452145601	-0.319931970150323	4.17129104461634e-05	0.0249919149896804
GKAP1	-0.192684311143117	0.015285335192335	-0.195862683756333	0.0136500349343256	0.0412372539406341
GKN1	-0.413542293588062	6.64156656609167e-08	-0.498553299724047	2.62411994767087e-11	6.8422630375908e-07
GKN2	-0.0992242348042111	0.214831426896884	-0.0505508413984465	0.528189429962434	0.350190062791893
GLA	0.158720804787808	0.0463827858179875	0.182230852723491	0.0219263566097258	0.10451739129855
GLB1L	-0.428119095928001	2.00948277951291e-08	-0.418692387791562	4.38169287033077e-08	2.28926843878915e-07
GLB1L2	0.240625825603861	0.00232309619182791	-0.0258139204102554	0.747488178651993	0.00801052648277801
GLB1L3	0.0684970524647608	0.392460934298799	0.0532266639892998	0.506557594456113	0.548069923419623
GLCCI1	0.329459348182236	2.36846984771333e-05	0.290299737510134	0.000215564407611622	0.000138248350971472
GLCE	0.272454638498779	0.000533664617527574	0.0435637479396833	0.586787027714555	0.00220731647016011
GLDC	0.127121166488816	0.111460057041821	0.190767110388848	0.0163522400000957	0.211653393516238
GLDN	-0.382001630206699	7.31922823678233e-07	-0.342714059375615	1.04387100521576e-05	6.03975759028071e-06
GLE1	0.352234582269048	5.6606738014775e-06	0.0622369681079834	0.437248290491696	3.80895484960529e-05
GLG1	0.171760933420908	0.0309339294096174	0.180131541616993	0.0235250660915828	0.0745996385159885
GLI1	0.0901871825961439	0.259771211540275	0.132179117010822	0.0978139335746574	0.403338787590155
GLI2	0.470591049311543	4.39485337011547e-10	0.392927147059139	3.27811829018085e-07	6.88589366330432e-09
GLI3	0.341292611130215	1.14179983439211e-05	0.18218977924532	0.0219567142890094	7.17914445053236e-05
GLIPR1	0.0524875363863539	0.512486372159312	-0.0711955500689708	0.374036954312438	0.658538012944296
GLIPR1L1	-0.00709247907308394	0.929523553263026	0.0503849877958672	0.529545396093456	0.958021872892522
GLIS1	0.0520171766293297	0.516277844903729	0.0302149824449643	0.70627259639942	0.661927342965159
GLIS2	-0.117220400414494	0.142430556993456	0.0323735069980934	0.686357297957918	0.256620332183355
GLIS3	-0.293234505693214	0.000184628073680912	-0.224322040408761	0.00460416326117174	0.000861161093606686
GLMN	0.366314343726107	2.20653550986327e-06	0.228517620141891	0.00387789489653743	1.62619370442685e-05
GLO1	0.394952767148014	2.81549544123294e-07	0.371258605464249	1.56806974089896e-06	2.52968624075639e-06
GLOD4	-0.240121001628704	0.00237446544226765	-0.26331446235205	0.000829613423270387	0.00816167079763768
GLOD5	-0.0190845470082645	0.811876507942437	0.0166378011389571	0.835629010211786	0.883632663979915
GLP1R	0.135279449839954	0.0901259267775321	0.211787023367067	0.00755454179680754	0.1789686454508
GLP2R	-0.00977475254535018	0.9029829359723	0.0784077485877415	0.327455039049168	0.942269608163342
GLRA1	0.0214669838210312	0.788911720703305	-0.00196239951259529	0.980476805415277	0.869385162267534
GLRA2	0.0671687278698253	0.401726744973336	0.0628896757092023	0.432446808869991	0.557607616983721
GLRA3	0.0165058287944814	0.836914605753703	0.00665086098377257	0.933901531461926	0.899952482068176
GLRB	-0.154484021581868	0.0526151637132723	-0.212206885588367	0.00743343123131725	0.116028776049316
GLRX	-0.474664239956703	2.96110115056494e-10	-0.396961777729639	2.41874669301342e-07	4.81146268154462e-09
GLRX2	0.110218924391353	0.168007711674774	-0.00268498848041139	0.973290334235101	0.291299865505047
GLRX3	0.349498843842682	6.76281146327895e-06	0.168421036618576	0.0344005894951721	4.46088710279346e-05
GLRX5	0.1790243706517	0.0244078634267808	-0.000100401835528131	0.999001040409019	0.0611358864880319
GLS	-0.425326902750295	2.53774027851877e-08	-0.353344484022139	5.26402537371806e-06	2.8332200441195e-07
GLS2	0.0132645269412504	0.86861665524359	0.0463673931348097	0.562918149371508	0.920318124309562
GLT1D1	0.114229791650787	0.152967617123805	0.0737533714783419	0.357071662896675	0.270990553430451
GLT8D1	0.169233716948828	0.033528148018116	0.0806667898871245	0.313666874415125	0.0797072837052577
GLT8D2	-0.259821395959828	0.000978007212552295	-0.269568279713507	0.000614469275888487	0.00377782475515899
GLTP	0.0454296815946506	0.57084957040243	0.117564464443713	0.141254588419558	0.707933025274254
GLTPD1	-0.0700068666766972	0.382086451461939	-0.00528630876454934	0.947440951689696	0.537824877392331
GLTPD2	0.0362988951156251	0.650695376535528	0.0443806537832985	0.57978449576476	0.770125482611319
GLTSCR1	0.224109496014098	0.00464400823893829	0.295122118188833	0.00016697067002288	0.0147161513561386
GLTSCR2	-0.213514925972636	0.00706710370594543	-0.164175255967682	0.0392770276476754	0.021168789604877
GLUD1	-0.424388542410954	2.74350757871376e-08	-0.393369827879422	3.17119739866564e-07	3.03351198343065e-07
GLUL	-0.155053687568502	0.0517388455082868	-0.422939689682388	3.09302341965613e-08	0.114443367272325
GLYAT	0.105499861467254	0.187079611310292	0.0789051940455855	0.324385896682191	0.315345842524832
GLYATL2	0.12769545354242	0.109839574892841	0.146265920750927	0.0666821030533779	0.209266953718017
GLYCTK	-0.0522308753120026	0.51455348044656	-0.0730727601453604	0.361538397186652	0.66013446515827
GLYR1	-0.00825271314951331	0.918031976794392	0.0428852749299023	0.592632241925656	0.951415380675234
GM2A	0.069359096039733	0.386516957549826	0.121779820295962	0.127440544875312	0.541906723668593
GMCL1	-0.00291321429825664	0.971020944949664	-0.0939198321768378	0.240484567999952	0.983521984843932
GMDS	-0.136306410918218	0.0876882671660008	-0.163180365051994	0.0405001828831541	0.175053242086974
GMEB1	0.353228123224137	5.30433867274027e-06	0.0394594426024878	0.622541984679237	3.58754133862411e-05
GMEB2	-0.0451674834823418	0.573076704313271	0.0585905559903936	0.464624911118641	0.709467032261419

H2AFZ	0.513623050891132	1.29294152007002e-06	0.568743914313535	8.217339261	6.37700682891482e-05	H2AFZ	0.716885939728979	0	0.598912161324626	9.40879753307272e-17	[illegible]
H2BFM	-0.0217197997584896	0.849312950655653	-0.0484893570167299	0.671301044	0.928415815719547	H2BFM	0.0566545106554184	0.479535576543647	0.110877093701566	0.165467011066041	[illegible]
H2BFXP	0.147554183318002	0.194389621748086	0.0525213294945568	0.645734051	0.455151193283973	H2BFXP	0.439884156851589	7.3412977918963e-09	0.16281224988391	0.040960821642211	[illegible]
H6PD	-0.0375458683447194	0.742524811578527	-0.0135832521908471	0.905288377	0.87364205672795	H6PD	-0.138574322437664	0.0824915549024096	-0.200741300219041	0.0114374545984979	[illegible]
HAAO	-0.0631608446228507	0.580269967638189	-0.276484907497566	0.013882142	0.785367783476911	HAAO	-0.0797108279569783	0.31945477240859	-0.106383350941716	0.183393557721649	[illegible]
HABP2	0.0575704043109275	0.614290049404769	-0.0199165885388161	0.861694012	0.804171869018237	HABP2	0.21489048440556	0.00669926861613845	0.17005497765298	0.0326656102720518	[illegible]
HABP4	-0.062041245008288	0.58701351371403	-0.0388851022395326	0.766441751	0.789607320496099	HABP4	0.251335974274734	0.00144528234754993	0.0468876571916373	0.558540446264049	[illegible]
HACE1	-0.0158623789757934	0.889648201655426	-0.0730038948393379	0.521802177	0.948293723288987	HACE1	-0.120664269720361	0.13099072428534	-0.0896041426437345	0.262876952787013	[illegible]
HACL1	0.0419346706738175	0.713660740971932	0.0787487828627069	0.489502531	0.858245577055906	HACL1	-0.145532149409208	0.0680747763923779	-0.269184927250581	0.000626011312088879	[illegible]
HADH	-0.25760803584285	0.0219053487071972	-0.244279454722493	0.030281983	0.116588801596057	HADH	0.0702442138901148	0.380470838871914	-0.0489732771387444	0.541157594805296	[illegible]
HADHA	0.116591550985907	0.306182269650497	0.180842259006816	0.110649319	0.57873244227573	HADHA	-0.341517078776222	1.12577844690499e-05	-0.435809379509176	1.04501342618176e-08	[illegible]
HADHB	-0.0557097126495573	0.625801102090964	-0.023904576436222	0.834065501	0.809489483136369	HADHB	-0.513252924762389	5.36703312886518e-12	-0.441031796196571	6.6407494304722e-09	[illegible]
HAGH	-0.422533991573318	0.000104847536324441	-0.394547224926972	0.000356262	0.00242011624998893	HAGH	-0.493302640636417	4.5428274971458e-11	-0.336076889551233	1.58074241718009e-05	[illegible]
HAGHL	0.0281738317663525	0.805316882313445	0.0971275559883155	0.393693282	0.906600347752025	HAGHL	0.174878748904333	0.0279703753601952	0.248258939573261	0.00165986486156129	[illegible]
HAL	0.220811595759208	0.0505188092966398	0.115561592606901	0.310504112	0.202505271639429	HAL	0.225182697942488	0.00444593471847954	0.203096179634155	0.0104871256877015	[illegible]
HAMP	-0.116889878944809	0.304937782428035	-0.156420044666895	0.168629178	0.577440826524361	HAMP	0.107388801340253	0.17926381880523	0.206265079302298	0.00931838467965556	[illegible]
HAND1	0.0868384565989731	0.446672746878427	0.0523035696071732	0.647104784	0.695997288880606	HAND1	0.0490740427085741	0.540324579119099	0.183643960217491	0.0209037177005088	[illegible]
HAND2	0.290167168794027	0.00948606363252669	0.272819873752898	0.014992372	0.0661849728320927	HAND2	-0.109370559171701	0.171325265351976	-0.0689776871793706	0.389140112892676	[illegible]
HAO1	0.0790907193447818	0.488400342646624	0.13330686798587	0.241515351	0.726005162968476	HAO1	0.156178589703976	0.0500439131761925	0.031748101074315	0.692412595754969	[illegible]
HAO2	-0.0849365343018024	0.456727791355871	0.319795476951312	0.004066540	0.702969825481433	HAO2	0.124706631832041	0.118478441282081	0.0887978173249709	0.267213774708818	[illegible]
HAP1	-0.0336592479206973	0.76838743522532	-0.0805749791578891	0.480250372	0.887631227081 58	HAP1	0.131435532552285	0.0997328801974127	0.146032948535735	0.0671217364843483	[illegible]
HAPLN1	0.0842311993941119	0.460488193591152	0.312151407881279	0.005101034	0.705813897735884	HAPLN1	0.40284984591881	1.54075537217579e-07	0.425761589756702	2.44752606405202e-08	[illegible]
HAPLN2	0.0491753053300858	0.666923966484158	-0.0160716904094268	0.888201377	0.832045355738867	HAPLN2	0.0940532039436 13	0.239814435276069	0.142432281952908	0.0742218554982503	[illegible]
HAPLN3	0.232586671489513	0.0391406878079354	0.0704722492697176	0.536371019	0.171139671339612	HAPLN3	0.123528285047264	0.122025899149828	0.134170319544093	0.0928189762387626	[illegible]
HAPLN4	-0.0420035574359909	0.713210754407466	0.215205964658226	0.056820822	0.858114133197754	HAPLN4	0.0362168148626189	0.651433372431129	0.108747440511867	0.173792752533425	[illegible]
HAR1A	-0.0355584382579139	0.755716146750866	0.033982473229 8	0.769704544	0.881282662172089	HAR1A	0.35609516303366	4.39149403064221e-06	0.415677033695889	5.59456615522552e-08	[illegible]
HARBI1	-0.143877151154254	0.205864743342258	0.0218597857838364	0.848088387	0.470354081525159	HARBI1	0.0146734607528765	0.854807548255708	-0.00402369798384455	0.959982374518543	[illegible]
HARS	-0.177025948375898	0.118590816564216	-0.214849074975657	0.057396941	0.344064716151836	HARS	0.0759793637549811	0.342704642413565	0.172645519910418	0.0300671545862243	[illegible]
HARS2	-0.364254401326355	0.000966453987567876	-0.332229795520935	0.002908955	0.0134609344831813	HARS2	-0.0475997886895271	0.552574990091438	0.034498705423833	0.666957879220814	[illegible]
HAS1	0.371826719982794	0.000740770492857479	0.318839131035275	0.004184823	0.0108908712223673	HAS1	0.0277347208867151	0.729404014205794	0.0945747979031213	0.237206185569229	[illegible]
HAS2	0.177163541556124	0.118300133665477	0.198125381869599	0.080065850	0.343422874451167	HAS2	0.0925451201269693	0.247468065753656	-0.00820100447472963	0.918543784003734	[illegible]
HAS3	0.411536633446705	0.000164614777897309	0.0497322297955209	0.662760685	0.00348955099384028	HAS3	0.0519506955039151	0.516814894862454	0.046487571089457	0.561905470806866	[illegible]
HAT1	0.470151998063982	1.23225231214441e-05	0.487609542356378	6.860758085	0.000439064556927087	HAT1	0.165037710686758	0.038241973723864	-0.0198993395536892	0.80400344074563	[illegible]
HAUS1	0.553096585929732	1.25086872751638e-07	0.414508276533593	0.000166674	8.05735573186286e-06	HAUS1	0.332400972154267	1.98109630291654e-05	0.010835792037377	0.892512053145435	[illegible]
HAUS2	0.417803924239306	0.000127558744991152	0.381085686465433	0.000580111	0.00284105502076014	HAUS2	0.380033826466428	8.43266636740836e-07	0.194612244532028	0.0142741846758678	[illegible]
HAUS5	0.202102534087171	0.0740708248032347	0.181669912366115	0.109006154	0.257148108295309	HAUS5	0.51272787842794	5.68745051054975e-12	0.324422670430308	3.20210419610181e-05	[illegible]
HAUS6	0.192599248277536	0.0890303459888626	0.231986368062317	0.039882605	0.287437509462573	HAUS6	0.44725357436845	3.83048881502646e-09	0.221585329770653	0.00514140577639843	[illegible]
HAUS8	0.541342804554042	2.58721468471279e-07	0.566236611489776	9.593416425	1.52746065773879e-05	HAUS8	0.510268926126701	7.45292716430868e-12	0.207074222056974	0.00903905835846292	[illegible]
HAVCR1	-0.0892362840152307	0.434173841039815	0.130600578342115	0.251289654	0.686637632183389	HAVCR1	-0.0677518385041697	0.397643317163358	-0.0700338554538444	0.381902530300791	[illegible]
HAVCR2	-0.00511581143018448	0.964309890105385	-0.104333008763389	0.359418104	0.983656764480082	HAVCR2	-0.168684565458132	0.0341156009549691	-0.216156024452473	0.00637601015943832	[illegible]
HAX1	-0.00511882816375876	0.964288857848099	-0.00747322297955209	0.947845408	0.983656764480082	HAX1	0.248467471683517	0.00164445303494865	0.111786795181048	0.16200262191588	[illegible]
HBB	0.192645396096295	0.0889523334720543	0.162877312560857	0.151264079	0.287298135356992	HBB	-0.476726507602439	2.41971388772922e-10	-0.336496751772532	1.54022020820029e-05	[illegible]
HBBP1	0.0188658867143587	0.868921847800837	0.117792130284855	0.301194058	0.939481880723849	HBBP1	0.103601667886797	0.195181865739393	0.0875779161263341	0.273867177172309	[illegible]
HBD	0.0797848547893786	0.484579819578653	0.105694903032057	0.353893731	0.723295439503447	HBD	-0.232726738637742	0.00325451996961264	-0.152028733289819	0.0565338915772862	[illegible]
HBE1	-0.0317564886653156	0.781143670323055	-0.112019195244659	0.325668295	0.894274826784571	HBE1	0.00133305144959087	0.986737253027727	-0.0196422500051399	0.806485525676363	[illegible]
HBEGF	0.243362883300049	0.0306811039201937	0.0353943524829601	0.756353233	0.145797903251704	HBEGF	-0.106070892567358	0.184691042380967	-0.0741361068579992	0.354575025550484	[illegible]
HBM	0.0707761385970413	0.535374194439871	-0.0581482091242076	0.610734194	0.757332917559377	HBM	-0.0711964909374927	0.374030624399856	-0.0346432233273194	0.665646472491444	[illegible]
HBQ1	0.156037651069278	0.16968564027343	0.425327718520959	9.329174377	0.422728629969505	HBQ1	-0.0249927266582554	0.755262511361915	0.00507181393410287	0.949570689308889	[illegible]
HBS1L	0.000550791140001219	0.996156200593455	-0.0834469328140214	0.463898180	0.998220547313688	HBS1L	0.181659240064335	0.0223521059434089	0.0686170483838165	0.391630263707355	[illegible]
HBZ	-0.0549868213041742	0.630297659587824	0.039713557927249	0.728221198	0.812393733298791	HBZ	0.0732666549003347	0.360262379906186	-0.0292671350564503	0.715082075725161	[illegible]
HCCS	-0.195479855300666	0.0842633175395116	-0.148271665043817	0.191810228	0.278084414199309	HCCS	0.364848204986701	2.43909801689313e-06	0.360696636614676	3.23071425418826e-06	[illegible]
HCFC1	0.0102091823923214	0.928845750997193	0.136295034079844	0.230520015	0.965761290254573	HCFC1	0.193126566306498	0.0150480198117657	0.139213229919483	0.0810730767697474	[illegible]
HCFC1R1	-0.257981974649347	0.0217073820729855	-0.295910418695229	0.008311358	0.116066126693568	HCFC1R1	-0.194053217903713	0.0145611762744937	-0.0162179389176577	0.839720549174816	[illegible]
HCFC2	-0.267823200469865	0.0170192371927464	-0.147419668938656	0.194397597	0.0991185138981562	HCFC2	-0.112408365830978	0.15966688288488	0.0499240520963972	0.533323041336915	[illegible]
HCG11	-0.0877217771979707	0.442045163867482	-0.265287244401169	0.018385807	0.692519152965296	HCG11	-0.244642611311527	0.00194893077365048	-0.261234927364672	0.000915246948248152	[illegible]

IDI2	0.205658906283367	0.0690197773922227	0.294060370009737	0.008739910	0.24624525218808	IDI2	0.0389777799448217
IDO1	0.279510704217714	0.0126069052146949	-0.0696689386562804	0.541035607	0.0812445002724782	IDO1	0.428170817421318
IDO2	-0.147510297766012	0.194521806817446	-0.11155414517141	0.327693542	0.455354698927145	IDO2	0.1017110079365
ID5	-0.275337178945437	0.0140527009614951	-0.180355404089581	0.111624848	0.0874557330448238	ID5	0.167811702260714
IDUA	-0.10523856248835	0.355987342125773	-0.250632911392405	0.026139891	0.623888616932301	IDUA	-0.0845389923915177
IER2	0.348610497476386	0.0016409553450023	0.256523855890945	0.022740305	0.0194223906723252	IER2	-0.212698639425779
IER3	0.374407577005303	0.000675581072543174	-0.0786757546251217	0.489906355	0.0101919469509984	IER3	-0.419442483148789
IER3IP1	0.247515457514823	0.0278606852426961	0.264824732229796	0.018596201	0.136765607527933	IER3IP1	0.248369569635134
IER5	0.296665985600144	0.00793439786746797	-0.0843476144109055	0.459074933	0.0588182658517271	IER5	-0.0367091648771525
IER5L	0.0817205412869912	0.474010215215052	0.0163583252190847	0.886040565	0.715299256691368	IER5L	0.254433675076693
IFFO1	-0.105710943706309	0.353820278563655	-0.0882424537487829	0.438539572	0.622560290121047	IFFO1	0.116433693161915
IFFO2	0.155241215684273	0.171901604551593	0.398904576436222	0.000302970	0.425515636398174	IFFO2	0.0132062228485532
IFI16	0.210883251961368	0.0621065613474427	0.00255598831548199	0.982207049	0.230285633732606	IFI16	-0.0991578717162423
IFI27	0.23173845046979	0.0398818891325798	0.251923076923077	0.025360494	0.173359121596519	IFI27	-0.003715910855217
IFI27L1	0.137273411870846	0.227668742205354	0.219084712755599	0.052569330	0.498100927360342	IFI27L1	-0.258074260976784
IFI27L2	0.0590177378452773	0.605399994547391	0.116553067185979	0.305667832	0.799364834950301	IFI27L2	-0.0676896693895008
IFI30	-0.190437096404255	0.0927464111492304	-0.244011684518014	0.030468104	0.29528952345613	IFI30	-0.143423792006164
IFI35	0.0604434170496694	0.59669863948056	-0.221129503407984	0.050361320	0.794981858649142	IFI35	-0.462521769521574
IFI44	0.235277302426283	0.0368650205793173	0.178067185978578	0.116299638	0.164437613220058	IFI44	0.20253466375194
IFI44L	0.00643385580490657	0.955123180909049	-0.0863924050632911	0.448228328	0.979285965371652	IFI44L	0.0560601729034139
IFI6	-0.0161554097756211	0.887622785945548	0.140798442064265	0.215368760	0.947251036552692	IFI6	0.267375743465831
IFIH1	-0.0218138631356025	0.84866807215483	-0.0653602726387536	0.566390347	0.928140309418009	IFIH1	-0.0125946728622206
IFIT1	-0.0267072104635802	0.815266878745636	-0.10253164556962	0.367808721	0.912227886519838	IFIT1	-0.455155359344643
IFIT2	-0.0458125201205185	0.688485728100574	-0.104089581304771	0.360544995	0.844299404278628	IFIT2	-0.036193416464098
IFIT3	0.0275637546042371	0.809452171806193	-0.0568159688412853	0.618266620	0.909153337693154	IFIT3	-0.185884831519535
IFIT5	-0.164376106318724	0.147732787746807	-0.265676728334956	0.018210228	0.393728483500384	IFIT5	-0.279341356366418
IFITM1	0.398240609195462	0.000278197607184172	0.00131450827653359	0.990890141	0.00527535213316256	IFITM1	-0.24093954009076
IFITM2	0.304951552264184	0.00628208475573699	-0.0528237585199611	0.643185989	0.0502008372925116	IFITM2	-0.511333574765914
IFITM3	0.41894047060461	0.000121728058902093	0.0586173320350536	0.607160412	0.00274519674910558	IFITM3	-0.593061208269932
IFLTD1	-0.115265782074895	0.311752633748088	-0.0721261033701225	0.527598506	0.583831416558061	IFLTD1	0.0895549323245839
IFNA1	0.360294618681195	0.00110783223777178	0.188683804046002	0.095848506	0.0147945347728901	IFNA1	0.143778198304906
IFNA14	0.103661955124522	0.36327929038767	0.148979949648579	0.190066338	0.630041001965412	IFNA14	0.0170629233157769
IFNA16	0.0586199026014136	0.607838011669904	0.109237991816107	0.337899173	0.80038749632539	IFNA16	0.0391342709667575
IFNA17	0.0376303787831488	0.741965492698923	0.103499567892493	0.364035514	0.873634925321693	IFNA17	0.0109716904424893
IFNA2	0.103844101777597	0.362432195707304	0.109175961724916	0.338175218	0.629700382410763	IFNA2	0.0116653114316382
IFNA21	-0.063719981181047	0.576915570033065	-0.126156858609354	0.267919180	0.783185952646988	IFNA21	0.145679232558939
IFNA4	-0.0863930585523476	0.449016330012516	-0.124134081609212	0.275729051	0.697571219249012	IFNA4	0.0555514292397507
IFNA5	0.073470202543098	0.519913103348438	0.288798839213029	0.009844663	0.746973801977728	IFNA5	0.0465437814864997
IFNA8	0.0586199026014136	0.607838011669904	0.109237991816107	0.337899173	0.80038749632539	IFNA8	0.142836717436602
IFNAR1	-0.0278162130187411	0.807740291825224	0.0965189873417722	0.396674749	0.908273080782861	IFNAR1	-0.1408267642359
IFNAR2	0.146485610846282	0.197675865920176	0.131353456669912	0.247987107	0.459805577029945	IFNAR2	0.196737531271338

[illegible]

[illegible]

[illegible]07205453	0.357844125	0.49318379894825	KCNIP4	0.166911657214073	0.0360719717422731	0.281826431087532	0.000334029551669419	0.0846969493812949	KCNIP4	0.0832016828757099	0.509946009302419	-0.020236013986014	0.8726582401934	0.583861458149299
[illegible]88519364	0.197478924	0.680712599908248	KCNJ1	0.00804807954055002	0.920057614282002	0.05963869030371	0.45666042993923	0.952443807857156	KCNJ1	-0.515063965175889	1.13350027893959e-05	-0.53986013986014	5.01157291012 86e-06	5.68714611357039e-05
[illegible]23242521	0.840076729	0.647254644414087	KCNJ10	0.0911183059487729	0.254863647021985	0.0688043702315528	0.390335644359889	0.397731604787212	KCNJ10	-0.16263218223576	0.195534834330224	-0.314291958041958	0.0110640172020258	0.268281206344075
[illegible]32954434	0.001103089	0.134793776144693	KCNJ11	-0.0454140781255152	0.570981993347651	0.105622811314383	0.186563424760158	0.707959219930647	KCNJ11	-0.149350632071205	0.252607844020499	-0.168487762237762	0.179298196216625	0.331392186898989
[illegible]28628595	0.163970000	0.199576384427504	KCNJ13	-0.00789909880932953	0.921532676224065	0.0975578589016452	0.222670659453412	0.953277224265207	KCNJ13	-0.281464611110418	0.0231263332394083	-0.288243006993007	0.0202021991831416	0.0440031118817529
[illegible]02044791	3.42675014e-05	0.0155760803818813	KCNJ14	0.277466298418493	0.000416241245155989	0.122001921326072	0.126742652713187	0.00177138762625422	KCNJ14	0.116222609183725	0.35654681864768 3	0.0809877622377622	0.520993203566795	0.438675325110515
[illegible]92014561	0.396849703	0.270215133332688	KCNJ15	-0.0686851985861029	0.391158965447626	0.0658164456685545	0.411291947816666	0.546957052551329	KCNJ15	-0.0153266993025555	0.903550633868739	-0.0899475524475525	0.475182757722127	0.924270551600653
[illegible]68540666	0.020167207	0.53031916833592	KCNJ16	-0.0173963153256393	0.828248550849708	-0.0253925369490237	0.751474341376958	0.89498213600766	KCNJ16	0.0157489514424573	0.900906640274142	-0.0765734265734266	0.5434414503415	0.922332145201535
[illegible]047711782	0.570334889	0.802967037825839	KCNJ2	0.00977174509255854	0.903012639697221	0.220893165601482	0.00528588166252 71	0.942269608163342	KCNJ2	0.473404065015833	6.81210958164424e-05	0.446372377622378	0.000224896724198195	0.000282333773300323
[illegible]56434376	0.063860974	0.506624778557417	KCNJ3	-0.0306750485375067	0.702010575267865	0.00190154991530552	0.981082061633985	0.806541106507826	KCNJ3	-0.520324607593408	8.88458805076619e-06	-0.538374125874126	5.3674008134227e-06	4.56040401430794 5e-05
[illegible]63505347	0.361415494	0.708456484922249	KCNJ4	0.19760330434737	0.012821025710872 7	0.0961667035567628	0.229368777284329	0.0354833258173732	KCNJ4	0.0863891178323778	0.493812242671583	-0.158085664335664	0.207966479108449	0.569131203795664
[illegible]43427459	0.104005171	0.624920386397275	KCNJ5	-0.265118875408397	0.000761595451016296	-0.370885901680849	1.60928756810755e-06	0.00302111016014006	KCNJ5	-0.623141192785327	2.96374902180202e-08	-0.634047202797203	3.32329288857004e-08	2.19719677800688e-07
[illegible]81768335	0.214821130	0.306347427321077	KCNJ6	0.152209156662214	0.0562379659095207	0.266334123617555	0.000718313682397017	0.122250673325573	KCNJ6	0.159405105131543	0.204680936889412	-0.185576923076923	0.138640187136336	0.278520005779012
[illegible]49561831	0.814786624	0.955733545793952	KCNJ8	-0.297979875808648	0.000143208640038814	-0.434035613748179	1.21687657644502e-08	0.000685688182864646	KCNJ8	-0.43181131104883	0.000328621671961911	-0.453321678321678	0.000175270327300473	0.00112238282298155
[illegible]33676007	0.630596009	0.533696084928562	KCNJ9	0.0975228814684756	0.222373505581 1	0.118711840531284	0.137386378564497	0.359925918913389	KCNJ9	-0.407147629298834	0.000762832473536685	-0.428103146853147	0.000423207331692171	0.00233322769243392
[illegible]42745862	0.046367273	0.194220813479491	KCNK1	0.6050925630659	0	0.460925050790531	1.09902170591327e-09	0	KCNK1	0.167257183099154	0.182955972004349	0.154589160839161	0.21829868011522 2	0.253864975832528
[illegible]29715242	0.026281983	0.703145705450606	KCNK10	0.00602831681181116	0.940076453208976	0.09421951644349	0.23898061825088	0.964221266007981	KCNK10	-0.41031865592 26	0.000686991232216222	-0.429938811188811	0.000397750714084285	0.0021295087961704 7
[illegible]18402288	0.176164516	0.480126980669353	KCNK12	0.143608235247878	0.0718390410647831	0.272816334478321	0.000524261386571527	0.148919800248019	KCNK12	0.146574637849 0064	0.243979066417875	-0.0753059440559441	0.550150242733848	0.321840586560232
[illegible]51051607	0.038561008	0.684456599491758	KCNK13	0.234951975958532	0.00296288626040586	0.209395634193579	0.000827833640119979	0.00990678097603921	KCNK13	0.06648616048423	0.598741818382 13	-0.0831730769230769	0.509169180609012	0.665194956057264
[illegible]93962999	0.001625942	0.217044112398366	KCNK15	-0.113502162621448	0.155618001432125	-0.0479525251442084	0.549631660709079	0.274731921475884	KCNK15	-0.262373939030404	0.0347357058708695	-0.281424825174825	0.0234613758868927	0.0623659481895257
[illegible]74166966	0.080228599	0.0694277699929954	KCNK17	-0.0548975875680629	0.493287377517 04	-0.056355897395331	0.481858173573365	0.641976162170544	KCNK17	-0.381287158676663	0.00172626071338283	-0.432561188811189	0.000363810083245566	0.00470935434768816
[illegible]07237997	0.190344223	0.452061313552692	KCNK2	-0.438347585548227	8.39145236506205e-09	-0.503547151212156	1.54349864 5497 7e-11	1.03128598822959e-07	KCNK2	-0.504564898929394	1.82126144518 63e-05	-0.487368881118881	4.79763153763951e-05	8.70417149618545e-05
[illegible]73904576	0.298813595	0.901106005810746	KCNK3	-0.22839484527049	0.003897590797851 5	-0.287187280608762	0.000253594540764917	0.0126121395242595	KCNK3	-0.495158546454473	2.74925000399519e-05	-0.497377622377622	3.19869339706 15e-05	0.000125860110876712
[illegible]13145083	0.672868804	0.906600347752025	KCNK5	-0.094974425074948	0.235221312729094	-0.0829517553600 93	0.300111118251964	0.374312764054164	KCNK5	-0.296804910955595	0.0163565950245726	-0.356297599426858	0.00357880428521677	0.0328123638604679
[illegible]59662025	0.364847070	0.140583575164751	KCNK6	-0.312123507870591	6.5412053919913 6e-05	-0.382867348300351	6.87514040907173e-07	0.000341405343324231	KCNK6	-0.293721457746884	0.0175608531828266	-0.35590034965035	0.00380351481361494	0.0348539784363723
[illegible]55822785	0.000312206	0.131555070275327	KCNK7	0.0680846640182832	0.395323744951371	0.064442225097378	0.421148149656957	0.551064597906772	KCNK7	-0.245550435275998	0.0486578899737164	-0.267744755244755	0.0313648120732542	0.0831672864790606
[illegible]58947586	0.063050560	0.924460672109075	KCNK9	0.0798919652707155	0.318352790896951	0.0405273530349234	0.611314889299940 8	0.471169296600 44	KCNK9	0.147140236907441	0.242144253371116	0.0628933566433566	0.617881844604608	0.319875707738709
[illegible]05647517	0.112311687	0.999504415153091	KCNMA1	0.0983977620427694	0.218694471209751	0.109497329083021	0.170826464916568	0.354790730963299	KCNMA1	0.00145226615578718	0.990839399995585	-0.00541958041958042	0.965863497034938	0.992629547583982
[illegible]779941577	0.386710229	0.781104225144546	KCNMB1	-0.111782883087872	0.162017403232785	-0.0428168191329513	0.593223471269437	0.283288098918755	KCNMB1	-0.566913688704859	8.50949774544067e-07	-0.529982517482517	7.86554012232803e-06	5.22897236612 38e-06
[illegible]55704856	0.026660508	0.685533013063333	KCNMB2	-0.0640057173168249	0.424307367321 35	-0.0633900679766247	0.428786361231244	0.579746076323739	KCNMB2	-0.30792828297792	0.0125820840871472	-0.483828671328671	5.52217995370753e-05	0.0262310799764451
[illegible]94741967	0.165639740	0.761662825997706	KCNMB3	0.238976531077964	0.00249475748521677	0.124148768590883	0.120147796088027	0.00852896663005399	KCNMB3	0.680784234283384	4.3892778300858e-10	0.666346153846154	9.91403265468298e-10	3.73871550366999e-09
[illegible]01363194	0.557589128	0.950031815853691	KCNMB4	-0.30710717917981	8.67645280172659e-05	-0.393336360600912	3.17916282841917e-07	0.000439190255924626	KCNMB4	-0.38987388469283 5	0.00132580537512002	-0.423732517482518	0.000489927483077185	0.0037666403934960 3
[illegible]20727951	0.525773622	0.846614747064581	KCNN1	0.228054580485353	0.00395264820418606	0.208301862682295	0.00862939368175502	0.012766247891218 3	KCNN1	-0.158152619869346	0.208296665818034	-0.170454545454545	0.174218981673417	0.282609887542218
[illegible]40311587	0.063450676	0.344406245988419	KCNN2	-0.529234834441559	8.74892407411498e-13	-0.460678609921508	1.12458905208312e-09	2.30096703149224e-11	KCNN2	-0.561344812537443	1.14719194957723e-06	-0.520192307692308	1.21491667984027e-05	6.90361348275421e-06
[illegible]62290166	0.531586028	0.378215559329659	KCNN3	0.11784547022495	0.140299639683115	0.146836609967729	0.0656150922273361	0.253624937801075	KCNN3	-0.236774514107481	0.0575648604661535	-0.334178321678322	0.00675411859465653	0.0958173184128299
[illegible]561830575	0.639972153	0.00910911020695769	KCNN4	0.0749001092697642	0.349624070010589	0.118145578097 83	0.139285214992965	0.504166630056993	KCNN4	-0.284709524158542	0.0215242689799475	-0.329122776659499	0.00742816827793215	0.0414485798266886
[illegible]62999026	0.001916230	0.0514513649454732	KCNQ1	-0.675720328542427	2.04720159573034e-22	-0.586206765401052	5.87097721235579 3e-16	1.23976912425643e-20	KCNQ1	-0.834410339080977	5.97012306593 9e-18	-0.734134615384615	0	5.84148923562092e-17
[illegible]01748262	0.080818220	0.801066056183473	KCNQ1DN	-0.0254357453649129	0.751065298224351	0.0432108202754026	0.589824287189596	0.842448799141552	KCNQ1DN	-0.403872333115256	0.000849057285172686	-0.386188811188811	0.00160181407698 4	0.00256110533011193
[illegible]37293087	0.120188873	0.422331911226451	KCNQ1OT1	0.139468589324411	0.0805116511870581	0.199763142942607	0.0118539384010063	0.163329972270385	KCNQ1OT1	0.360223476958106	0.00320348612616961	0.397639860139886	0.00113100276860803	0.00805829680483199
[illegible]10365741	0.055577528	0.653142177032298	KCNQ2	-0.0723009282915781	0.366645571363848	-0.0128225313888882	0.872957096483864	0.522158657755739	KCNQ2	-0.0945629175020867	0.453688982872933	-0.295192307692308	0.0172871718413334	0.531887463595906
[illegible]45458871	0.751009927	0.553543707351948	KCNQ3	-0.0198699253684384	0.804287322964155	0.0238408903519848	0.766208402 73 75	0.878741911542615	KCNQ3	-0.343613178388668 2	0.00507196629692054	-0.325393356643357	0.00843078512372682	0.0119864019833347
[illegible]01472832	0.626270174	0.912573428721559	KCNQ4	-0.118738997971246	0.137295811365237	-0.0845155269157029	0.291060233852681	0.249214068444584	KCNQ4	-0.332081433970651	0.00688128820830039	-0.404676573426573	0.000908163105349735	0.0155787521900525
[illegible]104632705	0.822174950	0.298703339003233	KCNQ5	-0.0549454855510645	0.492909717034685	0.000063680818565706	0.939725583864159	0.641667931735861	KCNQ5	-0.258423928204066	0.0376629946137211	-0.294405594405594	0.0175978812487014	0.0668153929723 31
[illegible]28889548	0.071429265	0.323096753209904	KCNS1	0.0468231269356374	0.559082537599515	0.0315459524749524	0.693968036605752	0.698047440977082	KCNS1	0.0850876939344176	0.500367245463069	-0.0201048951048951	0.873477383703268	0.574969258492914
[illegible]76793545	0.042366449	0.814083829590922	KCNS2	-0.130792333995636	0.101416641969958	-0.146791633803435	0.0656958297803 62	0.196431391758751	KCNS2	-0.435410761766723	0.000289039208581619	-0.426005244755245	0.000454120055057 85	0.00100407200701219
[illegible]08666018	0.222239983	0.200462949898879	KCNS3	-0.0570999500091704	0.476082246473935	0.0785583513410337	0.326523885133323	0.627347659980327	KCNS3	-0.0787536326669507	0.532902119038332	-0.176072728077907	0.160620500593801	0.605356733221884
[illegible]745331165	0.551029784	0.940152725834362	KCNT1	0.0544929745580159	0.496483738276611	0.138898333253508	0.0817697281805359	0.644863907776969	KCNT1	0.0665499476663045	0.598390353827 68	0.0186188811188811	0.882770116579	0.664941957268251 9
[illegible]492707574	0.537116133	0.972234810871 26	KCNT2	-0.0741791141054 74	0.354295167065442	-0.236213572959116	0.00280823902937713	0.509110263666992	KCNT2	-0.263649334803008	0.0338320314585136	-0.362281468531469	0.00319002228902063	0.0610193792211802
[illegible]216382097	0.509957825	0.948293732328898 7	KCNU1	0.0340217865072334	0.671292747230359	0.0319354384790712	0.690382201120679	0.784517201581344	KCNU1	-0.145129194914021	0.248711339304 09	-0.214665807112 98	0.0859442150702919	0.327188561106659
[illegible]89023972	0.934503153	0.653314971785256	KCNV1	-0.0588435931812754	0.462695173762235	0.0584358339932777	0.465807048575342	0.614852418012475	KCNV1	0.0881318795287444	0.485105071380768	0.0671765734265734	0.594105878669055	0.561162452084241
[illegible]64616547	0.022920689	0.331771575265292 9	KCNV2	0.0251286824354306	0.753973682189089	-0.0356959221611359	0.656124658830734	0.844476211541104	KCNV2	-0.283741774335036	0.0219918831910251	-0.316622230295008	0.0101804426513968	0.0421911874340 74
[illegible]950340799	0.486949085	0.867768019002138	KCP	0.104690484325404	0.190503752081692	0.133044601994232	0.0956173254060412	0.319735078477888	KCP	-0.458752174746082	0.000121366393472 63	-0.413636363636364	0.000682299285177695	0.000469256372758 95
[illegible]41187925998	0.997530923	0.724724242248401	KCTD1	-0.328689931897538	2.48098620241262e-05	-0.202327953468371	0.0107892064809758	0.000144358267972239	KCTD1	-0.461134278852301	0.000110685322639803	-0.441695804195804	0.000265242847251869	0.000432242642869002
[illegible]786757546	0.612252376	0.647655057215572 5	KCTD10	-0.0720656264143 23	0.368211370604653	0.0276318021292875	0.730369316567374	0.523376415444075	KCTD10	0.259812320888102	0.0366115707163255	0.292613636363636	0.0183235844970821	0.0652177122759894
[illegible]415092502	0.016949134	0.283695240979426	KCTD11	-0.0320069464978097	0.689724600813541	0.0768347864978008	0.337282301565 49	0.797517997225348	KCTD11	0.0689937000298246	0.584994815050002	-0.0364073426573427	0.772919137054523	0.652875863365365
[illegible]75073028	0.170987772	0.859612124927 59	KCTD12	-0.325102942224509	3.0752547980848 6e-05	-0.287089921253098	0.000254879017595845	0.000174147772478444	KCTD12	-0.429417764385355	0.000357619146210021	-0.329414335664336	0.00762272846345936	0.00120717962778372
[illegible]7166504382	0.934938582	0.789777592423854	KCTD13	0.285212395336724	0.000280862276763827	0.402795430499608	1.54725215612183e-07	0.00124895519691354	KCTD13	0.183031026783793	0.144463057731937	0.250305944055944	0.0445827443919 58	0.208536799867344
[illegible]94255112	0.070897393	0.0875083412807035	KCTD15	-0.0408773249770717	0.610084158190 03	0.0733461778722169	0.359739851547186	0.737856349518493	KCTD15	-0.0736512240260376	0.559848610187004	-0.192744755244755	0.123831637520787	0.629941257335235
[illegible]96541509	0.001329866	0.0340152307857642	KCTD16	-0.0364586898373526	0.649259612078173	0.0382728754553372	0.633050532964157	0.769167917628846	KCTD16	-0.340946614968566	0.00544819593433216	-0.290484724260164	0.0189057839496141	0.0127454765493827
[illegible]49756573	0.047907404	0.769873523851508	KCTD17	0.207236918814753	0.00898379594828684	0.0996031845637 03	0.213076547956451	0.0261282280263887	KCTD17	-0.36427943730662	0.00285289402582087	-0.337456293706294	0.00620829534040289	0.0072727601142555 2
[illegible]95715677	0.223222357	0.448881734132 73	KCTD18	-0.393718726075395	3.08928569865265e-07	-0.355647641279557	4.52342516422295e-06	2.74222515487923e-06	KCTD18	-0.0867709395018 15	0.491897650774482	-0.0277097902097902	0.826214663799983	0.567574212432095
[illegible]47021772	5.07411637 5	0.0124986203923138	KCTD19	0.166402832397161	0.0366506431494016	0.152776605155374	0.0553155666975524	0.0858554956585422	KCTD19	0.0705690910916405	0.576432024342671	-0.0591783216783217	0.638828331209612	0.645150451683479
[illegible]41187926	0.508397048	0.378461766606595	KCTD2	-0.307360996750082	8.55436555548158e-05	-0.163341616484812	0.0402997896103307	0.000434084536594311	KCTD2	-0.0362585018922563	0.774305577315121	-0.0600524475524476	0.633873412230038	0.817995578806825
[illegible]62609542	0.065730180	0.270215133332688	KCTD20	0.121979445763553	0.126813141790135	-0.0788717267670761	0.324591799435941	0.234263678714705	KCTD20	-0.0889726058100248	0.480933847721268	-0.151303146853147	0.222797749295802	0.557172123116631
[illegible]011684518	0.585068969	0.619569397369019	KCTD21	-0.515332045114811	4.26148435007382e-12	-0.327623608444714	2.64526742012617e-05	9.85602096543455e-11	KCTD21	-0.155774592462373	0.215305901490295	-0.110358391608392	0.380581607597039	0.290486116655903
[illegible]28529698	0.457257167	0.420430626467606	KCTD3	-0.346156878355442	8.38558175598844e-06	-0.328070853324977	2.57515376553043e-05	5.42821941376157e-05	KCTD3	-0.207071183283475	0.097912270633343	-0.167963286713287	0.180670729342273	0.150276906494883
[illegible]191173347	0.753269645	0.640385762992 78	KCTD4	0.0675748788813785	0.398879909464355	0.0558860152717438	0.485681537092554	0.554813534469321	KCTD4	-0.194000862666083	0.121504672185706	-0.204632867132867	0.101982423675 04	0.180179805085363
[illegible]96786758	0.005598365	0.00950412403466075	KCTD5	0.564049819753 76	1.19904086659517e-14	0.381210485645247	7.48884481711334e-07	4.32830242235629e-13	KCTD5	0.449929265824615	0.000169725207726401	0.532167832167832	7.12651571725887e-06	0.000630952778642608
[illegible]93865628	0.134764053	0.451400080996835	KCTD6	0.246596667379862	0.00178750224445379	0.0149446610943691	0.852154398323245	0.00637626738077781	KCTD6	0.390012723471838	0.00132008268 53319	0.288636363636364	0.0200266279555368	0.00375222324402145
[illegible]70204479	0.122223226	0.0126284090684177	KDELC1	0.566085108834037	9.10382880192628e-15	0.240101862225932	0.00237643311408118	3.36549173179644e-13	KDELC1	0.680980443076868	4.31977564829822e-10	0.74256993006993	0	3.68222318787585e-09
[illegible]38558909	0.071342401	7.46542045433589e-05	KDELC2	0.237879035519279	0.00261527083076496	0.0878820308857598	0.272198122419322	0.008896366591718 94	KDELC2	0.437685088513	0.000266328409849415	0.359309440559441	0.00346380548215256	0.000935279146643283
[illegible]27166504	0.072239182	0.282079635235234	KDELR1	0.454558723667968	1.97919214350861e-09	0.419097037613539	4.23956217584398e-08	2.74581215590908e-08	KDELR1	0.675676509509637	6.62199850509637e-10	0.667788461538461	4.82233670171835e-10	5.59115878155421e-09
[illegible]22200584	0.001296337	0.0132938880458923	KDELR2	0.459027306591749	1.31133104375181e-09	0.404864316807 46	1.31781068813248e-07	1.89049369737438e-08	KDELR2	0.604226161673884	9.85931940533646e-08	0.588592657342657	4.51279012462927e-07	6.85299632135632e-07
[illegible]94547225	0.013986160	0.0062950096301628756	KDM1	0.390354082345118	3.97157636062375e-07	0.314701947263266	5.64594 47995315e-05	3.45996257083333e-06	KDM3	0.462358107644339	0.000105540846420 56	0.363767482517483	0.00300605205155213 3	0.000414643936064 49
[illegible]39532619	0.002663186	0.00875084064685079	KDM1A	0.17676871690568 9	0.0262946629586649	-0.160232202063304	0.0443140081693801	0.0651699321 6685 79	KDM1A	0.229680454979741	0.0656944620614865	0.0971153846153846	0.440597592206388	0.106928367513109
[illegible]72346641	0.206469475	0.771533330896 52	KDM1B	-0.118928309803335	0.136665742746613	-0.128030595177554	0.108024541 54275	0.248446363579 06	KDM1B	0.170943903330909	0.173356464 6378824	0.0624562937062937	0.620330789451129	0.242814379276297
[illegible]9406037	0.192104658	0.167765986416122	KDM2A	-0.217736125366 19	0.00599195809192389	-0.279766672219273	0.00037086040723648	0.0183669577875989	KDM2A	-0.119170442137185	0.34439571 07605	-0.167438811188811	0.182050907415239	0.426489394782012
[illegible]90165531	0.661675679	0.688684435212361	KDM2B	0.353919224100246	5.06911117529 93e-06	0.341154824142525	1.151740891482 15e-05	3.44618551970923e-05	KDM2B	0.612988159670111	5.70564573365573e-08	0.668225524475525	3.51956559303574e-10	4.09237396071436e-07
[illegible]06329114	0.220528176	0.989076693807924	KDM3A	0.125800861997198	0.115256431156442	0.117297083089923	0.142167822530 42	0.217316350154094	KDM3A	0.582065992708854	3.66299222287481e-07	0.538505244755245	5.33507299730438e-06	2.35717976887415e-06
[illegible]93865628	0.090115599	0.0497344645990533	KDM3B	0.0291093256515656	0.716552481443113	0.117079188905327	0.142915349426155	0.817300776473229	KDM3B	0.332718312993039	0.0067682949748317 8	0.371547202797203	0.00245621561906061	0.015348449193880 7
[illegible]15774099	0.178478033	0.804069266249046	KDM4A	-0.0444330589186089	0.579336605802108	-0.0963142638301905	0.228651648307648	0.714296552681041	KDM4A	0.158594147758456	0.207013241584443	0.155506993006993	0.215421898394928	0.281066166906771
[illegible]09250243	0.011930915	0.358096627131137	KDM4B	0.285007095098436	0.000283847839154694	0.278724622865686	0.000390977580373539 6	0.00126162209532543	KDM4B	0.172745409134334	0.168801636936873	0.185052447552448	0.139774161316785	0.237598511696729
[illegible]442064265	0.604323043	0.849408369687015	KDM4C	-0.00807640707525899	0.919777173317505	0.0517541037348872	0.518404684182 12	0.952260587154 16	KDM4C	0.254909821176043	0.0404366951893527	0.282867132867133	0.0227369022874388	0.0709695256581 85
[illegible]400194742	0.938503401	0.917329333577554	KDM4D	0.140817796278516	0.077590703939886 27	0.052820533103727 3	0.509810872599559	0.158623295004937	KDM4D	-0.0126889802657938	0.920050095722744	-0.0383741258741259	0.761023401408133	0.937408685463 45

[illegible]

LCMT2	-0.0807578064311202	0.479251533350386	-0.13412852969815	0.238068898	0.719386522966297	LCMT2	0.264141186810816	0.000797663673574478	0.174515123787147	0.0283030251731646	0.00314402912548177
LCN1	0.276576450395141	0.0136091230532607	0.0863708997916618	0.449133102	0.0857647132383017	LCN1	0.0535026702565451	0.504352854349099	0.0491147898101187	0.539987908645591	0.651314257054357
LCN12	0.141960989834866	0.212032245553108	0.0868554458112835	0.446583488	0.478714963424944	LCN12	-0.0157527758419705	0.844258552309538	0.0261334205512297	0.744470246782302	0.904861148513628
LCN15	-0.0936459953836576	0.411712929139833	0.000256281600293229	0.998211485	0.670967230761999	LCN15	0.0320158188805512	0.689643025680751	0.0739203156888637	0.355981316712299	0.797510007963915
LCN2	-0.173507313991698	0.126212418845933	-0.132865941274549	0.243089691	0.359480014391541	LCN2	0.297019666840459	0.000150816288512212	0.358014690614129	3.8659122441766e-06	0.000718210600157546
LCN6	0.145011101350271	0.202275674169659	0.0227609456521666	0.842180677	0.465345467340093	LCN6	0.0383929894329644	0.631983382704341	0.11851828188123	0.138033198235384	0.756142104480804
LCN8	0.321692987327911	0.00384062155466469	0.18155687119051	0.109303668	0.0352973109238654	LCN8	-0.0286823656632659	0.720535932579676	-0.0796521834373355	0.319812075947083	0.820224123222693
LCOR	-0.00704299776821581	0.950879390507678	0.151144109055501	0.183271078	0.976808423905929	LCOR	0.194164898329167	0.01450344060716	0.275720383717852	0.000454124767755529	0.0394166432798665
LCORL	-0.093957547538425	0.410152030141641	-0.00903115871470302	0.936975469	0.669692632059884	LCORL	0.350173249658339	6.4736383902364e-06	0.413632743776917	6.59362507349055e-08	4.29167024718229e-05
LCP1	-0.13429075951107	0.238027748809208	-0.200973709834469	0.075809439	0.509772682402442	LCP1	-0.369189303204816	1.81030237945298e-06	-0.335480563497793	1.64002853288995e-05	1.36476932046394e-05
LCP2	-0.0133915540016337	0.906753257631665	-0.227166504381694	0.044284899	0.95573545793952	LCP2	-0.4301215485279	1.69755445172907e-08	-0.383308825727625	6.65965907896621e-07	1.96429776595425e-07
LCT	0.284602815570119	0.0110191724852209	0.390953550250325	0.000367505	0.0736741334292478	LCT	0.210004568204224	0.00808842736817139	0.250720498359032	0.00148606117471103	0.0237834128490713
LCTL	0.0148468913334537	0.896672590668664	0.226726528256045	0.044503496	0.950944886900442	LCTL	-0.103565828022541	0.195337257903583	-0.0577386616283367	0.471154193667963	0.326178142032126
LDB1	-0.325555900886852	0.00341493832110713	-0.349951314508277	0.001666123	0.0325340318472335	LDB1	-0.0488216709088441	0.542412099316345	0.033223880120218	0.678569388298569	0.683865686123963
LDB2	0.250930516406364	0.0257084073094553	0.166942551119766	0.141214910	0.129364690051595	LDB2	0.0125266028858513	0.87586527085387	-0.0251734783987806	0.753549175277129	0.924536374922094
LDB3	-0.00963129913193701	0.932863772656702	-0.0697663096397274	0.540469141	0.967753547362127	LDB3	0.0636606344100755	0.426814581746274	0.125150887985816	0.117161974858767	0.582091743829091
LDHA	0.216678484222674	0.0551063437879917	0.338729308666018	0.002379602	0.213574436483826	LDHA	0.348229328449617	7.34066094754127e-06	0.361498330058969	3.0609702318219e-06	4.80076618274369e-05
LDHAL6A	-0.0334784305749256	0.769597066240877	-0.0430038168808493	0.706687887	0.887946257926514	LDHAL6A	-0.00703786879779408	0.930064820137411	-0.0143483459545636	0.857990284223574	0.958144433255881
LDHAL6B	0.114282384659379	0.315926526985513	0.0753408664912058	0.509311895	0.588305701037518	LDHAL6B	0.0698689114793552	0.383027418563468	0.0686789427597608	0.391202213900515	0.538655165820292
LDHB	-0.19720148681382	0.0815123792506153	-0.183300876338851	0.105823852	0.272176707567416	LDHB	0.194537792842177	0.0143121101335748	0.244667289361353	0.0019468121635227	0.0389541613777516
LDHC	-0.065023781478997	0.569128760385044	-0.0399183726678666	0.726874415	0.779593185786246	LDHC	0.17626208640441	0.0267352067813542	0.126924750288275	0.112018558007269	0.0661035505454037
LDHD	-0.300948241139175	0.00703811060188212	-0.347492697176242	0.001803347	0.0542055107025018	LDHD	-0.172212083318093	0.0304892455915105	-0.274010508733445	0.000494290769186825	0.0737397545112596
LDLR	0.193746388193033	0.0871070753221246	0.253042843232717	0.024700174	0.283695240979426	LDLR	0.143959236482919	0.0711399643710975	0.230344629300517	0.00359519441580165	0.147753468419664
LDLRAD3	0.0802154649214839	0.482217745770912	0.138875365141188	0.221749942	0.721702805588884	LDLRAD3	0.181619728344173	0.0223817963602395	0.184209985875341	0.0205057940271654	0.056857854421417
LDLRAP1	-0.0341138424949701	0.765348737980299	0.0121713729308666	0.915102924	0.886181266829796	LDLRAP1	-0.192547968356591	0.0153591521672589	-0.206496150882721	0.00923784806878896	0.0414060611215751
LDOC1	-0.267102856814687	0.0173300511268053	-0.32991723466407	0.003121421	0.100191099440501	LDOC1	0.0164116394243121	0.837832406233814	0.11783372391172	0.140339458721272	0.900502559671629
LDOC1L	0.129822910980559	0.254147707953933	0.196445959104187	0.082754515	0.527976649293074	LDOC1L	-0.0627136415196661	0.433738774478895	-0.0324450052749088	0.685701215025019	0.588309941660389
LEAP2	0.0859821939024005	0.451184257715562	-0.0463242453748783	0.684604706	0.699039664376299	LEAP2	0.263640793205257	0.000816864095553704	0.0551495532114921	0.491302428876096	0.00321197522408024
LECT1	0.0444425629301166	0.697342592137831	0.130420061594536	0.251951115	0.849682219523289	LECT1	-0.0147140873207408	0.854409994687629	0.0841869390903381	0.292946802458204	0.911654572980135
LECT2	0.0150644613943786	0.895166915308496	0.172149972140589	0.129250467	0.950249515124744	LECT2	0.229666962528378	0.00369781109930314	0.147078264110515	0.0651674952050006	0.012066058549836
LEF1	-0.0798867258302593	0.484020460476308	0.0388266796494645	0.733564206	0.722802967525228	LEF1	0.0767592966520982	0.337758606509892	0.0911284262152434	0.254810660488274	0.491474545198159
LEFTY1	0.331291483909869	0.00286038449442794	0.366964884131632	0.000879345	0.0289226403531676	LEFTY1	0.0729415951236167	0.36240318048746	0.101008887090444	0.20665644327542	0.517598336134534
LEFTY2	0.219764512842555	0.0516502486828769	0.115065787757075	0.312598581	0.204922491163478	LEFTY2	-0.235421127157029	0.00290450065799834	-0.258898302828744	0.00102109692200085	0.00973640230666833
LEKR1	-0.0917140036946646	0.421469208394245	-0.0789435248296008	0.488426531	0.678425816197719	LEKR1	-0.0106194081188208	0.894645995151538	-0.0472740521344274	0.555299808912444	0.937151224508667
LEMD1	0.198712939143122	0.0791566091235927	0.148777468746292	0.190676045	0.268246294901675	LEMD1	0.0105086988076473	0.895738084909196	0.0181225588815879	0.82119612545982	0.937778987886962
LEMD2	-0.0103985495014974	0.927529463541531	0.0213242453748783	0.851769078	0.965308548845312	LEMD2	0.149645628707402	0.0605644424142477	0.0923453488469649	0.248494481266137	0.129635087181293
LEMD3	-0.0386623277575983	0.735146557776779	0.0066699123661149	0.953454088	0.870170172522772	LEMD3	0.469997769089707	4.6530290731539e-10	0.3558666998298	4.45838291040381e-06	7.2472305614859e-09
LENEP	-0.081864735556444	0.473227861577253	-0.0315798056621871	0.782331158	0.714993316986762	LENEP	0.129712083570192	0.104294875403591	0.141721754567754	0.0756923205604907	0.200886028058183
LENG1	-0.0633705455573503	0.579010870419313	-0.0596640701071081	0.600747406	0.784684406358396	LENG1	0.174498617717537	0.0283182049347133	0.184966042121666	0.0199844932107409	0.0693914751561389
LENG8	0.0522713503496771	0.64730769525879	0.056840311587147	0.618115954	0.821834286369156	LENG8	-0.127540483207046	0.110275031536018	-0.150916128718239	0.0583872436160326	0.209836003160526
LENG9	0.0779234682655865	0.494860621557807	0.147346640701071	0.194620540	0.730867909550406	LENG9	0.217343138439349	0.0060854959848633	0.254155270035506	0.0012712285097054	0.0186164807380082
LEO1	0.152479873653423	0.179749015589765	0.163972736124635	0.148504864	0.43593820114462	LEO1	0.0704537354439951	0.379048094073386	-0.141250170188758	0.0766812180124243	0.534990539592045
LEP	0.213091154223086	0.0593590085370836	0.197837057080264	0.080515064	0.223711376571428	LEP	-0.174127385362524	0.0286614473694903	-0.133564866051059	0.0943158308715068	0.0709926203602971
LEPRE1	0.45798893773504	2.19560674046271e-05	0.577604673807206	4.684424015	0.000704486604853126	LEPRE1	0.482163335055858	1.41179068435804e-10	0.36911061467992	1.82018242745921e-06	2.47533966657443e-09
LEPREL1	0.15041536255992	0.185784379479066	0.134079844206426	0.238240489	0.44524976692108	LEPREL1	-0.111409738700153	0.163431917432886	-0.144948304464044	0.0691997378938096	0.285030466079901

[illegible]

LY6G6E	0.0370439414026709	0.745849473044125	0.119593467129822	0.293809890	0.875610546558242	LY6G6E	-0.114146759602438	0.153268330533651	-0.0922115498708466	0.249183580979411	0.271392706081034
LY6H	-0.0843575365298185	0.459813398047338	-0.0686713039892463	0.547609744	0.705274449314512	LY6H	0.0081188262902179	0.919357244016584	0.110469737501932	0.167036109935175	0.952172712833676
LY6K	0.257783585973416	0.0218122199130169	-0.0437206358609546	0.702026307	0.116312236570409	LY6K	0.0628973191498139	0.43239076149224	0.114453615837945	0.152159217423904	0.587217019701397
LY86	-0.0979325676415802	0.390541332071493	-0.230306718597858	0.041373280	0.653396087632256	LY86	-0.512575162999175	5.78419572539086e-12	-0.460488454929977	1.1447084719994e-09	1.30179759540887e-10
LY9	-0.108921767517528	0.33930791186451	-0.1531994064137	0.177679487	0.610267969840021	LY9	0.107574924250371	0.178506917766568	0.0377267503196614	0.637912087260027	0.304826768197128
LY96	-0.188164467138721	0.0967828294160039	-0.251752677702045	0.025462284	0.30326240367525	LY96	-0.361689437869029	3.02177583733934e-06	-0.348601257913401	7.16669820552216e-06	2.15790896685094e-05
LYAR	0.522580522937985	7.80791233001565e-07	0.408812074001947	0.000207966	4.09615093005436e-05	LYAR	0.66669191846044	0	0.396206940353057	2.56109714628791e-07	0
LYG1	0.230223165544529	0.0412350431596034	0.509079844206427	2.351283708	0.177650287724711	LYG1	0.264430692146039	0.0007867454594912	0.117143170083881	0.142695540848213	0.00310647323638476
LYG2	0.0484128180317407	0.671790126087205	0.0590218134553078	0.605375040	0.834326995177711	LYG2	0.0842871151460047	0.292370787840461	0.0318091269832307	0.691544349282073	0.44198934177557
LYL1	0.00269484995605034	0.98119511926783	-0.175851996105161	0.120968204	0.991942984466387	LYL1	-0.311420435568404	6.8074702896211e-05	-0.255927515904576	0.00117185634285829	0.000354026915344419
LYN	0.0470454795249003	0.680550169241619	-0.0623661148977605	0.584334070	0.839447481977844	LYN	-0.513340533955597	5.31528898691259e-12	-0.326178181312118	2.88435781254722e-05	1.20807987961803e-10
LYNX1	-0.294334138485799	0.00846340773542491	-0.350267770204479	0.001649165	0.0616308093487606	LYNX1	-0.0826807955283184	0.301698082405468	-0.0481700624545193	0.5478202935634	0.452559414738423
LYPD1	0.269411849403677	0.0163506735875305	0.327217181220648	0.003245198	0.0963024485450203	LYPD1	0.045479184662233	0.570429545488646	0.113597070700419	0.155270354847813	0.707607838585403
LYPD3	0.312506782878073	0.00504820933627492	0.103529698149951	0.363145122	0.0431977891573883	LYPD3	-0.0570087312182561	0.476788331167574	-0.0719259093613588	0.369143055194579	0.627963455869965
LYPD4	-0.0269325371714815	0.813736252467286	0.0146080512167141	0.898325883	0.911301220241548	LYPD4	0.0138275345207772	0.863093553985757	-0.000762142945158536	0.992417082346272	0.916828361253118
LYPD5	-0.0728971261578942	0.523182952016763	0.0199367088607595	0.861320036	0.748827746295455	LYPD5	0.14088628942097	0.077451371412415	0.052493426341958	0.512438985750581	0.158395883992731
LYPD6	0.403983934283469	0.000222373726425529	0.352053841549272	0.001463791	0.00443357617060898	LYPD6	0.0901756311345466	0.25983249861303	0.138225945203456	0.0832733627413649	0.403399924056829
LYPD6B	0.0286830807973913	0.801869114844649	-0.0728584782338795	0.523403840	0.905396056177116	LYPD6B	0.182701295794756	0.0215812203382149	0.288619206444066	0.000235384157236453	0.055258729683802
LYPLA1	0.126745513574731	0.265674680272401	0.0786027263875365	0.490310355	0.538648265138652	LYPLA1	0.0580141837170668	0.46903700724559	0.0811627141050362	0.310691380037941	0.620838027370074
LYPLA2	0.27821408386218	0.013041590088825	0.227263875365141	0.044192124	0.0832321938618645	LYPLA2	0.205650799312205	0.00953550753437771	0.220303092074638	0.00541190428299436	0.0275456917790513
LYPLAL1	-0.330995885619995	0.00288686307505227	-0.308057448880234	0.005929461	0.0290862852258705	LYPLAL1	-0.383108754898754	6.75596559852886e-07	-0.328586304124861	2.49651951112944e-05	5.61016358452482e-06
LYRM1	-0.293975767069005	0.0085473917827643	-0.366333982473223	0.000968231	0.0619427983587107	LYRM1	-0.304469273670885	0.000100456500677834	-0.363489633130277	2.67524557829559e-06	0.000499838966886196
LYRM2	-0.0494711200388109	0.665039768813166	0.0192064264849075	0.866354985	0.831258199559281	LYRM2	-0.0527968928846621	0.510000573579499	0.0247886049659227	0.757198806605756	0.65639846408893
LYRM4	0.115752369865551	0.309700621458579	0.0754381694255112	0.507985312	0.582182993319407	LYRM4	-0.450804473418788	2.78423835409552e-09	-0.326488514258296	2.83137130668384e-05	3.75240322832346e-08
LYRM5	-0.218762149671089	0.0527527592114031	-0.147127555988316	0.195290477	0.207671988790432	LYRM5	-0.143594416044706	0.0718666779053207	0.0194064725765869	0.808763550346546	0.148952515158732
LYRM7	-0.26751809712374	0.0171502928744181	-0.207424537487829	0.066731897	0.0994425366374205	LYRM7	0.0499482104602788	0.53312471448744	0.197902666505472	0.0126829846635495	0.676400379690596
LYSMD1	0.0391235078044697	0.732105730257026	0.0850778967867575	0.455184605	0.868771499300268	LYSMD1	0.280851351295356	0.000350987327597307	0.160481685412192	0.0439800484685558	0.00152398035402551
LYSMD2	-0.0938915440479002	0.410482424249709	-0.128334956183057	0.259094730	0.669900271211382	LYSMD2	-0.354217524087353	4.97069475317577e-06	-0.299982429678869	0.000128479841746873	3.38551573828953e-05
LYSMD3	-0.186746322521091	0.0993705655092347	-0.121713729308666	0.284634891	0.307807524611064	LYSMD3	0.00311195443526074	0.969044938499452	-0.0514012151673822	0.521264674096309	0.982818274447714
LYSMD4	-0.099542826703861	0.382758881949883	-0.0613680623174294	0.590372690	0.646307962708511	LYSMD4	0.326720753877535	2.79231948014935e-05	0.295879870613658	0.000160335472235488	0.000160045459618772
LYST	-0.157198861554749	0.166492471387414	-0.142429406037001	0.210059909	0.418732738661259	LYST	-0.244320064874474	0.00197681699898976	-0.190568768792122	0.0164662284543694	0.00695588703192532
LYVE1	0.111691170237684	0.327095981189445	0.304673807205453	0.006522976	0.598898751709391	LYVE1	-0.356593085611145	4.24899749781649e-06	-0.236730794536079	0.00274697594844538	2.9474018061342e-05
LYZ	-0.00802766913178483	0.94402228793593	-0.17706913398247	0.118385497	0.972897337856121	LYZ	-0.34119800462106	1.1486167372388e-05	-0.25498415248308	0.00122383050896894	7.21707649575642e-05
LYZL1	0.0236135244357282	0.836349577739806	0.117432376551233	0.302683176	0.922550025016056	LYZL1	0.0557568285965479	0.486535912187945	0.10653859845016	0.182751384396994	0.636430733507181
LYZL4	0.244239665026427	0.030066378583915	0.168822350988528	0.136933800	0.143809633768481	LYZL4	0.0490124321697093	0.540833829866652	0.110825536237791	0.165664996336309	0.682717416884604
LYZL6	0.0440184397928638	0.700092859870065	0.170291638148444	0.133499751	0.850833785887391	LYZL6	-0.111815845985059	0.161892888902021	-0.106613058171485	0.182443972003852	0.283124164974466
LZIC	0.362546147610618	0.00102530342462614	0.238169425511198	0.034776295	0.0141018436246563	LZIC	0.0162347162098007	0.83955697212611	-0.0701369804666418	0.38120025694513	0.901291262280115
LZTFL1	-0.145616662121299	0.200377428145784	-0.186124634858812	0.100486340	0.46316957065656	LZTFL1	-0.0546013969003064	0.495626152448222	-0.13738774200079	0.085178776384802	0.644247508760274
LZTR1	0.0361743001759439	0.751620763919415	0.0517526777020448	0.649941055	0.878942557133459	LZTR1	-0.0182794711544709	0.819674235441628	-0.151244716543604	0.0578347600999272	0.889123419424957
LZTS1	0.250083371332832	0.0262287048726426	0.097078870496592	0.393931306	0.131160518402695	LZTS1	-0.00356355979163764	0.964555496701809	0.0679066809866161	0.396563159058961	0.979884488151435
LZTS2	-0.200096360205042	0.0770482724803315	-0.216942551119766	0.054967350	0.263643725452595	LZTS2	-0.143375071418528	0.0723064984001143	-0.137504877475573	0.084910422890296	0.149686566461948
M6PR	-0.11043099185717	0.332617732445598	-0.031110029211295	0.785092660	0.604355684414174	M6PR	0.356329793734111	4.32379112669246e-06	0.288574651426968	0.000235931968302331	2.99251859557926e-05
MAB21L1	0.164086765281624	0.148457034122424	-0.248535708705982	0.027202294	0.394348204766105	MAB21L1	-0.0824318263966471	0.3031611192043	-0.087813708674529	0.272572489245936	0.454346809227545
MAB21L2	0.0329022345541772	0.773455354685773	-0.0227868988399113	0.842003047	0.889952877129282	MAB21L2	-0.0173537469262129	0.828662356779918	0.0837959804277513	0.295202037313957	0.895180963990042
MACC1	-0.0723485935833557	0.52632242241633	-0.0831791626095424	0.465337461	0.750945380937107	MACC1	-0.141183771101838	0.0768212887716277	-0.265429793423286	0.000750112472867886	0.157311864382989
MACF1	0.160084143846209	0.158750434953792	0.278042843232717	0.013338664	0.408904911380829	MACF1	-0.30102249110209	0.000121400229079287	-0.307209840596963	8.62688237376977e-05	0.000590795193589657
MACROD1	-0.140171507476287	0.217909165567944	-0.174464459591042	0.123965221	0.486098162640279	MACROD1	-0.0270736591736993	0.735611622015609	-0.174308235156362	0.0284937924711721	0.831549180971838
MACROD2	0.0465551057522163	0.683702214652923	-0.163875365141188	0.148748583	0.841369723502236	MACROD2	-0.0210018079978649	0.793382066999409	0.134745348238481	0.091414886404447	0.872478576755219
MAD1L1	0.226357429285779	0.0448606769266768	0.282935735150925	0.011750391	0.187421729669585	MAD1L1	0.180442005316092	0.0232825008625603	0.0670927659717076	0.402260522079916	0.058749339029308
MAD2L1	0.556988645733811	9.77054566142499e-08	0.561708860759494	1.263747366	6.51172341824012e-06	MAD2L1	0.791989898773459	0	0.653040920593746	1.42542514194092e-20	0
MAD2L1BP	0.211358493801119	0.0615066612614348	0.0842259006815969	0.459725101	0.229091861447566	MAD2L1BP	0.352385743036027	5.60503364521736e-06	0.30009979341546	0.00012766208982215	3.77289467672584e-05
MAD2L2	0.56763677661708	4.89152138793969e-08	0.474902629016553	1.254788987	3.4800183457076e-06	MAD2L2	0.548741031144954	8.37108160567368e-14	0.327676602645379	2.63686671034801e-05	2.64342388268358e-12
MADCAM1	-0.136747204007837	0.229473040287329	-0.0120374097541868	0.916146270	0.500277534259567	MADCAM1	0.0872999865766527	0.275398566860792	0.117684731911683	0.140845276760849	0.422050080405159
MADD	-0.316012492572058	0.00455251494565603	-0.107521908471276	0.344857765	0.0399998403354887	MADD	-0.0924420219164117	0.247997411883592	0.0179354188011616	0.823012066012741	0.389260154555114
MAEA	-0.142609920120616	0.209929062516763	-0.18300876338851	0.106388417	0.476089553773253	MAEA	0.0172092481521626	0.830067374925483	-0.0610367098014426	0.44615652885315	0.896067806519183

[illegible]

MLF1	0.279432218090201	0.0126328551038131	0.319401168451801	0.004273763	0.0813393259244399	MLF1	0.296261054759275	0.000157091344227922	0.290203899394402	0.000216651609430924	[illegible]	MLF1	[illegible]	[illegible]	[illegible]	[illegible]	[illegible]
MLF1IP	0.637979719944778	2.55207854849004e-10	0.658666017526777	0	2.74320703978457e-08	MLF1IP	0.74448278208467	0	0.620837792468064	3.27690294194075e-18	[illegible]	MLF1IP	[illegible]	[illegible]	[illegible]	[illegible]	[illegible]
MLF2	-0.0423680292001032	0.710831559534506	0.0146786757546251	0.897683373	0.857297797699461	MLF2	0.189006318562467	0.0173886029496448	0.274944341634059	0.000471965205706205	[illegible]	MLF2	[illegible]	[illegible]	[illegible]	[illegible]	[illegible]
MLH1	0.022909819161657	0.841161855940711	0.101874391431353	0.370899845	0.924460672109075	MLH1	-0.0145965223625396	0.855560533160975	-0.102648706908056	0.19934426288946	[illegible]	MLH1	[illegible]	[illegible]	[illegible]	[illegible]	[illegible]
MLH3	-0.201717255994403	0.0746353639442	-0.278383641674781	0.013222278	0.258060433449855	MLH3	-0.429611737061624	1.77223175454346e-08	-0.168462110096746	0.034356039245082	[illegible]	MLH3	[illegible]	[illegible]	[illegible]	[illegible]	[illegible]
MLKL	0.413595906505651	0.000151466300917225	-0.0763145082765336	0.503057960	0.00325619619515313	MLKL	-0.00720942483076695	0.928364560444465	-0.138976339323089	0.081596707458926	[illegible]	MLKL	[illegible]	[illegible]	[illegible]	[illegible]	[illegible]
MLLT1	0.0692508899088489	0.544227089394017	0.137341772151899	0.226933507	0.762411345377169	MLLT1	0.283864646702191	0.000301006681295402	0.319495374289768	4.27896185040307e-05	[illegible]	MLLT1	[illegible]	[illegible]	[illegible]	[illegible]	[illegible]
MLLT10	0.100573509940184	0.377826819029863	0.12429406037001	0.274489065	0.642507651010974	MLLT10	0.0211350001358038	0.792101394567124	0.0751781562115867	0.347833084034377	[illegible]	MLLT10	[illegible]	[illegible]	[illegible]	[illegible]	[illegible]
MLLT11	0.0381536247084322	0.738505457053154	0.0731499513145083	0.520967827	0.872009607737151	MLLT11	0.403284436369282	1.48979790726145e-07	0.313813781835773	5.94047360462049e-05	[illegible]	MLLT11	[illegible]	[illegible]	[illegible]	[illegible]	[illegible]
MLLT3	-0.0470406053269366	0.680581473026329	-0.0060856864654333	0.957534570	0.839447481977844	MLLT3	-0.0700594173254949	0.381728382712581	-0.058555567471952	0.46489209255993	[illegible]	MLLT3	[illegible]	[illegible]	[illegible]	[illegible]	[illegible]
MLLT4	-0.0506765190201352	0.657383486789216	-0.0602726387536514	0.597032835	0.826813048019301	MLLT4	-0.0241818123350637	0.762963696337763	0.0545273241313688	0.496211959433012	[illegible]	MLLT4	[illegible]	[illegible]	[illegible]	[illegible]	[illegible]
MLLT6	-0.0648559145100833	0.570128562777658	-0.0744401168451801	0.513627256	0.779806550111793	MLLT6	-0.231435730475838	0.00343535349334211	-0.27651121876427	0.000436587127254724	[illegible]	MLLT6	[illegible]	[illegible]	[illegible]	[illegible]	[illegible]
MLN	0.305707317389989	0.00614769028676321	0.240008073168157	0.033131787	0.0495274741300781	MLN	0.119590522536482	0.134479137787257	0.122990820831267	0.12367101546973	[illegible]	MLN	[illegible]	[illegible]	[illegible]	[illegible]	[illegible]
MLNR	0.100468278839681	0.378328607835672	0.126065248119189	0.268269630	0.642999330220325	MLNR	0.0330667465430444	0.680005925261831	0.0898180819694545	0.261734403793229	[illegible]	MLNR	[illegible]	[illegible]	[illegible]	[illegible]	[illegible]
MLPH	0.311812450002683	0.00515187520515958	0.0659850284223689	0.563419516	0.0438064435974403	MLPH	0.168248064710378	0.0345887398251046	0.181348533562789	0.0225864989460114	[illegible]	MLPH	[illegible]	[illegible]	[illegible]	[illegible]	[illegible]
MLSTB	-0.196163677975774	0.0831619710224198	-0.197541382667965	0.081029367	0.275599440831337	MLSTB	0.0842074092737083	0.292829037146743	0.0951094418038533	0.234553349066729	[illegible]	MLSTB	[illegible]	[illegible]	[illegible]	[illegible]	[illegible]
MLX	0.0619460274596915	0.587588663710651	-0.0289922103213242	0.799409126	0.789867884537091	MLX	-0.0790238658467938	0.323656463990871	-0.166466243305642	0.0365781032857585	[illegible]	MLX	[illegible]	[illegible]	[illegible]	[illegible]	[illegible]
MLXIP	-0.161185091415432	0.155867796714377	0.00866601752677702	0.939522147	0.405518716576815	MLXIP	-0.0577315808061919	0.471208673353084	0.145316444527648	0.0684886525748866	[illegible]	MLXIP	[illegible]	[illegible]	[illegible]	[illegible]	[illegible]
MLXIPL	-0.0225686553685974	0.843497006834528	-0.0338120740019474	0.766930911	0.925528239050546	MLXIPL	-0.173708748478874	0.0290527708132692	-0.15240097481129	0.0559247528275705	[illegible]	MLXIPL	[illegible]	[illegible]	[illegible]	[illegible]	[illegible]
MLYCD	-0.203807040665446	0.071614177989193	-0.147200584225901	0.195066980	0.251528815183089	MLYCD	-0.35216990202012	5.68464128501399e-06	-0.20196741960443	0.0109335975073479	[illegible]	MLYCD	[illegible]	[illegible]	[illegible]	[illegible]	[illegible]
MMAA	-0.4239787312398	9.87278191873258e-05	-0.494961051606621	4.789610749	0.0023053586869975	MMAA	-0.153658802501386	0.0539063608076591	-0.184503725519428	0.0203018818311275	[illegible]	MMAA	[illegible]	[illegible]	[illegible]	[illegible]	[illegible]
[illegible]																	

NAPG	-0.224679824129547	0.0465141053509033	-0.0976874391431354	0.390962175	0.191885351090399	NAPG	0.0195700731621661	0.807182709223541	-0.145287540968935	0.0685442655592454
NAPRT1	0.282823352168803	0.0115530008450024	0.20898247322298	0.064677287	0.0763432177455588	NAPRT1	-0.440679648375554	6.84855824738135e-09	-0.408909293787298	9.59826934337275e-08
NAPSA	0.0587651366018486	0.606947490009349	0.123870968659685	0.276755998	0.800121630336759	NAPSA	0.000876995219568 72	0.991274405356705	0.0351741097133553	0.66083773388689
NAPSB	-0.195658083298278	0.0839751594688987	-0.209639727361246	0.063826062	0.277491367864917	NAPSB	-0.22665402912355 4	0.00418670623675265	-0.218955105927803	0.0057099263958 5223
NARFL	-0.260417826784675	0.0204548111782331	-0.191066212268744	0.091656681	0.112003116135962	NARFL	0.150388078129943	0.0592841912165234	0.116428983799118	0.145163759280207
NARG2	-0.0969914926813528	0.395132902166199	-0.120326192794547	0.290192728	0.657491043163645	NARG2	0.270261369049296	0.000594098063288806	0.0698020942910349	0.383483671311833
NARS	-0.237113736361 68	0.0353761119640014	-0.240701071080818	0.032850313	0.159960846461824	NARS	-0.115619830438 86	0.147999161270019	-0.269337051243806	0.000621407440629106
NARS2	-0.128871960791428	0.257672637943048	-0.162000973709834	0.153499014	0.531487557687364	NARS2	-0.31552126399204	5.38644497644374e-05	-0.377559697768443	1.00628296074561e-06
NASP	0.579022230789016	2.27097052274416e-08	0.534931840311587	5.92260891 0	1.72089099612391e-06	NASP	0.305260652118433	9.61502995604491e-05	-0.00497445457843923	0.950537497371948
NAT1	-0.135177688126076	0.234913863878228	-0.168232716650438	0.138133724	0.507108316937742	NAT1	0.257818003736063	0.00107373121967 18	0.254503827825876	0.00125109609807371
NAT10	0.189416554237966	0.0945422935262921	0.0126582278481013	0.911717081	0.298596628168431	NAT10	-0.234252546471274	0.00305190026749828	-0.198964491978179	0.0122038087941122
NAT14	0.197905204849301	0.080408708745 07	0.308860759493671	0.005795795	0.270187468834522	NAT14	0.376265920326847	1.10307789702446e-06	0.402228313947651	1.61654169941571e-07
NAT2	0.0431427398243683	0.705783601714415	0.229525030843096	0.041871188	0.854283235906224	NAT2	0.346383527432176	8.26479671856006e-06	0.344349654221 9	9.41024243949472e-06
NAT8B	-0.0208174592569868	0.855504168216294	-0.186493172895347	0.099838136	0.93239441444767	NAT8B	-0.0192017624168944	0.810742714026491	-0.0270279315501631	0.736041659175894
NAT8L	0.000427023758976105	0.997019929996797	0.255428432327166	0.023342174	0.998561431344425	NAT8L	0.202539278049516	0.0107053552827989	0.120926404693973	0.130149742575575
NAT9	-0.0331584490953494	0.771739022766387	0.00922590068159688	0.935617498	0.889219597569 1	NAT9	-0.248240223864263	0.00166125443530687	-0.297487596189988	0.000147063053539384
NAV1	0.415177400213736	0.000142034000091495	0.470715676728335	1.523722023	0.00308697632286768	NAV1	0.267085450705282	0.000692843293508716	0.131097414880959	0.100615228778474
NAV2	0.396077001349658	0.000302373112476495	0.502921129503408	3.218153950	0.00559956499865 18	NAV2	0.111079330409507	0.164692106257986	0.0211741547223887	0.791725019862345
NAV3	0.305253453042492	0.00622809120209933	-0.0867818889970789	0.446178693	0.0498780139799912	NAV3	-0.101585329085189	0.204064343836863	-0.247177149150801	0.00174194859035583
NBAS	0.158940000421891	0.161787896808873	0.242234664070107	0.031727951	0.413095923455447	NBAS	-0.112903540349129	0.157824239069977	-0.138601691466721	0.0824303854303813
NBEA	-0.199501012458677	0.0779500174519487	-0.271884128529698	0.015600953	0.265869116547321	NBEA	-0.537927138708297	3.13223447088998e-13	-0.445342990164552	4.5409463265224e-09
NBEAL2	0.0925449598866 92	0.417256806033831	0.00357838364167478	0.975057782	0.67488592654049	NBEAL2	-0.0925641249043176	0.247370573449501	-0.151214291744959	0.0578857345347475
NBLA00301	0.148257264144355	0.192248944422427	0.307238361595571	0.005883187	0.452584116022257	NBLA00301	-0.127571966916614	0.110186455195762	-0.212912324116103	0.00723382454716343
NBN	0.0176936740209904	0.877002466674319	0.0207156767283 35	0.855955543	0.942628034619391	NBN	0.198321681921081	0.0124919580711971	0.223391211485979	0.00478096088145294
NBPF1	0.14163793061924	0.213084827094372	0.299026290165531	0.007631316	0.479944392469251	NBPF1	-0.177689926462116	0.0255095269133808	-0.265289031784103	0.000755176900276457
NBPF4	0.0313565874918574	0.783832120087785	-0.0151485648754861	0.894584983	0.896105876941978	NBPF4	-0.0255814895466424	0.749686086396775	0.00771572893634366	0.923348592452069
NBR1	-0.407254450249959	0.000195390668579 58	-0.520350535540409	1.305115473	0.00396861773279544	NBR1	-0.291839718911915	0.000198775820842945	-0.249227780579467	0.00158935968062832
NBR2	-0.138853998303739	0.222308796029458	-0.193768257059396	0.087095097	0.491654791109067	NBR2	-0.0822911667665367	0.303989749791261	-0.120191645806699	0.132517534615974
NCALD	0.0150387861841015	0.895344579286251	0.0502921129503408	0.659198295	0.950363606994229	NCALD	0.0191656078496002	0.811092385860391	-0.0369022382763 85	0.645281071046093
NCAM1	-0.164678695335 39	0.146978215528522	-0.0599074975657254	0.599260343	0.392563828051736	NCAM1	-0.290426482114709	0.000214134388199164	-0.215047040541867	0.00665850386331 82
NCAM2	0.234249885973128	0.0377205911800709	0.269198466812232	0.016439136	0.167354833991143	NCAM2	-0.0656664971985031	0.412360772675872	-0.0775908427441263	0.33253555075854
NCAN	-0.0875463515219691	0.442962045814634	0.28954497208097	0.009647682	0.693383723159623	NCAN	-0.0564306747726721	0.481275991253553	0.0747035293527264	0.350893793233563
NCAPD2	0.710496379534739	2.23598917159507e-13	0.745350535540409	0	4.1330274612904e-11	NCAPD2	0.764601659964731	0	0.546321791170108	1.12907284222525e-13
NCAPD3	0.649520420738641	9.44577749351083e-11	0.656280428432327	0	1.14612630224943e-08	NCAPD3	0.594745183701917	2.22044604925031e-16	0.320190580938804	4.10871988663794e-05
NCAPG	0.791655182243935	0	0.768573515092502	0	0	NCAPG	0.818386048747984	0	0.569894509617063	5.49658325469326e-15
NCAPG2	0.693727061777982	1.36779476633819e-12	0.690847127555988	0	2.2173971220194e-10	NCAPG2	0.764533091201993	0	0.555747400959599	3.46929636123252e-14
NCAPH	0.646019348053816	1.28270283283882e-10	0.758227848101266	0	1.50114181230096e-08	NCAPH	0.822883492130266	0	0.501669946217705	1.88617251900293e-11
NCAPH2	0.60642502885959	3.16006643075184e-09	0.506791626095424	2.643769038	2.81530603006351e-07	NCAPH2	0.179463201135137	0.0240546199439 56	0.0940476163311464	0.239842484305615
NCBP1	0.479874083664271	7.64194538538376e-06	0.510029211295034	2.239147095	0.000290100567815969	NCBP1	0.637387605376702	0	0.370386934983073	1.6660858292166e-06
NCBP2	0.172259191268133	0.129003968861561	0.0538704965920156	0.636612402	0.363919625751527	NCBP2	-0.166575719882516	0.0364533151023 7078	-0.317140494874654	4.90616568991811e-05

[illegible]

NTNG2	-0.0141781207934173	0.901303022998972	0.161708860759494	0.154249463	0.953158549292785	NTNG2	0.377635875937085	1.00084367549158e-06	0.0718771202297683	0.369468740753291	7.97296930584162e-06
NTRK1	0.211010532810407	0.0619454175953902	0.0340056727414718	0.766071470	0.229859032628024	NTRK1	-0.0302274846139352	0.70615665577036	0.0398626015048117	0.618988627992368	0.809082899721952
NTRK2	0.289532801735447	0.00965086784730573	-0.00636566678241329	0.955598322	0.0669736365908817	NTRK2	-0.0767784890412678	0.337637471129958	-0.0767350959022049	0.337906277797561	0.491375956008105
NTRK3	-0.00127234496380975	0.991120847735112	-0.0979931446517122	0.390246865	0.996258798866896	NTRK3	-0.27991850757871	0.000367953686806664	-0.20295926804025 3	0.0105404098754878	0.00158679488735307
NTS	0.0615864614330721	0.589762879981516	0.121385989011053	0.286581026	0.791231192529808	NTS	0.00216598397131322	0.978451882945313	0.182249107602677	0.021912875920853 8	0.987625372280455
NTSR1	0.159470004427715	0.160375542576118	0.2122121345097 8	0.060440787	0.411028570826806	NTSR1	0.0884528881130749	0.269083773205157	0.176969018403975	0.0261222110923165	0.414407919082 06
NTSR2	-0.148340649484279	0.191996191764455	-0.141211716188282	0.214479156	0.452113427874436	NTSR2	0.0336509974688486	0.674670475746226	0.157649136658353	0.0478979525517059	0.787065677617896
NUAK1	-0.116009458368888	0.308619981172744	-0.0964946445959104	0.396794287	0.581322475012812	NUAK1	0.125604600191471	0.115829255048887	0.183692764298377	0.0208691471744834	0.217999326133797
NUB1	-0.188064342875814	0.0969637783057852	-0.126071080817916	0.267645079	0.303498972480708	NUB1	0.220024659100536	0.00547229706259111	0.254832028489855	0.00123240643074226	0.0170465294284776
NUBP1	-0.210674692928292	0.0623713421455208	-0.178651411879 26	0.115091913	0.230690551701773	NUBP1	-0.155055782185177	0.0517356458115272	-0.0151059240170501	0.850577548122876	0.114443367272325
NUBP2	0.102044240614173	0.370855859826074	0.126095423563778	0.267552136	0.637202137093452	NUBP2	0.172474400563322	0.0302331967707099	0.0979602454368 79	0.220759371200683	0.0732061354315225
NUBPL	-0.150890969740286	0.184381143778919	-0.147857838364167	0.193063816	0.442613212969955	NUBPL	-0.0513760039641019	0.521469306012447	0.0577584377474559	0.471002054884411	0.666545158903642
NUCB1	-0.0885101150622358	0.437937997521229	-0.0374391431353457	0.742750230	0.689228751092136	NUCB1	0.258167850939417	0.00105641993905103	0.187508176670855	0.0183148220096065	0.00403918817817 85
NUCB2	-0.0967559859989292	0.396286940518322	-0.00525803310613437	0.963317106	0.658427888534508	NUCB2	-0.00661379531363238	0.934269073669969	0.149606341136577	0.0606328156468199	0.960834235308873
NUCKS1	0.167161466097952	0.140895633052624	0.126411879259981	0.266345857	0.383682764538604	NUCKS1	0.378876017264538	9.16141190909769e-07	0.285171637698538	0.000281452663089 45	7.36192026392355e-06
NUDC	-0.0341613265268588	0.765031538408732	0.0302093476144109	0.791172823	0.886181266829796	NUDC	-0.288177047514556	0.000240873296686669	-0.347470976643743	7.70783090235856e-06	0.00108634918961332
NUDCD1	0.324516090425286	0.00352516911755796	0.177799415774099	0.116856446	0.0330710859094178	NUDCD1	0.286427601005929	0.000263778747956245	0.264601431334729	0.000780370883322333	0.00118011342645795
NUDCD2	0.0216421631450362	0.849845285254404	0.0244644595910419	0.830234758	0.928752828623 55	NUDCD2	0.399494927453403	1.99431155589735e-07	0.287792734101795	0.000245740684488974	1.84870472024523e-06
NUDCD3	-0.340650014547865	0.00212671822031886	-0.247711781888997	0.027980196	0.0234497349676549	NUDCD3	0.387346126704147	4.95901021579925e-07	0.350193996122461	6.46493117842097e-06	4.23055505434959e-06
NUDT1	0.441739655127075	4.59352159527526e-05	0.41572541382668	0.000158898	0.00127063874281614	NUDT1	0.670136087277657	0	0.445075251936477	4.65012714888485e-09	0
NUDT10	-0.120683112733703	0.289401418146656	-0.075	0.510458311	0.562810348879505	NUDT10	0.223761752060615	0.00470986576295029	0.103570735862936	0.195315973499899	0.014900950111001
NUDT11	-0.0828565115912826	0.467865707347	0.110881207400195	0.329926491	0.711330794392424	NUDT11	0.273379725252781	0.000509918744884041	0.193986994919875	0.0145955064064441	0.00212341191886795
NUDT12	-0.378894820150763	0.000574557757903 46	-0.396178188899708	0.000335379	0.00896890555312915	NUDT12	-0.214668958901522	0.00675732996655957	-0.128824682422186	0.106707024343738	0.0203604655784553
NUDT13	-0.124116364704103	0.275798121616 88	-0.349002629016553	0.001668746	0.548755568857297	NUDT13	-0.259943537297525	0.000972431233309534	-0.29276284764162	0.000189303834431515	0.00375865616062325
NUDT14	-0.133675011439411	0.240206286438386	-0.184956183057449	0.102668745	0.512436623145119	NUDT14	-0.314324888072391	5.76926931228806e-05	-0.271558486343527	0.000556818610488501	0.000304071709244842
NUDT15	0.191887672500625	0.0902401124685759	0.292064264849075	0.009223983	0.289782260822129	NUDT15	-0.0571938628291401	0.475355901364187	-0.28135028298874	0.000342213148361036	0.626793006278359
NUDT16	-0.2326999829612	0.0390425467534753	-0.274829600778968	0.014480580	0.17091268032099	NUDT16	-0.422138798337178	3.30419136802686e-08	-0.407307428138645	1.08874453293816e-07	3.61245993226357e-07
NUDT16L1	-0.141927416937268	0.212141459917783	-0.0728334956183058	0.522776439	0.478801072036279	NUDT16L1	-0.176939659497541	0.0261474272642199	-0.164258924163955	0.0391755942404162	0.0648490012035954
NUDT16P1	-0.134028741323165	0.238953100033163	-0.354373836124547	0.001354368	0.511158916580651	NUDT16P1	-0.245676957671812	0.00186190093925973	-0.295403497482814	0.000164477490824201	0.00660454649167083
NUDT18	-0.179097933932783	0.114271240639298	-0.164118792599805	0.148139848	0.335890334557601	NUDT18	-0.112957108485489	0.157625861517102	-0.0327234170725845	0.683148690186728	0.277109636223248
NUDT19	0.165592067265506	0.144718045833266	0.172955209347614	0.127289592	0.389366550195937	NUDT19	0.17736476070626	0.0257843369027393	0.228730593732406	0.00384394132967628	0.0641039355002607
NUDT2	-0.0284773276169732	0.803261673786819	-0.118476144109056	0.297714395	0.905894080615164	NUDT2	-0.190545339638267	0.016479738911492	-0.247905823078346	0.00168626264744695	0.0439698541102272
NUDT21	0.440522508327677	4.84742234174718e-05	0.326630963972736	0.003447306	0.00132391086576476	NUDT21	0.409750030444987	8.98155576578574e-08	0.24481770601589	0.00193394317472237	8.93786171070894e-07
NUDT22	-0.0423391858499832	0.711019742592717	-0.156402142161636	0.168364907	0.857389370657264	NUDT22	-0.262241716260426	0.000872823462272578	-0.219974336682407	0.00548327617079898	0.0034094377128025
NUDT4	-0.282107240521202	0.0117740964725946	-0.254406037000974	0.023916153	0.0773688094230914	NUDT4	-0.214151975526226	0.00689478430961768	-0.193162631424766	0.0144203889048186	0.0207169869658987
NUDT5	0.510924003922715	1.50094518058808e-06	0.355598831548199	0.001386015	7.22279654055257e-05	NUDT5	0.169053198423177	0.0337203043750272	0.0731594708214983	0.36096740960 95	0.0800710439241907
NUDT6	-0.209059035070467	0.0644536897825206	-0.182059396299903	0.108239508	0.23528332899939	NUDT6	-0.101273741554879	0.205462541746375	-0.116712570081656	0.144179840450774	0.338467426379609
NUDT7	-0.302609051431957	0.00671525630149942	-0.291699123661149	0.009315038	0.0526831967993 08	NUDT7	-0.436923557767588	9.49274244290051e-09	-0.367439051475756	2.04259117532623e-06	1.15354051731 88e-07
NUDT9	-0.147414750923479	0.194816182576216	-0.153188899707887	0.177363734	0.455708441678928	NUDT9	-0.0198031163859334	0.804932204804199	0.0159912863310403	0.841931060137105	0.879088917983348
NUDT9P1	0.140342963158031	0.217341158744255	0.311735100633211	0.005163539	0.485440252698348	NUDT9P1	-0.0235296242455601	0.769174414367891	-0.0245938859845954	0.759047310597095	0.856084938547 42
NUF2	0.76773680367892	0	0.807838364167478	0	0	NUF2	0.826823347743162	0	0.420707149458369	3.71664162394466e-08	0
NUFIP1	-0.101437092640837	0.373724075411819	-0.10491723466407	0.356722475	0.638962202930062	NUFIP1	0.0800491979627 18	0.317398239109752	-0.0845079207160416	0.291103812731039	0.470169147590442
NUFIP2	-0.0537520598119216	0.638009240883561	-0.176752677702045	0.119052866	0.816681558876129	NUFIP2	-0.125764393659576	0.115362703053997	-0.179659957237997	0.0238976690188693	0.217407871718502
NUMA1	-0.236683900754655	0.0357200181336694	-0.197273612463486	0.081448391	0.160927225203589	NUMA1	-0.173528066756365	0.0292230658908931	-0.217821782178281	0.00597174133837196	0.0711635771232934
NUMB	0.152087249331524	0.180885665418 38	0.185881207400195	0.100937978	0.437601574982952	NUMB	-0.149295531596883	0.0611759618024363	-0.0589050505082084	0.462988823233595	0.130700876962151
NUP107	0.180542669758611	0.111331892174265	0.386976630963973	0.000469777	0.330864023610617	NUP107	0.493117783163168	4.63067362233005e-11	0.265316414102884	0.000754189262165202	8.89882060408102e-10
NUP133	0.22851181253537	0.0428089072726752	0.231937682570594	0.039925173	0.181708378800839	NUP133	0.170463078409428	0.0322440450850228	0.193836392166583	0.808984246374577	0.0771727600123856
NUP153	0.245848584479568	0.0289653207533989	0.159006815968841	0.161321772	0.139964845800323	NUP153	0.448420611138039	3.45059647521 85e-09	0.342350483031808	1.06813411474 05e-05	4.61332469257 27e-08

[illegible]

PCMT1	0.122030990916672	0.284008929040562	0.174415774099318	0.124071407	0.558529243170353	PCMT1	0.363635877329181	2.6488183402229e-06	0.22955358453575
PCMTD1	-0.45737871327133	2.25886579803958e-05	-0.557010710808179	1.673765941	0.000721742290042436	PCMTD1	-0.502505640765674	1.72537194175274e-11	-0.359888858210654
PCMTD2	-0.324383395149007	0.00353946214063205	-0.365043816942551	0.001011511	0.0331612895392803	PCMTD2	-0.340204108591137	1.22259598316507e-05	-0.128835331101712
PCNA	0.51201974722434	1.41296179601014e-06	0.430647517039922	8.722801874	6.83265486588962e-05	PCNA	0.654807102568039	0	0.457055016402902
PCNP	-0.0141680896392118	0.901372502150237	-0.0704722492697176	0.536371019	0.95318105905161	PCNP	0.170370373854565	0.0323394007633839	-0.0167899251321816
PCNT	0.312453062558713	0.00505616343632931	0.476022395326193	1.190815646	0.0432155576679866	PCNT	0.281154955076826	0.000345623681412333	0.0825972433611427
PCNX	-0.0247495529491912	0.82859359250261	0.18398247322298	0.104515637	0.918451161380744	PCNX	-0.317616376273054	4.77284762349486e-05	-0.0369813427528617
PCNXL2	-0.206978279054738	0.067217975520185	-0.248709834469328	0.027339428	0.241627975252312	PCNXL2	0.0663931445873265	0.40719647111606	0.114334872067558
PCNXL3	0.332616759811846	0.00274432342596675	0.460150925024343	2.461871387	0.0280792699010711	PCNXL3	0.193832898004361	0.0146756642236983	0.095022731127715
PCOLCE	0.14001741936579	0.218420528072607	0.212293086660175	0.060481822	0.486751684982538	PCOLCE	-0.200133737751044	0.0116946034347477	-0.323254358162344
PCOLCE2	-0.266158988632173	0.017744688308042	-0.185370009737098	0.101891602	0.102021641091993	PCOLCE2	0.222525424408602	0.00500551771968416	0.266572958286918
PCP2	-0.115942020326804	0.308903211993606	-0.103507944815571	0.363996480	0.581468328429575	PCP2	-0.0203355970197285	0.799796056701797	0.0354710213006341
PCP4	0.0382230649159118	0.738046662408785	0.0884702616993525	0.438145111	0.871936377105367	PCP4	0.057485571595331	0.473103597900843	-0.0400223013774183
PCP4L1	0.0694324885580355	0.543169318912625	0.0265463446690442	0.816360050	0.761666085612728	PCP4L1	0.157471915840763	0.0481524406419847	0.112643253262902
PCSK1	0.263285546305984	0.0190598490794542	-0.0276428702356022	0.808915604	0.10691245031549	PCSK1	0.0447860299098208	0.576324074811046	0.148170403341862
PCSK1N	-0.109748483614713	0.335632783052496	-0.0980282375851996	0.389305327	0.606623103111324	PCSK1N	0.278416959542954	0.000396865701809856	0.311123990942627
PCSK2	-0.0920350907186361	0.419838616577294	-0.184748408053261	0.103107681	0.677013302785627	PCSK2	-0.0928628810596599	0.245841488450332	-0.0533848729422532
PCSK4	-0.0298387620908948	0.794058923179055	-0.132838364167478	0.242645127	0.901106005810746	PCSK4	0.0284544699462058	0.722665240729033	0.0851544476872455
PCSK5	-0.138142914357464	0.224709096263154	-0.12332035053554	0.278288708	0.494360011778939	PCSK5	-0.152279193760773	0.0561234375722669	-0.0374696607711121
PCSK6	-0.0177211613063397	0.87681288343741	0.347809152872444	0.001785129	0.942580998403364	PCSK6	0.304349408503089	0.000101124242240402	0.272639663136708
PCSK7	0.301414130254656	0.00694619300403998	0.333592989289192	0.002789887	0.0537487500915112	PCSK7	-0.0268392159875973	0.737817265805603	-0.0562130290034345
PCSK9	0.011454591463803	0.920192597566425	-2.44104318795809e-05	0.999829646	0.961871423004532	PCSK9	0.0941539134257639	0.239309280412452	0.197776403591096
PCTP	-0.229901843808211	0.0415268297723468	-0.333739045764362	0.002777393	0.178586135950745	PCTP	0.0854208192346756	0.285904484956736	0.160268711821678
PCYOX1	-0.0560762674253666	0.623526232568131	0.0327896786757546	0.773788686	0.808503365090965	PCYOX1	-0.014942628441123	0.85217427762487	-0.0109270664333116
PCYOX1L	-0.0358062145173117	0.754067658073856	0.0179649464459591	0.874926569	0.880533096637808	PCYOX1L	0.358400309450151	3.76779316857423e-06	0.466725538652179
PCYT1B	-0.292377684382384	0.008930855206869	-0.380192882367244	0.000548020	0.0636520555969208	PCYT1B	-0.12958282597925	0.104643532625182	-0.0944385749997332
PCYT2	-0.0664187987820655	0.560852069581139	-0.0644839337877313	0.571615134	0.773537812564558	PCYT2	0.0458279694356981	0.567474309048863	-0.0247688285768035
PDAP1	0.304604634176305	0.00634463909789718	0.272711781888997	0.015278795	0.0505373628989974	PDAP1	0.249049864127593	0.00160209884035556	0.199618776983014
PDC	0.0771416451788023	0.499212610221217	0.0984989635671285	0.387793234	0.7338655422146	PDC	0.0591782156210819	0.460150055047661	0.0455550510109912
PDCD1	0.0752237365545945	0.509972408146292	-0.0273462247421005	0.810927924	0.740427516439279	PDCD1	0.00300626550694352	0.970095745359467	0.0427015296954496
PDCD10	0.21806452374187	0.0535314083063692	0.0731499513145083	0.520967827	0.20969802180566	PDCD10	-0.00241207351907707	0.976004374707382	-0.194382517302259
PDCD11	0.512168092984748	1.40143087623557e-06	0.427555988315482	9.898121676	6.7935048701439e-05	PDCD11	0.297504554016367	0.000146928685253567	0.248283090581543
PDCD1LG2	0.208072457141477	0.0657526688895467	-0.157912353413059	0.164552561	0.238135192326946	PDCD1LG2	-0.0535570804077836	0.503918817843408	-0.0561094563388595
PDCD2	0.0632337691861541	0.579831967558399	0.0475900681596884	0.676459637	0.785043582582832	PDCD2	-0.170178756080537	0.0325372569345068	-0.198533981077354
PDCD2L	0.208012785664904	0.065831908993345	0.152604673807205	0.179037103	0.238334888318451	PDCD2L	0.371484389084215	1.54359094350909e-06	0.236047757806501
PDCD5	-0.0221219172447841	0.846556815899122	0.0214946445959104	0.850597600	0.927295141632005	PDCD5	0.193839888249392	0.0146720197487076	0.236088831284672
PDCD6	0.0126343920092707	0.912003814693525	-0.00401655306718598	0.971994382	0.95829329577545	PDCD6	0.188259828847797	0.0178449036874271	0.212374221980914
PDCD6IP	-0.429328586105595	7.87556136050814e-05	-0.41769717624148	0.000147011	0.00193734891278769	PDCD6IP	-0.119802575045492	0.1337846393939	-0.17221957272939
PDCD7	0.0114883600510341	0.919958096399718	0.0234664070107108	0.837066189	0.96172795764013	PDCD7	0.102914829301958	0.198175467940916	-0.0209489951069378
PDCL	0.332655983179412	0.0027409536373022	0.303675754625122	0.006707807	0.0280738379277902	PDCL	0.618692665225535	0	0.350667101741389
PDCL2	0.0755405602200761	0.508186808523669	0.336624348220107	0.002418577	0.739251786830955	PDCL2	0.127508859670664	0.110364057333939	0.178255988365548
PDDC1	0.0396742443679758	0.728479804054927	0.010491723466407	0.926795363	0.866378446458109	PDDC1	-0.520102265019401	2.49543674402424e-12	-0.400667215834398
PDE10A	0.183994227032649	0.104546534035137	0.313315481986368	0.005101968	0.318013126752836	PDE10A	0.136818425227555	0.0864927486519891	0.0320707802515767
PDE11A	-0.216623876351946	0.0551691609757236	-0.24101752677702	0.032616013	0.213738622091648	PDE11A	0.182912948492668	0.0214274772728502	0.149378268766791
PDE12	-0.390585306461891	0.000372649046076736	-0.346957156767283	0.001834561	0.00659235494928952	PDE12	0.0862244751416002	0.28137908475085	-0.111505365793583
PDE1A	0.138029481998234	0.22509366553347	0.00545277507302824	0.961956327	0.495024139997741	PDE1A	-0.273080100389234	0.00051750078063906	-0.285731459993604

[illegible]

PM20D1	-0.0662886983313505	0.561621539011865	-0.235593259420721	0.036605191	0.773951421305509	PM20D1	0.0525838603292665	0.511711702113058	0.121128729604961
PM20D2	0.0555886381749073	0.626553269687208	-0.0512171372930867	0.653329294	0.809985657093509	PM20D2	0.115049859212559	0.150021449614951	0.0525831794979604
PMAIP1	0.0993030353328484	0.383911861954206	0.0988315481986368	0.385416589	0.647258766063917	PMAIP1	0.491235601255761	5.62405677584366e-11	0.522364889174019
PMCH	0.0337759675338219	0.767606898256702	0.166308905109019	0.142962569	0.887313471032732	PMCH	0.313355746371908	6.09793783432622e-05	0.23701696792311
PMCHL2	-0.0703090176443116	0.538077895983998	-0.0480373966795753	0.674191027	0.759408009870619	PMCHL2	-0.00319922835693627	0.968177261959507	-0.0560942439279662
PMEPA1	0.403207707697881	0.000229262307013034	0.0487585199610516	0.668974047	0.00452982008801577	PMEPA1	-0.35192553211922	5.77606446872497e-06	-0.312430736044425
PMFBP1	-0.0728670327712463	0.523354943817781	0.0168939684397508	0.882521121	0.748827746295455	PMFBP1	-0.0460798272281023	0.565344854980407	-0.0518468993707541
PML	0.0472362210349816	0.679325585469204	-0.11531158714703	0.310875390	0.838944828561343	PML	0.119990759123465	0.13317061737043	0.104977725244322
PMM1	0.152879362209733	0.178597844308377	0.119133398247122	0.295027658	0.434157837356328	PMM1	-0.106019179081323	0.184906430105659	-0.230314204501872
PMM2	0.243174814383779	0.0308143310088775	0.32736124634858	0.003372362	0.14625530086239	PMM2	0.365925579584646	2.26605905950805e-06	0.246123117083842
PMP2	0.076009673307049	0.505548831691446	-0.000647082305703335	0.995484221	0.737517292379727	PMP2	-0.0331922117993688	0.678858811636756	-0.0874072124694895
PMP22	0.231683778143736	0.0399300612670055	0.178188899707887	0.116047223	0.173416063183758	PMP22	-0.418827977105468	4.33356588228866e-08	-0.440481107341099
PMPCA	0.252319563485465	0.0248742282299255	0.244863680623174	0.029879237	0.126273738688775	PMPCA	0.251496668114871	0.00143480495235782	-0.0229585530574327
PMPCB	-0.0577253783014852	0.613335450340414	0.0143135345666991	0.900217380	0.803826687219714	PMPCB	-0.255596113939368	0.00118988007380755	-0.297857257493524
PMS1	0.560039397729268	8.03351540845654e-08	0.425949367088608	0.000105654	5.46002981953448e-06	PMS1	-0.00402601977927896	0.959959302339816	-0.10319795301799
PMVK	-0.017551728544322	0.877981592205011	-0.0102482960077897	0.928491272	0.943065542137329	PMVK	-0.309534721070315	7.57263374253146e-05	-0.36763957566544
PNCK	0.000615469518792541	0.995704834430735	0.286222195564246	0.010552171	0.998220547313688	PNCK	0.0579650078094096	0.46941450570336	0.138187444049706
PNKD	0.0811027990567511	0.477369768451525	0.0391431353456667	0.731474347	0.717651207088824	PNKD	-0.447272696705306	3.82395123858728e-09	-0.402652433945977
PNKP	0.0192772832826518	0.866091731176271	0.0816699123661149	0.473495335	0.937606028519692	PNKP	0.0556054103742265	0.487722075256083	0.0432747123525568
PNLDC1	-0.160409115310676	0.157895469737076	-0.196903369985611	0.081983547	0.408049095689999	PNLDC1	0.0463733797608228	0.562867682483247	0.00343346725437518
PNLIP	-0.0761085510067467	0.504993713294765	0.0502343904996169	0.660187697	0.737278209257956	PNLIP	0.124900426965799	0.117902761529252	-0.0863959109008666
PNLIPRP1	0.0122250519754814	0.914843976334589	0.0744804489655272	0.514174129	0.959424368415372	PNLIPRP1	0.0861473956959647	0.281811021313676	0.10950341404275
PNLIPRP2	0.0439969737401699	0.700232161500656	0.148785165102979	0.190652844	0.850896515738509	PNLIPRP2	0.343865032061953	9.70452308046710e-06	0.320554401102596
PNLIPRP3	-0.152430190332595	0.179892560804976	-0.123248270560004	0.279196599	0.436117605036764	PNLIPRP3	0.0936807650262018	0.241689039899057	-0.0684680189468579
PNMA1	-0.000750021444650064	0.994765851454097	0.0437195715676728	0.701478022	0.997776960073439	PNMA1	-0.406027010427356	1.20356653107927e-07	-0.298804989971312
PNMA2	0.259780992759379	0.0207761668978581	0.357010710808179	0.001323022	0.113198630552572	PNMA2	0.196818223815662	0.0131893120588455	0.0587442012235503
PNMA3	-0.0349228281848792	0.759949918552022	0.0955452775073028	0.401472925	0.883422288896803	PNMA3	0.019234344257637	0.810427628141011	0.0803397233016920
PNMAL1	-0.119981342512497	0.292235595645561	-0.0225413826679649	0.843408598	0.565708901361614	PNMAL1	-0.163017205705311	0.0407037980887495	0.00936475302289661
PNMAL2	0.0845493180481108	0.458790087024898	0.0710321324245375	0.533131777	0.704660891883554	PNMAL2	-0.467769820804258	5.75985929316997e-10	-0.462204413573549
PNMT	-0.0705796781711582	0.536510491358686	-0.201403361417061	0.075097849	0.758586252365749	PNMT	-0.132169617327327	0.0978382635395237	-0.0278949766375658
PNN	0.431460044542395	7.18975944180578e-05	0.357887049659202	0.001285243	0.00181607997752279	PNN	0.014597670651467	0.855549294063984	-0.136809670826537
PNO1	0.265536981509145	0.0180225677776535	0.289143135345667	0.009974820	0.1031992893765	PNO1	0.145135301264322	0.0688377951076089	0.0400131739378248
PNOC	0.0608281102719558	0.594360337331765	0.159139712359947	0.161254627	0.793630755586528	PNOC	-0.0818843111499808	0.306394929539697	-0.0441250854746814
PNP	0.385359387148873	0.000453122635787562	0.45842259006816	2.659222635	0.00761957474944964	PNP	0.349198821635982	6.89535063602698e-06	0.298263428555433
PNPLA1	-0.117313282940379	0.303177193030317	0.00771683828948892	0.946186451	0.576062880560439	PNPLA1	0.231831560512106	0.00337896899725632	0.14156680345174
PNPLA2	-0.101965477913825	0.371227183053963	-0.0998052580331061	0.380734451	0.6373486022046	PNPLA2	-0.0822563702010142	0.304194967177149	-0.0408879179990203
PNPLA3	0.153177589841819	0.177741979885453	0.269588633183132	0.016277694	0.433469952416185	PNPLA3	0.180775009428153	0.0230247180643226	0.177619974488858
PNPLA4	-0.345391177869684	0.00182385028030301	-0.324050632911392	0.003724203	0.0209599714374392	PNPLA4	-0.462971193644949	9.07349280943250e-10	-0.187466960570276
PNPLA5	-0.0377093818937521	0.741442743990048	-0.0145203101886164	0.898933351	0.873302184237688	PNPLA5	0.107183938630537	0.180099657749798	0.0514726743474220
PNPLA6	-0.0770737822478201	0.499591304490429	0.0131207400194742	0.908501991	0.734203954540920	PNPLA6	-0.146612178054733	0.0660330349098456	-0.00193501719381489
PNPLA7	-0.161781183676842	0.154323374686826	-0.183666017526777	0.105121441	0.403038122878126	PNPLA7	-0.208100189108128	0.00869554286086558	-0.218801460694646
PNPLA8	-0.222189635430593	0.0490608571425580	-0.137706913339825	0.225691671	0.198693856448261	PNPLA8	-0.262895491352271	0.000846250082780516	-0.179818166190951
PNPO	-0.447833006797309	3.49807673583852e-05	-0.531231742940604	7.260242611	0.00102193444137983	PNPO	-0.292079515921704	0.000196273811484663	-0.393328754401251
PNPT1	0.401128489633457	0.000248692890383984	0.32134858812074	0.004035333	0.00485073765879136	PNPT1	0.117195140854168	0.142517183495089	-0.0713491953021275
PNRC1	-0.185080851917973	0.102478356842094	-0.269912366114898	0.016392250	0.313603116451019	PNRC1	-0.346616629602959	8.14229162353136e-06	-0.250612108917809
POC1A	0.550548349310409	1.46763778463566e-07	0.678943524829601	0	9.33342125547398e-06	POC1A	0.667775991975146	0	0.490791554380286
POC5	0.040506910566395	0.723009031652016	0.168427458617332	0.137673107	0.863089849590389	POC5	0.490868420228895	5.84057247010605e-11	0.527030532046212
PODN	0.128810649072922	0.257901040099346	0.0813291139240506	0.475348102	0.531665136446516	PODN	-0.0837053237491966	0.29572662858975	-0.12921411984484
PODNL1	0.239979892123596	0.0331530490753196	0.250778967867575	0.026050646	0.152844057019038	PODNL1	0.081165577510663	0.310674253639944	0.147329493205364
PODXL	0.126895082301003	0.265106413571405	0.00460077896786758	0.967910489	0.538102898975292	PODXL	-0.471361956759413	4.07999836317540e-10	-0.393751958597903

[illegible]

PSMB8	-0.0214780679094164	0.850970665304353	-0.124805258033106	0.272508293	0.929553618600988	PSMB8	-0.085492682498727	0.285497849927229	-0.289495001585976
PSMB9	0.352470030079016	0.00144359310589515	-0.00352969814995131	0.975398183	0.0176875066285704	PSMB9	0.0797673785801244	0.31911047143216	-0.264885903202058
PSMC1	0.0886008609870203	0.437466604915219	0.108933787731256	0.338531325	0.688749406889569	PSMC1	0.352622266731998	5.51901165879443e-06	0.204015008553231
PSMC2	0.0564245633507125	0.621367935765422	0.0958373904576436	0.400029881	0.807876349827013	PSMC2	0.0282704726184712	0.724385945302251	0.179694945756439
PSMC3	0.254827806643535	0.0234260832955053	0.198977604673807	0.078811208	0.121670450477548	PSMC3	0.148209455708815	0.0631053222900917	-0.0990068585102632
PSMC3IP	0.681336758398244	4.82813788948988e-12	0.571786757546251	6.793822949	7.02139052781845e-10	PSMC3IP	0.590615629688305	4.44089209850063e-16	0.295824880944045
PSMC4	0.140288108105915	0.217522771580138	0.124245374878286	0.274678214	0.485626524022121	PSMC4	0.496360371783551	3.30384608560053e-11	0.411979155970568
PSMC5	0.0138501517872435	0.903575027819484	-0.188096397273612	0.096886307	0.954285625044789	PSMC5	-0.397948179463744	2.24410286788779e-07	-0.478884809430609
PSMC6	0.137357604474962	0.227380979130021	0.255769230769231	0.023153484	0.497847625527856	PSMC6	0.145145569933365	0.0688179642279683	0.0402641785266451
PSMD1	0.308056433350184	0.00574602065727081	0.329333008763389	0.003177253	0.0473717367901218	PSMD1	-0.159588769607998	0.045185103828153	-0.161695634878123
PSMD10	-0.192814887295304	0.0886662731777867	-0.181158714703019	0.110018789	0.286683268090611	PSMD10	0.233070715753771	0.00320781028815431	0.311881568428885
PSMD11	0.202608160751109	0.0733351391317281	0.239970788704966	0.033396426	0.255401018092502	PSMD11	0.0987163947382789	0.217199327118723	-0.0743429954887845
PSMD12	0.097054272497794	0.39482560282122	-0.0197906523855891	0.862326588	0.657145566994706	PSMD12	-0.142851541347249	0.0733650840319444	-0.188875628747534
PSMD13	0.225352277404341	0.0458454032909716	0.0840068159688413	0.460896682	0.190010558736135	PSMD13	-0.246820302735434	0.00176982543233098	-0.266067906629412
PSMD14	0.210833099183564	0.0621701505189334	0.227020447906524	0.044424366	0.230435014423438	PSMD14	0.266857226714835	0.000700490904746776	0.162147443138
PSMD2	0.255157374632741	0.0232412471735246	0.252604673807205	0.024956793	0.121020038965164	PSMD2	-0.0229908717590921	0.774316002865328	-0.169066042349847
PSMD3	-0.0024561789207222	0.982860321660701	-0.0291626095423564	0.798254697	0.992371824561201	PSMD3	0.14601988550404	0.0671464570478493	-0.055481141568886
PSMD4	0.246250049009744	0.0286959472629118	0.0958373904576436	0.400029881	0.139207169380885	PSMD4	0.0641823652991897	0.423027230925777	-0.169624337404981
PSMD5	0.191899019844584	0.0902207193540916	0.137852969814995	0.225196278	0.289777283566207	PSMD5	0.0440624979083372	0.582507117057126	-0.0179567161602131
PSMD6	-0.205051511816651	0.0698622402777786	-0.168841285296982	0.136698171	0.247978353950809	PSMD6	-0.0280694548245028	0.72626739887899	-0.243517045873821
PSMD7	0.0934754486302127	0.412568842057439	0.111976630963973	0.325148151	0.671117962435743	PSMD7	0.320950919709661	3.92982484729298e-05	0.33562812377122
PSMD8	0.129834924486136	0.254103388423289	0.194547224926972	0.085814117	0.527960585800589	PSMD8	0.630780373197618	0	0.527649676698636
PSMD9	0.277389439251333	0.0133247578913276	0.207351509250243	0.066829490	0.0845754369623483	PSMD9	0.032449959505124	0.685655762672473	0.0231289319298441
PSME1	0.0421815079014225	0.712048787905959	0.0211295034079844	0.853108305	0.857967286700119	PSME1	-0.0458712954223143	0.567107717683302	-0.109667874786582
PSME2	0.128329456713033	0.259698386579893	0.179406037000974	0.113546344	0.533690057036264	PSME2	0.11833756526763	0.138639199108546	-0.0201138496831255
PSME3	-0.0256467377770622	0.822479778192676	-0.0361246348588121	0.751486227	0.915625636220576	PSME3	0.199068751134875	0.0121576269536441	0.0111841559818609
PSME4	0.142155870922456	0.211399073913938	0.294255111976631	0.008693902	0.47805292520808	PSME4	0.264419514147613	0.000787164456693912	0.030030797502437
PSMF1	-0.188127012844443	0.096850487411919	-0.236806231742941	0.035852119	0.303422927298105	PSMF1	0.161725009110527	0.04234694706046	0.121740268057724
PSMG1	0.240128419353091	0.033041116827085	0.153943524829601	0.175219319	0.152612612939301	PSMG1	-0.0585013421342059	0.465306338516751	-0.232066672903817
PSMG2	-0.00348717841198439	0.975667676999658	0.0107108081791626	0.925269325	0.98907669380924	PSMG2	-0.114006932221794	0.153775742971245	-0.139158465281922
PSMG3	0.329941971093105	0.00298306294883699	0.279552093476144	0.012829923	0.0298427005574598	PSMG3	0.464793146518121	7.64185381640914e-10	0.33887444978663
PSMG4	0.149151766741434	0.189550071586994	-0.0321324245374878	0.778206497	0.449569978749173	PSMG4	-0.0203403705266838	0.799750051293171	-0.0487101026304661
PSORS1C1	-0.0234646333528432	0.837367272809485	0.0612457735858684	0.591826291	0.92295589194106	PSORS1C1	0.042893962283103	0.592557231359896	0.135588218292011
PSORS1C2	-0.0920174451617912	0.419928132366335	0.0047640055940417	0.966762795	0.677015178509019	PSORS1C2	0.066888241112636	0.403699791350522	0.118379939089507
PSORS1C3	-0.0372652913748732	0.744382720077257	0.109313803005504	0.337561992	0.874667385794201	PSORS1C3	-0.219987516892983	0.00548039867297935	-0.139722845296785
PSPH	0.296785068104175	0.0079081793705531	0.357254138266797	0.001312428	0.0586896703905438	PSPH	0.301968380515822	0.000115279519049771	0.196819543673714
PSPN	0.144344606130505	0.204379744298126	0.266161805446066	0.017743438	0.468446237423611	PSPN	0.0305037602672741	0.703596302394856	-0.00104661307338416
PSRC1	0.789564476425215	0	0.773904576436222	0	0	PSRC1	0.767185202545602	0	0.553526388969177
PSTK	-0.0944387103282511	0.407748156485825	-0.0637536514118793	0.575986299	0.667695504051925	PSTK	0.328086179206657	2.5727824610744e-05	0.26526621318512
PSTPIP1	0.109740060573461	0.335670100134004	0.0408714703018501	0.720095479	0.606623103111324	PSTPIP1	0.103874142112975	0.194003424465161	0.0954136897903018
PSTPIP2	-0.0777361409635233	0.495901557749452	-0.124561830574489	0.273450313	0.731582151920195	PSTPIP2	-0.204042344555726	0.0101253341539682	-0.28406048186905

[illegible]

RER1	0.222525060874135	0.0487112690542626	0.211489776046738	0.061478849	0.197825765781356	RER1	-0.330780555876167	2.18640000683166e-05	-0.315594915103494	[illegible]
RERE	-0.0582249933694395	0.610262333246847	0.0157740993184031	0.890087618	0.801991113672318	RERE	-0.300009768855453	0.000128288915754414	-0.393147726849314	[illegible]
RERG	-0.328813849716737	0.00308920396486693	-0.299283710349446	0.007375319	0.0305644202187019	RERG	-0.591477856194454	2.77356993850304e-16	-0.383868163262623	[illegible]
RERGL	0.045514875209193	0.690406514624021	-0.0510626621997716	0.654938225	0.845397476859453	RERGL	-0.214527357155451	0.00679467771720443	-0.233533325828544	[illegible]
REST	-0.066655386510312	0.55945407276275	0.0803067185978578	0.480929840	0.772839444790286	REST	0.228383155769629	0.00389947074516384	0.265421379658209	[illegible]
RET	0.394995908240947	0.000315162090317678	0.0548567116411792	0.631108406	0.00577690066941894	RET	-0.069929357859743	0.38261495195436	-0.127300400010077	[illegible]
RETN	0.281871228673799	0.0118477654573221	0.119699968054462	0.293377073	0.0771976955718862	RETN	0.01045222900366	0.896295205676917	0.0755586960806634	[illegible]
RETNLB	0.274063345632396	0.0145216327382283	0.173787714252897	0.125591668	0.0888091689229064	RETNLB	0.080246579573946	0.316202578769062	0.123054619359183	[illegible]
RETSAT	-0.0754363078286097	0.508774013762019	-0.105355404089581	0.354709012	0.739541064885562	RETSAT	-0.369497164927312	1.77213579054888e-06	-0.439960843284271	[illegible]
REV1	0.0632256504165334	0.579880723055669	0.158057448880234	0.163863059	0.785055851912892	REV1	0.0246338738522099	0.758667586042897	0.0241314290451931	[illegible]
REV3L	0.0593777698840787	0.603197353072255	0.0748782862706913	0.511146355	0.798423052406844	REV3L	0.0343189658952799	0.668590306185812	0.0590180244113543	[illegible]
REXO2	0.266656186279823	0.0175252215816548	0.251338851022395	0.025710940	0.101090036455598	REXO2	0.00428491330471886	0.957386834650927	-0.0627420197654886	[illegible]
REXO4	0.410601565726525	0.000170925973240976	0.352117818889971	0.001553114	0.00358871963774949	REXO4	0.499891087916108	2.27826646437279e-11	0.0892511468247797	[illegible]
RFC1	-0.0463225025136266	0.685199247453097	-0.0578627069133398	0.611802337	0.842050634907412	RFC1	-0.00236594346857511	0.976463149392966	-0.0899433109939509	[illegible]
RFC2	0.643462010074814	1.59998236881 62e-10	0.628627069133398	0	1.82063546461109e-08	RFC2	0.688525481455625	0	0.349888226689608	[illegible]
RFC3	0.420816545916306	0.000112639589204377	0.506158714703019	2.730545477	0.00255773340445944	RFC3	0.371757516305029	1.51446539731204e-06	0.116662369163892	[illegible]
RFC4	0.697783442077124	8.92619311798626e-13	0.602969814995131	7.452983854	1.48354829821456e-10	RFC4	0.756348012402225	0	0.490504040033092	[illegible]
RFC5	0.441930795767336	4.55479078331766e-05	0.521324245374878	1.239302472	0.00126345935641594	RFC5	0.678323079047 61	0	0.509112234125089	[illegible]
RFESD	-0.215834860039219	0.0560833403086556	-0.176071080817916	0.120500139	0.215834671869614	RFESD	0.366051456906249	2.24662085046567e-06	0.267330535773175	[illegible]
RFK	-0.0715384350242648	0.530976477854515	-0.0102969814995131	0.928152065	0.754429078951623	RFK	0.0855540471882184	0.285150927235527	0.0130811421773697	[illegible]
RFNG	-0.0989444858041617	0.385639745073526	-0.118427458617332	0.297914051	0.649005545087138	RFNG	-0.324679610050708	3.15362519980226e-05	-0.34460065900164	[illegible]
RFPL1	0.151941590612826	0.181308676031813	0.222842366166926	0.048382455	0.438055582037283	RFPL1	0.0154753506108757	0.846967485750303	-0.00904379203386726	[illegible]
RFPL2	0.219056303669127	0.0524272350640462	0.153262996716338	0.177497428	0.206843378235928	RFPL2	0.154367411823459	0.0527960513 24248	0.0875489459320492	[illegible]
RFPL3	-0.0518146407854676	0.650186718279206	0.0289307438976627	0.800193723	0.823053821948677	RFPL3	-0.0131795631543233	0.869450708014568	0.0405714998524396	[illegible]
RFT1	0.0544757575363732	0.633484732113256	0.0493184031158715	0.665398602	0.814083829590922	RFT1	0.0997078821326318	0.212593515848243	-0.0530197753585145	[illegible]
RFTN1	0.207143538664557	0.0669949881560012	0.0567429406037001	0.618718712	0.241133189399343	RFTN1	-0.492887945362827	4.74218483640963e-11	-0.45445825983856	[illegible]
RFTN2	0.17329180581842	0.126691101277143	0.00842259006815969	0.941220272	0.360196099465502	RFTN2	-0.108126718668913	0.176276792856636	-0.0876994820938904	[illegible]
RFWD2	0.238897356085385	0.0339785109991748	0.17064264849075	0.132515379	0.155453849304113	RFWD2	0.237805731452407	0.00262350492013352	-0.133683522765775	[illegible]
RFWD3	0.6308561088016	4.61774840587736e-10	0.583154819863681	3.247197698	4.80683305112855e-08	RFWD3	0.684881153417353	0	0.480750215255614	[illegible]
RFX1	0.0629102367723587	0.581776327943923	0.0867088607594937	0.446562599	0.786545369750148	RFX1	0.160623500636173	0.0437911537088167	0.181838511130921	[illegible]
RFX2	-0.119793649432213	0.292996702592043	-0.10350535540409	0.363258433	0.565708901361614	RFX2	0.261807642103362	0.000890887794156914	0.356959221611359	[illegible]
RFX3	0.245388685884181	0.0292765213689086	0.159336412712376	0.160730673	0.141020711065337	RFX3	-0.0333694279316756	0.677239800975847	-0.0496989086586425	[illegible]
RFX4	-0.130688469660955	0.250968028171798	-0.0670721110412264	0.556995686	0.524531930802264	RFX4	0.0429285275291139	0.592258821996127	0.0776912445796544	[illegible]
RFX5	0.276051853455709	0.0137953920739713	0.29101752677702	0.009487120	0.0864528721289621	RFX5	0.132107353846725	0.0979978472455141	-0.165189923002489	[illegible]
RFX6	0.119774995787253	0.293072415462566	0.3605221604255	0.001099226	0.565708901361614	RFX6	0.00134865900088353	0.986581985360518	-0.0481236360976005	[illegible]
RFX7	0.00544191300455884	0.962036498098636	0.0719571567672834	0.527801430	0.982641742128921	RFX7	0.224673254043463	0.00453899396031132	0.0176783292526123	[illegible]
RFXANK	0.132560376315855	0.244184858060307	0.209225900681597	0.064360949	0.517083844599251	RFXANK	0.607087949210383	0	0.467301989104542	[illegible]
RFXAP	0.0363377710216787	0.750534854488089	-0.0210564751703992	0.853610623	0.87839980780315	RFXAP	0.113127215618808	0.156997148373567	0.00617167040511558	[illegible]
RGAG1	-0.155600889342828	0.170898250984349	-0.158204468141124	0.163763165	0.424173299834668	RGAG1	0.0418263583774527	0.601807512583671	0.0707726453678068	[illegible]
RGAG4	-0.209500728395896	0.0638789020126805	-0.169717624148004	0.134650934	0.234031510733268	RGAG4	0.139159707489106	0.0812911476349672	0.215968911940807	[illegible]
RGL1	-0.101180704221077	0.374939305232195	-0.021713729308666	0.849091892	0.640265030122807	RGL1	-0.4469087762293	3.95033613426883e-09	-0.421202436479765	[illegible]
[illegible]	[illegible]	[illegible]	[illegible]	[illegible]	[illegible]	[illegible]	[illegible]	[illegible]	[illegible]	[illegible]

S1PR2	0.17026770772751	0.1335551537955	0.231645569620253	0.040181377	0.372035750953155	S1PR2	0.110527809423968	0.166811747861986	0.131279963672828
S1PR4	0.0565408949574485	0.620647769571735	-0.228724440116845	0.042819768	0.807831217291121	S1PR4	-0.161524577080669	0.0426067265791195	-0.0988775531160224
S1PR5	0.379547059132802	0.000561080592430319	0.280817916260954	0.012416356	0.00893548023014583	S1PR5	0.0444382085519515	0.579292602228567	0.0897044763245886
SAAL1	0.408704913058168	0.000184417905320977	0.266601752677702	0.017799011	0.00380335515697625	SAAL1	0.33834884560115	1.37289151533881e-05	0.173853516653026
SAC3D1	0.267626709386361	0.0171035398545902	0.25167964946446	0.025506015	0.0992878811987335	SAC3D1	0.186065625604123	0.0192467922201858	0.00673149182028666
SACM1L	-0.298100001122443	0.00762368797877029	-0.276460564751704	0.013890785	0.0573531003591171	SACM1L	-0.162175460394433	0.0417679572257	-0.285784697391232
SACS	0.136899110040353	0.229638444274975	0.229625121713729	0.041991307	0.500327812536695	SACS	0.49942687587303	2.39295250281657e-11	0.240044237690878
SAE1	0.614123693087036	1.75464975882278e-09	0.585759493670886	2.72096996	1.66045277177019e-07	SAE1	0.663244108171776	0	0.550364984602635
SAFB	0.184470485504024	0.103636105048917	0.164654333008763	0.146807232	0.316314025564426	SAFB	0.370008872679608	1.71038649776634e-06	0.210293165753603
SAFB2	0.0401420162541108	0.725404766088628	0.0594206426484908	0.602236110	0.864662923580505	SAFB2	-0.0944404686415772	0.23787599819829	-0.136986134658677
SAG	0.0913645738433215	0.423247866336402	0.209973687080015	0.063268036	0.679426848919923	SAG	0.162012688500474	0.0419764064390049	0.209102034886656
SAGE1	-0.0636640279994898	0.577250843128347	-0.14418756450104	0.204877775	0.78358731471465	SAGE1	0.478967838844989	1.93997928832346e-10	0.202095664912907
SALL1	-0.0506706836905226	0.657420466839495	0.00390568910073294	0.972748647	0.826813048019301	SALL1	0.173158230468222	0.0295742956696077	0.00237009361717664
SALL2	-0.0537911593785033	0.637764453002315	0.215798442064265	0.056284467	0.816681558876129	SALL2	0.0939414824215815	0.24037569658321	0.0123387770904344
SALL3	-0.0678914802652844	0.552177076375265	0.11933885954951	0.326039209	0.76778390161347	SALL3	0.0482593953813426	0.547077292853048	-0.0616786730528497
SALL4	0.0859099400666232	0.451566104609677	0.0481745843472317	0.673313303	0.69925599278461	SALL4	0.160016523871201	0.0446044217215253	0.245242131956986
SAMD1	0.459464889584215	2.04936956231805e-05	0.47901655306186	1.034472566	0.000666885516446233	SAMD1	0.223953717673609	0.00467340754597911	0.143080221827248
SAMD10	-0.0493235358168086	0.665979551090633	-0.15496592015794	0.172344468	0.831444486900047	SAMD10	0.0374564811208367	0.640323732082955	0.207687281749669
SAMD11	-0.0568295659158807	0.618862254151404	0.0533252273949837	0.640683992	0.807059057273274	SAMD11	0.110802253902569	0.16575446041581	0.106305848563492
SAMD12	-0.32839855626252	0.00312911955623519	-0.466081220074478	1.498723722	0.0308820990934229	SAMD12	-0.272641337530355	0.000528791627653361	-0.356992417379661
SAMD13	-0.162966035977686	0.15128742176114	-0.237025316455696	0.035677357	0.398286177295327	SAMD13	-0.338489713045841	1.36089389596427e-05	-0.40510163023689
SAMD14	-0.0784042822033959	0.492194094324984	-0.20126582278481	0.075377968	0.72911609116622	SAMD14	0.18465060834332	0.0202005753984036	0.161262204156639
SAMD3	-0.0286632292486885	0.802003445376808	-0.327928915463391	0.003174813	0.905418353939295	SAMD3	-0.17333058301673	0.0294101693517571	-0.221635530688418
SAMD4A	0.0041080965898912	0.971336814570119	-0.0321324245374878	0.778206497	0.986997119047726	SAMD4A	-0.279641697534729	0.000373132462526418	-0.227083263609783
SAMD4B	0.0619019605848381	0.58785493078903	0.17991723466407	0.112508528	0.790064883198249	SAMD4B	-0.0479637899611664	0.549537790261748	-0.0495270084740814
SAMD5	0.239799913709463	0.0332891095349956	0.243281402142162	0.030980632	0.153256985191607	SAMD5	0.36985085876699	1.72923253849433e-06	0.12497746663354
SAMD8	-0.0843975976441689	0.459599535847676	-0.125194741966894	0.271005617	0.705072439243394	SAMD8	-0.176159643132474	0.0268250501283793	-0.0919178804260042
SAMD9	-0.0555450258097403	0.626824302135679	-0.0379503407984421	0.739361626	0.810183129178019	SAMD9	0.206665924968108	0.00917906845022554	0.158571008057293
SAMD9L	-0.0681328027099574	0.550761723441637	-0.0783106134371957	0.491928121	0.766778812201304	SAMD9L	0.103139903937908	0.197190833484643	0.0966991375330484
SAMHD1	-0.0904629452757371	0.427857156457785	0.00481986368062317	0.966379237	0.682550782552894	SAMHD1	-0.173179048637986	0.0295544297982601	-0.303285041571772
SAP130	0.220991683382862	0.050326280504164	0.247492697176241	0.028122546	0.20189719590494	SAP130	0.248092075937987	0.00167229170780078	0.0963097001103938
SAP18	-0.280549883777971	0.0122676574700686	-0.315676728334956	0.004765033	0.0795908691026172	SAP18	-0.100948037259452	0.206931443190033	0.0216974451536021
SAP30	0.307141582069327	0.00589960400546663	0.260540408958131	0.020645706	0.0481760396449707	SAP30	0.407374609067609	1.08301950740852e-07	0.12423403667871
SAP30BP	0.17326323180895	0.126754673183532	0.0207400194741967	0.855788007	0.360246289154174	SAP30BP	0.0104139509821448	0.896672878406487	-0.0591290749264081
SAP30L	-0.138350412881816	0.224006806342024	-0.0474683544303797	0.677241216	0.493693627795025	SAP30L	-0.0481130695797175	0.548294573260523	-0.0188801087990854
SAR1A	0.363390231801012	0.000995830116617302	0.348855890944499	0.001726050	0.0138020518895985	SAR1A	0.114402067625947	0.152345111216546	0.0425475596664944
SAR1B	-0.000144672635265199	0.998990371281474	0.0618549172346641	0.587423485	0.999344067735825	SAR1B	0.211042613234215	0.00777358185892707	0.221612712089434
SARDH	-0.0124014283424012	0.913620075143384	-0.0935004868549172	0.411660134	0.959062038981856	SARDH	-0.0377667634825299	0.637555366472313	0.00778418473329466
SARNP	0.215012719151634	0.0570490168301201	0.185077896786758	0.102439693	0.21786357498863	SARNP	0.247239664620995	0.00173710624438472	0.208618280588202
SARS	0.100767473809404	0.376902966819516	0.12738558909445	0.262657385	0.641793674516825	SARS	0.325230967638341	3.05191711738804e-05	0.173953918564922
SART1	0.232245385101221	0.0394375235821605	0.153067185978578	0.177711403	0.172070448137651	SART1	0.0997017781118536	0.212621655806093	0.011719632438011
SART3	0.0833293678959034	0.465320820286425	0.201192794547225	0.075485650	0.709350638478874	SART3	0.379484131365108	8.77147815714352e-07	0.365277090050664
SASH1	-0.178052234074276	0.11643583689211	-0.312025316455696	0.005294883	0.339706148702191	SASH1	-0.32032934995806	4.07550924625042e-05	-0.233799365186644
SASH3	0.0437060485308421	0.702121062495192	-0.23159688412853	0.040224210	0.851831086616974	SASH3	-0.410815710094246	8.25428806629628e-08	-0.497955073221225
SASS6	0.58199919024501	1.84913799827768e-08	0.429065238558909	9.307101933	1.43985241456441e-06	SASS6	0.421061215462508	3.61026155637489e-08	0.105332174148535
SAT1	0.0969724341322976	0.395226219418786	-0.161587147030185	0.154562959	0.657590995849647	SAT1	-0.500142107245152	2.21850743978162e-11	-0.323697038982628
SAT2	-0.168636026602884	0.137374011125857	-0.299050632911392	0.007626202	0.378146582052498	SAT2	-0.467484247418939	5.91892510255257e-10	-0.519587500266217
SATB1	-0.308204560969045	0.00572148860593964	-0.363583252190847	0.001062629	0.0471891583187132	SATB1	-0.511236262321266	6.70274532090477e-12	-0.302992963504781
SATB2	-0.102515663503635	0.368638086085635	0.00730282375851996	0.949034925	0.635625812295562	SATB2	-0.128916505887018	0.106455415247858	0.00453329499808835
SAV1	-0.0695913219783906	0.542244974249931	-0.01550632911392	0.891943467	0.761662825997706	SAV1	0.31712753336357	4.90984529628591e-05	0.123126117635998
[illegible]									

SIX5	0.155378238366218	0.171518854716477	0.209469328140214	0.064045870	0.424887263099008	SIX5	0.1990139604646	0.0121818774535778	0.301050340111305	0.000121215768170801	0.0339430919714331	SIX5	0.120689381297871	0.338236511843855	0.192613636363636	0.124091179107528	0.420214384855845
SIX6	0.109895251233659	0.334982970444411	0.112101854622079	0.325309158	0.60587783802458	SIX6	0.0118517113996679	0.882503775346419	0.0328420988579369	0.682061683812036	0.928835112456993	SIX6	0.0139699006446426	0.912053354019411	-0.108397530621275	0.390060127645205	0.930999434492898
SKA1	0.252764388699398	0.0246119983896058	0.676557935735151	0	0.125393123170949	SKA1	0.722832816319921	0	0.230354107163977	0.00359377758806184	0	SKA1	0.760617209809751	1.97175609173428e-13	0.788723776223776	0	1.86011694845482e-12
SKA2	0.543815189756733	2.22549707995512e-07	0.490968841285297	5.827172004	1.35433480761084e-05	SKA2	0.568487515810897	6.66133814775094e-15	0.398181509785111	2.20459229116435e-07	2.51817731622371e-13	SKA2	0.821607224139386	0	0.740952797202797	0	0
SKA3	0.754659420935415	8.88178419700125e-16	0.76110029211295	0	2.92773213080485e-13	SKA3	0.668601466208909	0	0.420151576462141	3.88965663859704e-08	0	SKA3	0.731333848704316	4.57101023698869e-12	0.645629370629371	1.46451310825029e-08	4.18271817813559e-11
SKAP1	-0.203998818759483	0.0713419199797779	-0.301243313316881	0.006979770	0.251112385363952	SKAP1	-0.247028382681841	0.00175352126426885	-0.289413294896696	0.000225816820533789	0.00627816539774203	SKAP1	-0.336547669622581	0.00612258820228312	-0.456424825174825	0.00015655233501298	0.0140925405252313
SKAP2	-0.239679865595796	0.0333801239247821	-0.146153846153846	0.198288132	0.153604488363039	SKAP2	-0.0290557674733483	0.71705175235266	0.0280607917901804	0.726348518600459	0.817546464409022	SKAP2	-0.230055908473369	0.0652422268841405	-0.118881118881119	0.344708049033974	0.106326787322264
SKI	0.269287045404381	0.0164023643993489	0.0780185004868549	0.493548704	0.0965206673877782	SKI	-0.288398371064441	0.000238111000035242	-0.341828697735049	1.10388865001263e-05	0.00107547191277921	SKI	-0.410121423259157	0.000691501077911231	-0.482998251748252	5.70623910723002e-05	0.00214128119619772
SKIL	0.0253486739839006	0.824509763938046	0.0570837390457644	0.616610166	0.9165441834064	SKIL	-0.180808088369267	0.0229992457843262	-0.18965906731264	0.0169979407445462	0.0581615542430557	SKIL	-0.0498784290829602	0.693155953240576	-0.00913461538461539	0.94240068350266	0.748868399358667
SKIV2L	-0.0151394977349705	0.89464771818561	-0.0620009737098345	0.586540035	0.950031815853691	SKIV2L	-0.144679889843172	0.0697219737128035	-0.12444199017739	0.119268097650756	0.145333054353794	SKIV2L	-0.0846059067806126	0.502805278802285	-0.139685314685315	0.266383637799333	0.57731249105453
SKIV2L2	-0.153010220696057	0.178221932547871	-0.092064264849075	0.418904907	0.433942584888295	SKIV2L2	0.0927200269666045	0.246571819280404	0.0714161298591462	0.3725547486678	0.387782948313022	SKIV2L2	0.452558534573257	0.000153729263710822	0.440821678321678	0.000273484027177897	0.000578169819347619
SKP1	-0.304504603639471	0.00636277781768218	-0.253821811100292	0.024249520	0.0506410541964258	SKP1	0.157693573412772	0.0478343174497295	0.115375400181213	0.148863872119396	0.107184735181295	SKP1	-0.0278904679885183	0.825450629699263	0.117788461538462	0.34918495617875	0.860293249205064
SKP2	0.260336174882138	0.0204957749235137	0.472176241480039	1.424298694	0.112103273351011	SKP2	0.735875652299905	0	0.41331632587101	6.76280232397063e-08	0	SKP2	0.712623563304762	2.77324829767167e-11	0.781075174825175	0	2.47508402982951e-10
SLA	0.0979292183460067	0.390557616947544	-0.268281402142162	0.017072785	0.653396087632256	SLA	-0.540658108318861	2.25444895126143e-13	-0.534466352835023	4.73146800452044e-13	6.64070483083567e-12	SLA	-0.501506629152531	2.08495857540794e-05	-0.532561188811189	7.00062031223484e-06	9.85059987891634e-05
SLA2	0.0816181466763826	0.474566199666817	-0.186976630963973	0.098918092	0.715541579299755	SLA2	0.162331971310094	0.0415683449928694	0.0998344130334041	0.212010799449721	0.0953968126799708	SLA2	-0.340538243662908	0.00550792122269485	-0.386232517482517	0.00159909335902913	0.0128708965308862
SLAIN1	-0.273241283700068	0.0148313846886371	-0.28777994157741	0.010343188	0.0900134713557927	SLAIN1	-0.139255874664085	0.0809791009986651	-0.0902475589803998	0.259451041783667	0.164133573641465	SLAIN1	-0.364168987667945	0.00286196885463598	-0.282255244755245	0.0230418913203904	0.0072942883980818
SLAIN2	-0.139488659768415	0.220181714480955	-0.132181110029211	0.244999693	0.489518204699228	SLAIN2	0.211808811159555	0.0075482142019121	0.232361793450673	0.00330474709515275	0.0224097118944044	SLAIN2	0.254148026197695	0.0410597514571212	0.305113636363636	0.0137560645956787	0.0718452586693055
SLAMF1	-0.00954394534911372	0.933471291846308	-0.173653660493969	0.125888145	0.967780170495177	SLAMF1	0.0239409474620846	0.765255691074895	0.0949451478911706	0.235366330502586	0.85316764202076	SLAMF1	-0.523254256884317	7.74387659587274e-06	-0.515472027972028	1.49196341299796e-05	4.01585718675353e-05
SLAMF6	0.0244247067889341	0.830809770752425	-0.185967538754288	0.100814483	0.919902226122034	SLAMF6	-0.0775224052547476	0.332963454810297	-0.109083717763958	0.172457882497188	0.486689734264197	SLAMF6	-0.327219415244282	0.00779976625682676	-0.277447552447552	0.0255617824983534	0.0173895443307766
SLAMF7	0.0886404309834815	0.437261142612939	-0.0477117818889971	0.675678401	0.688656703185118	SLAMF7	0.136213704788596	0.0879061345132606	-0.0149188113630624	0.852407215022348	0.175393078615924	SLAMF7	-0.0859578465177461	0.495979485209768	-0.428103146853147	0.000423207331692171	0.571145827240365
SLAMF8	0.187644284233473	0.0977258183555441	-0.151022395326193	0.183627180	0.304861393601885	SLAMF8	-0.193930018505082	0.0146250996311692	-0.250618193877538	0.00149294030736294	0.0397121068156084	SLAMF8	-0.00769988737014655	0.951458893521258	-0.264335664335664	0.0336509287050665	0.962345531223353
SLAMF9	0.25920780723153	0.0210690839983734	0.349089461420335	0.001615206	0.114071815855414	SLAMF9	0.0840092140367254	0.293970580952639	0.0902721734707948	0.259320591365962	0.443666842305743	SLAMF9	-0.390562320772993	0.00129764688945812	-0.364291958041958	0.00301594885866904	0.00369930117649417
SLBP	0.341525004374448	0.00206763410795174	0.324756572541383	0.003646537	0.0230093075531315	SLBP	0.447275276791357	3.8230698608667e-09	0.229264548948623	0.0037599878354736	5.05376470225354e-08	SLBP	0.568602914078369	7.76240179467536e-07	0.526660839160839	9.12784694115367e-06	4.79533810521956e-06
SLC10A1	-0.0852494181142487	0.455065124557081	-0.0412523550475666	0.718123059	0.701912887194615	SLC10A1	0.168089666924085	0.0347618000324872	0.0286008537204993	0.721297281202806	0.0820993891723014	SLC10A1	-0.450309138839659	0.000167323436676464	-0.457806575711067	0.000125863193380397	0.000623324882326991
SLC10A2	-0.0414566648596897	0.716785884230492	0.0266667297467056	0.815541934	0.859319836487731	SLC10A2	-0.1071204957526	0.180359085050208	0.0945086239157354	0.237535987058222	0.306905513982284	SLC10A2	-0.335002150704986	0.00637637070256913	-0.376021679628376	0.00202253581223794	0.0145724043291396
SLC10A3	-0.258801414101636	0.0212788952525428	-0.198052580331061	0.080234200	0.114649454145687	SLC10A3	0.239530559757294	0.00243585218960574	0.265142992750608	0.000760464419687707	0.00835084521613439	SLC10A3	0.0906946736475681	0.472449748815493	0.00944055944055944	0.94047036426521	0.549266411453583
SLC10A4	0.0965167595539888	0.397461246634081	0.09937722422459	0.383554919	0.659153084760698	SLC10A4	0.0420416068051473	0.599937302224747	-0.0070372719846375	0.930070735597091	0.730382603753313	SLC10A4	0.27386891204877	0.0272754845276133	0.140080201933646	0.265730962328839	0.0507308240972955
SLC10A7	-0.240323569337451	0.0328945311977079	-0.200194741966894	0.076969740	0.152338850577186	SLC10A7	-0.024751626923439	0.757549738637812	0.0660142570714892	0.409884452549557	0.847244437660052	SLC10A7	0.293453754345066	0.0176688952953954	0.301573426573427	0.0149368084941146	0.0350172526990722
SLC11A1	0.204910929664417	0.0700584037492855	0.111489776046738	0.327266354	0.248451696136519	SLC11A1	-0.0427730576167425	0.593601564681197	0.0415678811485786	0.6040567552179	0.725837194857919	SLC11A1	-0.546092661093525	2.53587339720645e-06	-0.588374125874126	4.56501593863986e-07	1.43912834793681e-05
SLC11A2	-0.19023531685132	0.0930993433406347	-0.235710808179163	0.036736750	0.296038428054356	SLC11A2	0.0050416125713402	0.949870590242234	0.0969881731201748	0.225396614530114	0.97096710529481	SLC11A2	0.338558755543223	0.00580567241249241	0.355463286713287	0.00384914741046977	0.0134674852837865
SLC12A1	0.0623871975768566	0.584925984136011	0.0561847921134635	0.622853392	0.788217354652996	SLC12A1	0.180203691720344	0.023468502352012	0.000641963251407142	0.993612778827632	0.0591451236551048	SLC12A1	-0.375173224681848	0.00207431827914462	-0.403321678321678	0.000947687112345248	0.00551401232436205
SLC12A2	-0.0321694747354341	0.778369943063808	0.0602482960077897	0.597181220	0.893487803848964	SLC12A2	-0.255709628757622	0.00118367805763289	-0.273874909961691	0.000497612492648715	0.00445452024550726	SLC12A2	-0.191656722372614	0.126160145289972	-0.266083916083916	0.0324616778746478	0.185940496685488
SLC12A3	-0.0406148136020929	0.722301096509454	-0.204879919345634	0.070101734	0.862748139441187	SLC12A3	0.00370112592964213	0.963188156266941	0.0471128007016095	0.556651094443922	0.978820791120145	SLC12A3	-0.437358112437963	0.00026948975473278	-0.472465034965035	8.5915801095965e-05	0.000944472890391159
SLC12A4	0.0261300874043508	0.81919036995083	0.166845180136319	0.141449547	0.913660809614138	SLC12A4	-0.0610892872618523	0.445764194413732	-0.00896618816064857	0.910973598317978	0.598797432992106	SLC12A4	-0.382501482463014	0.00166372406883616	-0.420192307692308	0.000550867890873474	0.0045697164888811
SLC12A5	-0.210637211233776	0.062419024768738	-0.124975657254138	0.271850182	0.230690551701773	SLC12A5	0.263108519132734	0.000837753179264267	0.371572546141286	1.53413232559161e-06	0.00328429216998619	SLC12A5	0.303666786166192	0.0139282622118051	0.230725524475524	0.0646058059354387	0.0286202247205577
SLC12A6	-0.265848137731151	0.0178830963030548	-0.300511197663096	0.007324870	0.102608610003428	SLC12A6	-0.0844803260792626	0.291261949621959	0.0292017217393638	0.715691444490457	0.440783599880005	SLC12A6	-0.0301216611970972	0.811733294558481	-0.034222027972028	0.786221856168109	0.849168575905615
SLC12A7	-0.0104068808250897	0.927471557096207	-0.0436952288218111	0.701636418	0.965308548845312	SLC12A7	0.379524706356115	8.74603196976409e-07	0.2541170457217	0.001273454241556	7.06513596153387e-06	SLC12A7	0.367057897215698	0.00263288174427645	0.34256993006993	0.00543441873986059	0.00677678830822878
SLC12A8	0.258153845587895	0.0216169053092319	0.236051606621227	0.036459590	0.115707483952906	SLC12A8	-0.0379348632154716	0.636057651859042	0.085190861769679	0.287208292430089	0.758957407948192	SLC12A8	-0.0656209730098143	0.603517957692556	-0.21604020979021	0.0839683184508223	0.669259080687556
SLC12A9	0.0155523384633482	0.891791956545578	0.0306231742940604	0.788377655	0.949169117889694	SLC12A9	0.0390694890996905	0.625987257896594	0.079078675546658	0.32331992616566	0.751119430220706	SLC12A9	-0.108378833841724	0.39014237439793	-0.0899038461538462	0.475398033458845	0.470952642085691
SLC13A1	-0.124950931024048	0.272557090656182	-0.130950467584798	0.250010953	0.545665972162969	SLC13A1	0.0529537008488838	0.508742951423915	0.126156427581029	0.114224273950001	0.655420415375387	SLC13A1	-0.0621754120498043	0.622702136057319	-0.0326048951048951	0.796105716287712	0.685837049224249
SLC13A2	-0.101007821249042	0.37576008582488	-0.194447875401377	0.085947270	0.640919027866487	SLC13A2	-0.123459708311027	0.122234856834349	0.0164005001840702	0.837940963744607	0.227454562294239	SLC13A2	-0.455294694176196	0.000138562984293975	-0.377797202797203	0.00205077980744569	0.000527989259007644
SLC13A3	-0.107689490455375	0.344832719425134	0.0186708860759494	0.870050692	0.614469190779621	SLC13A3	0.251603092795828	0.00142790417545191	0.355741958155356	4.49531126881453e-06	0.00525124168399314	SLC13A3	0.455490740321078	0.000137530966515254	0.441083916083916	0.000270987324279233	0.000524488256955917
SLC13A4	0.0166341259727006	0.884315474107183	0.0633862011230665	0.578916920	0.945813943699539	SLC13A4	0.141457944380484	0.0762442441686695	0.145831178235908	0.0675043965584649	0.156326599303397	SLC13A4	-0.403283342742561	0.00086546708918187	-0.373776223776224	0.00230403696794575	0.00260348328165374
SLC13A5	-0.0504606424368307	0.658752101474393	0.0937150067558256	0.411366879	0.827382934080177	SLC13A5	0.155937809411236	0.0504027747056621	0.0590378454358204	0.461216756750344	0.112072901999745	SLC13A5	0.0639095507580762	0.613014469458808	0.00408658311413287	0.974226486776528	0.677325276115929
SLC14A1	-0.13348572460916	0.240878749356276	-0.112755598831548	0.321777350	0.51317879891359	SLC14A1	-0.08809268886889	0.271046032497647	-0.208501145113419	0.00856446675786591	0.416523994848221	SLC14A1	-0.567469142197505	8.25668761114195e-07	-0.698033216783217	0	5.08712117781451e-06
SLC14A2	-0.0255031518765862	0.823455057132593	0.0430747509425634	0.706226108	0.916041066424907	SLC14A2	0.220955124123012	0.00527280260605689	0.327138083709364	2.72341548976814e-05	0.01648304339309	SLC14A2	-0.116879513020638	0.353816458042964	-0.186756993006993	0.136114199327785	0.435894950171554

[illegible]

SNRPB	0.190761593719443	0.0921810428690879	0.064873417721519	0.569290219	0.294294861317969	SNRPB	0.586769268669669	4.44089209850063e-16	0.54520022179694	1.29610878939094e-13	1.98438892071351e-14
SNRPB2	-0.0963827217584001	0.398120104655814	-0.199342745861733	0.078255098	0.659153084760698	SNRPB2	0.155127730110027	0.0516258390330426	0.148628183860144	0.0623556539676338	0.114303207078753
SNRPC	0.351363574745641	0.00149785616362452	0.14311100292113	0.207869164	0.018174600738752	SNRPC	0.555497057513657	3.59712259978551e-14	0.364451056767455	2.50599751451527e-06	1.20844939894254e-12
SNRPD1	0.556685737036557	9.96123956653605e-08	0.431231742940604	8.515815493	6.61118779016611e-06	SNRPD1	0.489499447071803	6.7212901910807e-11	0.17722901582671	0.025899808061886	1.24862716869622e-09
SNRPD2	0.301781375723618	0.00687448397969703	0.236952288218111	0.035735531	0.053360885990769	SNRPD2	0.564384308095179	1.15463194561016e-14	0.339439087985542	1.28258231566576e-05	4.19265761709725e-13
SNRPD3	0.313879485634708	0.00484868642310943	0.22611976630964	0.045292500	0.041894853681963	SNRPD3	0.0827935874480702	0.301036809041348	-0.152892219390225	0.0551291730473262	0.451850184937681
SNRPE	0.443149150701316	4.31496227037975e-05	0.358933787731256	0.001241409	0.0012105152309726	SNRPE	0.055254697064395	0.49047537890331	-0.0626188469601039	0.434435413461099	0.639622149233761
SNRPF	0.129800502509587	0.254230390082108	0.144571567672833	0.203229785	0.528058039807176	SNRPF	0.520346211188033	2.42739162104044e-12	0.43005757132536	1.70675705007194e-08	5.82637284789498e-11
SNRPG	0.510140166068848	1.56702386933105e-06	0.376217137293087	0.000688689	7.46542045435589e-05	SNRPG	0.00456625775077637	0.954591784929448	-0.199287904339353	0.0120610522729058	0.973738628133374
SNTA1	-0.150531025948535	0.185442414988571	-0.196275559883155	0.083025488	0.444298011343908	SNTA1	-0.0750176481742126	0.348866259612992	-0.00630401827922085	0.937341349774811	0.503340739770782
SNTB1	-0.169602365036336	0.135102509504042	-0.154065238558909	0.174875238	0.374290157300874	SNTB1	-0.484032973405268	1.17044221425095e-10	-0.322431367359	3.60230536595189e-05	2.07190780426538e-09
SNTB2	-0.105627980291154	0.354200282950155	-0.0497565725413827	0.662605642	0.622691367850683	SNTB2	0.0307994952445169	0.70085929584717	0.07881544088983	0.32493828040737	0.805751485926889
SNTG1	-0.0965297864331826	0.397397247855146	-0.112703227244245	0.322703935	0.659153084760698	SNTG1	0.0420971802409931	0.599454869513115	-0.0548969854349041	0.493292126123631	0.730089927492178
SNTG2	-0.074994293103968	0.511267544252122	0.0167426564029916	0.883565941	0.741337939165577	SNTG2	-0.0346308780602162	0.665758458344414	-0.0395431107987613	0.621803830921784	0.78061966762908
SNTN	-0.0724540162643687	0.525718314790087	-0.084411175494264	0.459527064	0.750299958862631	SNTN	-0.0505582880698658	0.528128589473544	-0.0858742554460889	0.283345224895798	0.67193139821755
SNUPN	-0.0566484869877977	0.619982024038771	-0.0957156767283	0.400630776	0.807667268570598	SNUPN	0.0485816027005472	0.544401537619374	-0.144849423868449	0.0693917516396146	0.685435118832764
SNW1	0.397146211236198	0.000290194884699613	0.356888997078871	0.001328348	0.00547137695861673	SNW1	0.155932730992951	0.050410366571362	0.104519832024717	0.19123150847049	0.112072901999745
SNX1	-0.292077418409695	0.00900456185462	-0.304065238558909	0.006635134	0.0639239857720259	SNX1	-0.300294850580015	0.000126313733402156	-0.358372181998207	3.77486938763681e-06	0.00061324784597407
SNX10	0.0259252948544917	0.820583696616284	0.163948393378773	0.148565765	0.914469136942636	SNX10	0.227009968921393	0.00412607086316408	0.0984637758544519	0.218384109890388	0.0132613415835982
SNX11	0.0355215911786358	0.755961388666827	-0.079357351509250	0.486144209	0.881326586980649	SNX11	-0.268018895227466	0.000662357434823048	-0.32217991932958	3.66941450527203e-05	0.00267529626482938
SNX12	-0.130812100188281	0.250516095747823	-0.0145326192794547	0.898696854	0.524141261155236	SNX12	0.0837350232298823	0.295554702411262	0.213798102557494	0.00698994450785649	0.445376284799
SNX13	-0.137887704283067	0.225574981648349	-0.0695959104186952	0.541460649	0.495699000988597	SNX13	0.322574820931067	3.57197149696287e-05	0.235740467340188	0.00286535593766549	0.00019885090795006
SNX16	-0.162678165286171	0.152020899086223	-0.171105160662123	0.131457338	0.399398159156126	SNX16	0.107574589380669	0.178508277454277	0.124458723816644	0.11921804598286	0.304826768197128
SNX17	0.0733525098303759	0.5205837881032	0.0450097370983447	0.693101459	0.747502988318941	SNX17	-0.394014929424161	3.02133671924158e-07	-0.411979155970568	7.52484366142633e-08	2.69490353688166e-06
SNX18	-0.305468612214855	0.00618986134048555	-0.318622200584226	0.004372602	0.0497048630093882	SNX18	0.286973849376253	0.000256418266299629	0.191295921479735	0.0160516775611729	0.00114941813551891
SNX19	-0.0350374629608116	0.759185815718287	0.0574732229795521	0.614204209	0.883037935972493	SNX19	0.0290655236229484	0.716960796312811	0.0117165899581465	0.88383882099673	0.8175455021045
SNX2	-0.156089323921234	0.169542596925677	-0.11572541382668	0.309133167	0.422602129425732	SNX2	0.0871300411847723	0.276337810650335	0.0529954355195986	0.50840850487856	0.423137244786881
SNX20	0.0121119157215248	0.915629149609004	-0.137671237047566	0.226311247	0.959450677870479	SNX20	-0.115257522214	0.149282246969878	-0.145708924430167	0.0677371161043523	0.265817406588205
SNX21	-0.286441229699616	0.0104903499813511	-0.35786270691334	0.001286279	0.0709326980961238	SNX21	-0.501075220656979	2.00935977009591e-11	-0.252983721972178	0.0013410863726357	4.12860640261894e-10
SNX22	0.149483367561113	0.188556560511866	0.0766553067185979	0.501148510	0.448229766082174	SNX22	0.139635971401475	0.0801453497137143	0.219186334397504	0.0056577858643106	0.162820115673083
SNX24	0.0309993334036156	0.786236004977193	0.0313534566699124	0.783451571	0.897326921033274	SNX24	-0.0522808301986239	0.514150815463914	0.15006271311625	0.0598424735639456	0.659789345612536
SNX25	-0.112210082928499	0.324839312595782	-0.0903846153846154	0.427470653	0.596922040743229	SNX25	-0.11663158229451	0.144460314281756	0.00222253154100909	0.977889464822058	0.259388899436958
SNX27	-0.0830202490929075	0.466983626384017	-0.133081791626095	0.241777056	0.710789761630221	SNX27	-0.21427629615331	0.00686134738854955	-0.20748495683868	0.00890001210359162	0.0206266174760283
SNX29	-0.0331244783743943	0.771966523323826	0.0223953261927946	0.844410957	0.889378103238692	SNX29	-0.0200601861312597	0.802451531721833	0.0170378872411374	0.831734281528571	0.877733509535913
SNX3	-0.029546400446439	0.796032848830997	-0.0497322297955209	0.662760685	0.902112124002227	SNX3	-0.316909721244171	4.9720682419283e-05	-0.141574194294326	0.0760006356396658	0.000266015043109271
SNX30	-0.00908029585611843	0.936696461768211	-0.0290408958130477	0.799079245	0.969871436228744	SNX30	0.225525505481168	0.00438428210904362	0.172560330474213	0.03014971914984	0.0139862473795691
SNX31	-0.108052223111	0.343200628014965	-0.0373164151015978	0.744044082	0.613406747310209	SNX31	0.0724539343792253	0.365629613011493	0.0784231995882511	0.327359429355878	0.521250879290803
SNX32	0.00826945927513158	0.942339100363384	-0.119791048084863	0.293007260	0.972223481087126	SNX32	0.0966624254226305	0.226965885888893	0.116724740001114	0.14413772980074	0.364515568281822
SNX33	-0.253746252737765	0.0240414783684588	-0.355696202531646	0.001381585	0.123429660684632	SNX33	-0.371275520189843	1.56622316520289e-06	-0.24765786096939	0.00170502744330991	1.19594228417193e-05
SNX4	-0.145612175315193	0.200391445854393	-0.19944011684518	0.078107334	0.463169570656	SNX4	-0.0855202723074858	0.285341837155647	-0.254609927459747	0.00124502600418862	0.433613278874321
SNX5	-0.259564474819638	0.0208864032559905	-0.333495618305745	0.002798244	0.113505504235568	SNX5	0.0166284183237479	0.835720397660893	-0.0191478470271604	0.811264174774378	0.899471150792206
SNX6	0.25532102690805	0.0231499259995136	0.280866601752678	0.012400684	0.120775319551142	SNX6	-0.133185150542102	0.0952643327335574	-0.0270400397956444	0.73592778139371	0.18675501710412
SNX7	0.350303596886537	0.00155156048885674	0.229722492697176	0.041902549	0.0185867736817738	SNX7	-0.0567046977645733	0.479145819625572	-0.096786658008468	0.226366494611042	0.629986754699813
SNX8	0.0599366324955897	0.599785305781805	0.0851509250243428	0.454796581	0.796535774221547	SNX8	0.130322972011187	0.102659424286025	0.122488718104388	0.12522333493468	0.198420997491414
SNX9	-0.255411433804386	0.0230996082869922	-0.399878286270691	0.000292113	0.120608250448454	SNX9	-0.459555089277557	1.24860966018504e-09	-0.474298271034892	3.06869472919949e-10	1.80999242866194e-08
SOAT1	0.160821842752504	0.156814562859518	0.130029211295034	0.333673788	0.406649171379449	SOAT1	-0.439048916095365	7.89534064636634e-09	-0.45420725524974	2.04380874098871e-09	9.76836164647879e-08
SOAT2	0.15227255427659	0.180348556245149	0.148173057772231	0.192504429	0.436803054790174	SOAT2	0.0519312082087241	0.516972372062194	0.148842791903346	0.061974268336104	0.662222472144795
SOBP	-0.0844340711064148	0.459404873460823	-0.0526290165530672	0.644412044	0.704950701164416	SOBP	-0.252766916676878	0.00135439244097241	0.0169223244921575	0.832858837484363	0.00501193263081449
SOCS1	0.0426598398112618	0.708928669006648	0.155355404089581	0.171258479	0.856206665430148	SOCS1	0.362246630612323	2.91020420561949e-06	0.282824364483085	0.000317462971837775	2.08795243279247e-05
SOCS2	-0.255288290341133	0.0231681691657333	-0.297151898734177	0.008034258	0.120838620717266	SOCS2	-0.275817667289177	0.000451932817606415	-0.184816960608306	0.0200863685106637	0.00190827595691149
SOCS3	0.432584174287915	6.85078392477223e-05	0.120764362220058	0.288429902	0.00175200134394642	SOCS3	-0.0763999359949364	0.340031864993966	-0.122383752549063	0.125549743114433	0.493900654119061
SOCS4	0.165721967205667	0.144398736290639	0.185662122687439	0.101345813	0.389265502369504	SOCS4	0.39220645929414	3.45959478842417e-07	0.157454417947026	0.0481776284272755	3.05034195665177e-06
SOCS5	-0.257538989845367	0.0219420702081546	-0.283373904576436	0.011616568	0.116615004753808	SOCS5	-0.28222947775926	0.0003272444710139	-0.222816012875839	0.00489322991620932	0.00143169456068581
SOCS6	-0.23589992087954	0.0363544703913445	-0.254527750730282	0.023847196	0.162750206816932	SOCS6	-0.145820122604945	0.0675254149742704	-0.251626967345039	0.00142636027169785	0.141685827129572
SOCS7	0.0428776830745853	0.707509277300091	0.109664070107108	0.335288021	0.855272812327217	SOCS7	0.0160091685185434	0.84175661258811	-0.0727699909189793	0.36355635135813	0.90291529787747

[illegible]

SUSD2	0.366299912174095	0.000900029224098153	-0.0247809152872444	0.828071307	0.012714841424438	SUSD2	-0.366586741282996	2.16571796110678e-06	-0.391463714244319	3.65665469677021e-07	1.59954947071002e-05
SUSD3	0.344265567584514	0.00189200621443208	-0.0811850337395788	0.476921804	0.021589576191583	SUSD3	-0.0361426442453668	0.652100542650977	-0.0138082948649825	0.863282187163336	0.770846987941729
SUSD4	-0.105811040573104	0.353362132734182	-0.0631694255111977	0.579494386	0.622236061999659	SUSD4	0.0152696223875162	0.848977488867763	0.165316185916865	0.0379127129032666	0.907913488800319
SUSD5	-0.022745386257477	0.842287173896636	-0.0982473222979552	0.388242436	0.924977257642211	SUSD5	0.00953035196385396	0.905397243450363	0.0530533233438579	0.507944801900547	0.943915155435812
SUV39H1	0.474681684914523	9.88121303024947e-06	0.479138266796495	1.028544643	0.000346609386776631	SUV39H1	0.597654624107347	0	0.457474878624201	1.51395478465577e-09	0
SUV39H2	0.687740707825854	2.53530529903401e-12	0.58047711781889	3.881418713	3.88707505459648e-10	SUV39H2	0.631875657417358	0	0.399698185997558	1.9635361305115e-07	0
SUV420H1	0.117913113162328	0.300694354505252	0.240262901655307	0.033177070	0.573659973319656	SUV420H1	-0.262768281248886	0.000851361704410537	-0.13762505543022	0.0846358033973485	0.00333125801874559
SUV420H2	0.3624068161625	0.0010302439235419	0.402215189873418	0.000267502	0.0141402944620484	SUV420H2	0.12703905877259	0.111693261490576	0.19187551389392	0.0157277877620202	0.211943201818337
SUZ12	0.11695120249416	0.304682377080581	0.14858812074002	0.190855588	0.577227992641649	SUZ12	0.0917135072591229	0.251760252543389	-0.0259310558850383	0.746381293439832	0.393923699585568
SV2A	0.0793422265472809	0.487014209969682	0.0215920155793574	0.849928330	0.725040801263107	SV2A	-0.20006597863151	0.0117235947145968	-0.278858491979723	0.000388153091067588	0.0328610503495321
SV2B	-0.220031963111466	0.0513592935057833	-0.290043816942551	0.009737793	0.204368095551289	SV2B	0.125942724625067	0.114843756210555	0.144312426172366	0.0704421138402195	0.216648586108855
SV2C	0.0409030132980373	0.720411406737407	0.160569761440144	0.157474092	0.861415167434237	SV2C	0.418068037351679	4.61000417839585e-08	0.113656399057777	0.155053332941952	4.89165284866096e-07
SVEP1	-0.116977155960854	0.304574325891042	0.0061587147030185	0.957024448	0.577164991613781	SVEP1	-0.122708448560546	0.124542167241916	-0.2091796181232	0.00834664892860764	0.23083539296802
SVIL	0.00961997456247684	0.932942529463195	-0.184079844206426	0.104329787	0.967753547362127	SVIL	-0.401835652126912	1.66624692703764e-07	-0.253957315528815	0.00128279364008203	1.5698877137545e-06
SVIP	-0.27523138968176	0.0140911395875704	-0.259931840311587	0.020952005	0.0876122473319608	SVIP	-0.0617797190990007	0.440629806787023	-0.0481991126581733	0.547578622723298	0.594503095855558
SVOP	-0.0992964058145446	0.38394376799787	-0.0952669186290896	0.403629788	0.647258766063917	SVOP	-0.0229208946055449	0.774984555612034	0.0921308540165184	0.249599824688563	0.859878840201304
SVOPL	-0.114019719191979	0.317047432381671	-0.232430734784817	0.039276083	0.589490001133165	SVOPL	-0.0483858580520858	0.546026321535304	-0.0126453357916773	0.874698246833667	0.686682463246461
SWAP70	0.0900484645579345	0.429985632324948	-0.00457643622200584	0.968080635	0.684116791579344	SWAP70	[illegible]	[illegible]	-0.20541759177076	0.00961909960264312	0.0190107866484792
SYAP1	0.0146088946269836	0.898320044625593	0.173076923076923	0.127018990	0.951836164288277	SYAP1	0.076160405215219	0.341552480171041	0.131038186193824	0.100770423627798	0.49554583969965
SYBU	-0.104749951694763	0.358237471669003	-0.0447419668938656	0.694836994	0.625737058612518	SYBU	-0.384464613772381	6.12240233967132e-07	-0.336697555443588	1.52118858742873e-05	5.13500806712296e-06
SYCE1	0.26756686459381	0.0171292869476549	0.0297286790564989	0.794802015	0.0993790077004161	SYCE1	0.304898149134088	9.81009395855104e-05	-0.0560729039025291	0.484064861361761	0.000489441273108197
SYCE1L	0.184103017788107	0.104338019868445	0.117547499299995	0.302206131	0.317623111737433	SYCE1L	0.0748691019025721	0.349824156651078	0.0387414173544685	0.628892080928639	0.504327542987185
SYCE2	0.352350772244873	0.00144935514633771	0.580671859785784	3.831787655	0.0177055874516783	SYCE2	0.524516083052559	1.50945922428036e-12	0.426541985841943	2.29324107500581e-08	3.80673209849335e-11
SYCN	-0.042778632700784	0.708154532133284	-0.0829653336122777	0.467279364	0.855812097944329	SYCN	0.0594245721393473	0.458281250993804	0.12362061464225	0.121745001914853	0.610831120034433
SYCP1	-0.0469284594753418	0.681301861374981	-0.0966808584271169	0.396655502	0.840021707765998	SYCP1	0.0429042615610789	0.592468308288046	0.135509010684468	0.0895763922089491	0.724981160224854
SYCP2	0.205802966487766	0.0688211695299867	0.165680761508718	0.144499967	0.245871584350447	SYCP2	0.161199836111533	0.0430304549624219	0.00455460621444801	0.954707527755692	0.0981405693580509
SYCP3	0.0202148452847964	0.859643761689411	0.142681648407585	0.209697503	0.934023749859538	SYCP3	-0.203903502024669	0.01017772552266	-0.109914148824456	0.169194001969999	0.0291584075431327
SYDE1	0.153818460330735	0.175912922690777	0.0736124634858812	0.518330195	0.430944566069226	SYDE1	-0.0816030470693793	0.308064940026311	-0.075982892135744	0.342682164035357	0.459899087405481
SYDE2	-0.0647434575343671	0.570798806482211	-0.0425267770204479	0.709254350	0.780149924697632	SYDE2	-0.00868732810984014	0.913731551876094	-0.000629793331949187	0.993733861100007	0.948722456152439
SYF2	-0.0638824359594477	0.575942646487483	-0.213777994157741	0.058673593	0.782348465812461	SYF2	-0.185432537804849	0.0196685922542956	-0.281564777819186	0.000338503884756498	0.0511150174197603
SYK	-0.0668725817733755	0.558172127272003	-0.28427458617332	0.011345661	0.772242767728252	SYK	-0.397649893835485	2.29559998342672e-07	-0.450847179162648	2.77351458595557e-09	2.09736951339384e-06
SYMPK	0.254257079335341	0.0237491308845506	0.327239532619279	0.003384750	0.122607755320971	SYMPK	0.124593592665884	0.118815237300258	0.19852485363776	0.0124002450451522	0.222318174478885
SYN1	-0.254956199704051	0.0233533926676816	-0.263096397273613	0.019400869	0.121359422441951	SYN1	-0.0341047391602057	0.67053798057559	0.0616208659354244	0.441808248749164	0.783933715780568
SYN2	-0.236933645573094	0.0355198617486839	-0.39613914663993	0.000301652	0.160317623383266	SYN2	0.0414914973412354	0.604722154364288	0.0668174215439714	0.404198869122444	0.734217164271354
SYN3	-0.0997199675926893	0.381908479533067	-0.0960404197465703	0.399805602	0.645698815045734	SYN3	-0.0348513757103565	0.663759403204581	-0.060717295598441	0.44854435761104	0.779523514480501
SYNC	-0.0777325871862943	0.495921316365853	-0.140895813047712	0.215049165	0.731582151920195	SYNC	-0.237934909660716	0.00260901032945248	-0.21122937097353	0.00771810592372188	0.00888150809577258
SYNCRIP	0.208305954203995	0.0654433996667251	0.103164556962025	0.364847070	0.237624082588928	SYNCRIP	0.18535781461047	0.0197189008424881	0.0479357915049537	0.549771117654877	0.0512023927376877
SYNE1	-0.0992062006246892	0.384378058792803	-0.307473222979552	0.006028383	0.647549339591487	SYNE1	0.117763740285893	0.140576875925903	-0.140840956646985	0.0775477550718766	0.253976475544247
SYNE2	0.103174176074237	0.365553739712965	0.04114818977604674	0.718497652	0.631875709145518	SYNE2	0.0864826732847889	0.279935441040002	-0.0126871410349184	0.874287407806226	0.427312414556089
SYNGAP1	-0.0906847756601816	0.426720469680024	-0.0173807205452775	0.878965327	0.681696616760481	SYNGAP1	0.178802359932054	0.0245882641244384	0.1947902096041	0.0141838510840632	0.061512425945225
SYNGR1	0.0212317506749273	0.852660489342346	0.060126582278481	0.597923393	0.930577152533546	SYNGR1	-0.416052973407743	5.42740325066778e-08	-0.381634983042088	7.51544267180185e-07	5.68688069691485e-07
SYNGR2	-0.219848951924109	0.0515582429560959	-0.261222005842259	0.020307223	0.204786486100253	SYNGR2	-0.118970482419205	0.136525685048797	-0.195579733128935	0.0137891366525009	0.248265296498257
SYNGR3	0.0832615009401468	0.465685612281688	0.273833214666796	0.014607777	0.709612730099581	SYNGR3	0.378449811939256	9.44449447981555e-07	0.371718955727559	1.51854543622851e-06	7.55970188580019e-06
SYNGR4	0.287936462729202	0.0100767019235719	0.494227883147725	3.673149289	0.0687942736087004	SYNGR4	0.277814261264024	0.00040905079965281	0.165584674894839	0.0375975237858936	0.00174278673797116
SYNJ1	-0.01400773760469853	0.902485759248689	0.0339337877312561	0.766115695	0.953797336027604	SYNJ1	0.00112309408428966	0.988826005313364	0.0830870826193254	0.299320597785886	0.994085940774841
SYNJ2	0.122530968712931	0.282025722791637	0.185832521908471	0.101028497	0.556200149470083	SYNJ2	-0.182017711410635	0.0220842847190192	-0.14897806904456	0.0617348475627968	0.0562519403290348
SYNM	-0.184181301638364	0.104188177310205	-0.216918208373905	0.054995108	0.317362354973238	SYNM	-0.16069884314704	0.0436910754300987	-0.267423331409042	0.000681662189438948	0.0994378413485124
SYNPO	-0.362152419503998	0.0010393202206274	-0.385370009737098	0.000497781	0.0141959083726303	SYNPO	-0.344813586353567	9.13645113946358e-06	-0.177886191477	0.0253448733760366	5.86683172227152e-05
SYNPO2	-0.243006922254184	0.030933675997071	-0.28960564751704	0.009852479	0.146645791915165	SYNPO2	-0.326380308256435	2.84974155762049e-05	-0.307474536345173	8.5002707659763e-05	0.000162880292070144
SYNPO2L	-0.16567377683472	0.144517132146351	-0.121242419709837	0.287155634	0.389265502369504	SYNPO2L	-0.0623538791983331	0.436386035485994	-0.0701702343546381	0.380973968048349	0.590725508330673
SYNPR	-0.123316873553335	0.278927016051732	-0.046650645351541	0.683087675	0.552601274513789	SYNPR	-0.0391114853824675	0.625615826525417	-0.0557078910638588	0.486919104557016	0.750771616343975
SYNRG	-0.241353914108043	0.0321296127352068	-0.282716650438169	0.011817806	0.149801857773909	SYNRG	0.0256777380509132	0.748775697997348	-0.0603673642312551	0.451168523852782	0.840854007571744
SYP	-0.135139446487507	0.235047538853999	0.0632181110029211	0.579201669	0.507286143982365	SYP	0.152516967612435	0.0557360513753817	0.289306367834378	0.000227083695808975	0.121489546036085
SYPL1	0.106821431262034	0.348758159447727	0.125608568646543	0.269415163	0.618076960353773	SYPL1	0.00617267521117093	0.938644252686381	-0.0650436557829744	0.416817775224541	0.963396370182097
SYPL2	-0.195970322713651	0.0834722180785941	-0.116455696202532	0.306074196	0.276211063938169	SYPL2	-0.334702228580394	1.72057095732997e-05	-0.368369770083917	1.9357518665855e-06	0.000103854791227688

[illegible]

TLR10	-0.162554776105796	0.152336098738591	-0.216773012158663	0.054997743	0.399772321008855	TLR10	-0.00220533941406768	0.978060455360085	0.0711058509974778	0.374640727113659
TLR2	0.0148415465496357	0.896709583410519	-0.224342745861733	0.047046631	0.950944886900442	TLR2	-0.48060383617214	1.6493364598763e-10	-0.402600711788281	1.57071564882393e-07
TLR3	-0.299547748528731	0.00732089951964929	-0.285491723466407	0.010988353	0.0557109467870811	TLR3	-0.000213376334021 08	0.997876989595878	0.110343138485349	0.167526006206577
TLR4	-0.0349686946410012	0.759644166184697	-0.222638753651412	0.048781171	0.883207472741223	TLR4	-0.54412462681446	1.47875430076754e-13	-0.440687995971884	6.84356049779349e-09
TLR5	-0.219938996300333	0.0514602778325058	-0.244693281402142	0.029996234	0.204579170848502	TLR5	-0.442957682033434	5.60739417282909e-09	-0.38891563735781	4.41747137539449e-07
TLR6	-0.165786779956303	0.144239616911324	-0.0987828627069134	0.385651602	0.389081943682663	TLR6	0.0764507286953136	0.339709970397781	0.119805250863909	0.133775893344647
TLR7	-0.128100171843694	0.260557793672529	-0.240081792128302	0.033076221	0.534151015937791	TLR7	-0.350635047513356	6.2824279942523e-06	-0.299340466427462	0.000133040011770499
TLR8	0.116109404339068	0.308200531109345	-0.12302148477034	0.280089016	0.58086431334864	TLR8	-0.145474469239431	0.0681852481837621	-0.306875167811869	8.78949249804331e-05
TLR9	-0.0488365868654915	0.669083971894096	0.00180136319376826	0.987484824	0.833315765897687	TLR9	0.272853950264674	0.000523292295425914	0.27195662640713	0.000546866532374059
TLX1	0.151051564913672	0.183909063832 8	0.146653078369901	0.197158226	0.441909058982 52	TLX1	0.228636013938032	0.00385898666968254	0.237829454535834	0.00262083759168938
TLX2	0.101956058913738	0.371271603643958	0.0263516193663842	0.817683785	0.6373486022046	TLX2	-0.0244461917762188	0.760450312080078	0.0336711246602978	0.674486958219162
TLX3	0.113281646668141	0.320210771497218	0.0287706457518771	0.801276652	0.592652324737569	TLX3	0.196539386953485	0.0133223278551156	0.129499558717863	0.10503104953743
TM2D1	0.237436197239751	0.0351199371384081	0.213412852969815	0.059114087	0.159422106202303	TM2D1	-0.0950424296561109	0.234884709486046	-0.236315496034576	0.00279606973069281
TM2D2	0.0824702951059399	0.46994989944871	0.0904576436222006	0.427096152	0.713101606511266	TM2D2	0.22882601001679	0.0038288164247553	0.155282087323781	0.0513909109880075
TM2D3	-0.0343579797186661	0.763718281105682	-0.265141187925998	0.018452025	0.885987960708119	TM2D3	-0.1999771346097	0.0117617029884814	-0.234578240031953	0.00301015414356785
TM4SF1	0.332189799439433	0.00278124549 33851	-0.188753651411879	0.095709137	0.0283105884550543	TM4SF1	-0.545066428440051	1.31757233871359e-13	-0.485506766855669	1.00883709445920e-10
TM4SF18	0.0336015007937534	0.768773691743862	-0.109055501460565	0.337989401	0.887797586949026	TM4SF18	-0.397604485443358	2.30353799984771e-07	-0.304281453727392	0.000101504643349844
TM4SF20	0.0425050894876082	0.709937571546433	0.113380022869105	0.319787971	0.856898894791002	TM4SF20	0.117970548461062	0.139876168312687	0.0995211890029686	0.213455389712536
TM4SF4	-0.112418454847274	0.323935938503316	-0.227388309506354	0.043868975	0.59612334865244	TM4SF4	0.0346391968977193	0.665682996054129	-0.102197054114419	0.2013392991162
TM4SF5	-0.108241288057 98	0.342351864368372	-0.135717205814039	0.233033583	0.612874291589216	TM4SF5	0.0625524515709668	0.434923729722957	-0.0143011874807943	0.858452136853116
TM6SF1	-0.136031992985524	0.231941361497248	-0.230842259006816	0.040893027	0.503243979212705	TM6SF1	-0.374409347732985	1.25760165454589e-06	-0.383897358821221	6.38024290220049e-07
TM6SF2	0.160896659460419	0.156619212963463	0.337296779601722	0.002367455	0.406404459983124	TM6SF2	-0.0105954221824519	0.894882587225681	0.0693609874680159	0.386503975863092
TM7SF2	-0.18447187528499	0.103633457373772	-0.178846153846154	0.114691504	0.316314025564426	TM7SF2	-0.649714964108925	2.57663035491311e-20	-0.460216503031834	1.17408737367679e-09
TM7SF3	0.218011210574988	0.0535912982814555	0.204503407984421	0.070726834	0.209886870774382	TM7SF3	-0.204842953305561	0.00982784308404332	-0.2678082051119	0.000669128498213075
TM9SF1	0.103669484319856	0.363244250922317	0.176290165530672	0.120033472	0.630041001965412	TM9SF1	0.111651808020233	0.162513235634801	0.162546008000248	0.0412966605383711
TM9SF2	0.0284113842990727	0.803572706757098	0.0813047711781889	0.475480592	0.905948868166328	TM9SF2	0.257049803039173	0.00111265903848801	-0.00764119399170619	0.924086827431555
TM9SF3	0.0385996100191699	0.735560407951541	0.0999026290165531	0.380268138	0.870237171434212	TM9SF3	-0.111621198990845	0.162629186975064	-0.0947413017402498	0.236377772656958
TM9SF4	-0.0996643063534131	0.382175570620951	-0.108081791626095	0.342340169	0.645892926612036	TM9SF4	0.46239599659027	9.57695700520844e-10	0.525122897171178	1.40795711090456e-12
TMBIM1	-0.209318455561594	0.0641155937430543	-0.299537487828627	0.007524564	0.234655479839032	TMBIM1	-0.660794091305792	3.48269614362799e-21	-0.54228552608676	1.85063796497384e-13
TMBIM4	-0.37788266923119	0.000596057631200107	-0.305574488802337	0.006360062	0.00926794640713 5	TMBIM4	-0.35576787560494	4.48761490030691e-06	-0.172374739202479	0.0303302602297563
TMBIM6	-0.186363814169707	0.100077727296257	-0.0520204479065239	0.648249601	0.309319782851285	TMBIM6	0.470310367112204	4.51522375044533e-10	0.490672897665571	5.95915565107984e-11
TMC1	0.216317026177414	0.0555232296626607	0.280814652625176	0.012182499	0.214689821362288	TMC1	0.0990104059631805	0.215826188421285	0.067450308659817	0.39975177848389
TMC2	-0.0434680584254573	0.703667612939423	0.00566405489289509	0.960488025	0.852922599051045	TMC2	-0.0167769207977293	0.83427426059617	0.0822844938092935	0.304029097387148
TMC4	-0.125639210627652	0.269903462515435	-0.05	0.661055990	0.542948651999647	TMC4	0.117320198714547	0.142088694832641	0.287713629625318	0.000246753826180382
TMC5	-0.11716868471358	0.303777711895566	-0.169620253164557	0.134877245	0.576649926659997	TMC5	-0.0409978820202831	0.609029991916099	0.0178213800274744	0.82419118707527
TMC6	0.149694510326517	0.187925919743817	-0.0996105160662123	0.381668114	0.44713577055608	TMC6	-0.146760012184168	0.0657574911165513	-0.206938884486334	0.00908525660563331
TMC7	0.376772450677309	0.000620482860320237	0.285029211295034	0.011122953	0.00952788044364414	TMC7	0.0898627462421355	0.2614963024919	0.00501705311261139	0.950114471993321
TMC8	-0.0183198814377932	0.8726851729635	-0.222297955209348	0.049134348	0.940917148187101	TMC8	-0.223788233338023	0.0047048213100915	-0.230334155817682	0.00359676066197453
TMCC1	-0.184314324636762	0.103933943317228	-0.173052580331061	0.127073076	0.316886362 98406	TMCC1	-0.22569148151706	0.00435470860701577	-0.257941442911363	0.00106759370222668
TMCC2	-0.278957208252295	0.0127908990839799	-0.21755111976631	0.054277134	0.0820825444785706	TMCC2	-0.0532574809805518	0.506311179474711	0.036696870885532	0.647121934086904
TMCC3	0.10050990013077	0.378130090888335	0.018889970788705	0.868538469	0.642772405465362	TMCC3	0.237907732814255	0.00261205370282847	0.334913141003066	1.69838454681855e-05

[illegible]

TRIM29	0.345641342136291	0.00180900650086557	0.0311202125907968	0.785422410	0.0208499595420275
TRIM3	0.133858606129102	0.239555285642692	-0.0467137293086666	0.682094626	0.511842614355521
TRIM31	-0.190718274455477	0.0922563602267528	-0.330534419194113	0.002928638	0.294392754528028
TRIM32	0.178498373285001	0.115508499012067	0.200925024342746	0.075881544	0.338097838309999
TRIM33	-0.160228231128032	0.158370933779188	-0.171080817916261	0.131512863	0.408298535514408
TRIM35	-0.0421162918501562	0.712474554457977	0.00815481986368062	0.943088514	0.857967286700119
TRIM36	-0.12967310428139	0.254700809085702	-0.108154819863681	0.342012643	0.528303001789033
TRIM37	0.242412979884486	0.0313590025704515	0.178529698149951	0.115342717	0.147565632366974
TRIM38	-0.0718491412346147	0.529189170905177	-0.10912852969815	0.337664510	0.75296660876124
TRIM4	-0.272831041204157	0.0149880869723386	-0.234591041869523	0.037659892	0.0905696254625459
TRIM40	0.00984193122570541	0.931399048364343	0.157512588751391	0.165637413	0.966992670790025
TRIM41	-0.248449080681327	0.0272576809747595	-0.158252190847128	0.163339362	0.134793776144693
TRIM42	0.17039065722854	0.13327069356787	0.125109319543329	0.271944886	0.37145261800808
TRIM44	-0.0184734724943564	0.871626824779018	0.124172346640701	0.274962102	0.940276826686998
TRIM45	-0.0736743604291952	0.518750724531238	0.0260223953261928	0.819596471	0.746421650242808
TRIM46	0.31651998063716	0.00448446029628879	0.276662041514337	0.013578939	0.0395424234239856
TRIM47	0.0410386910645109	0.719522362885174	-0.12921129503408	0.255835528	0.860997127524808
TRIM48	0.108752709520077	0.340062556304271	0.130036483852252	0.253360593	0.610932622271403
TRIM5	-0.0322629623107398	0.777742437633479	-0.0268013631937683	0.814289443	0.892963539505105
TRIM50	-0.215771516204474	0.0561572655781551	-0.343490405863261	0.001940266	0.216001244380543
TRIM52	-0.20038713718703	0.0766108859742251	-0.168622200584226	0.137213658	0.262696310032626
TRIM55	-0.0944113994012019	0.40788438022588	-0.136541785380899	0.230180093	0.667695504051925
TRIM56	-0.198359884590294	0.0797019664728535	-0.0672590068159688	0.555148068	0.269088020150225
TRIM59	0.675350497106708	8.68882743532140e-12	0.542015579357352	3.985636106	1.23631387781141e-09
TRIM61	-0.0192801423155492	0.866072055012415	-0.0419137874532810	0.713797174	0.937606028519692
TRIM62	0.0690346607481425	0.545487882613057	-0.0278481012658228	0.807171369	0.763177767859293
TRIM63	-0.135351421708012	0.234307236601261	-0.215580936068586	0.056380161	0.506517491037243
TRIM65	0.123928285545651	0.276532069399186	0.0132911392405063	0.907317853	0.549728743449302
TRIM66	-0.0611058570652111	0.592674650760582	-0.0734420642648491	0.519301162	0.792618927402252
TRIM67	-0.121876226970489	0.284624685240079	-0.051581095001422	0.651660936	0.559130776125551
TRIM68	-0.338661148790068	0.00226669957175457	-0.32149464459591	0.004017939	0.024484316837882
TRIM69	0.0357010374103757	0.754767281756915	0.122638753651412	0.280969345	0.880851318734187
TRIM7	0.338215871423164	0.00229915091062405	0.30562317429406	0.006351360	0.0247942239423787
TRIM72	-0.0685570293401467	0.548277890380655	-0.057882128769307	0.612370562	0.765045441685221
TRIM78P	-0.15544036479652	0.17134552118557	-0.100122323044229	0.379981112	0.424617431150007
TRIM8	0.139166288061334	0.221260354491311	-0.0065238558909445	0.954474100	0.490537752620687
TRIM9	-0.245293541748744	0.0293412536044376	-0.225243427458617	0.046150643	0.141263708322436
TRIML1	-0.026966996633178	0.813502233414391	0.0466888823360132	0.682842160	0.911300916531094
TRIML2	0.000644838771364967	0.99549987786161	-0.0730625899203863	0.522237790	0.998124129795545
TRIO	0.101272361167115	0.374504594949953	0.241942551119766	0.031939202	0.639812687511377
TRIP10	-0.101890088475321	0.371582814951881	-0.145253164556962	0.201090349	0.637458023890738

[illegible]

USP29	0.182415789558672	0.107608484248569	0.101134880169653	0.375156753	0.323725605098774	USP29	0.142615783499966	0.0738458722575615	0.152895378165322
USP3	0.239991577511647	0.0331442312845869	-0.000925024342745862	0.993614515	0.152844057019038	USP3	-0.0474992234941357	0.553415534343294	-0.137920175977075
USP30	-0.452543488316476	2.82363651729908e-05	-0.423417721518987	0.000117021	0.000857847665731816	USP30	-0.0722685123289235	0.366861036490085	0.0776167628598115
USP31	0.0651727019063755	0.568242489808296	0.0642161635832522	0.573216089	0.77900464153521	USP31	0.18952739981344	0.017076122257073	0.144980250502622
USP32	-0.116602964439592	0.306134597334929	-0.18300876338851	0.106388417	0.57873244227573	USP32	-0.306624611205494	8.9131047155795e-05	-0.267812768831697
USP33	-0.0335297986353821	0.769253368394221	-0.145374878286271	0.200710017	0.887912113775567	USP33	-0.046826895644208	0.559050871321161	-0.242001890901306
USP34	0.145297312517905	0.201376895528062	0.211027263875365	0.062058949	0.464425733090306	USP34	-0.130636662776735	0.101827508156856	-0.155560474231381
USP35	-0.226173668159171	0.0450393859668082	-0.31966893865628	0.004240250	0.187930163639564	USP35	-0.196371838060847	0.0134028186160526	-0.0397302233104273
USP36	-0.0694882368845086	0.542844799167276	-0.121713729308666	0.284634891	0.761666085612728	USP36	-0.210460328674461	0.00794882628456862	-0.30091669988043
USP37	0.431397098980073	7.20919255547958e-05	0.435978578383642	6.995600056	0.00181634917658949	USP37	-0.0221269351398908	0.782581403591575	-0.144592334319899
USP38	-0.259056696840939	0.0211468914677832	-0.257935735150925	0.021984189	0.114367847812364	USP38	0.381211965763187	7.74805864356409e-07	0.34863472519191
USP39	0.276944193200162	0.0134798504709974	0.27794547224927	0.013372081	0.0851771509953312	USP39	0.420769866849964	3.69758117457764e-08	0.152913516749276
USP4	-0.0857045012078888	0.45265278589886	-0.215433300876339	0.056710231	0.699911406419174	USP4	-0.0669392680276453	0.403340422429465	-0.145791071386508
USP40	-0.224581844911878	0.0466122064306498	-0.246932814021422	0.028489124	0.192181825888137	USP40	-0.427312506632831	2.15010476992823e-08	-0.254841155929449
USP42	0.189604108705038	0.0942102142871413	0.205963972736125	0.068705866	0.297892295890179	USP42	0.582512882193895	8.88178419700125e-16	0.469555402079232
USP43	0.0901901348733569	0.429257437929234	0.246048401601572	0.028830982	0.683783312448807	USP43	0.145588695165234	0.0679666188150554	0.0917233012478255
USP44	0.198498939460085	0.0794868144749235	0.152793671247678	0.178844318	0.268595844222337	USP44	-0.0970966940075207	0.224875529178272	0.0761687151494362
USP45	-0.19021480872584	0.0931352731335203	-0.16419182083739	0.147957593	0.296038428054356	USP45	0.0317072481073801	0.692482226100466	0.0700424502003295
USP46	-0.194753556145268	0.0854457150624187	-0.16222005842259	0.152937976	0.280349262193848	USP46	-0.316280280514754	5.15606830000653e-05	-0.257447039933383
USP47	-0.20679325251902	0.0674683448639007	-0.114143135345667	0.315828994	0.242351784365824	USP47	-0.267633050574587	0.000674805987111907	-0.205522557326085
USP48	0.293138919711677	0.00874635615477315	0.369839337877313	0.000859022	0.0627360144191274	USP48	-0.336871672910916	1.50486615502785e-05	-0.448128380480492
USP49	0.0444041305661401	0.69759165061744	0.177629016553067	0.117211851	0.849776279004172	USP49	0.0273802615875048	0.732730360319092	0.047321210572327
USP5	-0.171033910337887	0.131789929767021	-0.0199123661148978	0.861487779	0.36898941547737	USP5	0.368472012504712	1.90228426788508e-06	0.396733289369614
USP51	-0.129029570600317	0.257086131707042	-0.0328140214216164	0.773625201	0.530804933685386	USP51	-0.0554420294500305	0.489003681883703	0.109824395668453
USP53	-0.298711679610942	0.00749444171463727	-0.407570593962999	0.000218138	0.0566826264749889	USP53	-0.285479394295702	0.000277023052064212	-0.381493507728389
USP54	-0.105964116085514	0.352662213869906	-0.00377312560856865	0.973696223	0.621487415657044	USP54	0.128893292228161	0.106518979876436	0.0863562929180142
USP6	-0.168209760077126	0.138385133618952	-0.196007789678676	0.083452732	0.37958550196264	USP6	-0.125644659057899	0.115712156697584	0.00370421923501515
USP6NL	0.163260132078107	0.150540815245724	0.0646786757546251	0.570452120	0.397145023866871	USP6NL	-0.173686573233996	0.0290736256524487	-0.16192938030723
USP7	-0.211009455882051	0.061946779614894	-0.0560856864654333	0.622793928	0.229859032628024	USP7	-0.122421031722419	0.125433742105157	-0.089729688723911
USP8	0.0244644202612406	0.830538764782082	-0.00109542356377799	0.992422590	0.919902226122034	USP8	-0.133089863444609	0.0955035366253158	-0.23156010000638
USP9X	-0.247796645902861	0.0276778973893339	-0.22205452775073	0.049387909	0.136138635803593	USP9X	-0.270377276880347	0.000590752707238011	-0.0808037014810265
USP9Y	-0.1369103842875	0.228912452718893	0.148327493387073	0.192036053	0.499641672603301	USP9Y	-0.0823530931185817	0.303624756180585	-0.136100773018111
USPL1	0.155066101728687	0.172391667558578	0.165993184031159	0.143515226	0.426248581194344	USPL1	0.0858427469450769	0.283522565013132	-0.0707848152872648
UST	-0.0667098298426624	0.559132601914235	0.000438169425511198	0.997020082	0.772700451107225	UST	-0.0191792653575236	0.810960291772485	0.0271358779113758
UTF1	-0.0979510284322906	0.39045157965371	-0.0191149402947145	0.867209125	0.653329216784355	UTF1	-0.0768189205590223	0.337382372571618	-0.0461986760609792
UTP11L	0.535451974777728	3.68619313073637e-07	0.433836416747809	7.647347333	2.09498642930184e-05	UTP11L	0.0255625674019741	0.749865106486311	-0.167811147046206
UTP14A	0.126080757860499	0.268210276898103	0.0702044790652386	0.537923664	0.541181682971912	UTP14A	0.1216440175568	0.127868724406885	0.0359925367969028
UTP15	-0.0440440422909617	0.699926728157433	0.0701314508276534	0.538347498	0.850829030970162	UTP15	0.445583281093351	4.44506498276098e-09	0.305735759102618
UTP18	0.410986871458526	0.000168298826126456	0.0772151898734177	0.498019788	0.00355621173411223	UTP18	-0.051498338756894	0.520476733284717	-0.194785645884304
UTP20	0.0214678167383198	0.851040978668956	0.163704965920156	0.149175816	0.929579084117447	UTP20	0.153767879087045	0.0537342038721982	0.151806054078602

[illegible]

ZCCHC6	0.0480692250282847	0.673987351475205	0.0159444985394353	0.888906931	0.835775291812717	ZCCHC6	0.0743302776840395	0.353312606154589	0.0890883741520296	0.265645433347521	0.508083508772534	ZCCHC6	0.169600589301922	0.176810710868704	0.174344405594406	0.164485517301484	0.246786962196058
ZCCHC7	0.292485827008974	0.00890443898521864	0.253481012658228	0.024445810	0.0635324654580282	ZCCHC7	0.305481428877249	9.49801265623944e-05	0.229314749866387	0.00375218027853491	0.000475675769862262	ZCCHC7	0.452219322233471	0.000155712128002028	0.41333041958042	0.000689078587159347	0.00058505724927433
ZCCHC8	0.163283963947839	0.150480435342716	0.121543330087634	0.285313585	0.397091667806302	ZCCHC8	0.215179635998857	0.00662415098776181	0.171735818430936	0.0309588456170748	0.0200214447027902	ZCCHC8	0.608885668437306	7.38619774232063e-08	0.605332167832168	1.8284261361687e-07	5.22015073885096e-07
ZCCHC9	-0.0549588193717448	0.630472110121151	0.0739532619279455	0.516391038	0.812455352238301	ZCCHC9	0.381550652914202	7.56127725320965e-07	0.455059149611797	1.89058036769618e-09	6.21997829453037e-06	ZCCHC9	0.622031939377403	3.18724406866977e-08	0.678671328671329	0	2.35384479050995e-07
ZCRB1	-0.330003333008825	0.00297738428874483	-0.294109055501461	0.008728388	0.0298160539052128	ZCRB1	0.0209273536097382	0.794098203485907	0.0851833512459582	0.287250942334691	0.873005370009289	ZCRB1	0.162545947551318	0.195775103071784	0.13208041958042	0.293489999105051	0.268547227383471
ZCWPW1	-0.127960905118764	0.261080734738995	-0.355428432327167	0.001393798	0.534761264671483	ZCWPW1	-0.379849168535486	8.54506587730127e-07	-0.326392924403445	2.84759393639687e-05	6.92406086272519e-06	ZCWPW1	-0.420561948781536	0.000486368396058808	-0.396765734265734	0.00116189486538765	0.00157741101424478
ZCWPW2	-0.302092294398654	0.00681428390867502	-0.371543330087634	0.000810103	0.0531229432975067	ZCWPW2	-0.00222374478160239	0.977877398135332	0.079258181995199	0.322219318008779	0.987400112970232	ZCWPW2	0.140806857278503	0.263234438686055	0.0761363636363636	0.545750303048637	0.343078761956614
ZDBF2	-0.138604687145108	0.223148301030737	-0.106767283349562	0.348269405	0.492569988592178	ZDBF2	-0.0948721750290446	0.235728057464677	-0.0292397527376699	0.715337138467033	0.374864181038559	ZDBF2	-0.367666921164561	0.00258672836658681	-0.402447552447552	0.000974008376454178	0.00667133952896999
ZDHHC1	-0.0928479600599227	0.415726869019165	-0.171397273612464	0.130792433	0.673733695137745	ZDHHC1	-0.0369728798806585	0.644648353509665	0.0269487858974866	0.736786163664134	0.76567588310406	ZDHHC1	-0.177280966716225	0.15772328307706	-0.144055944055944	0.251595894607916	0.224460565077099
ZDHHC11	0.0962524568056643	0.398761035122616	0.252726387536514	0.024885283	0.659558436250749	ZDHHC11	0.306605511770036	8.92259383351579e-05	0.132328097986145	0.0974329992519477	0.000449794502943663	ZDHHC11	-0.234289540187859	0.0603142253815302	-0.184659090909091	0.140629230081309	0.0996559506196932
ZDHHC12	0.290502927358706	0.00939983571696579	0.163047711781889	0.150832359	0.0657622747825077	ZDHHC12	0.575186347593978	2.66453525910038e-15	0.339816355775696	1.2526608785037e-05	1.07105008995716e-13	ZDHHC12	0.472975596015806	6.93072821167373e-05	0.445104895104895	0.000235234001999434	0.000286481644444697
ZDHHC13	0.0948775691089704	0.405562827523033	0.182083739045764	0.108191732	0.665219904026418	ZDHHC13	0.318302304920416	4.58665854528029e-05	0.388931145585815	4.41241356128927e-07	0.000247989379790338	ZDHHC13	0.614273703145086	5.25826711061939e-08	0.611013986013986	1.33032974388941e-07	3.78438366320525e-07
ZDHHC14	-0.15148490432752	0.182639614141198	-0.0506329113924051	0.657033601	0.439749707204446	ZDHHC14	-0.104939459554784	0.189445608899222	-0.0299699479051472	0.708546318608028	0.318249261778874	ZDHHC14	-0.125473023157783	0.319293688355877	-0.17770979020979	0.156393976466092	0.401500804773706
ZDHHC15	-0.263334363966975	0.0190368264785727	-0.267599805258033	0.017364340	0.106908453543646	ZDHHC15	-0.0610130457911644	0.446333208723482	-0.0635544585708874	0.427587723863126	0.599343134398199	ZDHHC15	0.1637075466495	0.192556336349885	-0.000131118881118881	0.999308770577706	0.264809958552125
ZDHHC16	0.418246517520023	0.000125258154248131	0.292283349561831	0.009169727	0.00280561242890095	ZDHHC16	0.499211887530903	2.4479529514565e-11	0.28453575940686	0.000290813958701616	4.95239712486969e-10	ZDHHC16	0.673475655750064	7.88603404799915e-10	0.553977272727273	2.57571513818129e-06	6.62701554976997e-09
ZDHHC17	-0.300730981741688	0.00708133965581346	-0.29690847127556	0.008087941	0.0544258655635335	ZDHHC17	0.0611148932177118	0.445573180886045	0.269235128168345	0.000624488571834521	0.598653607373879	ZDHHC17	0.634952178744673	1.34225217607309e-08	0.661276223776224	2.91889617337745e-09	1.02426887637077e-07
ZDHHC18	0.507792262238547	1.78173852916075e-06	0.391699123661149	0.000395618	8.30276108936309e-05	ZDHHC18	0.197532991337789	0.0128536385512015	0.123606829454588	0.121786909199225	0.0355628923546167	ZDHHC18	-0.217598263143393	0.0816443134182377	-0.256293706293706	0.0396026461939336	0.128806696101252
ZDHHC19	-0.150445141025218	0.185696294325927	0.152361246348588	0.179737744	0.444501007538098	ZDHHC19	0.346684760084327	8.10681204965746e-06	0.37527277816625	1.18333434724834e-06	5.27000034725261e-05	ZDHHC19	0.224735302602649	0.0718895098685679	0.10590034965035	0.400211058370332	0.11556611706504
ZDHHC2	-0.139531327093106	0.220039229307246	-0.216820837390458	0.055106255	0.489256422398956	ZDHHC2	-0.557639058635715	2.72543811416252e-14	-0.354853554034925	4.76683018225989e-06	9.27455003359186e-13	ZDHHC2	-0.476753612806204	5.94740793595539e-05	-0.487805944055944	4.71457950207837e-05	0.000250167031959184
ZDHHC20	0.016141595114377	0.887718256209589	0.144206426484908	0.204182604	0.947301805941149	ZDHHC20	0.39214482626784	3.47555263235932e-07	0.194800858283626	0.0141784623830097	3.06147961539402e-06	ZDHHC20	0.518221858565848	9.7978726729675e-06	0.510270979020979	1.86508376177726e-05	4.98612900694193e-05
ZDHHC21	-0.146479603666359	0.19769445210647	-0.0482716650438169	0.672089157	0.459405577929945	ZDHHC21	-0.023277076082948	0.771583373284952	-0.0454485642157341	0.570689337442969	0.857521877583819	ZDHHC21	-0.0635697460113814	0.614907648683753	-0.0555944055944056	0.65930755852721	0.679028328971331
ZDHHC22	0.0537444786521691	0.638056708192293	0.366855426038991	0.000882720	0.816689462504994	ZDHHC22	0.12523501597821	0.116913963698329	0.145997960017293	0.0671879656587324	0.219663786692553	ZDHHC22	-0.206973904648644	0.0980735873167427	-0.337718531468531	0.00616636099579348	0.150471760898614
ZDHHC23	-0.0787825742072588	0.490101462647298	0.143889970788705	0.205385499	0.727392070256511	ZDHHC23	0.621550063948087	0	0.508974188322331	8.58550638708994e-12	0	ZDHHC23	0.654746171260276	3.29134919319074e-09	0.599956293706294	2.45624925515626e-07	2.64587460306482e-08
ZDHHC24	-0.146983282990562	0.196140421328881	-0.237804284323272	0.035061765	0.457460525122949	ZDHHC24	0.171338752015702	0.0313550420209221	0.119280423087284	0.135499717738201	0.0754219807937554	ZDHHC24	-0.276559166543293	0.025739468697287	-0.215777972027972	0.0843519384534969	0.04826541130509
ZDHHC3	-0.0497526004594433	0.66324879732709	0.0746105160662123	0.512661728	0.8301312943637	ZDHHC3	-0.184627364227009	0.0202165779258597	-0.226590209147738	0.00419766240213294	0.052347004165271	ZDHHC3	-0.327651198444111	0.00771407218770244	-0.324606643356643	0.00859738885121973	0.0172266813101136
ZDHHC4	-0.0171533830276354	0.880730312134613	0.0305258033106134	0.789035102	0.944072630935905	ZDHHC4	0.0592432027097904	0.459656668247741	0.0855530776228478	0.285156406436487	0.612188328325321	ZDHHC4	0.0976308340409831	0.439106345609705	0.113374125874126	0.367638392356034	0.518254240652335
ZDHHC5	0.193789237022114	0.0870358778972409	0.0702775073028238	0.537499995	0.283556597307383	ZDHHC5	-0.169780628868222	0.03295163051948	-0.169654762203626	0.0330835606827747	0.0785904285352542	ZDHHC5	-0.0339949649674356	0.788055969283045	-0.26722027972028	0.0317077689801537	0.829494898359102
ZDHHC6	0.255528765196772	0.0230344440763202	0.0678675754625122	0.551567649	0.120363338161548	ZDHHC6	-0.0205166628928571	0.798051489748705	-0.14461971663868	0.0698394871576232	0.87523239061436	ZDHHC6	0.378975815697215	0.00185114978713385	0.323265877313899	0.00862390566231264	0.00500264049825204
ZDHHC7	0.116853411234692	0.30508973216341	0.163802336903603	0.148931570	0.577573887544102	ZDHHC7	-0.0672649024902626	0.401051539143088	-0.0818853030728523	0.306389050513579	0.556856387029508	ZDHHC7	-0.155259167951722	0.216846728462923	-0.130856643356643	0.298015885386808	0.292258509730057
ZDHHC8	0.231832043575937	0.0397995358579848	0.172224926971762	0.128922490	0.173115289245486	ZDHHC8	-0.19833656516505	0.012485219543869	-0.159054883336021	0.0459187031868804	0.0346686111316181	ZDHHC8	0.00539414462853194	0.965984164187661	-0.103103146853147	0.412826941716425	0.97393475497893
ZDHHC8P1	-0.053519781802404	0.639464244419471	-0.272195649951255	0.015233612	0.817643914414975	ZDHHC8P1	-0.0272009999235219	0.734414501706127	0.0676815887854217	0.39813394739609	0.830654971825877	ZDHHC8P1	0.0240356882199342	0.849270475620783	-0.0911713286713287	0.46917641796745	0.880625658952916
ZDHHC9	-0.106041634246164	0.35230809990761	0.0384615384615385	0.735978000	0.621369153363559	ZDHHC9	0.203474454142062	0.0103411379031755	0.259774537029717	0.000980153754764552	0.029530068062271	ZDHHC9	0.232561326521235	0.0622882185274292	0.27583041958042	0.0264603308963641	0.102326226492783
ZEB2	0.0357036891798209	0.754749640123639	-0.0130720545277507	0.908840352	0.880851318734187	ZEB2	-0.554052610555214	4.30109224600274e-14	-0.493961818399083	4.24254149565356e-11	1.4190521191561e-12	ZEB2	-0.610078452822019	6.85468825135129e-08	-0.610052447552448	1.40456144283116e-07	4.8578512821694e-07
ZER1	-0.0772614000256928	0.498544709287046	-0.07317429406037	0.520828835	0.733638472740651	ZER1	0.351846908399198	5.80577414410754e-06	0.194682201568911	0.0142386091199598	3.89378149337048e-05	ZER1	0.0467333624521593	0.711630013397999	-0.00734265734265734	0.953713151340592	0.7652930493266
ZFAND1	0.125919709183001	0.268827013864325	0.0267283349561831	0.814786624	0.541798727387674	ZFAND1	0.0440483066971218	0.582628696035735	-0.0472208087367988	0.55574581256331	0.716847844283759	ZFAND1	0.18169245455907	0.147472831935856	0.156774475524476	0.211799463052337	0.212167398908835
ZFAND2A	0.138985573275833	0.221866638768086	0.0266309639727361	0.815449647	0.491034600633073	ZFAND2A	0.0846903612666055	0.290059737507362	0.101343483046191	0.205148990889284	0.439253086082959	ZFAND2A	0.327987411870233	0.00764791670449672	0.279326923076923	0.0245501932751773	0.0170999126071194
ZFAND2B	0.106421307763922	0.350576885987823	0.000365141187925998	0.997530923	0.619579094903687	ZFAND2B	-0.363633500913162	2.64924578342042e-06	-0.312873654842567	6.26789410169737e-05	1.91518111772762e-05	ZFAND2B	-0.0266293306039267	0.833227430267685	-0.200043706293706	0.110029405182057	0.867071678873094
ZFAND3	-0.0360850132207025	0.752214086002136	-0.0214216163583252	0.851099623	0.879201527147499	ZFAND3	-0.0781104138134664	0.329298421531028	0.0297235070361236	0.710835687767903	0.482943036755057	ZFAND3	0.255824292817716	0.0396991204557002	0.139335664335664	0.267591511136771	0.0699134385030809
ZFAND5	-0.134754552480002	0.236395889381802	-0.159980525803311	0.158745904	0.50842082429244	ZFAND5	-0.0687715645456293	0.390562184876181	-0.205895417717591	0.00944851530050087	0.546371567140615	ZFAND5	-0.589832902532169	2.33831894997826e-07	-0.604589160839161	1.90516073586866e-07	1.55039985346397e-06
ZFAND6	-0.0219615271925038	0.847655919216049	-0.0169912366114898	0.881659564	0.927777463766188	ZFAND6	-0.369500333887981	1.77174693541243e-06	-0.351187365798216	6.06076356037006e-06	1.33763919245612e-05	ZFAND6	-0.0917207973437106	0.467432866612936	-0.0407342657342657	0.746775575438805	0.5446955155997
ZFAT	-0.0418627391455546	0.714130720997927	-0.0537000973709834	0.637680619	0.85839779992081	ZFAT	0.155591665732231	0.0509224106612778	0.14099775160567	0.0772148023541248	0.112973591739849	ZFAT	-0.285669382678299	0.0210688395332876	-0.273694792346656	0.0273775034627117	0.0407477514480953
ZFC3H1	-0.0635736951214794	0.577792310175452	0.0631450827653359	0.579640771	0.783945689143863	ZFC3H1	0.0145067351151913	0.856439432639781	-0.0670988509314366	0.402217748127337	0.912392220062402	ZFC3H1	0.208167082404601	0.0961092078677774	0.0434440559440559	0.730545304971731	0.147938937539394
ZFHX3	-0.178846544831924	0.11478875841228	-0.0393622200584226	0.730028670	0.336588890122769	ZFHX3	-0.189493603231267	0.0170962403110241	-0.0773900979375013	0.333791701366093	0.0453322460213098	ZFHX3	-0.178010697478227	0.155992291525129	-0.0166958041958042	0.894819387328025	0.222338696025724
ZFHX4	-0.0637581769343654	0.576686751653755	-0.0354430379746835	0.756028467	0.783020235731193	ZFHX4	-0.105286617961758	0.187977357846047	-0.0147088689048712	0.854461057743176	0.316408304142404	ZFHX4	0.0901035274977126	0.475353041618051	0.0951048951048951	0.450152587296485	0.551897344830953
ZFP1	-0.00204998939605355	0.985694461255065	-0.00192307692307692	0.986633528	0.993654977812177	ZFP1	0.287307998152051	0.000252010244133238	0.299081855638981	0.000134919366044327	0.00113103573732153	ZFP1	0.230911534418511	0.0642209810484009	0.267875874125874	0.0312795624722723	0.104957412228023
ZFP14	-0.265239090738899	0.0181569672576259	-0.219182083739046	0.052462409	0.103608914720521	ZFP14	-0.129382775722193	0.105184951004091	-0.017763527341715	0.824680426332895	0.20232524793494	ZFP14	0.306010034599988	0.0131734562673544	0.320367132867133	0.00954581846961879	0.0272651695399399
[illegible]	[illegible]	[illegible]	[illegible]	[illegible]	[illegible]	[illegible]	[illegible]	[illegible]	[illegible]	[illegible]	[illegible]	[illegible]	[illegible]	[illegible]	[illegible]	[illegible]	[illegible]

ZNF780A	-0.2358140033659	0.0364245698627485	-0.276630963972736	0.013830386	0.162950722177209	ZNF780A	0.0672801531258658	0.400944532200928	0.00376506883230492	0.962552631202585	0.556749799201922	ZNF780A	0.627001788078412	2.29606058610443e-08	0.545498251748252	3.85339916993982e-06	1.71870598494436e-07
ZNF780B	-0.230021735622296	0.0414177583188191	-0.189581304771178	0.094242852	0.178233338561707	ZNF780B	0.0912018972083153	0.254426216953497	0.252348035621381	0.00138044274832537	0.397284703487182	ZNF780B	0.604010518922889	9.99086582353925e-08	0.600437062937063	2.39275449269938e-07	6.94026552109085e-07
ZNF781	-0.0183737975566732	0.872313628217851	-0.0284810126582279	0.802874998	0.940812462585487	ZNF781	-0.000520743169525812	0.994818840762711	0.0630645226311244	0.431165744168577	0.99682223145058	ZNF781	0.116415011452898	0.355745777681232	-0.000743006993006993	0.995437909666345	0.437875861559169
ZNF782	0.194961688815271	0.0851055439394419	0.178213242453749	0.115996791	0.279557789077277	ZNF782	0.413763795124672	6.52475220519477e-08	0.332086677208955	2.01943829961314e-05	6.73322242699752e-07	ZNF782	0.589848611877736	2.3361692291779e-07	0.597770979020979	2.76542208191916e-07	1.54941807983277e-06
ZNF784	-0.140935820231659	0.215385160499601	-0.0332521908471276	0.770684169	0.483254419099388	ZNF784	0.108542458849458	0.17461017928507	0.112337569482687	0.159931641511489	0.299587455791066	ZNF784	-0.0996875031130582	0.429479447807466	-0.0269667832167832	0.830807954052948	0.509170960997671
ZNF785	-0.0859470916333289	0.451369743723199	-0.0196932814021422	0.862997746	0.699184742430876	ZNF785	-0.0899971198623005	0.260780862863735	0.0339525540477631	0.671922928586873	0.404497066755528	ZNF785	0.428568031404034	0.000368462527114755	0.542744755244755	4.38329319844327e-06	0.00123999928369145
ZNF786	0.254286753542381	0.023732241573613	0.307594936708861	0.006007654	0.122552551917211	ZNF786	0.445800790546351	4.35995861636229e-09	0.414769110006304	6.01889820024924e-08	5.69268355512268e-08	ZNF786	0.630140123558592	1.86097128818119e-08	0.520205445134239	8.934145372115e-06	1.40757985080908e-07
ZNF787	0.155684979437568	0.170664292848645	0.215579357351509	0.056539610	0.423877455712886	ZNF787	0.200652709750536	0.0114746379308572	0.228192248363905	0.00393028880919232	0.0322910553816999	ZNF787	0.0153505345499137	0.903401357902938	-0.0564685314685315	0.654288701252284	0.924232505037196
ZNF788	0.160086514170183	0.158744186472948	0.067794547224927	0.551996708	0.408904911380829	ZNF788	-0.229807075243732	0.00367638059547095	-0.180990556888808	0.0228591719760129	0.0120067707579599	ZNF788	0.307229254370675	0.0127948741036628	0.175262237762238	0.162248657141224	0.0266052508745583
ZNF789	0.140427693281012	0.217060846364113	0.146859785783836	0.196111549	0.485362285968279	ZNF789	-0.0361314298999497	0.652201440539886	0.0228414175826499	0.775744069282521	0.770867265044896	ZNF789	0.356130057064735	0.00359565887211	0.372027972027972	0.00242265052576007	0.00890836439321852
ZNF79	0.0372799590540711	0.74428555823827	0.0573758519961052	0.614805316	0.874651660690737	ZNF79	0.331173748218514	2.13481668016602e-05	0.254246351115941	0.00126593935138235	0.000125927507471504	ZNF79	0.197806338932491	0.114227721632567	0.134309440559441	0.285363359527749	0.171147459996866
ZNF791	-0.0909731853500313	0.425245206022586	0.00820350535540409	0.942748811	0.680712599908248	ZNF791	0.181113694571653	0.0227650594113591	0.244671666982395	0.00194643653613043	0.0576881959756533	ZNF791	0.582705462237251	3.53165089173046e-07	0.606512237762238	1.71248262170837e-07	2.27768835697886e-06
ZNF792	-0.0671639062804808	0.556454852741383	-0.126387536514119	0.266438517	0.77117791211284	ZNF792	-0.00303123704165942	0.96984746242385	0.107825732440792	0.177490691089773	0.983198886741359	ZNF792	0.0983573017387267	0.435692142192587	0.132473776223776	0.292044918179341	0.515038281603732
ZNF793	-0.105233180219414	0.356012080629889	-0.125827653359299	0.268575718	0.623888616932301	ZNF793	-0.0799659383892651	0.317903472079946	-0.00856459384733821	0.914945734006281	0.470731353735366	ZNF793	-0.11895890614632	0.345258981956375	-0.0117569930069930	0.925867021386127	0.427398472505727
ZNF8	0.372928344482577	0.000712274576856409	0.328554040895813	0.003253090	0.0105681669775439	ZNF8	0.184434403944365	0.0203498458264626	0.277697785911421	0.000411445187689134	0.0526402503393532	ZNF8	0.411112318366713	0.000669112031368879	0.339055278159206	0.00572968569535923	0.00208260108137391
ZNF80	0.0236683965366608	0.835974585510129	0.0710139766526843	0.534000168	0.922254484797018	ZNF80	-0.00543442034427981	0.945970555217192	0.0144495194776096	0.856999590807301	0.968236603889319	ZNF80	-0.197205717836957	0.1153534472049	-0.127928321678322	0.309030579186884	0.172493920277988
ZNF800	-0.0641501189951916	0.574341181319221	0.00394352482960078	0.972504921	0.781458998006427	ZNF800	0.180694432890674	0.0230868672042377	0.184858034086477	0.0200582561277032	0.0583526433376862	ZNF800	0.0853576844997512	0.499003666892045	0.119711538461538	0.341329760782675	0.573870702314565
ZNF804A	0.183246463848051	0.105988526558318	-0.0242845069010398	0.831766656	0.320582884763605	ZNF804A	-0.0841182904197314	0.293341969241822	-0.0535278694958842	0.504151812628144	0.443089223242048	ZNF804A	-0.188643568774557	0.132341902022838	-0.312306734139427	0.0113175370638393	0.193573225391495
ZNF804B	-0.0888786538586686	0.436025351291855	-0.232273574949157	0.039412934	0.688135125905706	ZNF804B	-0.100895960344986	0.207167004290051	-0.154415685273393	0.0527211061536841	0.340622001337844	ZNF804B	-0.432375845909146	0.000322103097945901	-0.509758499382867	1.44325818954045e-05	0.00110255804735842
ZNF805	-0.0778723137054219	0.495144762408601	0.00847127555988316	0.940880626	0.73094582115155	ZNF805	0.210207967618258	0.00802585904486408	0.249001115829562	0.00160560552107066	0.0236409704025516	ZNF805	0.459590686602717	0.000117502972703853	0.384309440559441	0.00169332810111297	0.000455993439648 33
ZNF808	-0.0748290073561032	0.512201572715275	-0.0761927945472249	0.503740824	0.742073511499152	ZNF808	0.074021730630411	0.355319971621648	0.12257847126039	0.124944752184603	0.510023229108128	ZNF808	0.0331416598139392	0.793256602537244	0.0524095730372198	0.678415703378468	0.833907807850872
ZNF81	-0.223684809865605	0.0475183172803715	-0.148198636806232	0.192031020	0.194659751278208	ZNF81	0.00260877306365933	0.974048239471777	0.05765955715186	0.471763017202965	0.985558015316044	ZNF81	-0.0912282472882046	0.469837417718705	-0.0774038461538462	0.53906782777734	0.546943181420719
ZNF813	0.130702893733316	0.250915272129911	0.138607594936709	0.222648934	0.524477092811814	ZNF813	0.232278869727208	0.00331625686324877	0.188573188866083	0.0176521085577412	0.0109797651642504	ZNF813	0.531295802287645	5.27552205209503e-06	0.507298951048951	2.11572515814428e-05	2.82303814063126e-05
ZNF814	-0.0779195565687338	0.494882346278645	-0.0620496592015579	0.586245687	0.730867909550406	ZNF814	0.103465586428106	0.195772355884185	0.187489921777783	0.0183263668014759	0.32664211272683	ZNF814	0.462626061731589	0.000104444267824633	0.373994755244755	0.00228958295062186	0.000410893365520384
ZNF821	0.00639656221108272	0.9553830408808	0.0700097370983447	0.539054254	0.979400082026666	ZNF821	0.100097408593107	0.210803277202612	0.158584699216683	0.0465729736774097	0.345305483877577	ZNF821	0.110859318518189	0.37932053926419	0.151442307692308	0.22790277794001	0.46065039533106
ZNF823	0.0276325906309374	0.808985316751656	0.0467624148003895	0.681781109	0.908888671912646	ZNF823	0.136155357268458	0.0880434777205807	0.228285217708847	0.0039152535669919	0.175629041590193	ZNF823	0.433072322893091	0.000314223795952584	0.460402097902098	0.000135255328854413	0.00107893596949768
ZNF827	-0.162634053879394	0.152133526432732	-0.0861246348588121	0.449640503	0.399481795776231	ZNF827	0.0822559834443477	0.304197248638035	0.203619486170847	0.0102856423714153	0.455528822793739	ZNF827	0.0571135840128034	0.651341663774218	0.145367132867133	0.247271387278722	0.711425812724528
ZNF829	-0.0965563652196939	0.397266689821849	-0.0439873417721519	0.699736507	0.659153084760698	ZNF829	0.203696833583811	0.0102561533697534	0.222493679243274	0.00495717134372339	0.0293328854337673	ZNF829	0.242962478262577	0.0511584556938369	0.277272727272727	0.0256576571234941	0.0867353672155242
ZNF83	0.0340829190901912	0.765555331135559	-0.089508276533593	0.431979343	0.886181266829796	ZNF83	0.228042344747993	0.00395464100219822	0.454555619194224	1.97975412417595e-09	0.0127683165293703	ZNF83	0.647311844012622	5.64747870690496e-09	0.680594405594406	0	4.45034388744195e-08
ZNF830	0.0885677855877051	0.437638386613004	0.00489289191820837	0.965868847	0.688921681823623	ZNF830	0.0831185089123533	0.299137216679255	-0.191880077613717	0.0157252602073683	0.449523806959847	ZNF830	0.0926909465046983	0.462716286233858	-0.0502185314685315	0.690498322390085	0.540485334493308
ZNF831	-0.0589340810332748	0.605912304214397	-0.28868496622145	0.009875033	0.799693208305086	ZNF831	0.0452615294217191	0.57227740040029	0.0364580362161696	0.649265481281944	0.708896981655856	ZNF831	-0.0997181154357029	0.429337067214166	-0.183916083916084	0.142255125638167	0.50903278778 95
ZNF835	-0.273403395142525	0.0147698534623175	-0.23986365702455	0.032340867	0.089716880152861	ZNF835	-0.111991073162004	0.161232184915183	-0.104107576003079	0.192997952285545	0.282263648182628	ZNF835	-0.308190412604038	0.0125030816355626	-0.29943508999919	0.0153860129026418	0.0260945634567568
ZNF836	0.0399290786546285	0.726804040105134	0.25927458617332	0.021287180	0.865374168033191	ZNF836	-0.0165292572321647	0.836686348351819	-0.0502678523210844	0.53050411333645	0.89983617672374	ZNF836	-0.253589707004885	0.0415214120148356	-0.283479020979021	0.0224353616203401	0.0725379725626908
ZNF837	0.0520300739956454	0.648828021090209	-0.033033106134372	0.772154282	0.822650208418626	ZNF837	0.00124807909697968	0.987582586966909	0.0637125708422605	0.426436689608767	0.993275760319069	ZNF837	0.119920405151913	0.34134598115987	0.186013986013986	0.137700534739521	0.423459902708372
ZNF839	0.0817094955728489	0.474070174577149	0.0706669912366115	0.535243221	0.715300573145167	ZNF839	-0.0245356919424848	0.759600023670287	0.0108814623419995	0.892061758204097	0.848609529447781	ZNF839	-0.0905987181128442	0.472920365722601	-0.0881993006993007	0.483834632690731	0.549647998702034
ZNF84	-0.203027484886578	0.0727294781087055	-0.207960077896787	0.066019735	0.254052210885549	ZNF84	0.0979500987934339	0.220807422922974	0.171827092826871	0.0308683752382459	0.357431166447899	ZNF84	0.609653374484024	7.03981817284927e-08	0.641826923076923	1.94774980990036e-08	4.98142954119123e-07
ZNF843	-0.163132798976088	0.150863730454254	-0.0957887049659202	0.400270175	0.397531263022835	ZNF843	0.0416279356024652	0.603533834080175	0.109207104516631	0.171970007175088	0.73335475449911	ZNF843	-0.316210245112306	0.0102845332043493	-0.290777972027972	0.0190934903213221	0.0220260577966275
ZNF844	-0.129247779830455	0.256275619968371	-0.0847127555988316	0.457127479	0.530190294114481	ZNF844	0.242090245005332	0.00217973316265851	0.322055866057765	3.68285757907187e-05	0.00757577638749163	ZNF844	0.246237419090799	0.0480111986388632	0.311756993006993	0.0117572455695 21	0.0822197101801295
ZNF85	-0.0117064872661895	0.918443508148957	0.083033106134372	0.466123553	0.960906459171079	ZNF85	0.0811270873080718	0.310904520099388	0.203540536511307	0.0103158192856045	0.462896110935765	ZNF85	0.375376116517746	0.0020618286915024	0.406075174825175	0.000868939661125676	0.00548494744948261
ZNF853	-0.0155927373501346	0.891512576808668	-0.0367332035053554	0.747437806	0.949036943124648	ZNF853	-0.0342834100183066	0.668913415340442	-0.0644016925315672	0.421440926396113	0.783028929638045	ZNF853	-0.123755079742363	0.326017096580383	-0.150611888111888	0.230485747855559	0.408160222541849
ZNF862	-0.083243729905602	0.465781159181159	-0.108179162609542	0.341903511	0.709612730099581	ZNF862	0.318126765643036	4.63364659908372e-05	0.317732257208297	4.740902569615e-05	0.000250309371740409	ZNF862	0.37926254842569	0.00183522874473763	0.414160839160839	0.000670818794543892	0.00496424874189716
ZNF876P	0.139981771070706	0.21853895212304	0.148247322229796	0.312933220	0.486905868546748	ZNF876P	-0.0845288125978884	0.290984125431239	-0.0116542191209244	0.88444795700792	0.440399354586412	ZNF876P	-0.217161345236629	0.0822739503105009	-0.155532245059977	0.216029421133457	0.129658698368614
ZNF879	-0.242125224641142	0.0315668284353805	-0.267599805258033	0.017364340	0.148085562807153	ZNF879	0.231911511409026	0.00336768210549954	0.362546464372285	2.85179702790018e-06	0.0111268893686731	ZNF879	0.288466751021613	0.0197880156413324	0.277534965034965	0.025513959334409	0.0386026315914133
ZNF880	-0.0885273675616315	0.43784835491602	-0.0145813047711782	0.898359008	0.689181399094315	ZNF880	0.065825724433784	0.411225862811323	0.0883292754258396	0.269756086961008	0.566963838415072	ZNF880	0.31299113867657	0.0111301746001331	0.263854895104895	0.033984310661978	0.0235432316868283
ZNF883	-0.131567829678332	0.247765631742578	-0.0649951314508277	0.568564597	0.521087692960943	ZNF883	0.336650915091323	1.52558913244505e-05	0.29851899686405	0.000139095672622384	9.33402988644512e-05	ZNF883	0.478558541422546	5.52465570091698e-05	0.377185314685315	0.00208762799600123	0.000234514343902561
ZNF90	0.501540520877713	2.49648887429288e-06	0.40309152872444	0.000258776	0.000110801832673682	ZNF90	0.0147589548089585	0.853970983893995	-0.0934042739301183	0.243087297480147	0.91135131731106	ZNF90	0.0975296856150794	0.439582902562365	0.0132870036527281	0.916336681121908	0.518631687383819
ZNF91	-0.223235761791489	0.0479773325390102	-0.13468841285297	0.236101788	0.195931381985658	ZNF91	0.121843366998237	0.127240564944889	0.119157202652773	0.135906894126737	0.234789896826241	ZNF91	0.0898670763662364	0.476516991719637	0.257954545454545	0.0383064521460967	0.55294020382919
ZNF92	0.107191899886133	0.347079475990572	0.315579357351509	0.004778526	0.616815336161518	ZNF92	0.198928548853388	0.0122197655312393	0.145062397458963	0.0689787208605944	0.034018733317725	ZNF92	0.631868600614638	1.65595523959894e-08	0.681861888111888	0	1.25620449882448e-07
ZNF93	0.379354180838233	0.000565035655085255	0.456937682570594	2.840498950	0.00893548023014583	ZNF93	0.494939668180441	3.83222342748013e-11	0.294592676598927	0.000171757532915201	7.44997183737161e-10	ZNF93	0.713256380845486	2.6153079701885e-11	0.756424825174825	0	2.33592489739937e-10
ZNF99	-0.0427238759243181	0.708511328599419	0.0341940170447308	0.764813183	0.856117855390965	ZNF99	0.0768917128307593	0.336923406463965	0.0287833807579965	0.719592798706703	0.490724676661519	ZNF99	0.355463843295377	0.00366336333576833	0.398426573426573	0.00110383810648356	0.00905576847650705
ZNFX1	-0.176130876332496	0.120495189898388	-0.163218111002921	0.150401565	0.347651913320251	ZNFX1	-0.0343273455127716	0.668514166410026	-0.0395902993498216	0.621387681005376	0.782800021424625	ZNFX1	0.0804958472155004	0.523850247975462	0.124388111888112	0.322695167165199	0.597006778029313
ZNHIT1	0.0032308374685521	0.977455850071479	-0.0102726387536514	0.928321667	0.990273629890058	ZNHIT1	0.308595931179096	7.98292651806864e-05	0.227314319355486	0.0040748510256569	0.000408692094542947	ZNHIT1	0.488611434826747	3.6360355777143e-05	0.499912587412587	2.88126835162597e-05	0.000162068098498582
ZNHIT2	-0.152853803115646	0.178671334317382	-0.190920155793574	0.091908540	0.434229743196016	ZNHIT2	0.00731056821743683	0.927362298544683	-0.0102835819419722	0.89795934708066	0.956724007632817	ZNHIT2	-0.291684642742959	0.0183972807250189	-0.34138986013986	0.00560499554850039	0.0362498742208131
ZNHIT3	-0.0977678152237255	0.391342867225012	-0.177848101265823	0.116755055	0.653981583935657	ZNHIT3	-0.0741332446252498	0.354593655691416	-0.117120262383497	0.142774211142956	0.509448197384031	ZNHIT3	0.296583364287681	0.0164406952108556	0.203583916083916	0.103779923028008	0.0329411282189995
ZNHIT6	0.319434882600498	0.00411078545939692	0.0178675754625122	0.875599475	0.0369644783414997	ZNHIT6	0.126586679635334	0.112984963733846	-0.0775862790243296	0.332564074376156	0.21388721669307	ZNHIT6	0.354532989139712	0.00375986031509301	0.299606643356643	0.0156298742062516	0.00926365584016533
ZNRD1	0.12073558395157	0.289190239036817	0.0861976630963973	0.449255118	0.562586071948336	ZNRD1	0.15472226783131	0.0522471856541133	0.163924251378861	0.0395826538383705	0.115359881013699	ZNRD1	-0.136910381969628	0.276807005135668	-0.194143356643357	0.121089011039826	0.357688458346375
ZNRF1	-0.104138901936652	0.361063764966702	-0.0443524829600779	0.697364222	0.628528715665593	ZNRF1	0.368250777839224	1.93154013894414e-06	0.319213944902303	4.34974453214673e-05	1.44024519876338e-05	ZNRF1	0.212802635908008	0.0887676820955576	0.206031468531469	0.0996237079589402	0.138413755963988
ZNRF2	-0.173211948432835	0.126868830293704	-0.159298928919182	0.160545768	0.360363596947993	ZNRF2	0.0219900811422852	0.783892964219091	-0.0197791615990419	0.805163463341366	0.866338664381887	ZNRF2	0.159637435055582	0.204002914532696	0.179370629370629	0.152511714467399	0.277776912256878
ZNRF3	-0.123785164901352	0.277091447835239	-0.117575462512171	0.301422301	0.550287644872513	ZNRF3	-0.132436749915758	0.097155924855489	-0.25640803305966	0.00114616628803961	0.189836613944975	ZNRF3	-0.298377765323598	0.0157701098615315	-0.22145979020979	0.0763461105535367	0.0318038788221064
ZNRF4	-0.00465412466352306	0.967528986023404	-0.0599890187354516	0.599465929	0.985174279017918	ZNRF4	0.037208866608904	0.64253653434798	0.143200399781896	0.0726583015166063	0.76430171204667	ZNRF4	-0.443542872871818	0.000215142812442178	-0.413723776223776	0.000680373529361597	0.000772273331705028
ZP1	-0.0625132959041532	0.584165930158981	0.0781679836451405	0.493503628	0.787729853868162	ZP1	-0.0748489427460628	0.349954279747134	0.0401303399365338	0.616633695450959	0.504416889179876	ZP1	-0.122654448989872	0.330371419602328	-0.232736013986014	0.0622622299671231	0.412601427915827
ZP2	0.052409093025067	0.646440399763792	0.155519147102068	0.171125902	0.821272938585259	ZP2	0.0814375845127538	0.309050157986102	0.171839524155475	0.0308560708237021	0.460921371396965	ZP2	-0.160117538791424	0.202628107108041	-0.149132392292458	0.235757204957014	0.276262257056704
ZP4	0.145642417110162	0.200296977818146	0.205120578372935	0.069766029	0.46316957065656	ZP4	0.035141243277779	0.661135036456339	-0.0112663200769781	0.888268564002553	0.777311317285684	ZP4	-0.250616297033942	0.0440528625535074	-0.191346153846154	0.126621607418219	0.0763328068785898
ZPBP	0.0151555012666987	0.894536991055624	0.0361540039897076	0.751755621	0.950031815853691	ZPBP	0.101804147816238	0.203086537611002	0.202099014842209	0.010880698737176	0.335802331365057	ZPBP	0.274723749406063	0.0267792565491622	0.226594540275128	0.0695077295570729	0.0499321778986069
ZPBP2	0.167120639936235	0.140994095130722	0.216779380857215	0.054990432	0.383732105889008	ZPBP2	0.0722352452336908	0.367082240255736	0.0875214970218178	0.274177578834016	0.522369665683363	ZPBP2	0.15067601413301	0.230888879934239	-0.206383373956529	0.0990573123547268	0.308029173921485
ZPLD1	0.0273007024973912	0.811236837226783	0.0754603264690153	0.508638697	0.910024511750401	ZPLD1	-0.0724641024567169	0.365562158840297	-0.0571910152527287	0.475377916373106	0.521220519226291	ZPLD1	-0.238803387325816	0.0553963061022663	-0.249344405594406	0.0454288365459868	0.0927342572698582
ZRANB1	0.0659937428725363	0.563367882067087	0.112731256085686	0.321882346	0.77543948580436	ZRANB1	-0.26631488306938	0.000718977118007353	-0.346679931878976	8.10932155597581e-06	0.00287497149055503	ZRANB1	-0.411044253484042	0.000670628644504241	-0.488199300699301	4.64097649626172e-05	0.000208648030718944
ZRANB2	0.157885673204038	0.164624800514448	-0.0129990262901655	0.909347926	0.416877144673711	ZRANB2	0.22910682762379	0.00378461286253051	0.0304400110442107	0.704186797059296	0.0123100216959694	ZRANB2	0.562360810771898	1.08734948178757e-06	0.47923951048951	6.61275910940615e-05	6.57075477687454e-06
ZRANB3	0.215132429650806	0.0569075686736058	0.225925024342746	0.045482045	0.217554218756689	ZRANB3	0.0719871613461412	0.368734425884484	-0.0222314003698199	0.781580657082392	0.523824384726176	ZRANB3	0.263393305850042	0.034011848290878	0.218269230769231	0.0807632481186722	0.0612625918818428
ZRSR2	-0.154773712463166	0.173212212050001	-0.0659931840311587	0.562630986	0.426983313822515	ZRSR2	0.0345729418828128	0.666284105070533	0.0672144651662872	0.401405556416904	0.781093375850007	ZRSR2	0.163917510962364	0.191978604373183	0.174606643356643	0.163844100438114	0.264121686900494
ZSCAN1	-0.034846287713885	0.760460230172098	-0.0317417867956436	0.781242462	0.883729819252741	ZSCAN1	0.0358178264241691	0.65502554626345	0.129103610279213	0.105944164763015	0.772892079199096	ZSCAN1	-0.293616217690978	0.0176032592903112	-0.256523449696494	0.0391427556732475	0.0349200415557694
ZSCAN10	0.252573750967319	0.0247240927231853	0.283195446881217	0.011439551	0.125737491869159	ZSCAN10	0.0276413712748322	0.730279547144751	0.105695830886671	0.186257356673604	0.82770710231699	ZSCAN10	-0.23069623581601	0.0644767333424958	-0.184702797202797	0.140534027893	0.105286691406064
ZSCAN12	0.116123782944626	0.308140217870607	0.113145082765336	0.320100409	0.580805987710366	ZSCAN12	0.19963686240918	0.0119086657775156	0.0953391490336219	0.233419985992807	0.0332936274812547	ZSCAN12	0.410757494465633	0.000677052476443674	0.400262237762238	0.00104273847045394	0.00210363193416856
ZSCAN12P1	-0.249183177844	0.0267913328904167	-0.308424467048476	0.005685239	0.133216043425805	ZSCAN12P1	0.091034764798388	0.255301332100036	0.0348470696332896	0.663798419933563	0.398245850191634	ZSCAN12P1	-0.368767893774121	0.00250512262654411	-0.345410839160839	0.00504248120511301	0.00649486622979532
ZSCAN16	0.0297003259426141	0.794993436270429	-0.095715676728335	0.400630776	0.90167336750525	ZSCAN16	-0.142009287307168	0.0750944486422461	-0.106320980104494	0.183652017147214	0.154381775488973	ZSCAN16	-0.0989240498439063	0.43303898699061	-0.266870629370629	0.031938155576944	0.512425042852163
ZSCAN18	-0.24629524135136	0.0286655570955579	-0.239118792599805	0.034043207	0.13916331463818	ZSCAN18	0.187363992377719	0.0184061793077426	0.203873533239532	0.0101890653870715	0.0482703363326982	ZSCAN18	0.162379475795738	0.196239527078499	0.306993006993007	0.0131627611847857	0.269040871789382
ZSCAN2	-0.0611249849146776	0.59255864013671	0.0061587147030185	0.957024448	0.792562709449101	ZSCAN2	0.135937115101409	0.0885587139891517	0.202941013161066	0.0105475324227427	0.176446528629901	ZSCAN2	0.269236938974715	0.0301009312929033	0.198951048951049	0.112016076633286	0.0552757349146559
ZSCAN20	0.293851390100014	0.0085767096858318	0.271275559883155	0.015841573	0.0620446833088447	ZSCAN20	0.263861657818561	0.000808337621354571	0.172876542077684	0.0298442187079308	0.00318184639921694	ZSCAN20	0.0487118764203798	0.699988191610919	0.0221153846153846	0.860932330033764	0.754946750382271
ZSCAN21	0.185031490243815	0.102571609933759	0.271226874391431	0.015860960	0.313791384573841	ZSCAN21	0.156589055228491	0.0494370423825123	0.14694884674713	0.0654068936902506	0.110266046796953	ZSCAN21	0.515505943357692	1.11071215511682e-05	0.468356643356643	0.000100451966037251	5.58612236970805e-05
ZSCAN22	0.114082191129956	0.316780606545307	0.125876338851022	0.268389415	0.589230399346664	ZSCAN22	0.237552529003863	0.00265212747346499	0.226526489371089	0.00420862705541616	0.00899349176395109	ZSCAN22	0.173072749877201	0.167983503717652	0.0798513986013986	0.526278779955719	0.236691485435348
ZSCAN23	0.00482526607379705	0.966335644869615	0.0272882181110029	0.810976793	0.984707423578301	ZSCAN23	-0.0498351337353062	0.534053330106425	-0.0620331219570627	0.438754038964742	0.677129601050915	ZSCAN23	-0.0443445806535275	0.725773612867918	0.00126748251748252	0.992120109428234	0.777255011283685
ZSCAN29	0.0227310951879121	0.842384991639777	0.0665043816942551	0.559603269	0.924977257642211	ZSCAN29	0.114334230728547	0.152590006281751	0.00322808095760143	0.96789041782058	0.270451720000677	ZSCAN29	0.470884178401992	7.53757252751619e-05	0.437718531468531	0.000304675332064252	0.000308564139152201
ZSCAN4	-0.126651486249972	0.266032345387564	-0.239358551247221	0.033624755	0.539134359072484	ZSCAN4	-0.0111162960312173	0.889746918680929	0.0213749422879662	0.78979570015745	0.934252026060338	ZSCAN4	-0.298849558863837	0.0155976926265645	-0.299868881118881	0.0155358907928671	0.0315082914288036
ZSCAN5A	0.0539367888069767	0.6368530620504	0.069620253164557	0.541318950	0.816099511548063	ZSCAN5A	0.315788201997373	5.30434468259955e-05	0.192998188963916	0.0151165741363834	0.000281258599097516	ZSCAN5A	-0.000996419398209209	0.993714717422038	-0.0400786713286713	0.750729191561689	0.994874545259776
ZSWIM1	-0.357407779524229	0.00122244360637555	-0.36952288218111	0.000868399	0.01574055315531	ZSWIM1	-0.00630401044060377	0.937341427527757	0.164958694532787	0.0383358374047098	0.962542150869367	ZSWIM1	-0.0345160175847803	0.784884826139552	-0.0399912587412587	0.751245493181546	0.826677850788248
ZSWIM2	-0.173380641319575	0.126493614068496	-0.210914079163295	0.062067501	0.359918097978235	ZSWIM2	0.0973708698360349	0.223562816154934	0.149346777089514	0.0610861354742406	0.360654700789724	ZSWIM2	-0.201606234512367	0.107300244011162	-0.23951048951049	0.0548687185455488	0.162356310591439
ZSWIM3	-0.172631954670559	0.128165366506958	-0.20547711781889	0.069374263	0.36269203301969	ZSWIM3	0.0765912210758471	0.338820619605732	0.158354991986914	0.0468954304825007	0.492632413060568	ZSWIM3	-0.209354578320029	0.0941848285796183	-0.0784099476658222	0.534698847255751	0.145459121485846
ZSWIM4	-0.0704361778338021	0.537341226321409	-0.0173807205452775	0.878965327	0.759008285629848	ZSWIM4	0.0359885320136129	0.65348767514858	0.00847482766253363	0.915833895071027	0.771743415195674	ZSWIM4	-0.314651250736918	0.0106868350547309	-0.274344405594406	0.027309491343615	0.0227667890288397
ZSWIM5	-0.281680796807486	0.0119074988291044	-0.178188899707887	0.116047223	0.0779306789682418	ZSWIM5	-0.424759469149958	2.66032246920482e-08	-0.397855653668414	2.25995741214327e-07	2.94861749898018e-07	ZSWIM5	-0.371193200691697	0.00233341026684818	-0.379020979020979	0.00197883303829516	0.00610710608884663
ZSWIM6	0.0986049624766786	0.387280224885602	-0.108666017526777	0.339725492	0.65055446643345	ZSWIM6	-0.0916962351767359	0.251849940274689	-0.00736736499185969	0.926799531229468	0.394030543082946	ZSWIM6	-0.118953686072282	0.34528030191008	-0.130987762237762	0.29752879100419	0.427402019895107
ZSWIM7	-0.233026110613037	0.0387612247308435	-0.277434274586173	0.013548716	0.17017081081612	ZSWIM7	-0.541437658836961	2.05133995176074e-13	-0.426882743586766	2.2288451903758e-08	6.08134758645978e-12	ZSWIM7	-0.435534745216198	0.000287756917059 18	-0.526267482517483	9.28931543782973e-06	0.00100036778731471
ZUFSP	0.109724730451131	0.335738024772075	0.128140214216164	0.259822829	0.606656631235535	ZUFSP	0.428538301563858	1.93993328068132e-08	0.313244599408176	6.13673383142074e-05	2.22241267562807e-07	ZUFSP	0.564855276734762	9.51119636383169e-07	0.475961538461538	7.51049074062784e-05	5.79682388911426e-06
ZW10	0.404148122142573	0.000220941275171693	0.242624148003895	0.031448119	0.00441266692312981	ZW10	0.193283091259704	0.0149648015134312	0.0691327487208474	0.388072378939797	0.0405090421794248	ZW10	0.334475208284726	0.00646498468456747	0.298080180089692	0.0158796343138085	0.0147414635516966
ZWILCH	0.526053231712708	6.39607958152766e-07	0.621324245374878	7.699154378	3.40058231084554e-05	ZWILCH	0.780533263797945	0	0.493367013585576	4.51261855623009e-11	0	ZWILCH	0.933459113650564	0	0.898120629370629	0	0
ZWINT	0.762935279222321	4.44089209850063e-16	0.795740019474197	0	1.54091164779203e-13	ZWINT	0.729465852658262	0	0.61547390046697	7.63281007582866e-18	0	ZWINT	0.901007394385493	0	0.831774475524475	0	0
ZXDA	-0.186247122800657	0.100294241576381	-0.109983203129061	0.334593945	0.309609130610837	ZXDA	-0.179978014927036	0.0236458169127109	-0.0610488797209005	0.446065717734216	0.0594536314344452	ZXDA	0.0481157461586278	0.703488805299816	-0.0281031468531469	0.82378527416912	0.758157316460853
ZXDB	-0.258570262094244	0.0213990276145944	-0.194571567672834	0.085774329	0.114939298515571	ZXDB	0.138043444473206	0.0836852865166002	0.239326029860488	0.002457449773867	0.168513907820528	ZXDB	0.320565783705407	0.00922950315638271	0.193968531468531	0.121429265565532	0.0200803469330036
ZXDC	-0.105620410138537	0.354234969743489	-0.1019717624148	0.370440901	0.622691367850683	ZXDC	0.132096469829308	0.0980257645371099	0.13034592235443	0.102598378404708	0.191171009017817	ZXDC	0.320198053862911	0.00931478054515522	0.373339160839161	0.00233319101698036	0.0202430625340898
ZYG11A	0.646749959021866	1.20374821221958e-10	0.387669662743636	0.000415766	1.43420073140234e-08	ZYG11A	0.153043108028509	0.0548866901374983	0.125335053349925	0.116619582956729	0.119973301417986	ZYG11A	0.380445810484478	0.00177081803862134	-0.0352533633118853	0.78040327635601	0.00481702440386073
ZYG11B	-0.170574763541879	0.132845598704507	-0.160516066212269	0.157342313	0.370726047207869	ZYG11B	-0.29099496411462	0.000207828242969767	-0.390186169484512	4.0209446376524e-07	0.000956290415664436	ZYG11B	0.0255097465876414	0.840144737618005	0.0561625874125874	0.656043570518775	0.873367391859284
ZYX	0.18609899662844	0.100567927928438	0.0227117818889971	0.842239494	0.309769892317185	ZYX	-0.211328950126309	0.00768867003148504	-0.316186677437137	5.18397033715158e-05	0.02276063921525	ZYX	-0.281873147081521	0.0229192313621586	-0.370979020979021	0.00249642083310423	0.0436844208993164
ZZEF1	-0.13798979312694	0.225228331328233	-0.0678188899707887	0.551853670	0.495226896832662	ZZEF1	0.0308768799552218	0.700143738371508	-0.0920487070601772	0.250024047418488	0.805489746036144	ZZEF1	0.114599962262309	0.363346546353957	-0.153671328671329	0.221069884319595	0.445479407811416
ZZZ3	0.219808216046266	0.0516026124033644	0.0338364167478092	0.766767848	0.204855717806866	ZZZ3	0.0122467031677662	0.878617454815602	-0.0364063140584733	0.64973006887177	0.926104851892547	ZZZ3	0.234767114522225	0.0597777506930357	0.222115384615385	0.0754628871975292	0.0988965430424107

EZH2_E2F1_94_gene_Signature_ACC
MCM7
RRM2
DNMT1
CDCA7
POLD1
MCM2
KIAA0101
CLSPN
FANCG
PKMYT1
MCM6
FANCC
POLE2
FBXO5
POLD3
MCM3
MSH2
E2F8
GMNN
NOLC1
NASP
MCM4
CDC6
ATAD5
SNRPD1
ZNF367
MXD3
ILF3
H2AFZ
PCNA
ATAD2
CDC25A
H2AFV
SUV39H1
CTDSPL2
E2F1
TOPBP1
HNRNPD
MTF2
SMC3
EED
MAZ
PRKDC
CASP8AP2
GEN1
UBR7
SMC6
RAVER1
POLA1
HOXC10
EZH2
STMN1
CDK1
ARHGAP11A
E2F7
SASS6
SETD8
MCM8
FANCD2
RBL1
CBX3
CD3EAP
TMPO
RASAL2
RAD51
CDT1
HMGN2
POLA2
INTS7
LIG1
WEE1
KIF15
USP1
PLK4
TIPIN
MELK
CDC45
SMC2
YWHAQ
CASP2
WDR67
RPS19
NUP155
ESPL1
NCBP1
DLEU2
CCDC150
KNTC1
PRIM1
ANP32E
ANP32A
SLBP
RPA2
FUS

Supplementary Table 5.

Overall Survival

TCGA

	HR	95% CI	Log Rank
**Univariate**			
**EZH2_E2F1_Metagene**	**HR**	**95% CI**	**Log Rank**
high vs low	10,18	3,418 to 30,32	5,00E-07
**Prognosis group**			
C1A vs C1B	8,856	2,642 to 29,69	2,00E-05
**MKi67**			
high vs low	10,31	3,514 to 30,24	2,00E-07
**Weiss score**			
[4-9] vs [0-3]	3,131	0,8856 to 11,07	6,00E-02
**ENSAT-rank**			
ENSAT [3-4] vs [1-2]	6,699	2,716 to 16,52	3,00E-06
**Multivariate**			
**EZH2_E2F1_Metagene + Prognosis**	**HR**	**95% CI**	**Log Rank**
high vs low	7,182	2,247 to 22,96	1,00E-07
**EZH2_E2F1_Metagene + MKI67**			
high vs low	4,013	1,098 to 14,67	1,00E-07
**EZH2_E2F1_Metagene + Weiss score**			
high vs low	20,9765	4,7876 to 91,91	2,00E-05
**EZH2_E2F1_Metagene + ENSAT**			
high vs low	8,711	2,334 to 32,51	5,00E-08
**EZH2_E2F1_Metagene + Prognosis + MKi67 + Weiss + ENSAT**			
high vs low	6,207	1,18843 to 32,420	7,00E-06
**Adjusted**			
**EZH2_E2F1_Metagene (~Prognosis)**	**HR**	**95% CI**	**Log Rank**
high vs low	6,161	1,981 to 19,16	6,00E-04
**EZH2_E2F1_Metagene (~MKI67)**			
high vs low	4.319	1,046 to 17,83	4,00E-02
**EZH2_E2F1_Metagene (~Weiss)**			
high vs low	11.44	2,362 to 55,39	4,00E-04
**EZH2_E2F1_Metagene (~ENSAT)**			
high vs low	7,931	2,095 to 30,03	1,00E-03

Cochin

	HR	95% CI	Log Rank
**Univariate**			
**EZH2_E2F1_Metagene**	**HR**	**95% CI**	**Log Rank**
high vs low	4,045	1,55 to 10,56	2,00E-03
**Prognosis group**			
C1A vs C1B	11,86	2,72 to 51,69	4,00E-05
**MKi67**			
high vs low	5,631	2,008 to 15,79	3,00E-04
**Weiss score**			
[4-9] vs [0-3]	9,803	1,308 to 73,47	6,00E-03
**ENSAT-rank**			
ENSAT [3-4] vs [1-2]	14,88	4,885 to 45,3	1,00E-09
**Multivariate**			
**EZH2_E2F1_Metagene + Prognosis**	**HR**	**95% CI**	**Log Rank**
high vs low	2,675	1,007 to 7,108	3,00E-05
**EZH2_E2F1_Metagene + MKI67**			
high vs low	2,317	0,813 to 6,604	3,00E-04
**EZH2_E2F1_Metagene + Weiss score**			
high vs low	2,948	1,469 to 28,90	3,00E-04
**EZH2_E2F1_Metagene + ENSAT**			
high vs low	2,816	0,9908 to 8,002	2,00E-09
**EZH2_E2F1_Metagene + Prognosis + MKi67 + Weiss + ENSAT**			
high vs low	1,326	0,3984 to 4,415	1,00E-08
**Adjusted**			
**EZH2_E2F1_Metagene (~Prognosis)**	**HR**	**95% CI**	**Log Rank**
high vs low	2,738	1,029 to 7,286	4,00E-02
**EZH2_E2F1_Metagene (~MKI67)**			
high vs low	2,219	0,769 to 6,396	1,00E-01
**EZH2_E2F1_Metagene (~Weiss)**			
high vs low	3,106	1.171 to 8.242	2,00E-02
**EZH2_E2F1_Metagene(~ENSAT)**			
high vs low	2,6	0,8646 to 7,819	8,00E-02

Michigan

	HR	95% CI	Log Rank
**Univariate**			
**EZH2_E2F1_Metagene**	**HR**	**95% CI**	**Log Rank**
high vs low	2,3722	0,8492 to 6,627	9,00E-02
**Prognosis group**			
C1A vs C1B	3,532	1,251 to 9,974	1,00E-02
**MKi67**			
high vs low	0,861	0,3215 to 2,306	8,00E-01
**Weiss score**			
[4-9] vs [0-3]	3,111	0,8668 to 11,16	0,07
**ENSAT-rank**			
ENSAT [3-4] vs [1-2]	2,558	0,9217 to 7,102	6,00E-02
**Multivariate**			
**EZH2_E2F1_Metagene + Prognosis**	**HR**	**95% CI**	**Log Rank**
high vs low	1,8981	0,6637 to 5,429	2,00E-02
**EZH2_E2F1_Metagene + MKI67**			
high vs low	2,3641	0,8374 to 6,674	2,00E-01
**EZH2_E2F1_Metagene + Weiss score**			
high vs low	2,381	0,5585 to 10,154	1,00E-01
**EZH2_E2F1_Metagene + ENSAT**			
high vs low	2,142	0,7485 to 6,127	6,00E-02
**EZH2_E2F1_Metagene + Prognosis + MKi67 + Weiss + ENSAT**			
high vs low	1,183	0,2866 to 4,885	1,00E-02
**Adjusted**			
**EZH2_E2F1_Metagene (~Prognosis)**	**HR**	**95% CI**	**Log Rank**
high vs low	1,8594	0,6258 to 5,525	3,00E-01
**EZH2_E2F1_Metagene (~MKI67)**			
high vs low	2,245	0,7916 to 6,367	1,00E-01
**EZH2_E2F1_Metagene (~Weiss)**			
high vs low	1,676	0,4947 to 5,676	4,00E-01
**EZH2_E2F1_Metagene(~ENSAT)**			
high vs low	2,305	0,7627 to 6,968	1,00E-01

Supplementary Table 6.

EZH2_E2F1_94_gene_Signature_ACC	EZH2_E2F1_gene_Signature_CrPc_XU_et_al	Intersection
MCM7	ARL6IP1	ATAD2
RRM2	ATAD2	CDC6
DNMT1	BIRC5	CDK1
CDCA7	BNIP2	POLE2
POLD1	BRIX1	SMC2
MCM2	BTG3	TMPO
KIAA0101	CCNA2	
CLSPN	CCNE2	
FANCG	CDC6	
PKMYT1	CDK1	
MCM6	CENPK	
FANCC	CHEK1	
POLE2	CKS2	
FBXO5	CNOT6	
POLD3	DSCC1	
MCM3	FANCI	
MSH2	FOXM1	
E2F8	GGH	
GMNN	GLOD4	
NOLC1	GPSM2	
NASP	GUCY1B3	
MCM4	H1F0	
CDC6	H1FX	
ATAD5	H2AFX	
SNRPD1	HMGB2	
ZNF367	HMMR	
MXD3	IER5	
ILF3	KIF23	
H2AFZ	MMD	
PCNA	MND1	
ATAD2	MRPL20	
CDC25A	MTFR2	
H2AFV	NCAPG	
SUV39H1	NCAPG2	
CTDSPL2	NDC1	
E2F1	NME1	
TOPBP1	NMU	
HNRNPD	NUF2	
MTF2	PA2G4	
SMC3	PBK	
EED	PLK1	
MAZ	POLE2	
PRKDC	PPIH	
CASP8AP2	PRC1	
GEN1	RFC2	
UBR7	RFWD3	
SMC6	RRM1	
RAVER1	SMC2	
POLA1	SMC4	
HOXC10	SNRPA1	
EZH2	SPAG5	
STMN1	TACC3	
CDK1	TMPO	
ARHGAP11A	TRIP13	
E2F7	TYMS	
SASS6	WDR34	
SETD8		
MCM8		
FANCD2		
RBL1		
CBX3		
CD3EAP		
TMPO		
RASAL2		
RAD51		
CDT1		
HMGN2		
POLA2		
INTS7		
LIG1		
WEE1		
KIF15		
USP1		
PLK4		
TIPIN		
MELK		
CDC45		
SMC2		
YWHAQ		
CASP2		
WDR67		
RPS19		
NUP155		
ESPL1		
NCBP1		
DLEU2		
CCDC150		
KNTC1		
PRIM1		
ANP32E		
ANP32A		
SLBP		
RPA2		
FUS		

Supplementary Table 7.
